# Decoding Prolonged Residence Time of 5‑HT_2A_ Receptor Antagonists: Insights from Ritanserin Derivatives

**DOI:** 10.1021/acs.jcim.6c00316

**Published:** 2026-05-11

**Authors:** Szymon K. Kordylewski, Kinga Kurowska, Krystyna Nędza, Dorota Satała, Ryszard Bugno, Sabina Podlewska

**Affiliations:** † Maj Institute of Pharmacology Polish Academy of Sciences, Smetna 12, 31-343 Krakow, Poland; ‡ Department of Comparative Biochemistry and Bioanalytics, Faculty of Biochemistry, Biophysics and Biotechnology, Jagiellonian University, Gronostajowa 7, Kraków 30-387, Poland

## Abstract

The lifetime of a ligand–receptor complex has been suggested to play an important role in compound activity and sustained pharmacological action. However, due to the multitude of other factors considered during optimization, this aspect is often neglected in the early stages of drug design and development. Consequently, data on ligand residence time (RT) remain limited, and the molecular determinants underlying prolonged binding are not fully understood. In this study, we focused on ritanserin, a known 5-HT_2A_ receptor (5-HT_2A_R) antagonist with an RT of 68 min. By modifying its structure, we synthesized a series of derivatives with varying RT, which was determined in vitro. Comprehensive molecular modeling analyses, including docking and molecular dynamics simulations, enabled us to propose a set of key receptor–ligand contacts contributing to prolonged binding. Both conventional molecular dynamics and enhanced sampling approaches (metadynamics and random-accelerated molecular dynamics) were applied to the native and mutant receptors, providing in-depth insights into the determinants of long ligand occupancy at the 5-HT_2A_R. The study indicated that prolonged RT is associated with, for example, stable and geometrically optimized π–π interactions with a hydrophobic aromatic cluster (Trp336^6.48^ and Phe340^6.52^, in particular). To our knowledge, this represents the first such a comprehensive study of its kind, offering novel hypotheses that may guide the design of new 5-HT_2A_R ligands with prolonged duration of action.

## Graphical abstract

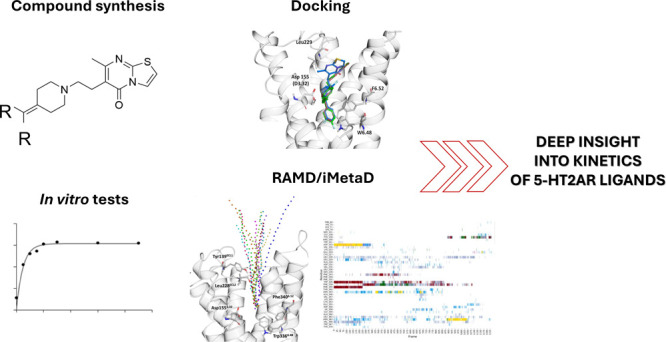

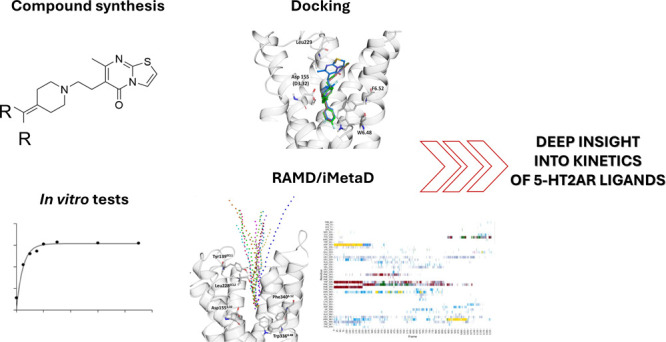

## Introduction

It has long been recognized that the duration of ligand binding to a biological target can critically influence pharmacological outcomes.^1,2^ This principle was encapsulated in the maxim *corpora non agunt nisi fixata*, emphasizing that a drug must remain bound to its protein target to elicit the desired therapeutic effect. Despite this early insight, most ligand discovery efforts have historically focused on equilibrium binding parameters, such as the inhibition constant (*K*_*i*_), or on functional readouts including *IC_50_* and *EC_50_* values. In recent years, however, increasing attention has shifted toward the kinetic dimension of drug–target interactions, in particular the concept of ligand residence time (RT).^3–6^ RT reflects the lifetime of the ligand–target complex and, in some cases, provides a more reliable predictor of in vivo efficacy than equilibrium affinity alone, as previously described.^7^ Nevertheless, in the context of G protein-coupled receptors (GPCRs), the role of ligand binding kinetics in drug optimization remains relatively underexplored. This is partly due to the limited number of kinetic studies performed for central nervous system (CNS)-GPCR targets, which are often technically challenging. Therefore, the primary aim of this study was not to prolong RT itself, but rather to gain insight into the molecular determinants underlying this phenomenon and, in the longer term, to explore whether these factors may influence the pharmacological effects of drugs acting in the CNS. Moreover, it offers a mechanistic framework to rationalize divergent pharmacological outcomes observed among ligands with similar affinities but distinct kinetic profiles.^8^

The scarcity of experimental data directly addressing RT has substantially limited our understanding of the molecular determinants underlying the prolongation. Current hypotheses are based on the energetic and topological barriers to ligand dissociation, the conformational flexibility of the target protein, the role of solvation and desolvation processes, compound lipophilicity, as well as discrete noncovalent interactions within the binding interface.^7^

Subtle structural variations at the ligand–protein interface, even among closely related analogues, can give rise to markedly different RTs.^9^ To probe the molecular origins of such differences and indicate molecular determinants for prolonged RT, molecular dynamics (MD) simulations constitute the standard computational approach. Classical MD provides atomistic resolution of biomolecular motions and binding-site dynamics; however, its applicability is limited by the intrinsic time scales it can access, typically extending only from nanoseconds to microseconds. These windows are orders of magnitude shorter than the experimentally observed RT of many drug-like molecules, which frequently persist for minutes to hours. This mismatch highlights a central methodological challenge: spontaneous ligand unbinding events are rarely captured in unbiased simulations. To overcome this limitation, a diverse set of enhanced sampling techniques has been developed. These approaches typically rely on either biasing the energy landscape, accelerating barrier crossing, or guiding the system along plausible dissociation pathways, thereby enabling reconstruction of unbinding trajectories and associated free-energy profiles. The suite of enhanced sampling techniques applied to studies of ligand–receptor kinetics comprises, among others, such methods as scaledMD, acceleratedMD, random acceleration MD (RAMD), targetedMD, steeredMD, metadynamics (MetaD), replica-exchange MD (REMD), transition path sampling, and weighted ensemble simulations.

In this study, we addressed the problem of RT from the perspective of a representative of class A GPCRs: serotonin 5-HT_2A_R. This receptor is broadly expressed in the CNS, with high abundance in cortical regions and plays a central role in modulating cognition, perception, and mood. Dysregulation of 5-HT_2A_R signaling has been implicated in a spectrum of neuropsychiatric conditions, including schizophrenia, depression, and anxiety disorders, making it an intensively pursued pharmacological target.^10^ Among a wide range of known 5-HT_2A_R ligands, ritanserin represents a well-characterized antagonist with high affinity for the receptor and documented therapeutic potential in sleep disorders, anxiety, and as an adjunct in schizophrenia treatment. Its long RT of 68 min provides a valuable starting point for investigating the molecular determinants underlying prolonged ligand binding to the 5-HT_2A_R.

Here, we used ritanserin as a template structure to synthesize a series of structurally related derivatives and systematically characterized their binding affinity and RT at 5-HT_2A_R. To complement the experimental data, we employed an integrative computational approach combining molecular docking, classical MD, and advanced enhanced sampling techniques, including RAMD and MetaD. This strategy enabled us to obtain a detailed description of ligand behavior within the 5-HT_2A_R binding pocket and elucidate the molecular determinants governing RT at this receptor. To the best of our knowledge, this work represents the first such comprehensive study of a congeneric ligand series at 5-HT_2A_R that integrates such a broad range of experimental and computational methodologies. The insights gained not only advance the fundamental understanding of ligand–receptor kinetics at 5-HT_2A_R, but also provide a framework for the rational design of ligands with optimized kinetic profiles, which might have important implications in their therapeutic potential.

## Results and Discussion

### Compound Design and Synthesis

The design of ritanserin derivatives was aimed at obtaining compounds with a high affinity for the 5-HT_2A_R and diversified RTs. Because of the limited available data on structural determinants governing RT, the design strategy was guided by compounds with confirmed activity reported in the original patent.^11^

To simplify the molecular framework and facilitate SAR analysis, the investigated series was designed to be symmetrical with both aromatic rings bearing identical substituents. Compounds **1**, **2**, and **12** were directly derived from the structures disclosed in the patents.

Subsequent modifications, including variation of fluorine substitution and the introduction of CF_3_ and OMe groups, were also inspired by structural motifs described therein and were intended to systematically probe steric and electronic effects. Additional analogues were rationally designed to further systematically explore the influence of substituent properties on receptor affinity and RT within the defined chemical scaffold.

All the compounds (**1**–**12**) share a “symmetric” scaffold in which both phenyl rings carry identical substituents. The synthetic route involved a three-step sequence (Scheme 1). Initially, a Grignard reaction between the corresponding bromo derivative and ethylpiperidine-4-carboxylate bearing a *tert*-butoxycarbonyl (Boc)-protected amino group furnished intermediates **12a–l**. Subsequent dehydration and Boc-deprotection using trifluoroacetic acid (TFA) afforded amines **14a–l**, which were subjected to *N*-alkylation to yield the final products **1–12**. All target compounds were obtained in high chemical purity (>95%) and subsequently evaluated in vitro.

**Scheme 1:**
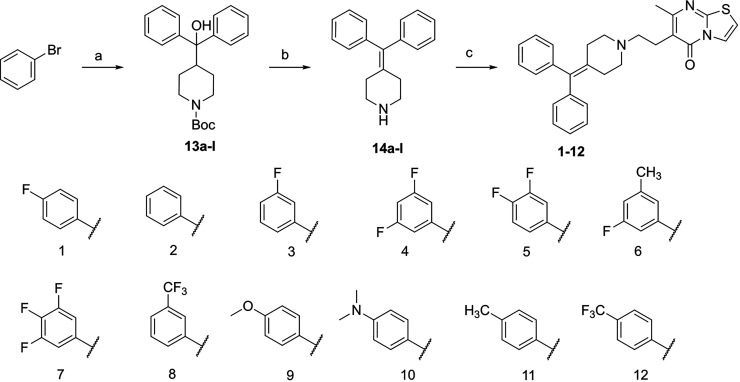
Three-Step Synthetic Route Leading to the Preparation of Derivatives **1**–**12** ^a^

a (a) Mg, THF, and 1-(*tert*-butyl)-4-ethylpiperidine-1,4-dicarboxylate; (b) TFA and DCM; and (c) *n*-BuOH, TEA, KI, and reflux.

### In Vitro Tests and Molecular Modeling Studies

To dissect both the equilibrium and kinetic aspects of receptor engagement (as well as structure–activity relationship (SAR) and structure-kinetics relationship), we evaluated the synthesized compounds at the 5-HT_2A_R in an in vitro assay in terms of their binding affinity, kinetics, and the kinetic stability of the ligand–receptor complexes, quantified in terms of RT (Table 1). In parallel, we performed molecular docking (Glide, Schrödinger) into the crystal structure of the 5-HT_2A_R in complex with risperidone (PDB ID: 6A93), selected for its close structural similarity to the studied series.

**Table 1:**
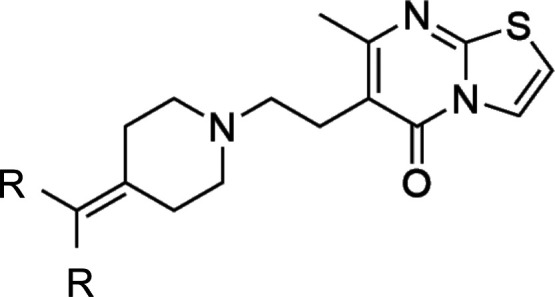

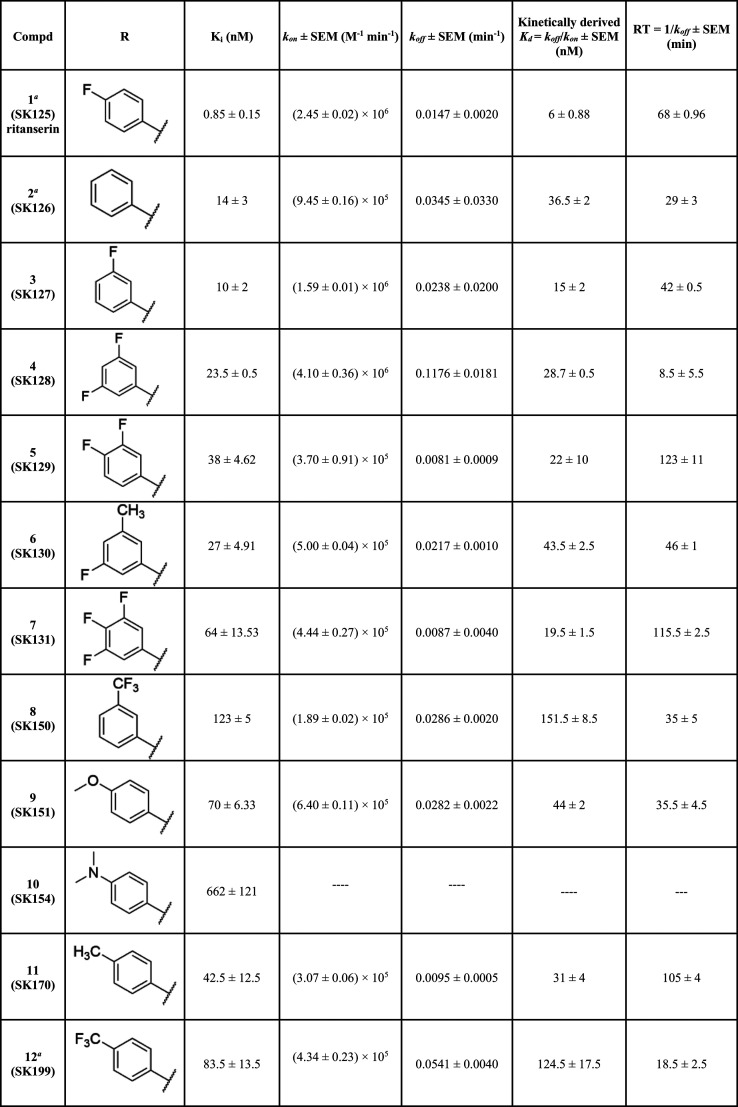
General Structure of the Compounds Examined in the Study Together with the Outcome of the In Vitro Examination of Their Affinity and Kinetics at 5-HT_2A_R

a Compounds **1**, **2**, and **12** are derived from patent EP0110435A1.

The obtained data showed that compound **1** exhibited the highest binding affinity and the longest RT (*K*_*i*_ = 0.85 nM; RT = 68 min). In contrast, compound **2**, lacking fluorine substitution, displayed an over 14-fold lower binding affinity and a 2.3-fold shorter RT (*K*_*i*_ = 14 nM; RT = 29 min). Compound **3**, bearing fluorine at the 3-position, showed an intermediate profile, with *K*_*i*_ = 15 nM and RT = 42 min. Docking analyses revealed nearly identical binding poses for these ligands (Figure 1a), suggesting that distinct molecular determinants may govern equilibrium affinity and kinetic stability. Although all compounds generally occupy the same region of the binding pocket, subtle differences in their binding poses can be visually discerned. For instance, compounds **1** and **2** are almost perfectly aligned within the 5-HT_2A_R binding site, whereas in compound **3**, the thiazolo-pyrimidine moiety adopts a slightly altered geometry. This change enables the formation of an additional hydrogen bond with Leu229^ECL2^. A comparable binding mode and corresponding interactions are also observed for compounds **8** and **9**; however, compound **9** does not form interactions with Leu229^ECL2^. Overall, all compounds exhibited good to moderate affinity toward 5-HT_2A_R, with *K*_*i*_ values ranging from 6.85 nM to 123 nM (except for compound **10** with a K_i_ of 662 nM) and short to long RT spanning 8.5–123 min.

**Figure 1:**
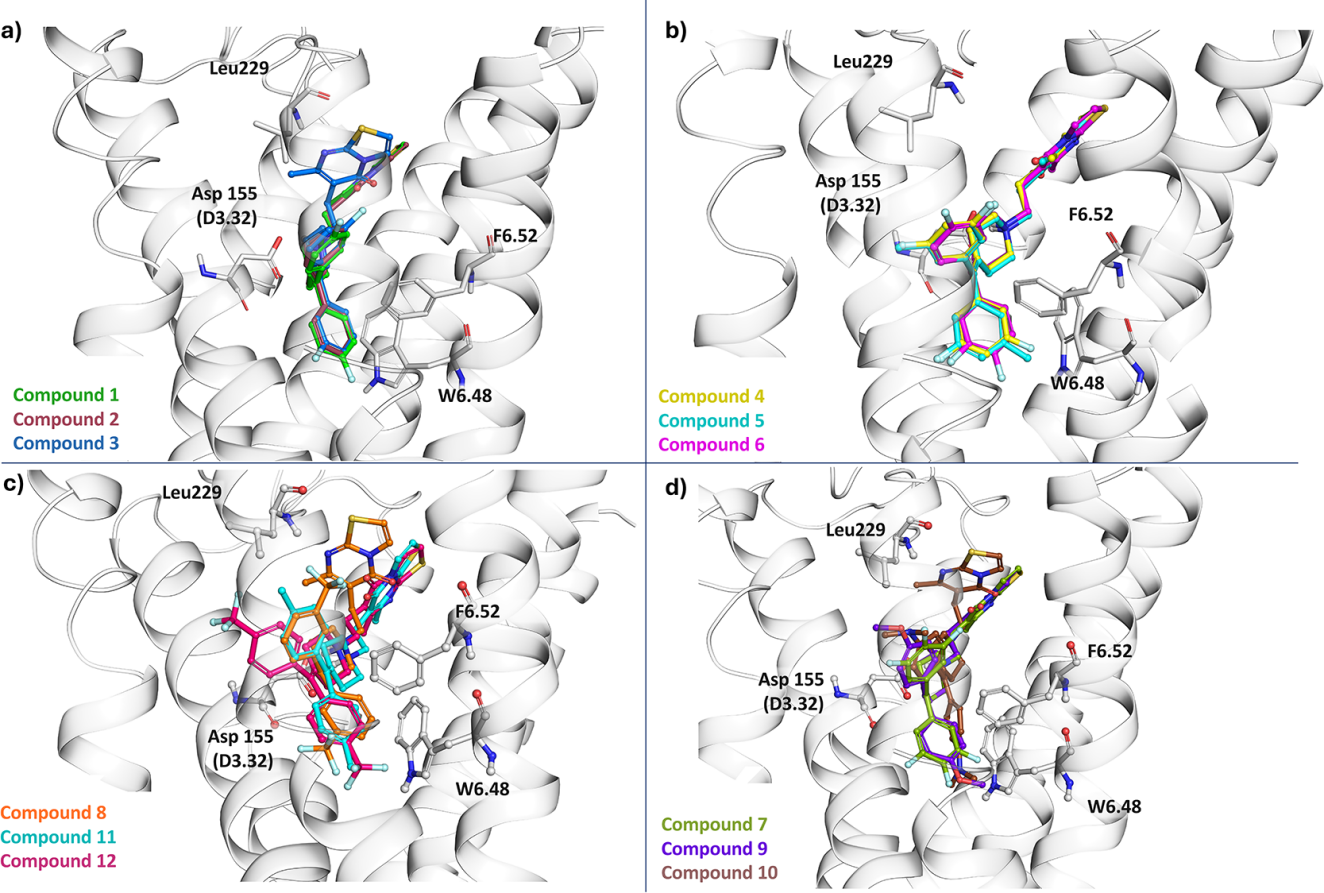
Binding poses of the studied ligands obtained from molecular docking. (a) Compounds **1–3** exhibit nearly identical orientations of the proximal moiety located within the orthosteric binding pocket (OBP), with differences observed in the distal region interacting with Leu229 ^ECL2^. (b) Compounds **4**–**6** display analogous binding orientations. (c) Compounds **8**, **11**, and **12** show altered positioning due to steric hindrance introduced by the CF_3_ group in **8** and **12** and the CH_3_ group in **11**. (d) Compounds **7**, **9**, and **10**; note the deviation of compound **10** caused by the bulky NMe_2_ substituent.

Introduction of two fluorine atoms at positions 3- and 5- (compound **4**) or at positions 3- and 4- (compound **5**) resulted in over 25- and 40-fold reduction in affinity, respectively, relative to compound **1** (*K*_*i*_ = 23.5 and 38 nM, respectively), whereas replacement of fluorine with a methyl group at position 5- (compound **6**) led to *K*_*i*_ of 27 nM (Figure 1b). The diminished affinities observed for these derivatives, as well as for compound **7** (*K*_*i*_ = 64 nM), likely reflect steric constraints introduced by the substituents. This interpretation is further supported by compounds **9**, **10**, and **12**, which bear bulkier groups at position 4- and show substantially reduced affinities (*K*_*i*_ = 70 nM, 662 nM, and 83.5 nM) (Figure 1c). Because of its relatively low binding affinity, compound **10** was not subjected to kinetic studies (a threshold of *K*_*i*_ = 200 nM was applied for further kinetic characterization). In contrast, the RT varied considerably. While compound **4** displayed a very short RT (8.5 min), compounds **5** and **6** exhibited significantly prolonged RTs (123 min and 46 min, respectively). These observations suggest that the fluorine atom at position 4- plays a crucial role in stabilizing the ligand–receptor complex, whereas in compound **6**, the methyl substituent at position 5- likely counteracts the unfavorable kinetic effects associated with fluorine at position 3-. The binding poses obtained in molecular docking are presented in Figure 1, whereas detailed interaction of compounds with the receptor in the form of the 2D ligand–protein interaction diagrams can be found in the Supporting Information (Figure S1).

### Classical MD

To gain more detailed insights into ligand binding, we performed 2000 ns MD simulations using the Desmond engine (Schrödinger Suite). Although conventional MD cannot directly capture ligand unbinding due to the extended time scales required, it nonetheless provides valuable information on ligand stability and conformational behavior within the receptor binding pocket. To verify the robustness of the obtained results, additional MD simulations were performed for selected compounds with a shorter duration of 500 ns but with increased temporal resolution, yielding 10,000 frames per simulation.

All simulations employed the OPLS4 force field and the TIP3P water model, with the POPC membrane constructed based on the OPM database. Overall, most compounds maintained stable binding modes, as reflected by low RMSD values relative to their initial pose throughout the trajectories (Figure S3a).^9^ The largest deviations were observed for compounds **4**, **9**, **11**, and **12** (mean RMSD = 1.75 Å, 0.95 Å, 1.29 Å, and 1.07 Å, respectively), whereas the remaining ligands exhibited lower RMSD values, ranging from 0.40 Å (compound **3**) to 0.83 Å (compound **10**). Notably, compounds **4** and **12**, which display short RTs, also show higher RMSD values, suggesting reduced stability of compound fitting in the binding site.

In contrast, the increased RMSD observed for compounds **9** and **11** may be associated with the presence of bulkier substituents at position 4, which could introduce steric perturbations and disturb their stability within the binding pocket.

To further evaluate the structural dynamics of the receptor, the distances of the so-called ionic lock between residues Arg^3.50^ and Glu^6.30^ were monitored (Supporting Information, Figure S3c), which ranged from 5.55 Å (compound **7**) to 7.08 Å (compound **3**). Additionally, the transmembrane helix 5 (TM5) bulge was quantified by measuring the distance between C_α_ atoms of residues Ser242^5.46^ and Gly369^7.41^, which serves as a structural descriptor of the TM5 protrusion (Supporting Information, Figures S3b).^12,13^ For the majority of the compounds, the corresponding distances were highly comparable, whereas a noticeably shorter distance was observed for compound **12**. Importantly, despite the associated constriction of the putative exit channel in this case, compound **12** still exhibited a low RT. This suggests that TM5 bulge narrowing alone is not a determining factor governing ligand dissociation kinetics.

Having established the global stability of the complexes, we shifted our focus to the specific molecular determinants within the orthosteric site. MD simulations validated the docking-derived binding poses (Figure 2a), the thiazolopyrimidinone scaffold positioned itself within the extended or secondary binding pocket (EBP),an antechamber adjacent to the OBP, while the two phenyl rings penetrated deeper into the orthosteric cavity. Throughout the trajectories, a persistent salt bridge was observed between the protonated nitrogen of the piperidine ring and Asp155^3.32^. Notably, it was present in more than 85% of simulation frames for each compound (Figure 2b and Supporting Information, Figures S2, S4, and S5). This interaction, known to stabilize ligands of 5-HT_2A_R, aligns with structural evidence from crystallographic and cryo-EM studies.^14^ Across all studied complexes, one phenyl ring consistently intercalates between Trp336^6.48^ and Phe340^6.52^, anchoring the ligand within a hydrophobic cleft. Trp336^6.48^, the so-called toggle-switch residue, plays a pivotal role in the activation mechanism of aminergic GPCRs.^14^ In agonist-bound receptors, activation requires an outward displacement of TM6 and a concerted inward movement of TM3 and TM7, all structurally coupled to the reorganization of the PIF motif and the rotation of Trp336^6.48^.^14^ By forming dual T-shaped π–π interactions with Trp336^6.48^ and Phe340^6.52^, the studied ligands effectively restrict the toggle switch in an inactive conformation, thereby providing a clear structural rationale for their antagonistic pharmacological profiles. A π–π interaction between one of the phenyl rings and Trp336^6.48^ was observed in more than 90% of frames for most ligands. Exceptions were compounds **4**, **6**, **7**, and **10**, for which a greater contribution of hydrophobic interactions was noted. Overall, a markedly weaker propensity to form interactions with this residue was observed for compounds **4** and **7**. Similarly π–π interactions with Phe340^6.52^ were significantly reduced for compound **4** (shortest RT) and compound **10** (lowest affinity).

**Figure 2:**
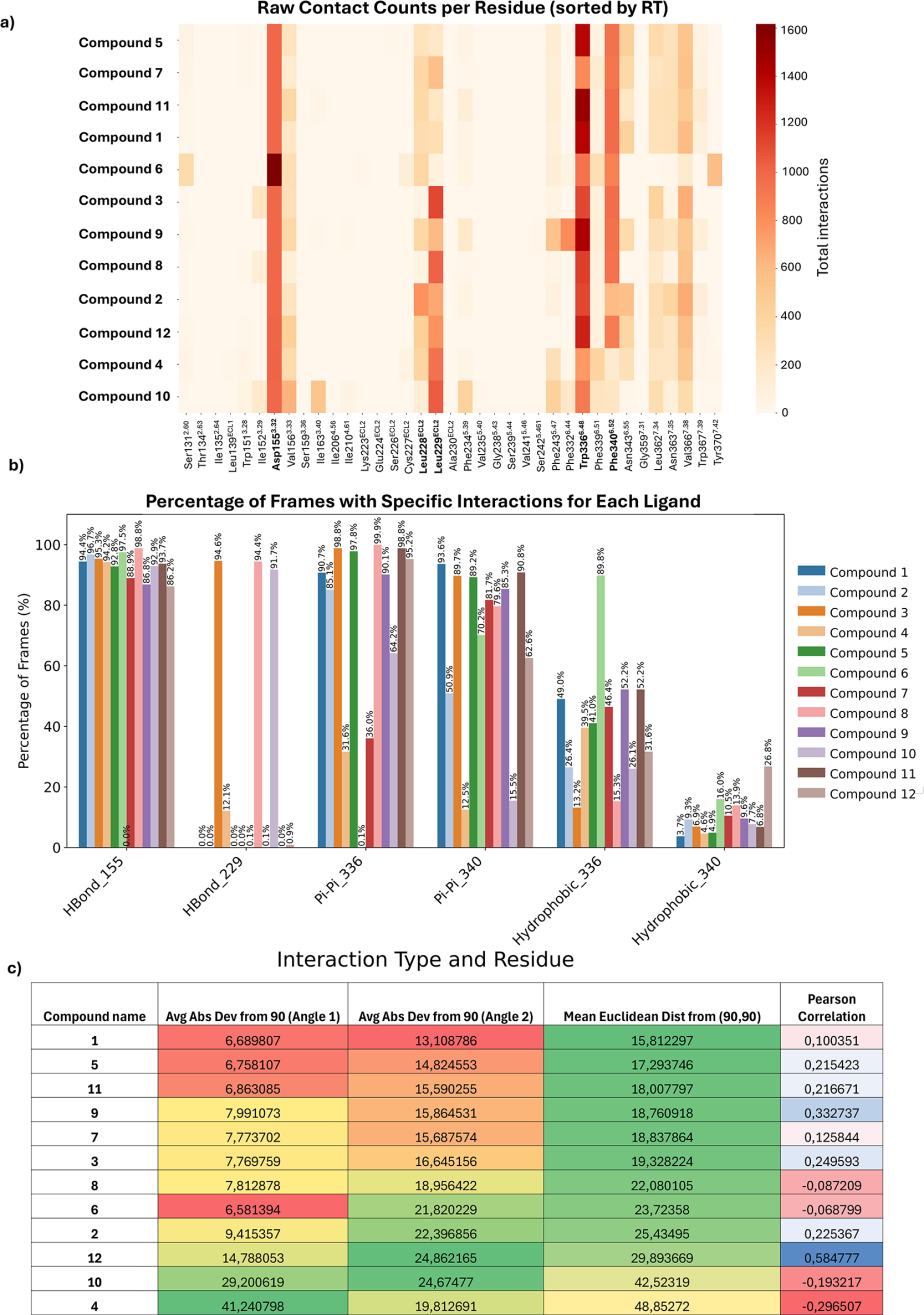
(a) Analysis of the frequency of contacts between the ligand and individual amino acid residues during the simulations. (b) Percentage of a given type of interaction with individual amino acid residues for the analyzed compounds. (c) Mean absolute deviation of the interaction angle from 90°, Euclidean distance from 90°, and Pearson correlation between these two contacts.

To better characterize the geometric constraints governing key ligand–protein interactions, we quantified the relative orientation (angles and distances) of the ligand phenyl rings within the hydrophobic cleft with respect to the aromatic side chains of Trp336^6.48^ and Phe340^6.52^ (Figures S6–S9).

For each interaction, the deviation from the ideal T-shaped π–π stacking geometry was evaluated by calculating the mean absolute deviation of the interaction angle from 90° (Figure 2c). To further assess the cooperative integrity of the dual Trp336^6.48^/Phe340^6.52^ interaction system, we computed the Euclidean distance of the angle pair (relative to Trp336^6.48^ and Phe340^6.52^) from the optimal reference point (90°, 90°), corresponding to idealized orthogonal π–π interactions. In addition, Pearson correlation analysis was applied to evaluate the degree of angular interdependence between these contacts.

Comparison across the compound series revealed a clear relationship between interaction geometry and experimental RT. Compounds exhibiting longer RTs (**1**, **5**, **7**, and **11**) consistently maintained geometries closer to the ideal π–π stacking arrangement, whereas rapidly dissociating ligands (e.g., **4** and **12**) and the low-affinity compound **10** displayed significantly more distorted interaction patterns. To further probe side-chain dynamics, two dihedral angles, (C–C_α_–C_β_–C_γ_) and (C_α_–C_β_–C_γ_–C_δ2_), were monitored for both critical residues (Supporting Information, Figures S10–S18). The Trp336^6.48^ rotameric state remained remarkably stable across all trajectories, with mean dihedrals averaging 103.77° and 150.36° (Figure S19). This rigid conformation aligns with previously reported values, structurally corroborating the compounds’ antagonistic mode of action.^13^

In stark contrast, Phe340^6.52^ exhibited stable dihedral angles only when bound to compounds **1** and **3**, maintaining strict conformational rigidity within the π–π stacking interface. For the remaining ligands, multiple discrete dihedral clusters were identified (Supporting Information, Figure S19). This pronounced conformational heterogeneity disrupts optimal ring–ring alignment, likely attenuating the overall π–π interaction energy. Compound **8,** in particular, demonstrated near-continuous interconversion between these states, highlighting heightened side-chain plasticity and reduced geometric stabilization at this locus.

Expanding our structural analysis to the extracellular vestibule, we evaluated the dynamics of Leu229^ECL2^, a residue located within the extracellular loop 2 (ECL2; Supporting Information, Figures S10, S11, S16, and S19). Previous studies suggest that for agonists like LSD, this residue acts as a critical kinetic “lid”, sterically impeding ligand egress to prolong RT.^15^ However, across our antagonist series, we observed substantial conformational variability at this site (Supporting Information, Figure S19). This flexibility indicates weak ligand-ECL2 coupling, suggesting that the thiazolopyrimidinone scaffold does not effectively exploit the ECL2 lid mechanism to hinder dissociation. Despite this localized plasticity, the overall binding mode remains robustly anchored by supplementary hydrophobic contacts with residues Val156^3.33^, Leu228^ECL2^, Leu229^ECL2^, Val362^7.34^, and Val366^7.38^. Finally, to contextualize the MD-derived binding modes within established structural frameworks, we performed a comparative analysis by superimposing representative snapshots from the final stages of the MD simulations with available experimental structures of the 5-HT_2A_R in complex with structurally related ligands, including risperidone (PDB ID: 6A93), zotepine (PDB ID: 6A94), aripiprazole (PDB ID: 7VOE), and pimavanserin (PDB ID: 8ZMG) (Supporting Information, Figures S20–S23).

The structural overlays reveal a high degree of spatial correspondence between key aromatic features of our compounds and those observed in the experimentally resolved complexes. In particular, the phenyl ring of our ligands occupies a region analogous to that of the fluorinated aromatic moiety of risperidone, indicating conserved engagement of this subpocket. A similar topological alignment is observed with zotepine, where the two phenyl rings of our derivatives effectively recapitulate the spatial footprint of its tricyclic core, suggesting a comparable steric complementarity within this region of the binding site.

In contrast, aripiprazole adopts a more deeply inserted binding mode within the orthosteric pocket, consistent with its elongated and conformationally flexible scaffold, which enables penetration into regions not accessed by our compounds. Finally, comparison with pimavanserin highlights a conserved aromatic anchoring motif, with one of the phenyl rings of our ligands closely overlapping the position of its fluorinated aromatic group.

These observations demonstrate that key interaction patterns inferred from the simulations obtained in our study are consistent with experimentally determined ligand–receptor complexes.

### Generation of Unbinding Pathways and Calculation of RT

To predict RT, we employed the protocol implemented in Desmond (Schrödinger Suite),^16^ which combines two complementary approaches for exploring ligand unbinding pathways, accelerating dissociation events, and estimating RTs.

The first approach, RAMD, assumes that unbinding is driven primarily by ligand motion. In RAMD, a defined force (15 kcal·mol^–1^·Å^–2^ in our case) is applied for a fixed interval (0.24 ps) to the ligand’s center of mass, which has been previously docked into the receptor binding site. The direction of the applied force is random. The ligand’s displacement is monitored, and if it exceeds a predefined threshold (0.05 Å), the force vector is maintained; otherwise, its direction is reassigned randomly. Each RAMD sampling run begins with a constant force of 15 kcal·mol^–1^·Å^–2^, which increases linearly by 5 kcal·mol^–1^·Å^–2^ per nanosecond until the ligand unbinds. The unbinding event was considered to occur when the minimum distance between any protein atom and any ligand atom exceeded 8 Å. To prevent structural distortion of the protein during the process, a weak restraining force (*K* = 0.01 kcal·mol^–1^·Å^–2^) is applied to the protein Cα atoms. During the RAMD stage, 30 egress trajectories are generated (Figure 3a) and subsequently clustered as described in the original protocol,^16^ allowing identification of the predominant dissociation pathway corresponding to the lowest free energy path. The second stage involves infrequent metadynamics (iMetaD) simulations, in which an additional biasing potential acts on selected degrees of freedom known as collective variables (CVs). In our case, the CVs were derived from the main RAMD trajectory and defined as S, the progress along the unbinding path, and Z, the distance from the path. From ten independent iMetaD simulations, the RT was estimated by fitting the cumulative distribution function of the escape times to an exponential distribution, as described by Salvalaglio et al.^17^ The Kolmogorov–Smirnov (KS) test was used to verify that the sampled distribution follows an exponential behavior, with statistical uncertainties estimated using bootstrapping.

**Figure 3:**
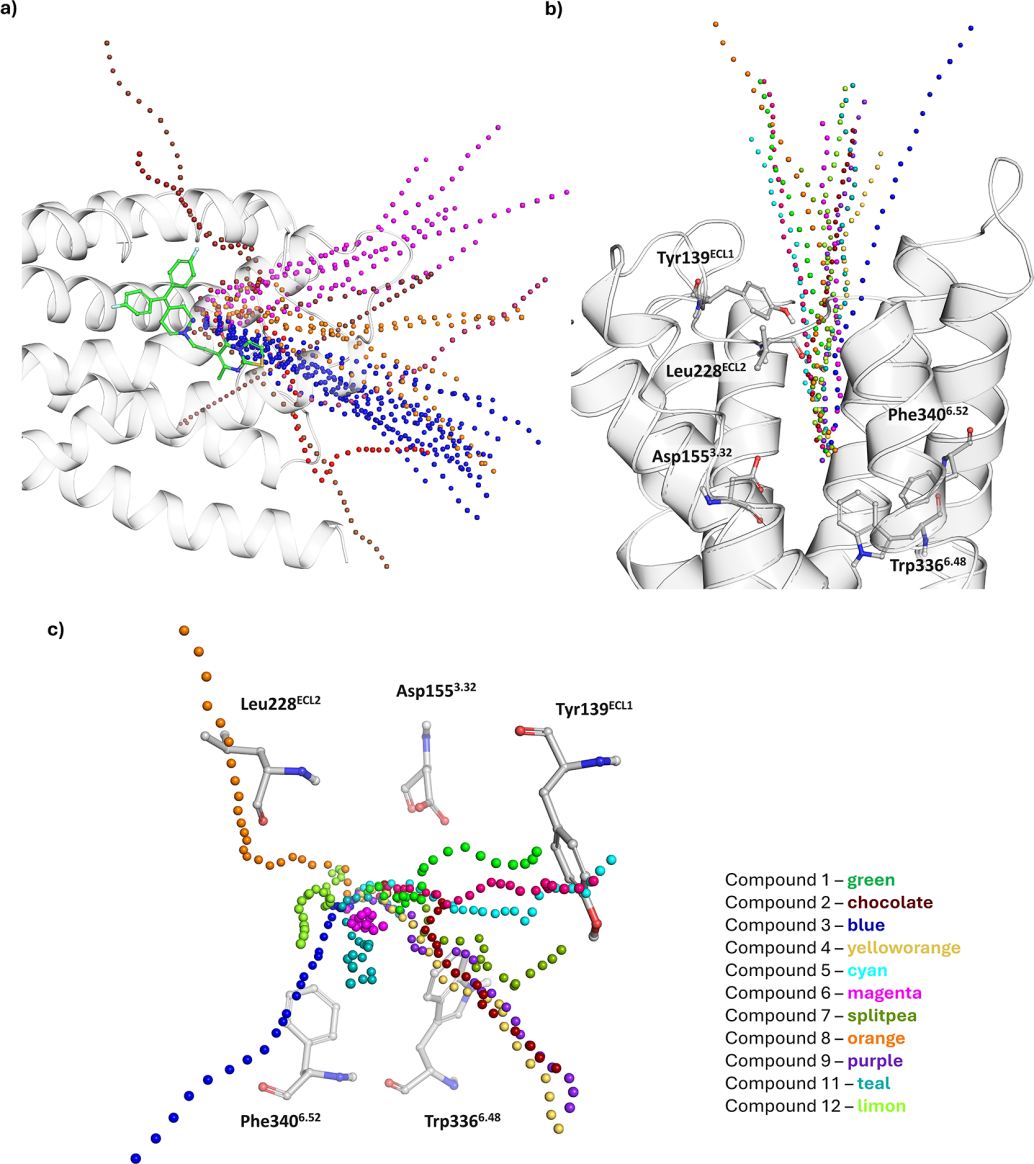
(a) Thirty unbinding pathways obtained for compound **1**. Individual clusters are distinguished by different colors, with the most highly populated cluster shown in **blue**. Notably, several trajectories correspond to clearly nonphysical unbinding routes, such as pathways passing through the protein core or the membrane; however, these pathways are not statistically significant and were therefore not considered further. (b) Comparison of unbinding pathways for the individual compounds shown in a side view. (c) Comparison of unbinding pathways for the individual compounds shown in a top view.

### RAMD Simulations

The exit routes observed for compounds **3** and **8** differ from those of the remaining compounds for which dissociation occurs predominantly in the vicinity of Tyr139^ECL1^. As will be demonstrated by more detailed iMetaD simulations, this apparent difference disappears upon the inclusion of protein flexibility effects. In this respect, RAMD does not explicitly account for protein conformational flexibility and primarily probes the lowest energy, sterically favorable unbinding pathways.

As noted above, for each compound, 30 independent egress trajectories were generated, from which the main egress path was identified through clustering (Figure 3a).^16^ In the early stages, all compounds exhibited interaction patterns (Figure S24) consistent with those observed in conventional MD simulations, dominated by contacts with Asp155^3.32^, Trp336^6.48^, and Phe340^6.52^. As the ligand advanced toward dissociation, interactions with residues in ECL1 and ECL2 – particularly Tyr139^ECL1^, Leu228^ECL2^, and Leu229^ECL2^ – became progressively more prominent. Interactions with Tyr139^ECL1^ were absent only for compounds **8** and **3**, whose egress pathways displayed a modest deviation from those of the remaining ligands (Figure 3b,c).

While RAMD simulation times along the dominant egress route frequently correlate with experimental RTs by approximating the kinetically most favorable dissociation pathway, our findings diverge from this paradigm. This relationship assumes that the main egress path generally corresponds to the lowest energy pathway, representing the simplest route of ligand dissociation. The RAMD-derived parameters are listed in Table 2. In the present simulations, the main cluster typically comprised 10 and 14 trajectories. In contrast to earlier observations reported by Kokh et al.,^18,19^ Nunes-Alves et al.,^20^ and Berger et al.,^21^ no statistically significant correlation emerged between the experimental RTs and any of the computed metrics, including the main-path simulation time and overall mean simulation time. While the investigated compounds follow broadly similar dissociation pathways in the RAMD simulations, the lack of correlation between experimental RTs and RAMD egress times indicates that the unbinding process is not predominantly governed by steric factors (e.g., ECL2 gating). Instead, dissociation kinetics in this system appear to be influenced by localized energetic barriers and specific ligand–receptor interactions, which are not fully captured by the sterically driven RAMD methodology.

**Table 2:** Summary of RAMD Simulation Results for Individual Compounds, Including Main Cluster Population, Main-Path Simulation Time, and Average Simulation Time over All Trajectories

compound	centroid cluster size (per 30 RAMD simulations)	main path simulation time [ns]	all simulation mean time [ns]
**1**	13	0.46	0.54
**2**	10	0.26	0.34
**3**	11	0.68	0.67
**4**	14	0.39	0.52
**5**	13	0.28	0.69
**6**	12	0.80	0.69
**7**	11	0.23	0.59
**8**	11	0.52	0.82
**9**	13	0.63	1.25
**11**	15	0.65	0.89
**12**	17	0.77	0.91

### iMetaD Simulations

Building upon the RAMD egress profiles, we performed ten independent iMetaD simulations for each compound (excluding the low-affinity compound **10**). Rather than reconstructing the complete free-energy landscape, we deployed iMetaD specifically to accelerate the rare dissociation events along the predefined primary pathway. This targeted approach utilized a tailored set of CVs to capture the essential mechanistic features of ligand unbinding. Absolute RTs were extrapolated by fitting the cumulative distribution of predicted unbinding times to an exponential model, as described by Salvalaglio et al.^17^ The validity of the exponential assumption was assessed using the Kolmogorov–Smirnov test, while statistical uncertainties were quantified by bootstrapping analysis. The RT values obtained from the iMetaD simulations are summarized in Table 3. High sampling efficiency was achieved across the data set, with 7 to 10 simulation replicas per compound reaching full dissociation (Table 3).

**Table 3:** Results of MetaD Simulations for Individual Compounds in the Form of Predicted RT Values^a^

replica/compound	**1**	**2**	**3**	**4**	**5**	**6**
**0**	1.27 × 10^5^	1.61 × 10^5^	4.90 × 10^1^	1.56 × 10°^0^	**1.71 × 10** ^**3**^	**2.29 × 10** ^**4**^
**1**	8.92 × 10^6^	1.69 × 10^3^	**5.20 × 10** ^**3**^	4.20 × 10^–1^	4.17 × 10°^0^	omit
**2**	2.55 × 10^1^	5.19 × 10^4^	4.30 × 10^–3^	6.15 × 10^4^	omit	omit
**3**	omit	omit	7.96 × 10^1^	8.60 × 10^3^	6.01 × 10^1^	2.01 × 10°^0^
**4**	4.63 × 10^6^	4.95 × 10^4^	omit	**9.08 × 10** ^**2**^	4.44 × 10^1^	7.02 × 10^3^
**5**	3.23 × 10°^0^	**2.48 × 10** ^**3**^	5.98 × 10^3^	4.20 × 10^–1^	1.00 × 10^4^	7.91 × 10^7^
**6**	omit	7.66 × 10^2^	**2.72 × 10** ^**2**^	1.81 × 10^5^	**1.20 × 10** ^**3**^	omit
**7**	2.47 × 10^1^	1.58 × 10^3^	3.74 × 10^5^	**1.52 × 10** ^**3**^	1.67 × 10^5^	8.12 × 10^7^
**8**	**1.29 × 10** ^**4**^	7.14 × 10^0^	2.56 × 10^1^	2.41 × 10^2^	omit	2.35 × 10°^0^
**9**	**5.28 × 10** ^**4**^	**6.47 × 10** ^**3**^	2.15 × 10^7^	8.28 × 10^6^	4.32 × 10^5^	**1.56 × 10** ^**4**^
**fitted RT [s]**	**3.99 × 10** ^**4**^	**6.07 × 10** ^**3**^	**1100.00**	**1930.00**	**2240.00**	**20700.00**
*p*-value	**0.2132**	**0.2164**	**0.0453**	**0.0721**	**0.1874**	**0.5255**

replica/compound	**7**	**8**	**9**	**11**	**12**
**0**	1.17 × 10^2^	1.31 × 10^0^	**4.04 × 10** ^**3**^	6.04 × 10^0^	**5.38 × 10** ^**0**^
**1**	**2.99 × 10** ^**3**^	2.23 × 10^3^	omit	**3.05 × 10** ^**4**^	1.60 × 10^–1^
**2**	1.90 × 10°^0^	6.40 × 10^3^	omit	8.68 × 10^1^	7.83 × 10^3^
**3**	3.55 × 10^2^	1.99 × 10^3^	**7.72 × 10** ^**3**^	8.57 × 10^7^	omit
**4**	**2.58 × 10** ^**3**^	**4.20 × 10** ^**2**^	1.40 × 10^4^	3.04 × 10^5^	omit
**5**	2.92 × 10^2^	omit	2.37 × 10^3^	5.91 × 10^5^	3.40 × 10^–1^
**6**	6.93 × 10^8^	7.00 × 10^–1^	1.45 × 10^3^	omit	1.12 × 10^4^
**7**	1.91 × 10^8^	7.40 × 10^–1^	4.60 × 10^5^	**4.36 × 10** ^**4**^	1.12 × 10^4^
**8**	2.40 × 10^–1^	**1.01 × 10** ^**3**^	2.20 × 10^8^	4.99 × 10^2^	omit
**9**	4.39 × 10^3^	2.05 × 10^1^	5.30 × 10^1^	5.62 × 10^3^	omit
**fitted RT [s]**	**1510.00**	**407.00**	**8980.00**	**27800.00**	**9.25**
*p*-value	**0.2132**	**0.0886**	**0.6134**	**0.2156**	**0.4234**

a RTs are reported for individual replicas. Simulations exceeding 200 ns without an observed unbinding event were excluded from the analysis and are labeled as “omit”.

In addition, the fitted RT and the Kolmogorov–Smirnov test coefficient are provided. Green color corresponds to the replica that is closest to the estimated mean RT; red corresponds to the second-closest trajectory. All values are given in seconds.

To comprehensively map the dynamic interaction profiles, we extracted frame-by-frame contact frequencies for each ligand–residue pair across all simulation replicas, rigorously categorizing them by specific noncovalent interaction types (Supporting Information, Figures S26–S36). Given the inherent variability in individual trajectory lengths, we normalized the temporal axis, expressing simulation progress as a percentage (0–100% in 1% increments). This temporal standardization enabled the superposition of data from all independent replicas, yielding a consensus interaction fingerprint for each compound (Supporting Information, Figures S37–S40). Building upon these compound-specific profiles, we constructed a global interaction density map (Supporting Information, Figures S41 and 4a), selectively filtering for persistent contacts maintained throughout >30% of the normalized simulation time.

Crucially, a comparative analysis of these interaction heatmaps reveals that in the initial stages of the simulations, the compounds remain within the OBP for a substantial fraction of the trajectory, maintaining stable interactions with Asp155^3.32^, Val156^3.33^, Leu228^ECL2^, Leu229^ECL2^, Trp336^6.48^, Phe340^6.52^, Asn343^6.55^, and Val366^7.38^ (Figure 4). To further elucidate the detailed mechanism of ligand egress from this highly stabilized state, we quantified the normalized simulation time (%) at which the initial disruption of specific contacts with Asp155^3.32^, Trp336^6.48^, and Phe340^6.52^ occurred, explicitly excluding water-mediated bridges. This initial disruption was defined as the point beyond which a given interaction type remained absent for a contiguous 10% of the simulation. The dissociation process is universally initiated by the rupture of π–π interactions with Trp336^6.48^ and Phe340^6.52^; hydrophobic contacts with these residues exhibit comparable temporal stability. Concomitantly, or marginally later, the ionic and hydrogen-bonding interactions with Asp155^3.32^ are abolished. Following the disruption of these primary orthosteric anchors, we analyzed the intermediate interaction profiles, excluding the final 10% of the simulations. During this transitional egress phase, the most frequently engaged residues included Leu228^ECL2^, Leu362^7.34^, Val235^5.39^, Phe339^6.51^, Lys263^5.67^, and Ala346^6.58^. Notably, interactions with Val366^7.38^ ranked highly and emerged predominantly after the dissociation from Trp336^6.48^, suggesting that these contacts co-occur while bonds with Asp155^3.32^ and Phe340^6.52^ are still maintained. Similarly, contacts with Tyr139^ECL1^ manifest following the loss of interactions with Trp336^6.48^ and Phe339^6.51^, indicating their coexistence with the Asp155^3.32^ anchor. Finally, mapping the interactions that define the last 5% of the simulations revealed the terminal contacts governing complete dissociation. The most durable interactions in this final phase comprised ionic bonds with Glu73^1.31^, π–π interactions with Phe240^5.45^, and a combination of π-cation and π–π contacts with Tyr139^ECL1^. Additionally, persistent terminal hydrogen bonds were observed with Ala230^ECL2^, Asn354^7.26^, and Phe240^5.45^. Consequently, while classical MD simulations did not implicate dynamic rearrangements of ECL2 as the primary determinant of RT for these ritanserin derivatives, our unbinding profiles clearly reveal the transient engagement of ECL2 residues during ligand egress. Although elucidating the precise functional contribution of this loop warrants future biochemical validation, such as in vitro studies on mutant receptors, our data strongly suggest that these extracellular elements provide critical supplementary stabilization to the bound state.

**Figure 4:**
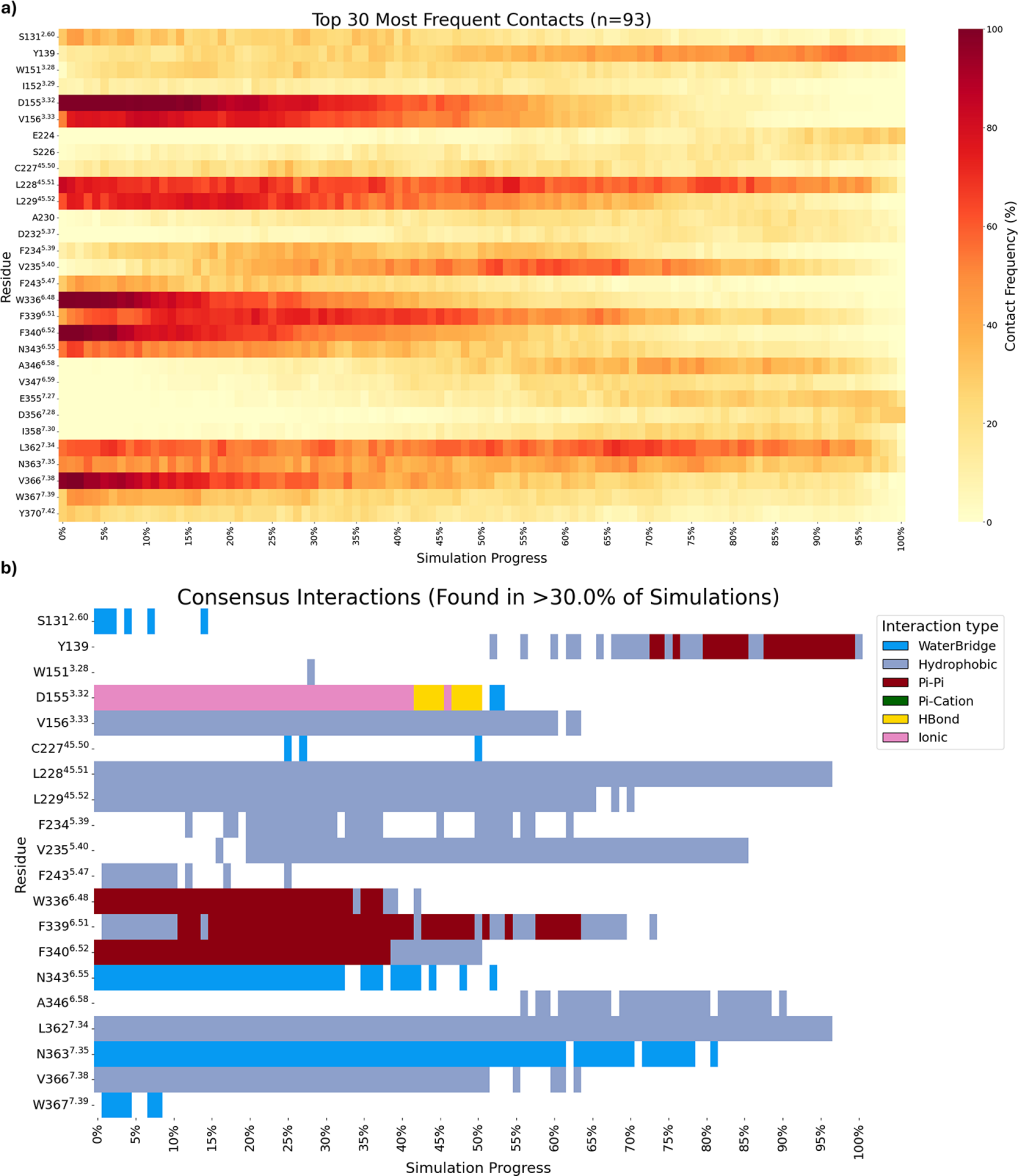
(a) Statistical analysis of the ligand–receptor contact network. Distribution of the 30 most frequent interactions as a function of normalized simulation time. (b) Consensus map of interactions observed in more than 30% of the independent trajectories.

### Mutant Receptor Models

Ligand RT at GPCRs is profoundly shaped by the structural architecture of the OBP and the topography of dissociation pathways. Within the 5-HT_2A_R, residues Trp336^6.48^ and Phe340^6.52^ situated in the extracellular region of TM6, constitute a hydrophobic cleft essential for ligand stabilization.^7,22^ Building upon our preceding findings, which identified this aromatic cluster as a significant determinant of complex stability, we further investigated the mechanistic contribution of these residues to the unbinding process. To this end, we performed a series of RAMD and iMetaD simulations using F340A (for compounds **1**–**5**) and W336A (for compounds **1**–**3**) in silico mutant receptor models. Consistent with our previously established workflow, frame-by-frame contact reports were generated for each simulation replica, rigorously categorizing all noncovalent interactions (Supporting Information, Figures S44–S51). To ensure comparability across trajectories of varying lengths, we applied temporal normalization (0–100% of the simulation progress, Supporting Information, Figures S52–S55), from which Global Interaction Density Maps were derived for each mutant complex (Supporting Information, Figures S54–S55). Subsequently, we performed a differential analysis between the wild-type (WT) and mutant interaction profiles. The resulting difference heatmaps (Figure 5) visualize the localized gain or loss of specific ligand–receptor contacts throughout the normalized simulation time, providing an atomic-level resolution of how these alanine substitutions perturb the stability of the binding mode and the dynamics of the egress pathway. For the Phe340^6.52^Ala mutant, the most pronounced attenuation of interactions was observed for the primary binding anchors Trp336^6.48^ and Asp155^3.32^, alongside Phe243^5.47^ and Phe234^5.39^. Conversely, a marginal increase in contact frequency was noted for Phe339^6.51^. In the Trp336^6.48^Ala mutant, decreased occupancy was recorded for Phe234^5.39^, Val235^5.40^, Val367^7.39^, and Ala346^6.58^. This was accompanied by a reciprocal increase in interactions with Phe339^6.51^, Tyr370^7.42^, Tyr366^7.38^, Phe332^6.44^, and Phe340^6.52^, as well as with the ECL2 residues Ser226^ECL2^, Cys227^45.50^, and Leu229^45.52^.

**Figure 5:**
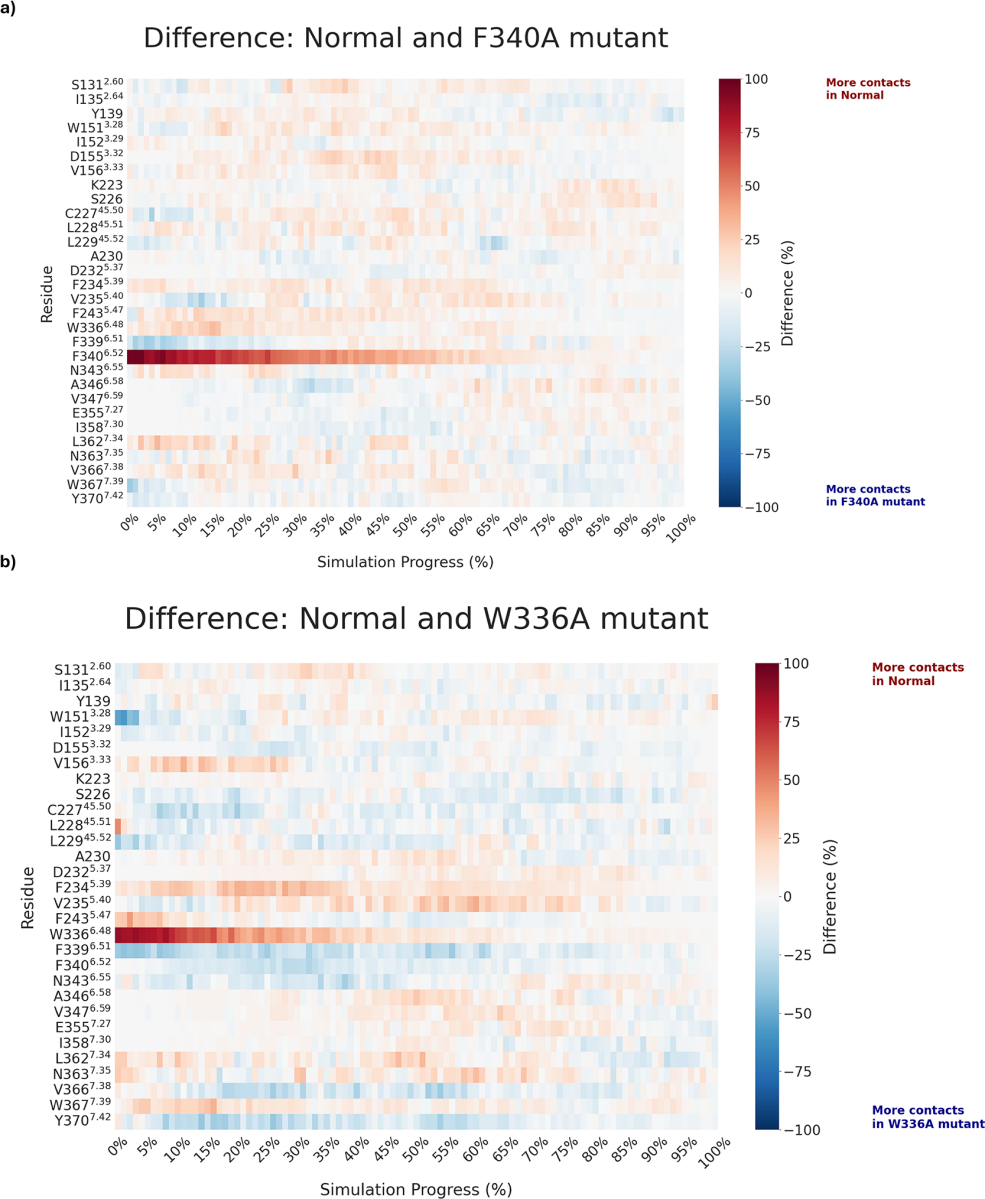
Differential interaction heatmaps highlighting the impact of alanine substitutions. Difference density maps illustrating the localized gain or loss of contact occupancy in mutant complexes relative to the wild-type (WT) 5-HT_2A_R. Panels show the WT-referenced shift in interaction frequency for (a) the Phe340^6.52^Ala mutant and (b) the Trp336^6.48^Ala mutant throughout the normalized simulation progress.

### Implications for Prospective Ligand Design

Quantitative analysis of our MD trajectories reveals that the kinetic diversity of these structurally similar compounds is fundamentally rooted in the geometric optimization of their binding within the receptor’s hydrophobic cleft. While docking studies failed to identify specific direct interactions for substituents at position 4- of the aromatic ring, our MD simulations demonstrate that this position is an important determinant of RT. Specifically, the presence of a fluorine or methyl group at position 4- promotes optimal steric complementarity, ensuring the optimal spatial orientation of the ligand’s aromatic moiety. This MD-derived mechanistic insight suggests that for ritanserin-like scaffolds, the nature of the substituent at position 4- is not merely an auxiliary feature, but a geometric anchor essential for prolonged binding.

The MD simulations further elucidate that the prolonged RT observed for these derivatives is a direct consequence of the enhanced stability and persistence of the π–π stacking network. Compounds with extended RT values exhibited highly rigid and geometrically optimized interactions with Trp336^6.48^ and Phe340^6.52^. Geometric profiling shows that the position 4 substituents promote a deeper, more persistent engagement with this hydrophobic cleft, effectively locking the ligand in a kinetically stable state. In stark contrast, our simulations reveal that ligands with short RT values fail to maintain this geometric precision; instead, they induce significant conformational heterogeneity in Phe340^6.52^. This conformational drift destabilizes the aromatic stacking network, thereby lowering the energetic barrier for ligand egress and facilitating rapid dissociation.

Future ligands may therefore be optimized to maximize shape complementarity and enable π–π stacking with the aromatic cluster, for example, by introducing substituents that favor coplanar alignment of the aromatic rings of the ligand and protein.

Further ligand optimization may also take into account insights from MD simulations regarding the role of extracellular loop regions (ECL1 and ECL2) in modulating ligand kinetics. In the case of LSD, interactions with these regions, particularly the formation of a steric “lid” over the binding pocket, have been shown to be critical for its prolonged RT by hindering ligand egress. In contrast to standard MD simulations, where no significant stabilizing contacts with ECL1 or ECL2 were detected, enhanced sampling via iMetaD reveals that these loops actively participate in modulating the unbinding process, particularly during the later stages of ligand egress. Strategically incorporating polar substituents could potentially engage residues within ECL1, such as Tyr139^ECL1^, thereby imposing additional kinetic barriers during the late stages of ligand egress. Such interactions might further prolong RT by slowing the final dissociation steps; however, this proposed mechanism warrants further systematic investigation.

## Conclusions

In this study, we present a comprehensive, mechanistically driven investigation into the molecular determinants governing ligand RT at the 5-HT_2A_R. By integrating in vitro kinetic profiling of a novel congeneric series of ritanserin derivatives with an advanced computational framework: combining classical molecular dynamics, RAMD, and iiMetaD, we demonstrate that prolonged target occupancy is not merely a function of equilibrium affinity. Rather, it is profoundly dictated by the precise geometric optimization of the ligand within the receptor’s orthosteric pocket and the spatiotemporal orchestration of its dissociation pathway.

Crucially, our structural and dynamic analyses reveal that substitution at position 4- of the ligand’s aromatic moiety serves as a kinetic anchor. This modification enforces optimal steric complementarity and promotes highly rigid, persistent π–π stacking interactions with the conserved aromatic microswitch Trp336^6.48^ and Phe340^6.52^. As corroborated by our in silico mutational analyses, these aromatic side chains act as essential, dynamic gatekeepers of the bound state. Ligands lacking this geometric precision induce conformational drift within the hydrophobic cleft, significantly lowering the energetic barrier to egress and facilitating rapid dissociation.

Beyond the orthosteric core, the complete reconstruction of unbinding trajectories via iMetaD highlighted the critical, dynamic role of the extracellular vestibule. While equilibrium simulations showed limited engagement with the extracellular loops (ECL1 and ECL2), our pathway-aware approach revealed that these regions form transient kinetic barriers that actively modulate the late stages of ligand exit. This demonstrates that extended RT does not emerge from a single structural motif but rather from a highly coordinated sequence of bond-breaking and bond-forming events along the entire dissociation route.

Overall, this study provides the first comprehensive computational and experimental kinetic analysis of a congeneric ligand series at the 5-HT_2A_R, offering practical guidance for the rational design of 5-HT_2A_R ligands with optimized RT.

## Experimental Section

### Chemistry

#### Chemicals

All organic reagents were purchased from Merck or Combi-Blocks and were used without purification. Solvents and inorganic reagents were acquired from Merck. Reaction progress was monitored by TLC on Merck Silica Gel 60 F 254 on aluminum plates. Column chromatography was performed on Merck Silica Gel 60 (0.063–0.200 mm; 70–230 mesh ASTM).

### Analytical Methods

UPLC/MS analysis was performed on Waters TQD spectrometer combined with UPLC Acquity H-Class with PDA eLambda detector. Waters Acquity UPLC BEH C18 1.7 μm 2.1 × 100 mm chromatographic column was used, at 40 °C, 0.300 mL/min flow rate, and 1.0 μL injection volume (the samples were dissolved in LC–MS grade acetonitrile, typically at a concentration of 0.1–1 mg/mL prior to injection). All mass spectra were recorded under electrospray ionization in positive mode (ESI+), and chromatograms were recorded with UV detection in the range of 190–300 nm. The gradient conditions used were: 80% phase A (water + 0.1% formic acid) and 20% phase B (acetonitrile + 0.1% formic acid) to 100% phase B (acetonitrile + 0.1% formic acid) at 3.0 min, kept until 3.5 min, then to initial conditions until 4.0 min and kept for additional 2.0 min. Total time of analysis:6.0 min.

^1^H and ^13^C NMR spectra were recorded on a Bruker Avance III HD 400 NMR spectrometer. All samples were dissolved in DMSO-*d*_6_ with TMS as an internal standard. The spectral data for the compounds refer to their free bases.

All presented compounds were of at least 95% purity as determined by LC–MS. Syntheses and characterization details for intermediate products and final compounds, as well as the spectral data for all compounds, are included in the Supporting Information.

### Procedure A

In a well-dried flask under an argon atmosphere, anhydrous tetrahydrofuran (THF) (freshly distilled over LiAlH_4_) and magnesium turnings (3.0 equiv) were combined. Bromophenol (3.0 equiv) was added dropwise, and the resulting mixture was stirred until the solution became clear, indicating the completion of the reaction initiation. Subsequently, 1-(*tert*-butyl)-4-ethylpiperidine-1,4-dicarboxylate (1.0 equiv) was added, and the reaction mixture was stirred at room temperature for 24 h. After completion, the reaction was quenched by the addition of a saturated aqueous NH_4_Cl solution. The organic solvent was removed under reduced pressure, and the resulting aqueous layer was extracted with DCM (3 × 25 mL). The combined organic layers were washed with brine, dried over anhydrous MgSO_4_, filtered, and concentrated under reduced pressure. The crude product was taken directly into the subsequent reaction without further purification or purified by flash chromatography on silica gel using hexane and a hexane/ethyl acetate (1:1) gradient as the eluent.

### Procedure B

The compound **14a**–**l** obtained from step 1 was dissolved in anhydrous dichloromethane (DCM, 40 mL). To the stirred solution, trifluoroacetic acid (TFA, 10 mL) was added, and the reaction mixture was stirred until complete conversion of the substrate was observed. Subsequently, the reaction was neutralized by the addition of an aqueous NaOH solution. The aqueous layer was extracted with DCM (3 × 20 mL). The combined organic layers were washed with brine, dried over anhydrous MgSO_4_, filtered, and concentrated under reduced pressure. The crude product was taken directly into the subsequent reaction without further purification or purified by flash chromatography on silica gel using chloroform and chloroform/methanol (9:1) mixture containing 0.5% NH_3_ as the eluent.

### Procedure C

The compound obtained from Step 2 was dissolved in n-butanol or DMF. 6-(2-Chloroethyl)-7-methyl-5*H*-thiazolo­[3,2-*a*]­pyrimidin-5-one was then added, followed by a base (either K_2_CO_3_ or triethylamine) and a catalytic amount of KI. The reaction mixture was stirred for a minimum of 4 h at 120 °C. Upon completion, the solvent was removed under reduced pressure, water was added, and the mixture was extracted with DCM (3 × 30 mL). The combined organic layers were dried over anhydrous MgSO_4_, filtered, and concentrated under reduced pressure. The crude product was purified by flash column chromatography on silica gel using chloroform or a chloroform/methanol mixture (19:1) as the eluent, unless stated otherwise. In selected cases, a small sample of the compound was additionally purified by preparative HPLC.

### *tert*-Butyl 4-(Bis­(4-fluorophenyl)­(hydroxy)­methyl)­piperidine-1-carboxylate (**13a**)

The compound was synthesized according to procedure A from 1-bromo-4-fluorobenzene (5.44 g, 31.09 mmol, 2 equiv) and 1-*tert*-butyl 4-ethylpiperidine-1,4-dicarboxylate (4 g, 15.54 mmol, 1 equiv). White solid. Yield: 6.24 g, 99%. LCMS: MS [M + H^+^]: 404.48; tR = 3.76 min.

### *tert*-Butyl 4-(Hydroxydiphenylmethyl)­piperidine-1-carboxylate (**13b**)

The compound was synthesized according to procedure A from bromobenzene (1.6 g, 10.18 mmol, 2 equiv) and 1-*tert*-butyl 4-ethylpiperidine-1,4-dicarboxylate (1.32 g, 5.13 mmol, 1 equiv). White solid. Yield: 0.587 g, 31%. LCMS: MS [M + H^+^]: 368.054; tR = 3.71 min. ^1^H NMR (400 MHz, CDCl_3_-*d*_6_) δ: 7.55–7.45 (m, 4H), 7.36–7.30 (m, 4H), 7.22 (tt, 2H), 4.30–4.04 (m, 2H), 2.73 (t, J = 12.2 Hz, 2H), 2.58 (tt, J = 11.9, 3.2 Hz, 1H), 2.17 (s, 1H), 1.50 (t, *J* = 5.6 Hz, 2H), 1.42–1.26 (m, 2H). ^13^C NMR (101 MHz, CDCl_3_) δ: 154.74, 145.64, 128.28, 126.70, 125.80, 79.65, 79.37, 44.38, 28.46, 26.47.

### *tert*-Butyl 4-(Bis­(3-fluorophenyl)­(hydroxy)­methyl)­piperidine-1-carboxylate (**13c**)

The compound was synthesized according to procedure A from 1-bromo-3-fluorobenzene (2.04 g, 11.16 mmol, 3 equiv) and 1-*tert*-butyl 4-ethylpiperidine-1,4-dicarboxylate (1 g, 3.89 mmol, 1 equiv). White solid. Yield: 0.855 g, 54%. LCMS: MS [M + H^+^]: 404.041; tR = 3.79 min. ^1^H NMR (400 MHz, CDCl_3_) δ: 7.33–7.26 (m, 2H), 7.25–7.19 (m, 4H), 6.92 (tdd, *J* = 8.1, 2.6, 1.3 Hz, 2H), 4.24–4.09 (m, 2H), 2.78–2.67 (m, 2H), 2.51 (tt, *J* = 11.8, 3.3 Hz, 1H), 2.31 (s, 1H), 1.45 (s, 9H), 1.40–1.26 (m, 2H). ^13^C NMR (101 MHz, CDCl_3_) δ: 164.18, 161.73, 154.67, 147.90, 129.94, 129.86, 121.19, 113.94, 113.73, 113.14, 112.91, 79.53, 79.18, 79.16, 79.15, 44.41, 28.42, 26.27.

### *tert*-Butyl 4-(Bis­(3,5-difluorophenyl)­(hydroxy)­methyl)­piperidine-1-carboxylate (**13d**)

The compound was synthesized according to procedure A from 1-bromo-3,5-difluorobenzene (4.5 g, 23.32 mmol, 3 equiv) and 1-*tert*-butyl 4-ethylpiperidine-1,4-dicarboxylate (2 g, 7.77 mmol, 1 equiv). White solid. Yield: 2.07 g, 61%. LCMS: MS [M + H^+^] 439.981; tR = 3.920 min.

### *tert*-Butyl 4-(Bis­(3,4-difluorophenyl)­(hydroxy)­methyl)­piperidine-1-carboxylate (**13e**)

The compound was synthesized according to procedure A from 4-bromo-1,2-difluorobenzene (2.25 g, 11.16 mmol, 3 equiv) and 1-*tert*-butyl 4-ethylpiperidine-1,4-dicarboxylate (1 g, 3.89 mmol, 1 equiv). White solid. Yield: 0.742 g, 43%. LCMS: MS [M + H^+^] 440.011; tR = 3.874 min.

### *tert*-Butyl 4-(Bis­(3-fluoro-5-methylphenyl)­(hydroxy)­methyl)­piperidine-1-carboxylate (**13f**)

The compound was synthesized according to procedure A from 1-bromo-3-fluoro-5-methylbenzene (2.20 g, 11.16 mmol, 3 equiv) and 1-*tert*-butyl 4-ethylpiperidine-1,4-dicarboxylate (1 g, 3.89 mmol, 1 equiv). White solid. Yield: 0.994 g, 56%. LCMS: MS [M + H^+^] 432.060; tR = 4.018 min.

### *tert*-Butyl 4-(Hydroxybis­(3,4,5-trifluorophenyl)­methyl)­piperidine-1-carboxylate (**13g**)

The compound was synthesized according to procedure A from 5-bromo-1,2,3-trifluorobenzene (2.46 g, 11.16 mmol, 3 equiv) and 1-*tert*-butyl 4-ethylpiperidine-1,4-dicarboxylate (1 g, 3.89 mmol, 1 equiv). Colorless oil. Yield: 0.755 g, 41%. LCMS: MS [M + H^+^] 476.19; tR = 4.069 min.

### *tert*-Butyl 4-(Hydroxybis­(3-(trifluoromethyl)­phenyl)­methyl)­piperidine-1-carboxylate (**13h**)

The compound was synthesized according to procedure A from 1-bromo-3-(trifluoromethyl)­benzene (5.25 g, 23.32 mmol, 3 equiv) and 1-*tert*-butyl 4-ethylpiperidine-1,4-dicarboxylate (2 g, 7.77 mmol, 1 equiv). White solid. Yield: 3.546 g, 91%. LCMS: MS [M + H^+^] 504.32; tR = 4.11 min.

### *tert*-Butyl 4-(Hydroxybis­(4-methoxyphenyl)­methyl)­piperidine-1-carboxylate (**13i**)

The compound was synthesized according to procedure A from 1-bromo-4-methoxybenzene (4.36 g, 23.32 mmol, 3 equiv) and 1-*tert*-butyl 4-ethylpiperidine-1,4-dicarboxylate (2 g, 7.77 mmol, 1 equiv). White solid. Yield: 3.23 g, 97%. LCMS: MS [M + H^+^] 428.47; tR = 3.61 min.

### *tert*-Butyl 4-(Bis­(4-(dimethylamino)­phenyl)­(hydroxy)­methyl)­piperidine-1-carboxylate (**13j**)

The compound was synthesized according to procedure A from 4-bromo-*N*,*N*-dimethylaniline (4.67 g, 23.32 mmol, 3 equiv) and 1-*tert*-butyl 4-ethylpiperidine-1,4-dicarboxylate (2 g, 7.77 mmol, 1 equiv). White solid. Yield: 2.28 g, 79%. LCMS: MS [M + H^+^] 454.036; tR = 2.44 min.

### *tert*-Butyl 4-(Hydroxydi-*p*-tolylmethyl)­piperidine-1-carboxylate (**13k**)

The compound was synthesized according to procedure A from 1-bromo-4-methylbenzene (5.98 g, 34.97 mmol, 3 equiv) and 1-*tert*-butyl 4-ethylpiperidine-1,4-dicarboxylate (3 g, 11.66 mmol, 1 equiv). White solid. Yield: 4.54 g, 98%. LCMS: MS [M + H^+^] 396.15; tR = 4.06 min.

### *tert*-Butyl 4-(Hydroxybis­(4-(trifluoromethyl)­phenyl)­methyl)­piperidine-1-carboxylate (**13l**)

The compound was synthesized according to procedure A from 1-bromo-4-(trifluoromethyl)­benzene (7.87 g, 34.97 mmol, 3 equiv) and 1-*tert*-butyl 4-ethylpiperidine-1,4-dicarboxylate (3 g, 11.66 mmol, 1 equiv). White solid. Yield: 5.36 g, 91%. LCMS: MS [M + H^+^] 504.25; tR = 4.13 min.

### 4-(Bis­(4-fluorophenyl)­methylene)­piperidine (**14a**)

The compound was synthesized according to procedure B from *tert*-butyl 4-(bis­(4-fluorophenyl)­(hydroxy)­methyl)­piperidine-1-carboxylate (5.24 g, 12.98 mmol). Yellow solid. Yield: 4.27 g, 97%. LCMS: MS [M + H^+^] 285.876; tR = 2.513 min.

### 4-(Diphenylmethylene)­piperidine (**14b**)

The compound was synthesized according to procedure B from *tert*-butyl 4-(hydroxydiphenylmethyl)­piperidine-1-carboxylate (744 mg, 2.02 mmol). Yellow solid. Yield: 779 mg, 99%. LCMS: MS [M + H^+^] 250.88; tR = 2.23 min.

### 4-(Bis­(3-fluorophenyl)­methylene)­piperidine (**14c**)

The compound was synthesized according to procedure B from *tert*-butyl 4-(bis­(3-fluorophenyl)­(hydroxy)­methyl)­piperidine-1-carboxylate (845 mg, 2.07 mmol). Yellow solid. Yield: 458 mg, 77%. LCMS: MS [M + H^+^] 286.86; tR = 2.46 min.

### 4-(Bis­(3,5-difluorophenyl)­methylene)­piperidine (**14d**)

The compound was synthesized according to procedure B from *tert*-butyl 4-(bis­(3,5-difluorophenyl)­(hydroxy)­methyl)­piperidine-1-carboxylate (2.1 g, 4.77 mmol). H_2_SO_4_ instead of TFA. Yellow solid. Yield: 660 mg, 43%. LCMS: MS [M + H^+^] 321.492; tR = 2.560 min.

### 4-(Bis­(3,4-difluorophenyl)­methylene)­piperidine (**14e**)

The compound was synthesized according to procedure B from *tert*-butyl 4-(bis­(3,4-difluorophenyl)­(hydroxy)­methyl)­piperidine-1-carboxylate (722 mg, 1.64 mmol). Yellow solid. Yield: 296 mg, 56%. LCMS: MS [M + H+]: 321.630; tR = 2.519 min.

### 4-(Bis­(3-fluoro-5-methylphenyl)­methylene)­piperidine (**14f**)

The compound was synthesized according to procedure B from *tert*-butyl 4-(bis­(3-fluoro-5-methylphenyl)­(hydroxy)­methyl)­piperidine-1-carboxylate (994 mg, 1.59 mmol). Yellow solid. Yield: 448 mg, 62%. LCMS: MS [M + H^+^] 313.566; tR = 2.512 min.

### 4-(Bis­(3,4,5-trifluorophenyl)­methylene)­piperidine (**14g**)

The compound was synthesized according to procedure B from *tert*-butyl 4-(hydroxybis­(3,4,5-trifluorophenyl)­methyl)­piperidine-1-carboxylate (755 mg, 1.59 mmol). Yellow solid. Yield: 230 mg, 41%. LCMS: MS [M + H^+^] 357.682; tR = 2.559 min.

### 4-(Bis­(3-(trifluoromethyl)­phenyl)­methylene)­piperidine (**14h**)

The compound was synthesized according to procedure B from *tert*-butyl 4-(hydroxybis­(3-(trifluoromethyl)­phenyl)­methyl)­piperidine-1-carboxylate (3.54 g, 7.03 mmol). Yellow solid. Yield: 2.67 g, 99%. LCMS: MS [M + H^+^] 385.667; tR = 2.650 min.

### 4-(Bis­(4-methoxyphenyl)­methylene)­piperidine (**14i**)

The compound was synthesized according to procedure B from *tert*-butyl 4-(hydroxybis­(4-methoxyphenyl)­methyl)­piperidine-1-carboxylate (3.23 g, 7.56 mmol). Yellow solid. Yield: 860 mg, 37%. LCMS: MS [M + H^+^] 310.92; tR = 2.19 min.

### 4,4′-(Piperidin-4-ylidenemethylene)­bis­(*N*,*N*-dimethylaniline) (**14j**)

The compound was synthesized according to procedure B from *tert*-butyl 4-(bis­(4-(dimethylamino)­phenyl)­(hydroxy)­methyl)­piperidine-1-carboxylate (216 mg, 0.6 mmol). Yellow solid. Yield: 120 mg, 35%. LCMS: MS [M + H^+^] 335.733; tR = 1.782 min.

### 4-(Di-*p*-tolylmethylene)­piperidine­(**14k**)

The compound was synthesized according to procedure B from *tert*-butyl 4-(hydroxydi-*p*-tolylmethyl)­piperidine-1-carboxylate (2 g, 7.21 mmol). Yellow solid. Yield: 2.3 mg, 68%. LCMS: MS [M + H^+^] 278.27; tR = 2.46 min.

### 4-(Bis­(4-(trifluoromethyl)­phenyl)­methylene)­piperidine (**14l**)

The compound was synthesized according to procedure B from *tert*-butyl 4-(hydroxybis­(4-(trifluoromethyl)­phenyl)­methyl)­piperidine-1-carboxylate (3.55 g, 8.95 mmol). Yellow solid. Yield: 4.5 g, 87%. LCMS: MS [M + H^+^] 386.22; tR = 2.71 min.

### 6-(2-(4-(Bis­(4-fluorophenyl)­methylene)­piperidin-1-yl)­ethyl)-7-methyl-5*H*-thiazolo­[3,2-*a*]­pyrimidin-5-one (**1**)

The compound was synthesized according to procedure C from 4-(bis­(4-fluorophenyl)­methylene)­piperidine (300 mg, 1.05 mmol, 1 equiv) and 6-(2-chloroethyl)-7-methyl-5*H*-thiazolo­[3,2-*a*]­pyrimidin-5-one (721 mg, 3.15 mmol, 3 equiv). *n*-BuOH as a solvent, TEA as the base. White solid. Yield: 315 mg, 63%. Purity: 100%. LCMS: MS [M + H^+^]: 477.851; tR = 2.442 min. Calculated exact mass value (ChemDraw): 477.17 ^1^H NMR (400 MHz, DMSO) δ: 7.94 (d, *J* = 4.9, 1.4 Hz, 1H), 7.48 (d, *J* = 4.9, 1.4 Hz, 1H), 7.21–7.08 (m, 8H), 2.68 (dd, *J* = 9.3, 6.1 Hz, 2H), 2.56–2.52 (m, 4H), 2.40 (dd, *J* = 9.4, 6.2 Hz, 2H), 2.35 (s, 3H), 2.25 (t, *J* = 5.5 Hz, 4H). ^13^C NMR (101 MHz, DMSO) δ: 162.49, 160.08 (d), 158.45, 138.7, 138.66, 136.9, 133.36, 131.76 (d, *J* = 8.0 Hz), 122.08, 115.47 (d, *J* = 21.2 Hz), 113.7, 113.39, 56.43, 54.95, 31.75, 23.7, 22.19.

### 6-(2-(4-(Diphenylmethylene)­piperidin-1-yl)­ethyl)-7-methyl-5*H*-thiazolo­[3,2-*a*]­pyrimidin-5-one (**2**)

The compound was synthesized according to procedure C from 4-(diphenylmethylene)­piperidine (0.759 g, 3.04 mmol, 1 equiv) and 6-(2-chloroethyl)-7-methyl-5*H*-thiazolo­[3,2-*a*]­pyrimidin-5-one (1.04 g, 4.57 mmol, 1.5 equiv). *n*-BuOH as a solvent, TEA as the base. Yellow solid. Yield: 233 mg, 17%. Purity: 100%. LCMS: MS [M + H^+^]: 441.693; tR = 2.313 min. Calculated exact mass value (ChemDraw): 441.59 ^1^H NMR (400 MHz, DMSO) δ: 7.95 (d, *J* = 4.9 Hz, 1H), 7.48 (d, *J* = 4.9 Hz, 1H), 7.36–7.27 (m, 4H), 7.26–7.16 (m, 2H), 7.14–7.07 (m, 4H), 2.75–2.66 (m, 2H), 2.59–2.54 (m, 4H), 2.49–2.40 (m, 2H), 2.35 (s, 3H), 2.31–2.24 (m, 4H). ^13^C NMR (101 MHz, DMSO) δ: 160.20, 159.72, 158.46, 142.48, 135.81, 129.82, 128.61, 126.89, 122.09, 113.45, 56.31, 54.90, 40.62, 31.50, 23.50, 22.21.

### 6-(2-(4-(Bis­(3-fluorophenyl)­methylene)­piperidin-1-yl)­ethyl)-7-methyl-5*H*-thiazolo­[3,2-*a*]­pyrimidin-5-one (**3**)

Compound was synthesized according to procedure C from 4-(bis­(3-fluorophenyl)­methylene)­piperidine (438 mg, 1.54 mmol, 1 equiv) and 6-(2-chloroethyl)-7-methyl-5*H*-thiazolo­[3,2-*a*]­pyrimidin-5-one (527 mg, 2.30 mmol, 1.5 equiv). *n*-BuOH as a solvent, TEA as the base. White solid. Yield: 326 mg, 44%. Purity: 100%. LCMS: MS [M + H^+^]: 477.837; tR = 2.425 min. Calculated exact mass value (ChemDraw): 477.17 ^1^H NMR (400 MHz, DMSO) δ: 7.94 (d, *J* = 4.9 Hz, 1H), 7.48 (d, *J* = 4.9 Hz, 1H), 7.41–7.34 (m, 2H), 7.13–7.01 (m, 2H), 7.01–6.89 (m, 4H), 2.73–2.65 (m, 2H), 2.58–2.52 (m, 4H), 2.46–2.38 (m, 2H), 2.35 (s, 3H), 2.25 (t, *J* = 5.5 Hz, 4H). ^13^C NMR (101 MHz, DMSO) δ: 163.63, 161.21, 160.12, 159.65, 158.46, 144.35 (d), 130.70 (d), 126.02, 122.08, 116.53, 116.32, 113.97 (d), 113.64, 113.41, 56.33, 54.76, 31.64, 23.67, 22.20.

### 6-(2-(4-(Bis­(3,5-difluorophenyl)­methylene)­piperidin-1-yl)­ethyl)-7-methyl-5*H*-thiazolo­[3,2-*a*]­pyrimidin-5-one (**4**)

The compound was synthesized according to procedure C from 4-(bis­(3,5-difluorophenyl)­methylene)­piperidine (200 mg, 0.62 mmol, 1 equiv) and 6-(2-chloroethyl)-7-methyl-5*H*-thiazolo­[3,2-*a*]­pyrimidin-5-one (427 mg, 1.67 mmol, 3 equiv). *n*-BuOH as a solvent, TEA as the base. White solid. Yield: 176 mg, 55%. Purity: 100%. LCMS: MS [M + H^+^]: 513.423; tR = 2.533 min. Calculated exact mass value (ChemDraw): 513.15 ^1^H NMR (400 MHz, DMSO) δ: 7.94 (d, *J* = 4.9 Hz, 1H), 7.48 (d, *J* = 4.9 Hz, 1H), 7.14 (ddd, *J* = 11.8, 8.2, 2.5 Hz, 2H), 6.96–6.87 (m, 4H), 2.68 (dd, *J* = 9.4, 6.0 Hz, 2H), 2.54 (t, *J* = 5.2 Hz, 4H), 2.41 (dd, *J* = 9.4, 6.0 Hz, 2H), 2.35 (s, 3H), 2.21 (t, *J* = 5.6 Hz, 4H). ^13^C NMR (101 MHz, DMSO) δ: 163.92 (d, *J* = 13.8 Hz), 161.47 (d, *J* = 13.8 Hz), 160.10, 159.64, 158.47, 144.99 (t, *J* = 9.3 Hz), 139.50, 131.28, 122.09, 113.54 (d, *J* = 26.5 Hz), 113.12–112.71 (m), 102.97 (t, *J* = 25.8 Hz), 56.28, 54.51, 31.59, 23.71, 22.20.

### 6-(2-(4-(Bis­(3,4-difluorophenyl)­methylene)­piperidin-1-yl)­ethyl)-7-methyl-5*H*-thiazolo­[3,2-*a*]­pyrimidin-5-one (**5**)

The compound was synthesized according to procedure C from 4-(bis­(3,4-difluorophenyl)­methylene)­piperidine (296 mg, 0.91 mmol, 1 equiv) and 6-(2-chloroethyl)-7-methyl-5*H*-thiazolo­[3,2-*a*]­pyrimidin-5-one (316 mg, 1.38 mmol, 1.5 equiv). *n*-BuOH as a solvent. TEA as the base. White solid. Yield: 8.15 mg, 2%. Purity: 100%. LCMS: MS [M + H^+^]: 513.860; tR = 2.573 min. Calculated exact mass value (ChemDraw): 513.15 ^1^H NMR (400 MHz, CDCl_3_) δ: 7.90 (d, *J* = 4.9 Hz, 1H), 6.92 (d, *J* = 4.9 Hz, 1H), 6.76–6.71 (m, 2H), 6.71–6.69 (m, 2H), 6.66–6.58 (m, 2H), 2.92–2.83 (m, 2H), 2.66 (t, *J* = 5.5 Hz, 4H), 2.62–2.54 (m, 2H), 2.45 (s, 3H), 2.42 (m, 4H). ^13^C NMR (101 MHz, CDCl_3_) δ: 163.78, 161.34, 160.62, 159.28, 158.89, 143.58 (d, *J* = 8.1 Hz), 139.98 (d, *J* = 8.2 Hz), 134.32, 125.98, 125.96, 121.82, 114.17 (d, *J* = 20.9 Hz), 113.97, 113.52 (d, *J* = 21.0 Hz), 110.93, 77.23, 56.29, 54.96, 31.36, 23.51, 22.18.

### 6-(2-(4-(Bis­(3-fluoro-5-methylphenyl)­methylene)­piperidin-1-yl)­ethyl)-7-methyl-5*H*-thiazolo­[3,2-*a*]­pyrimidin-5-one (**6**)

The compound was synthesized according to procedure C from 4-(bis­(3-fluoro-5-methylphenyl)­methylene)­piperidine (200 mg, 0.64 mmol, 1 equiv) and 6-(2-chloroethyl)-7-methyl-5*H*-thiazolo­[3,2-*a*]­pyrimidin-5-one (437 mg, 1.91 mmol, 3 equiv). *n*-BuOH as a solvent. TEA as the base. White solid. Yield: 36 mg, 11%. Purity: 100%. LCMS: MS [M + H^+^]: 505.870; tR = 2.667 min. Calculated exact mass value (ChemDraw): 505.20 ^1^H NMR (400 MHz, CDCl_3_) δ: 7.90 (d, *J* = 4.9 Hz, 1H), 6.92 (d, *J* = 4.9 Hz, 1H), 6.76–6.71 (m, 2H), 6.70 (s, 2H), 6.66–6.58 (m, 2H), 2.92–2.83 (m, 2H), 2.70–2.62 (m, 4H), 2.61–2.54 (m, 2H), 2.45 (s, 3H), 2.41 (t, *J* = 5.7 Hz, 4H), 2.31 (s, 6H). ^13^C NMR (101 MHz, DMSO) δ: 175.67, 163.59, 161.17, 158.48, 144.07, 140.81, 140.72, 126.36, 122.10, 117.84, 114.58, 114.39, 113.52, 113.31, 40.68, 40.47, 34.76, 31.62, 30.30, 29.46, 22.23, 21.32.

### 6-(2-(4-(Bis­(3,4,5-trifluorophenyl)­methylene)­piperidin-1-yl)­ethyl)-7-methyl-5*H*-thiazolo­[3,2-*a*]­pyrimidin-5-one (**7**)

The compound was synthesized according to procedure C from 4-(bis­(3,4,5-trifluorophenyl)­methylene)­piperidine (200 mg, 0.56 mmol, 1 equiv) and 6-(2-chloroethyl)-7-methyl-5*H*-thiazolo­[3,2-*a*]­pyrimidin-5-one (384 mg, 1.68 mmol, 3 equiv). *n*-BuOH as a solvent. TEA as the base. White solid. Yield: 220 mg, 72%. Purity: 100%. LCMS: MS [M + H^+^]: 549.425; tR = 2.730 min. Calculated exact mass value (ChemDraw): 549.13 ^1^H NMR (400 MHz, DMSO) δ: 7.94 (d, *J* = 4.9 Hz, 1H), 7.48 (d, *J* = 4.9 Hz, 1H), 7.21–7.09 (m, 4H), 2.68 (dd, *J* = 9.3, 6.0 Hz, 2H), 2.55–2.53 (m, 4H), 2.41 (dd, *J* = 9.3, 6.2 Hz, 2H), 2.35 (s, 3H), 2.20 (t, *J* = 5.5 Hz, 4H). ^13^C NMR (101 MHz, DMSO) δ: 159.03, 158.58, 157.40, 150.73, 148.27, 139.18, 136.85, 128.98, 121.01, 113.70–113.29 (m), 112.48 (d, *J* = 24.7 Hz), 55.18, 53.32, 30.48, 22.63, 21.13.

### 6-(2-(4-(Bis­(3-(trifluoromethyl)­phenyl)­methylene)­piperidin-1-yl)­ethyl)-7-methyl-5*H*-thiazolo­[3,2-*a*]­pyrimidin-5-one (**8**)

The compound was synthesized according to procedure C from 4-(bis­(3-(trifluoromethyl)­phenyl)­methylene)­piperidine (1.19 g, 5.19 mmol, 2 equiv) and 6-(2-chloroethyl)-7-methyl-5*H*-thiazolo­[3,2-*a*]­pyrimidin-5-one (1 g, 2.60 mmol, 1 equiv). DMF as a solvent. TEA as the base. Yellow solid. Yield: 343 mg, 23%. Purity: 98.36%. LCMS: MS [M + H^+^]: 577.415; tR = 2.667 min. Calculated exact mass value (ChemDraw): 577.16 ^1^H NMR (400 MHz, DMSO) δ: 7.88 (d, *J* = 4.9 Hz, 1H), 7.62–7.46 (m, 5H), 7.45–7.37 (m, 5H), 2.66–2.58 (m, 2H), 2.52–2.44 (m, 4H), 2.39–2.30 (m, 2H), 2.28 (s, 3H), 2.18 (t, *J* = 5.4 Hz, 4H). ^13^C NMR (101 MHz, DMSO) δ: 160.11, 159.63, 158.46, 142.76, 139.27, 134.13, 132.66, 130.04, 129.59 (d, *J* = 31.2 Hz), 126.06 (q, *J* = 4.0 Hz), 125.91, 124.10, 124.05 (q, *J* = 3.6 Hz), 123.99, 123.20, 122.07, 113.66, 113.40, 56.29, 54.66, 31.71, 23.70, 22.17.

### 6-(2-(4-(Bis­(4-methoxyphenyl)­methylene)­piperidin-1-yl)­ethyl)-7-methyl-5H-thiazolo­[3,2-*a*]­pyrimidin-5-one (**9**)

The compound was synthesized according to procedure C from 4-(bis­(4-methoxyphenyl)­methylene)­piperidine (860 mg, 3.76 mmol, 1.5 equiv) and 6-(2-chloroethyl)-7-methyl-5*H*-thiazolo­[3,2-*a*]­pyrimidin-5-one (776 mg, 2.51 mmol, 1 equiv). *n*-BuOH as a solvent, TEA as the base. White solid. Yield: 749 mg, 60%. Purity: 100%. LCMS: MS [M + H^+^]: 501.865; tR = 2.433 min. Calculated exact mass value (ChemDraw): 501.21 ^1^H NMR (400 MHz, DMSO) δ: 7.94 (t, *J* = 4.2 Hz, 1H), 7.49 (dd, *J* = 8.7, 4.9 Hz, 1H), 7.07–6.94 (m, 4H), 6.88 (dd, *J* = 11.0, 8.4 Hz, 4H), 3.74 (d, *J* = 4.1 Hz, 6H), 3.30–3.17 (m, 3H), 3.13–3.04 (m, 1H), 2.97–2.85 (m, 1H), 2.73–2.64 (m, 1H), 2.42–2.39 (m, 2H), 2.38–2.33 (m, 2H), 2.27 (t, *J* = 5.4 Hz, 2H). ^13^C NMR (101 MHz, DMSO) δ: 160.97, 160.22, 160.12, 159.62, 158.47, 158.35, 158.12, 135.15, 134.83, 134.66, 131.04, 122.09, 114.03, 113.91, 31.88, 26.54, 23.71, 22.26, 22.20.

### 6-(2-(4-(Bis­(4-(dimethylamino)­phenyl)­methylene)­piperidin-1-yl)­ethyl)-7-methyl-5H-thiazolo­[3,2-*a*]­pyrimidin-5-one (**10**)

The compound was synthesized according to procedure C from 4,4′-(piperidin-4-ylidenemethylene)­bis­(*N*,*N*-dimethylaniline) (216 mg, 0.643 mmol, 1 equiv) and 6-(2-chloroethyl)-7-methyl-5H-thiazolo­[3,2-*a*]­pyrimidin-5-one (295 mg, 1.29 mmol, 2 equiv). *n*-BuOH as a solvent. TEA as the base. Yellow solid. Yield: 120 mg, 35%. Purity: 100%. LCMS: MS [M + H+]: 527.684; tR = 1.670 min. Calculated exact mass value (ChemDraw): 527.73 ^1^H NMR (400 MHz, DMSO) δ: 8.00 (d, *J* = 4.9 Hz, 1H), 7.56 (d, *J* = 4.8 Hz, 1H), 7.16 (d, *J* = 7.9 Hz, 4H), 7.03 (d, *J* = 7.8 Hz, 4H), 3.36 (m, 2H), 3.23–3.14 (m, 2H), 2.96 (dd, *J* = 10.1, 6.0 Hz, 2H), 2.60–2.48 (m, 4H), 2.42 (s, 3H), 2.29 (s, 6H). ^13^C NMR (101 MHz, DMSO) δ: 161.23, 160.64, 158.52, 138.88, 138.14, 136.55, 129.64, 129.31, 122.15, 114.09, 110.47, 54.03, 53.02, 28.78, 22.44, 21.37, 21.20.

### 6-(2-(4-(Di-*p*-tolylmethylene)­piperidin-1-yl)­ethyl)-7-methyl-5*H*-thiazolo­[3,2-*a*]­pyrimidin-5-one (**11**)

The compound was synthesized according to procedure C from 4-(di-*p*-tolylmethylene)­piperidine (2 g, 7.21 mmol, 1 equiv) and 6-(2-chloroethyl)-7-methyl-5H-thiazolo­[3,2-*a*]­pyrimidin-5-one (3.3 g, 14.42 mmol, 2 equiv). *n*-BuOH as a solvent. TEA as the base. Yellow solid. Yield: 2.3 g, 68%. Purity: 100%. LCMS: MS [M + H+]: 469.424; tR = 2.580 min. Calculated exact mass value (ChemDraw): 469.22 ^1^H NMR (400 MHz, DMSO) δ: 8.00 (d, *J* = 4.9 Hz, 1H), 7.56 (d, *J* = 4.8 Hz, 1H), 7.16 (d, *J* = 7.9 Hz, 4H), 7.03 (d, *J* = 7.8 Hz, 4H), 3.36 (m, 2H), 3.23–3.14 (m, 2H), 2.96 (dd, *J* = 10.1, 6.0 Hz, 2H), 2.60–2.48 (m, 4H), 2.42 (s, 3H), 2.29 (s, 6H). ^13^C NMR (101 MHz, DMSO) δ: 161.23, 160.64, 158.52, 138.88, 138.14, 136.55, 129.64, 129.31, 122.15, 114.09, 110.47, 54.03, 53.02, 28.78, 22.44, 21.37, 21.20.

### 6-(2-(4-(Bis­(4-(trifluoromethyl)­phenyl)­methylene)­piperidin-1-yl)­ethyl)-7-methyl-5*H*-thiazolo­[3,2-*a*]­pyrimidin-5-one (**12**)

The compound was synthesized according to procedure C from 4-(bis­(4-(trifluoromethyl)­phenyl)­methylene)­piperidine (3.45 g, 8.95 mmol, 1 equiv) and 6-(2-chloroethyl)-7-methyl-5H-thiazolo­[3,2-*a*]­pyrimidin-5-one (3.07 g, 13.29 mmol, 2 equiv). *n*-BuOH as a solvent. TEA as the base. Yellow solid. Yield: 4.5 g, 87.02%. Purity: 100%. LCMS: MS [M + H+]: 577.554; tR = 2.810 min. Calculated exact mass value (ChemDraw): 577.16 ^1^H NMR (400 MHz, DMSO) δ: 7.99 (d, *J* = 4.9 Hz, 1H), 7.76 (d, *J* = 7.9 Hz, 4H), 7.54 (d, *J* = 4.9 Hz, 1H), 7.42 (d, *J* = 7.7 Hz, 4H), 3.79–3.54 (m, 2H), 3.24–3.08 (m, 4H), 2.96–2.91 (m, 2H), 2.41 (s, 3H). ^13^C NMR (101 MHz, DMSO) δ: 160.63, 158.52, 145.16, 130.72, 129.58, 129.03, 128.35, 128.03, 125.93, 123.31, 122.15, 114.02, 56.30, 52.71, 30.07, 28.81, 22.35.

### Docking

All compounds were docked to the inactive conformation of the human 5-HT_2A_ receptor, consistent with the postulated antagonistic activity of the investigated ligands. The X-ray crystal structure of the 5-HT_2A_R in complex with risperidone (PDB ID: 6A93) was used as the receptor model.

Ligands were prepared for docking using LigPrep (Schrödinger Suite 2024-4), with protonation states and possible tautomers generated at physiological pH (7.4 ± 0.0). The receptor structure was processed using the Protein Preparation Workflow, which included assignment of bond orders, addition of hydrogen atoms, optimization of hydrogen-bonding networks, and restrained energy minimization.

Molecular docking was performed using Glide in the standard precision mode. The docking grid was centered on the orthosteric binding site defined by the cocrystallized ligand. The resulting docking poses were visually inspected and analyzed by using PyMOL.

### MD

The resulting ligand–receptor complexes constituted starting points for MD simulations. They were carried out in Desmond, using the TIP3P solvent model, POPC (palmitoyl-oleoyl-phosphatidylcholine) as a membrane model, the OPLS4 force-field under the pressure of 1.01325 bar, and a temperature of 300 K. The box shape was orthorhombic, with a size of +10 Å × + 10 Å × + 10 Å. In each case, the system was neutralized by the addition of the appropriate number of Cl- ions; 0.15 M NaCl was added to mimic the ionic strength inside the cell and relaxed before simulation; the duration of each simulation was equal to 2000 ns, recording frames every 1 ns with a time step of 2 fs. We used NPγT assembly (thermostat method: Nose–Hoover chain with relaxation time of 1.0 ps; barostat method: Martyna-Tobias-Klein with relaxation time of 1.0 ps). Additional MD simulations were performed for selected compounds with a shorter duration of 500 ns but with increased temporal resolution, yielding 10,000 frames per simulation (Figure S58). The interactions between ligands and the respective proteins during MD simulations were analyzed using the Simulation Interaction Diagram from the Schrödinger Suite. In addition, RAMD and MetaD protocols from the Schrodinger Suite 2024_4 were used. In RAMD, the ligand’s displacement is monitored, and if it exceeds a predefined threshold (0.05 Å), the force vector is maintained; otherwise, its direction is reassigned randomly. Each RAMD sampling run begins with a constant force of 15 kcal·mol-1·Å^–2^, which increases linearly by 5 kcal·mol-1·Å^–2^ per nanosecond until the ligand unbinds. The unbinding event was considered to occur when the minimum distance between any protein atom and any ligand atom exceeded 8 Å. To prevent structural distortion of the protein during the process, a weak restraining force (*K* = 0.01 kcal·mol-1·Å^–2^) was applied to the protein Cα atoms. During the RAMD stage, 30 egress trajectories were generated and subsequently clustered, allowing identification of the predominant dissociation pathway corresponding to the lowest free energy path. The second stage involves iMetaD simulations, in which an additional biasing potential acts on selected CVs. In our case, the CVs were derived from the main RAMD trajectory and defined as S, the progress along the unbinding path, and Z, the distance from the path. From 10 independent iMetaD simulations, the RT was estimated by fitting the cumulative distribution function of the escape times to an exponential distribution, as described by Weis and Kobilka.^12^ The KS test was used to verify that the sampled distribution follows an exponential behavior, with statistical uncertainties estimated using bootstrapping.

### In Vitro Examination of Compound RT

Membrane preparations were obtained from CHO–K1 cells stably expressing the human 5-HT_2A_R (Revvity, cat. no. ES-313-C). Cells were cultured at 37 °C in a humidified atmosphere with 5% CO_2_ in DMEM supplemented with 10% dialyzed fetal bovine serum and 500 μg/mL G418 sulfate. Cells were grown to approximately 90% confluence in 150 cm^2^ flasks, washed twice with prewarmed PBS, and harvested by centrifugation (200*g*) in PBS containing 0.1 mM EDTA and 1 mM DTT. Cell pellets were stored at −80 °C until further use.

For membrane preparation, pellets were thawed, homogenized in 10 volumes of assay buffer using an Ultra Turrax homogenizer, and centrifuged twice at 35,000 × *g* for 15 min at 4 °C, with an intermediate 15 min incubation at 37 °C. The final membrane pellets were resuspended in assay buffer consisting of 50 mM Tris–HCl, 0.1 mM EDTA, 4 mM MgCl_2_, and 0.1% ascorbate. The membrane protein concentration was adjusted such that 3 μg of protein was used per well in all binding assays.

Kinetic binding experiments were performed using the radioligand [^3^H]­ketanserin (Revvity). The kinetic parameters of the radioligand were determined in preliminary experiments, yielding *k*_off_ = 0.1679 min^–1^ and *k*_on_ = 9.35 × 10^7^ M^–1^ min^–1^, corresponding to a calculated K_D_ of 1.8 nM and a RT of approximately 6 min, consistent with literature values.

Competition association assays were conducted by simultaneous addition of [^3^H]­ketanserin (1 nM) and unlabeled test compounds at final concentrations of 10 or 100 nM. Incubations were performed at room temperature (22 °C) for increasing time intervals (1, 2, 3, 5, 10, 15, 30, and 60 min). At each time point, reactions were terminated by rapid vacuum filtration through Unifilter plates using a 96-well harvester, followed by quantification of filter-bound radioactivity using a MicroBeta plate reader (Revvity).

Nonspecific binding was determined in the presence of 10 μM methiothepine. Under the applied assay conditions, radioligand depletion did not exceed 10% of the total added radioligand.

All experiments were performed in duplicate, with at least two independent biological replicates. Data points were excluded only in cases of clear technical artifacts (e.g., pipetting errors), with a maximum of three excluded points per compound data set.

Kinetic parameters for unlabeled ligands were obtained by nonlinear regression using the Motulsky-Mahan model (“Kinetics of competitive binding”) implemented in GraphPad Prism 10, assuming competitive binding at the orthosteric site and using fixed radioligand kinetic parameters. The equilibrium dissociation constant was calculated from the kinetic rate constants as *K*_D_ = *k*_off_/*k*_on_. The RT of each compound was calculated as the reciprocal of its dissociation rate constant (RT = 1/*k*_off_).

Representative competition association curves for selected compounds are provided in the Supporting Information to facilitate inspection of kinetic behavior beyond the summarized parameters reported.

## Supplementary Material



## Notes

Additional analyses and spectral data; representative interaction diagrams illustrating the interactions between the 5-HT2A receptor and the ligand as obtained from docking studies; interactions between individual amino acid residues and the ligand as identified through molecular dynamics simulations; different colors denote distinct types of interactions; ligand RMSD relative to the initial frame for individual compounds; time dependence of the TM5 bulge, quantified as the distance between C2 atoms of residues Ser2425 × 46 and Gly3697 × 41, shown for all compounds; time evolution of the ionic lock distance, measured as the distance between N (NH2) atom of Arg1733.50 and Cδ of Glu3186.30, shown for all compounds; number of simulation frames in which a contact with a given residue is observed; percentages exceeding 100% indicate the presence of frames in which more than one interaction with the same residue occurs; number of simulation frames in which a contact of a given type with a specific residue is observed; measurement of the angle between one of the compound’s phenylalanine rings and the phenyl ring of Trp3366.48 and Phe3406.52 during MD simulations; dihedral angle values; plot showing the relationship between dihedral angles; correlation plots of dihedral angles; structural validation against the risperidone-bound 5-HT2A receptor; superposition of the final MD simulation frame with the experimental crystal structure of the 5-HT2A receptor cocrystallized with risperidone; interactions between individual amino acid residues and the ligand as identified through RAMD unbinding simulations; computational estimation of ligand residence times; quantitative analysis of dissociation kinetics for the investigated compounds; data illustrates the cumulative distribution of simulated unbinding events used to extrapolate absolute RT values, as generated by the automated analysis protocol within the Schrödinger suite; interactions between individual amino acid residues and the ligand as identified through iMetaD simulation; global interaction density map; spatiotemporal mapping of ligand–residue contacts derived from iMetaD simulations; heatmap illustrates the frequency and persistence of individual noncovalent interactions throughout the normalized simulation time; kinetic profiling of specific interaction ruptures during ligand egress; charts quantify the mean temporal point of dissociation for individual ligand–receptor contacts, expressed as a percentage of the normalized simulation time; rupture point is strictly defined as the threshold beyond which a given interaction remains undetected for a contiguous 10% of the trajectory; panels denote the dissociation profiles for distinct noncovalent modalities; characterization of the intermediate and terminal phases of ligand egress, frequencies of transient interactions formed with secondary receptor residues following the targeted rupture of primary orthosteric anchors, dominant terminal contacts observed during the final 5% of the unbinding trajectories, immediately preceding complete ligand dissociation; global interaction density map for the F3406.52A and W3366.48A mutant; consensus map of ligand–residue interactions identified across iMetaD trajectories for the 5-HT2A receptor carrying the F3406.52A and W3366.48A substitutions; ^1^H NMR and ^13^C NMR spectrum for compounds **1–13**; HPLC-MS trace and spectrum for compounds **1–12**; results of the second MD simulation for the selected compounds; and representative competition association curves (PDF)

## Abbreviations

CVs: collective variables
EBP: extended binding pocket
ECL2: extracellular loop 2
GPCRs: G protein-coupled receptors
iMetaD: infrequent metadynamics
*K*_*i*_: inhibition constant
KS: Kolmogorov–Smirnov
MetaD: metadynamics
MD: molecular dynamics
OBP: orthosteric binding pocket
RAMD: random acceleration MD
REMD: replica exchange MD
RT: residence time
SKR: structure–kinetics relationship
TFA: trifluoroacetic acid
TM5: transmembrane helix 5.
