# Supplementary material for: Decoding Prolonged Residence Time of 5‑HT2A Receptor Antagonists: Insights from Ritanserin Derivatives
Source: J Chem Inf Model. 2026 May 11;66(10):6141–58. doi: 10.1021/acs.jcim.6c00316 (PMC13308886; doi:10.1021/acs.jcim.6c00316)
Supplement: Supplementary file 1 [file ci6c00316_si_001.pdf]

Decoding Prolonged Residence Time of 5-HT_{2A} Receptor Antagonists: Insights from Ritanserin Derivatives

Szymon K. Kordylewski[1], Kinga Kurowska[1], Krystyna Nędza[1], Dorota Satała[1,2], Ryszard Bugno[1], Sabina Podlewska[1,*]

[1]Maj Institute of Pharmacology Polish Academy of Sciences, Smetna Street, 31-315 Krakow, Poland

[2]Department of Comparative Biochemistry and Bioanalytics, Faculty of Biochemistry, Biophysics and Biotechnology, Jagiellonian University, Gronostajowa 7, Kraków, 30-387, Poland

e-mail: smusz@if-pan.krakow.pl

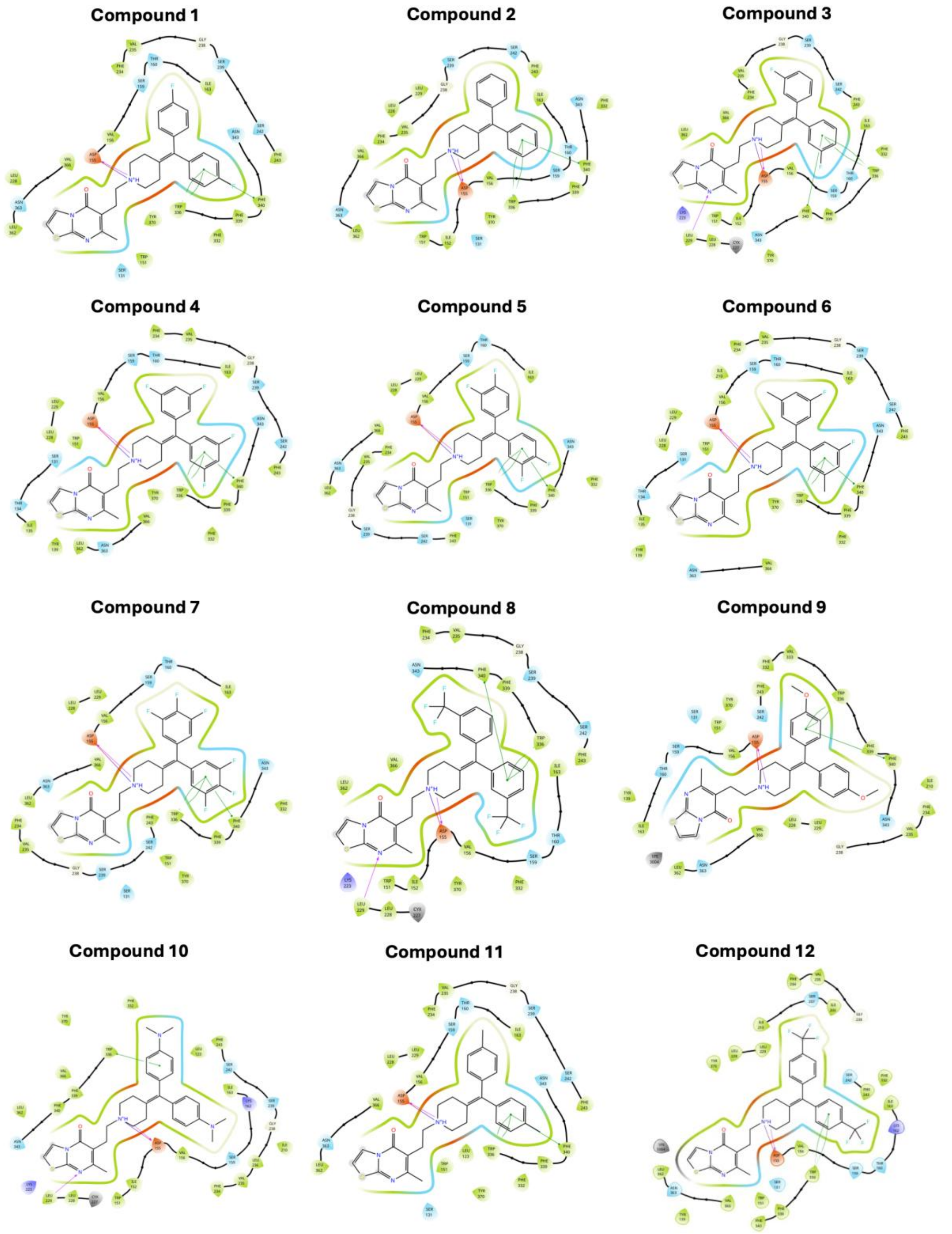

Figure S1. Representative interaction diagrams illustrating the interactions between the 5-HT$_{2A}$ receptor (PDB ID: 6A93) and the ligand as obtained from docking studies.

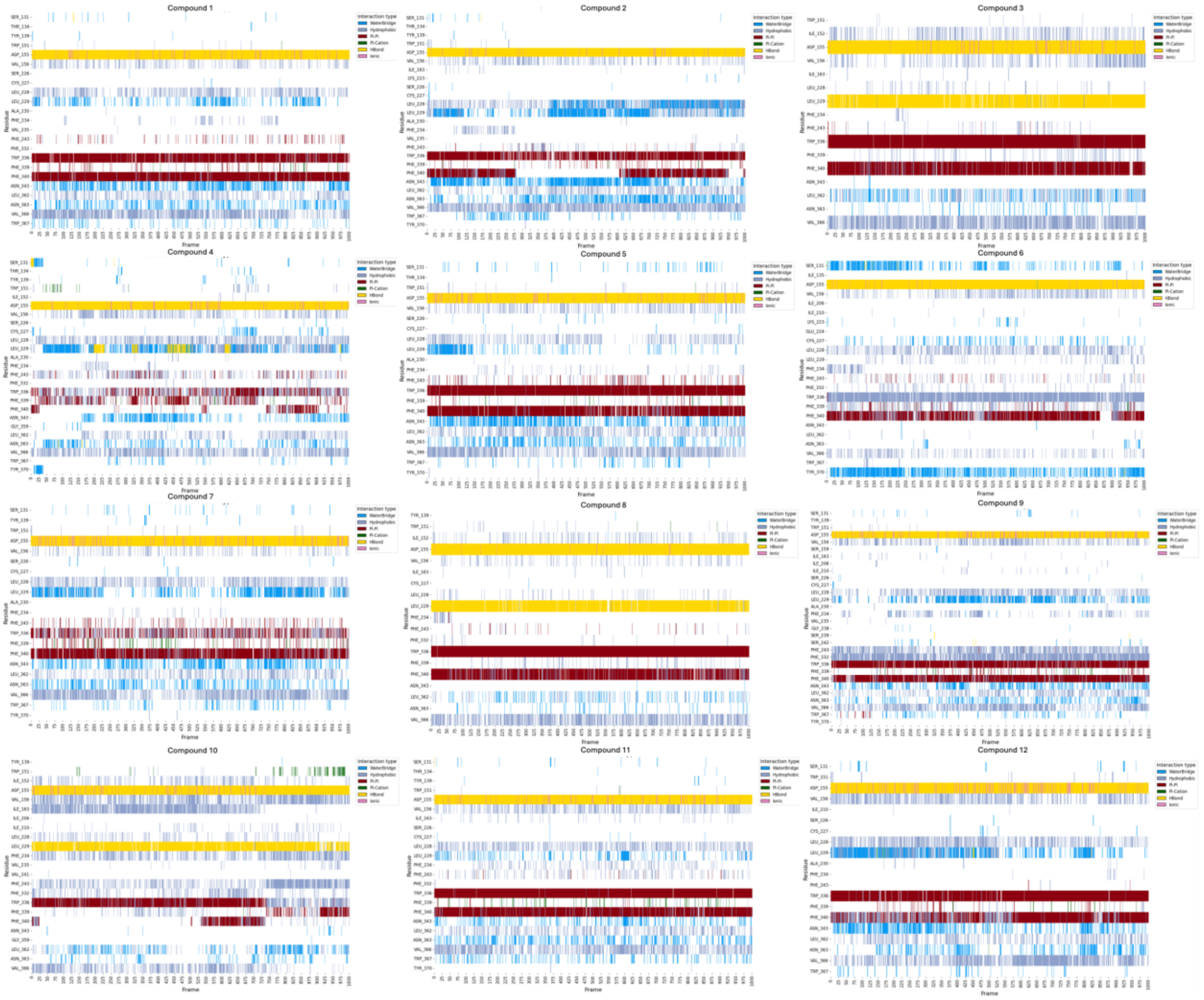

Figure S2. Interactions between individual amino acid residues and the ligand as identified through molecular dynamics simulations. Different colors denote distinct types of interactions: water bridge – blue; hydrophobic – blue-gray; π–π – ruby; π–cation – green; hydrogen bond – yellow; ionic – magenta.

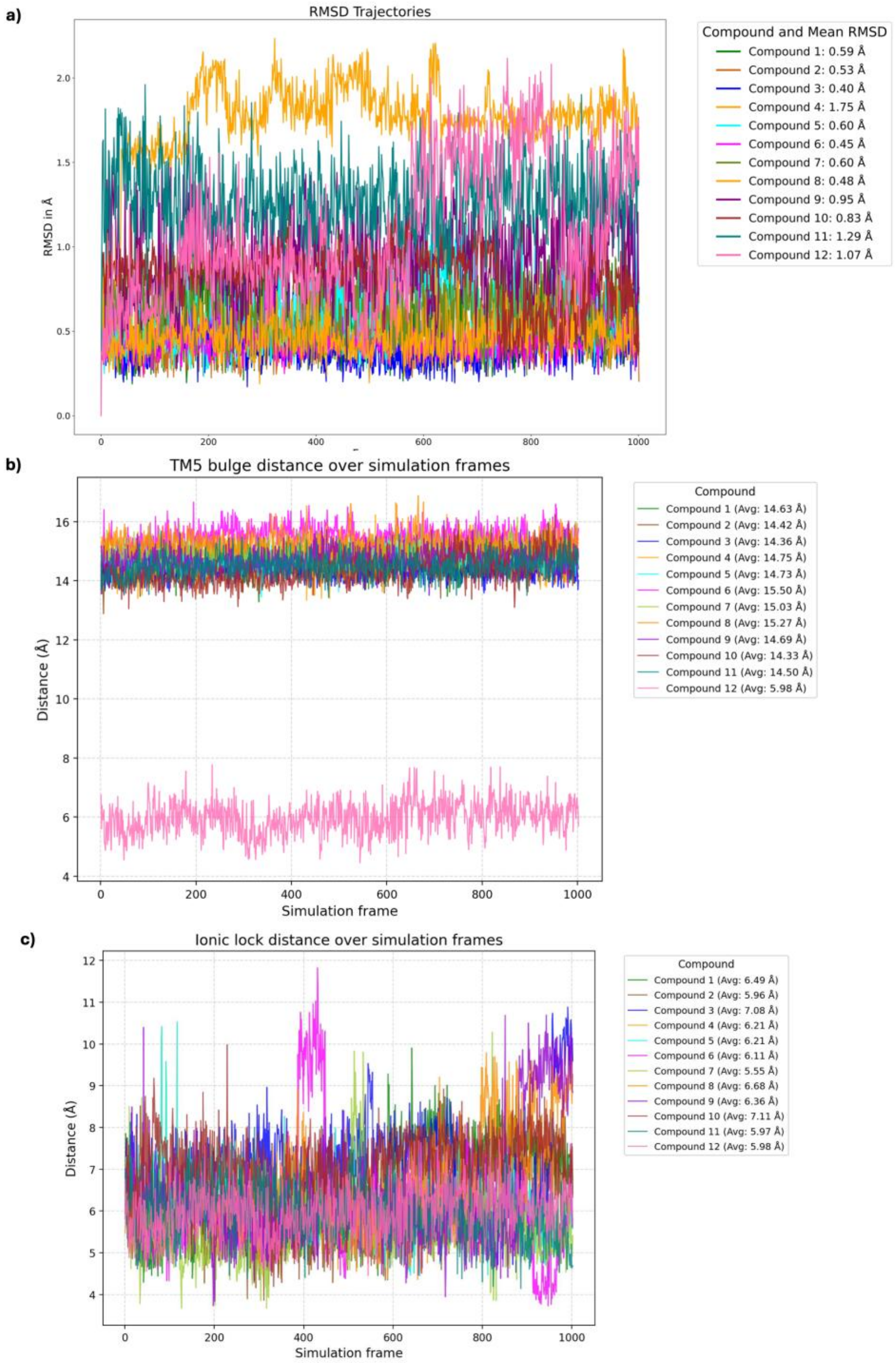

Figure S3. (a) Ligand RMSD relative to the initial frame for individual compounds; (b) time dependence of the TM5 bulge, quantified as the distance between C_2 atoms of residues $Ser242^{5\times46}$ and $Gly369^{7\times41}$, shown for all compounds; (c) time evolution of the ionic lock distance, measured as the distance between N (NH_2) atom of $Arg173^{3.50}$ and C_δ of $Glu318^{6.30}$, shown for all compounds.

	D155 3x32	V156 3x33	L228 45x51	L229 45x52	W336 6x48	P339 6x51	P340 6x52	N343 6x55	L362 7x34	L363 7x35	V366 7x38	RT = 1/k_{off} ± SEM
**Compound 1**	98%	21%	31%	29%	137%	8%	95%	41%	25%	27%	58%	68 ± 0.96
**Compound 2**	98%	24%	77%	67%	109%	8%	59%	55%	29%	39%	66%	29 ± 3
**Compound 3**	98%	27%	5%	110%	110%	10%	95%	0%	41%	6%	65%	42 ± 0.5
**Compound 4**	99%	32%	38%	92%	73%	39%	17%	27%	22%	22%	50%	8.5 ± 5.5
**Compound 5**	98%	24%	25%	20%	136%	7%	92%	43%	33%	25%	52%	123 ± 11
**Compound 6**	158%	28%	36%	8%	92%	29%	84%	0%	1%	2%	18%	46 ± 1
**Compound 7**	98%	16%	36%	55%	81%	15%	90%	35%	24%	27%	55%	115.5 ± 2.5
**Compound 8**	98%	11%	6%	100%	113%	8%	92%	0%	19%	5%	52%	35 ± 5
**Compound 9**	98%	35%	37%	60%	140%	11%	93%	32%	27%	23%	49%	35.5 ± 4.5
**Compound 10**	98%	63%	13%	102%	89%	24%	23%	1%	30%	9%	33%	-
**Compound 11**	98%	35%	35%	22%	148%	9%	96%	21%	27%	28%	48%	105 ± 4
**Compound 12**	99%	43%	43%	77%	124%	8%	87%	37%	27%	22%	56%	18.5 ± 2.5

Figure S4. Number of simulation frames in which a contact with a given residue is observed. Percentages exceeding 100% indicate the presence of frames in which more than one interaction with the same residue occurs.

Ligand	HBond_155	HBond_229	Pi-Pi_336	Pi-Pi_340	Hydrophobic_336	Hydrophobic_340
Compound 1	94,41%	0,00%	90,72%	93,61%	49,00%	3,69%
Compound 2	96,71%	0,00%	85,13%	50,90%	26,45%	9,28%
Compound 3	95,31%	94,61%	98,80%	89,72%	13,17%	6,89%
Compound 4	94,21%	12,08%	31,64%	12,48%	39,52%	4,59%
Compound 5	92,81%	0,00%	97,80%	89,22%	41,02%	4,89%
Compound 6	97,50%	0,00%	0,10%	70,16%	89,82%	15,97%
Compound 7	88,92%	0,10%	36,03%	81,74%	46,41%	10,48%
Compound 8	98,80%	94,41%	99,90%	79,64%	15,27%	13,87%
Compound 9	86,83%	0,10%	90,12%	85,33%	52,20%	9,58%
Compound 10	92,91%	91,72%	64,17%	15,47%	26,05%	7,68%
Compound 11	93,71%	0,00%	98,80%	90,82%	52,20%	6,79%
Compound 12	86,23%	0,90%	95,21%	62,57%	31,64%	26,75%

Figure S5. Number of simulation frames in which a contact of a given type with a specific residue is observed.

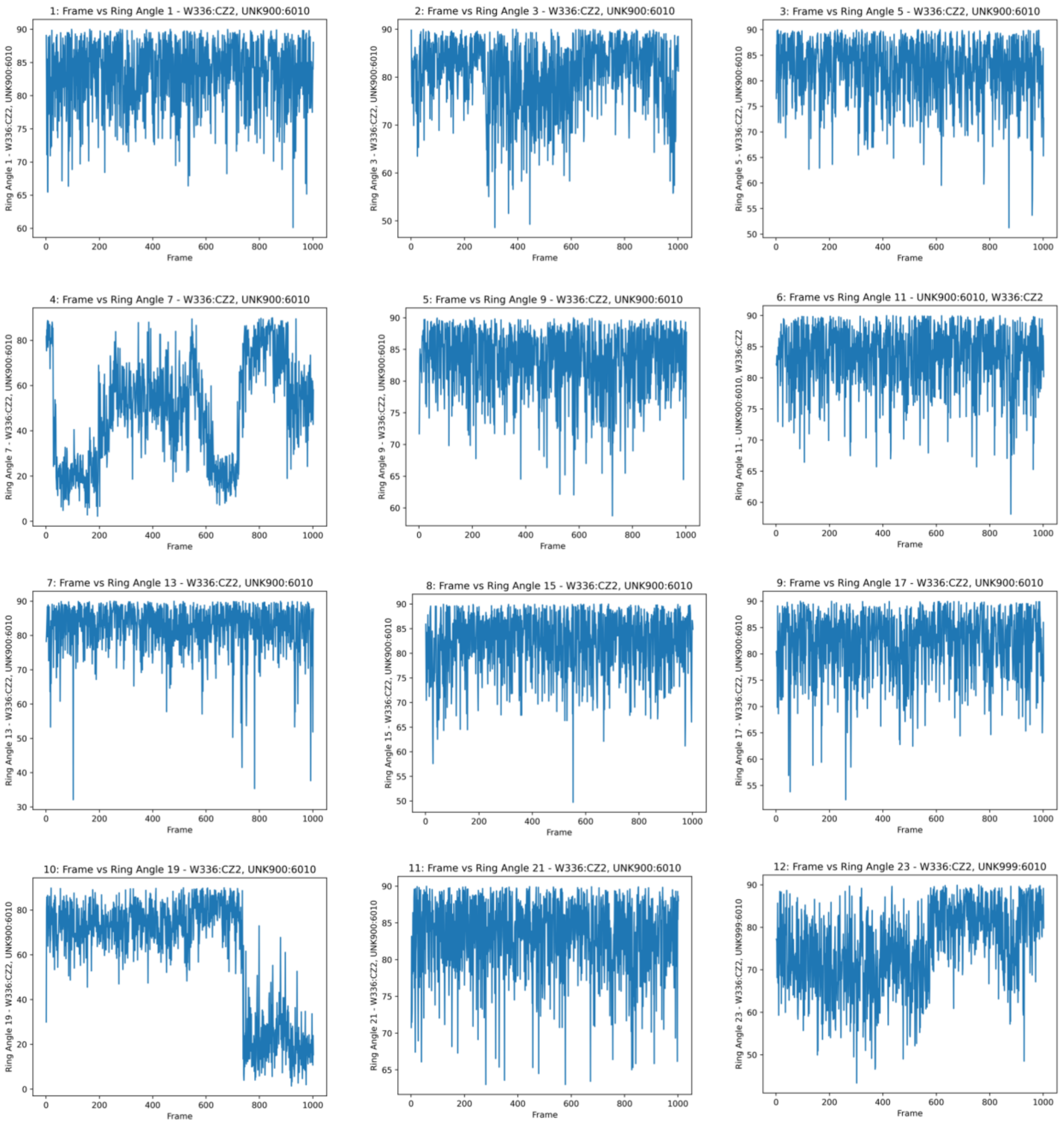

Figure S6. Measurement of the angle between one of the compound's phenylalanine rings (located in the hydrophobic cleft) and the phenyl ring of Trp336$^{6.48}$ during MD simulations.

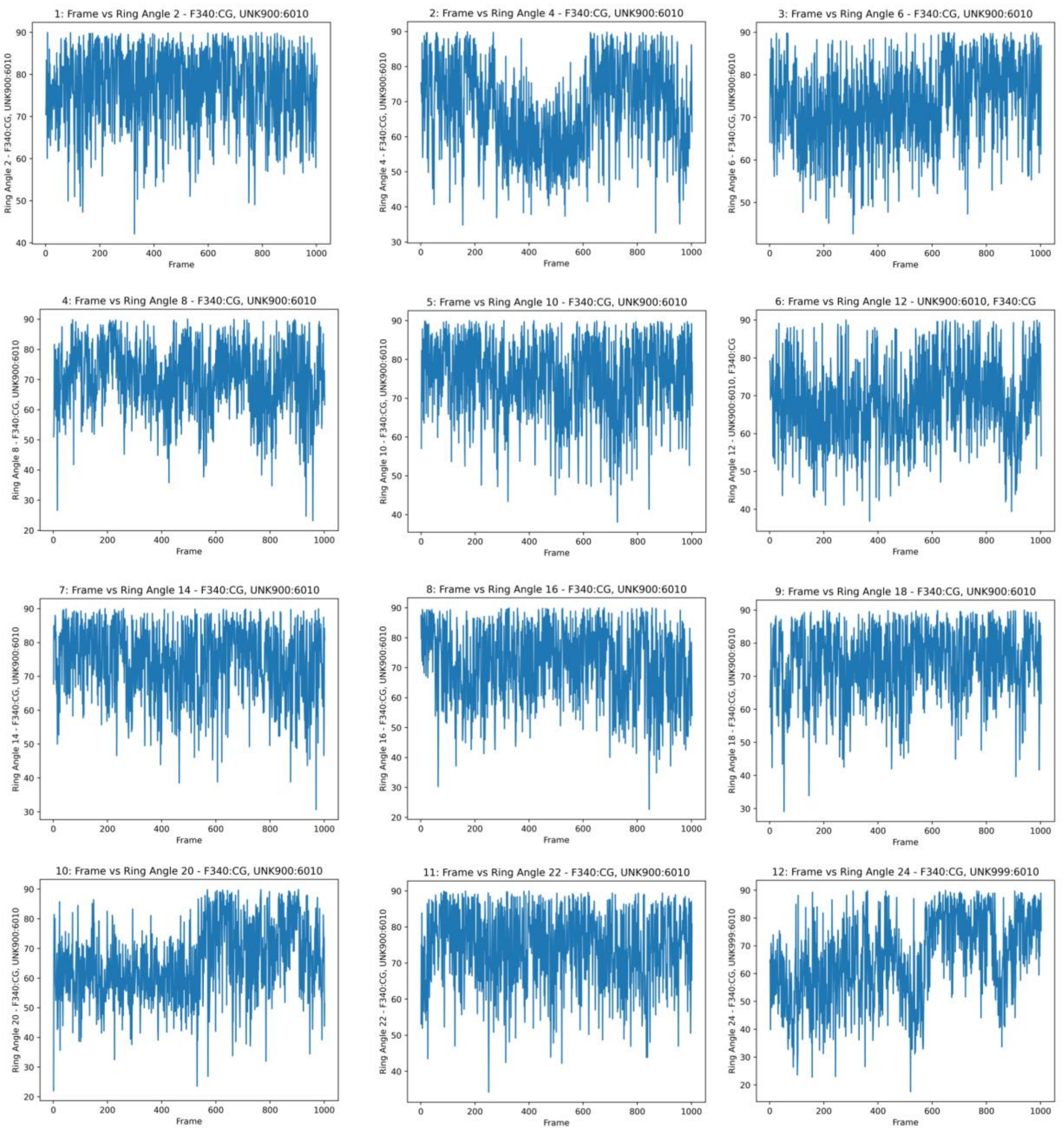

Figure S7. Measurement of the angle between one of the compound's phenylalanine rings (located in the hydrophobic cleft) and the phenyl ring of Phe340$^{6.52}$ during MD simulations.

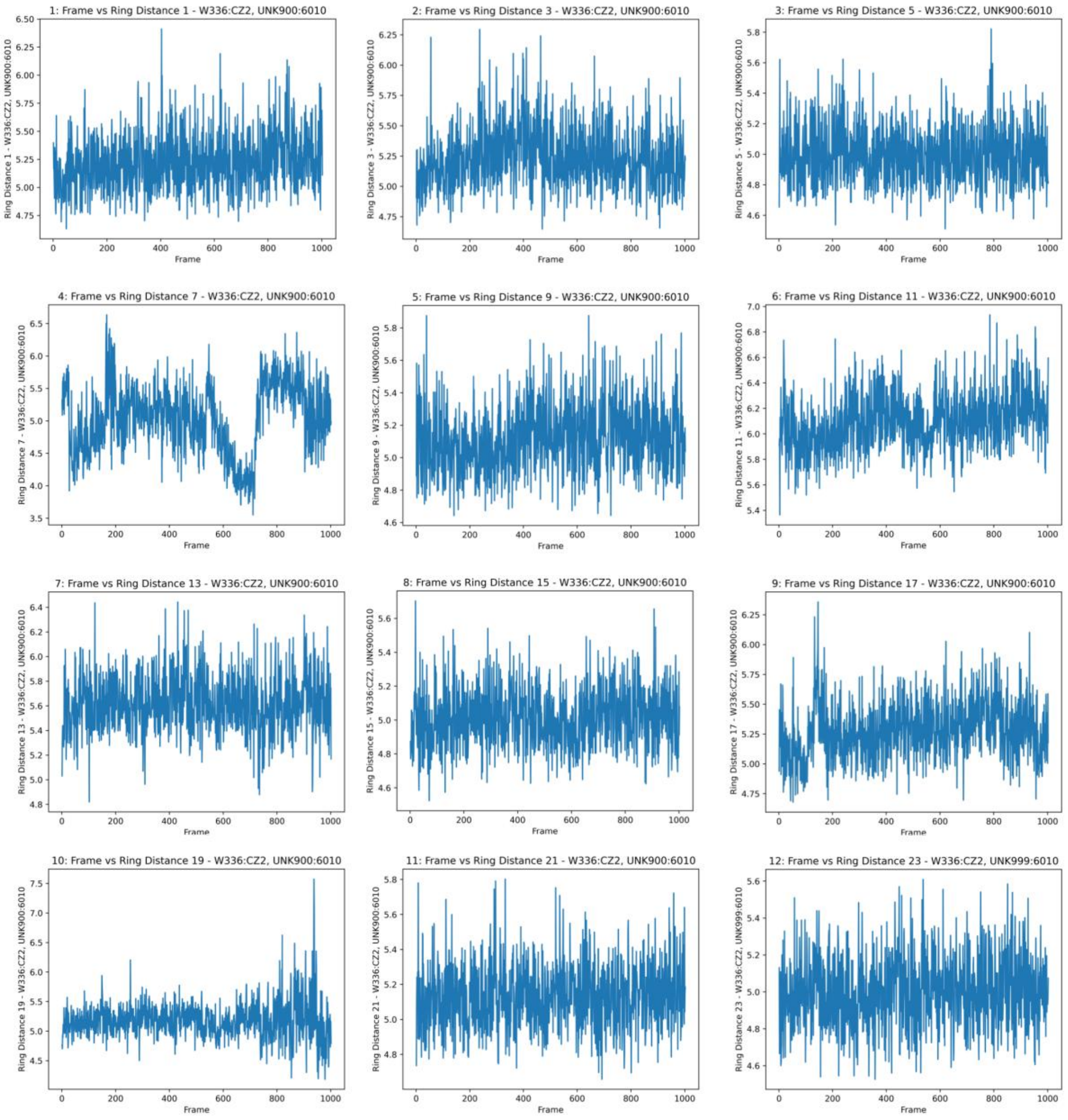

Figure S8. Measurement of the distance between the centroid of one of the compound's phenylalanine rings (located in the hydrophobic cleft) and the centroid of the phenyl ring of Trp336$^{6.48}$ during MD simulations.

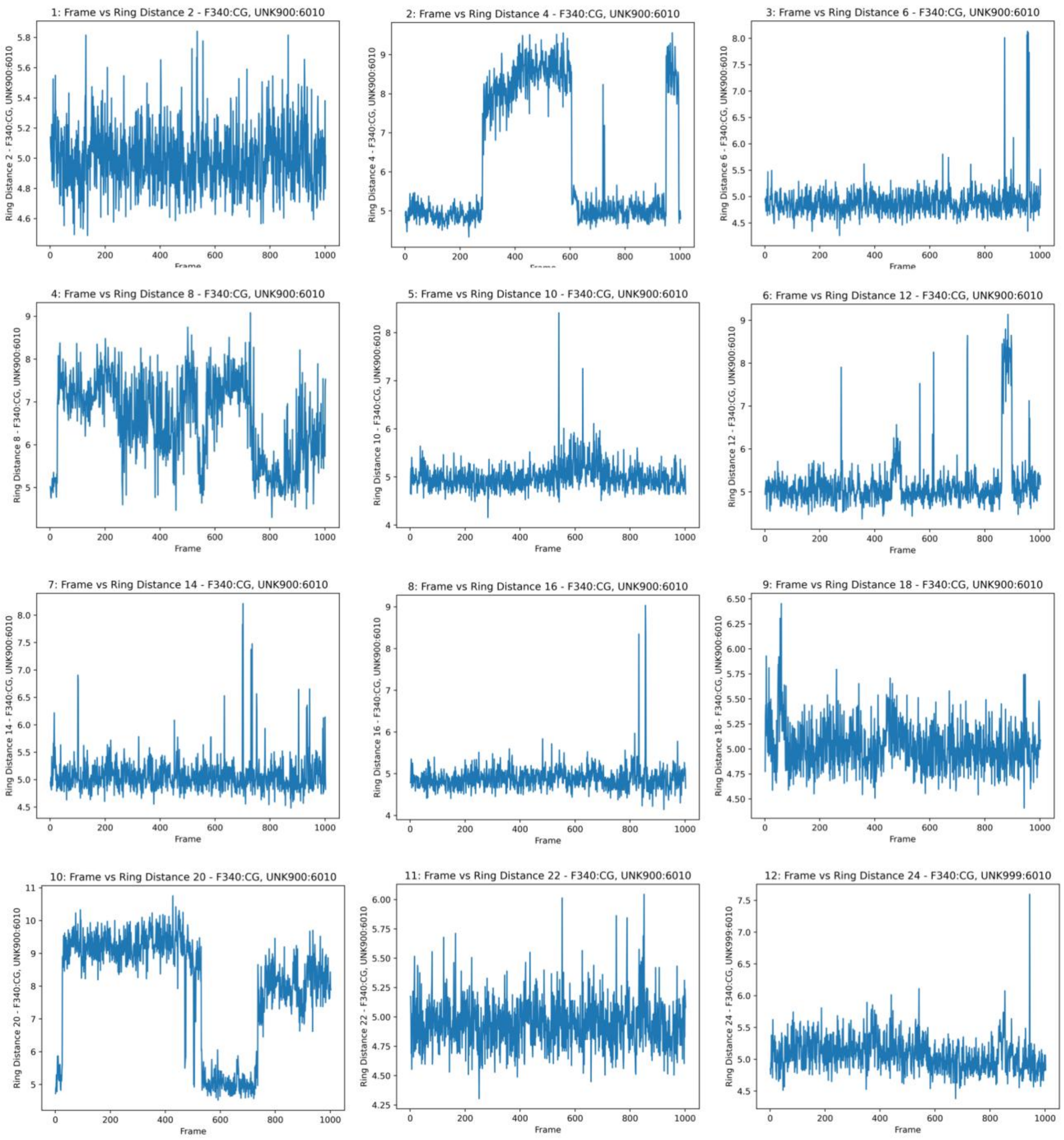

Figure S9. Measurement of the distance between the centroid of one of the compound's phenylalanine rings (located in the hydrophobic cleft) and the centroid of the phenyl ring of Phe340$^{6.52}$ during MD simulations.

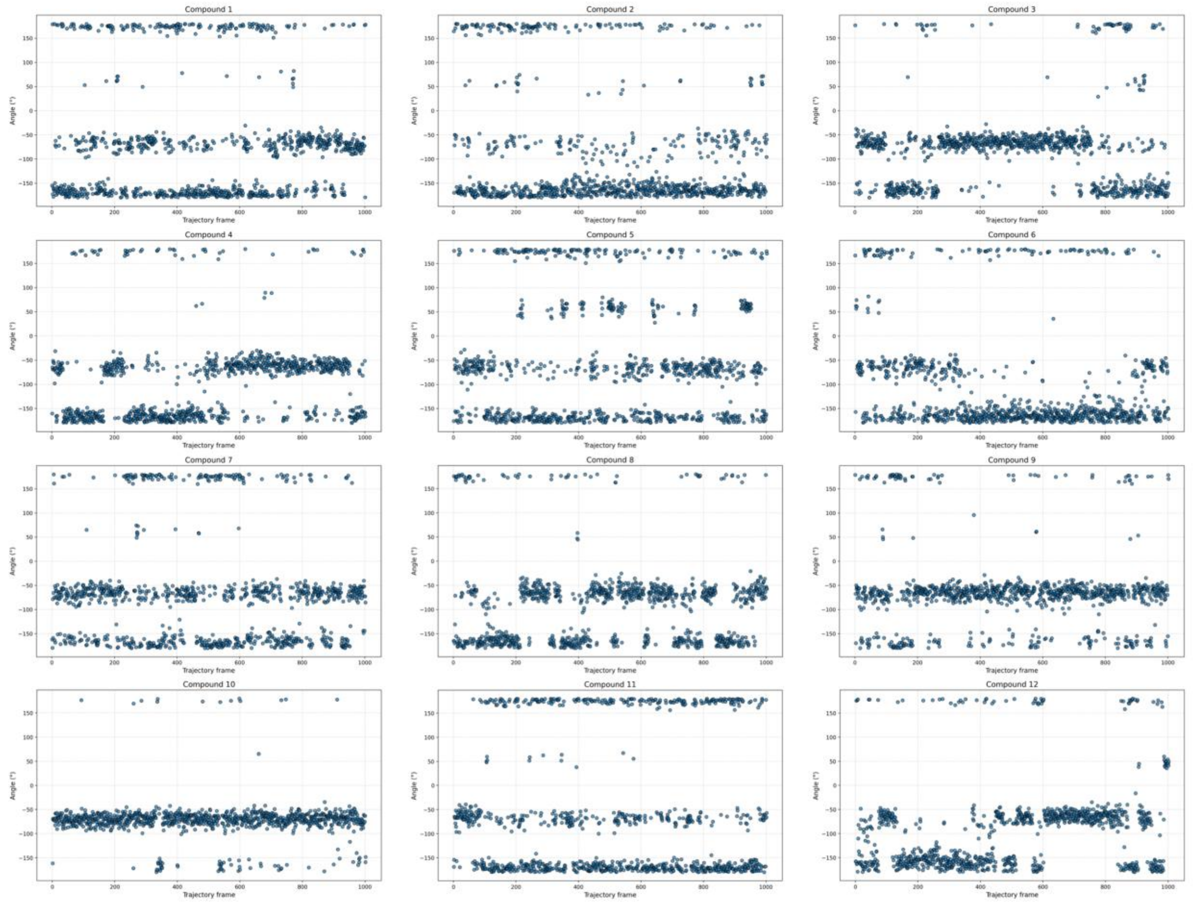

Figure S10. Dihedral angle 1 values – defined as the angle between atoms Cα, Cβ, Cγ, and Cδ2 in Leu229 - measured across individual simulation frames.

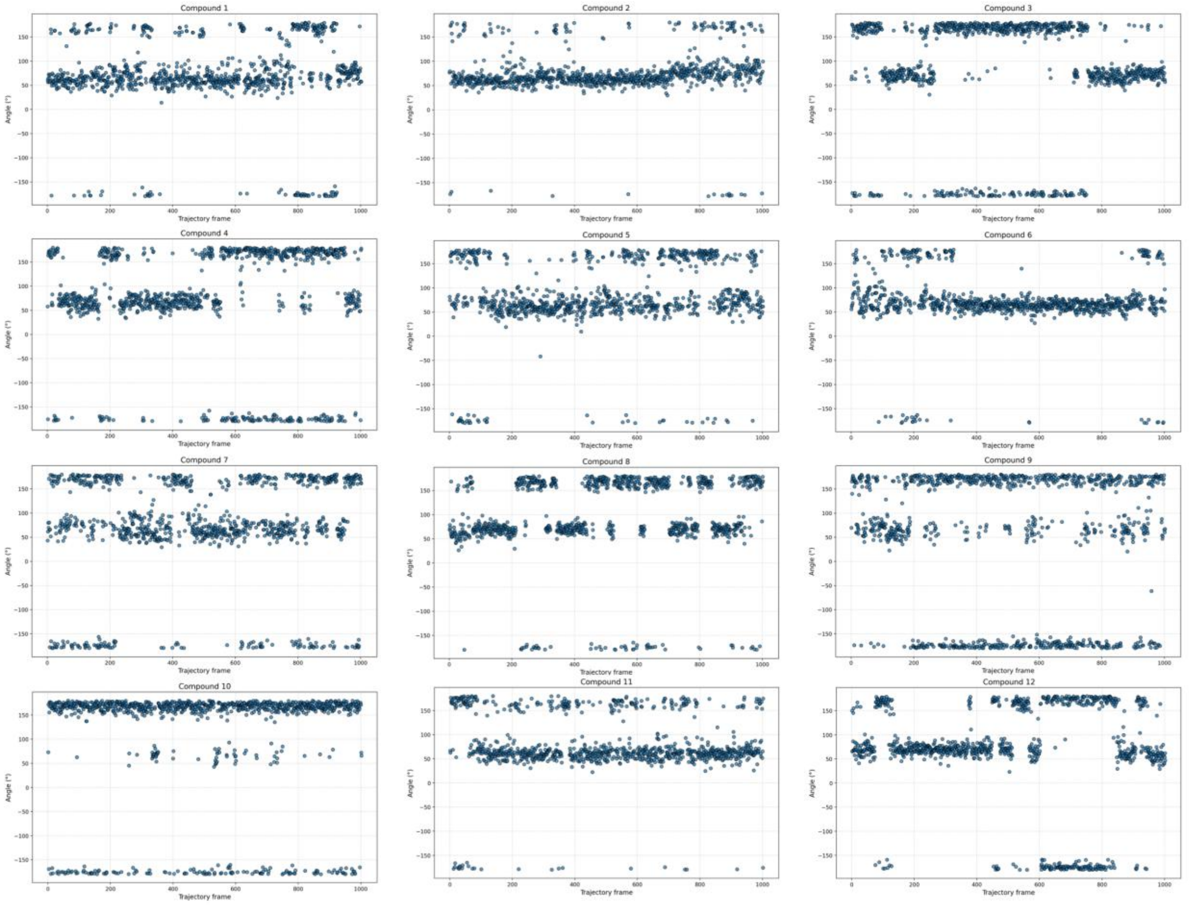

Figure S11. Dihedral angle 2 values – defined as the angle between atoms C, Cα, Cβ and Cγ in Leu229 - measured across individual simulation frames.

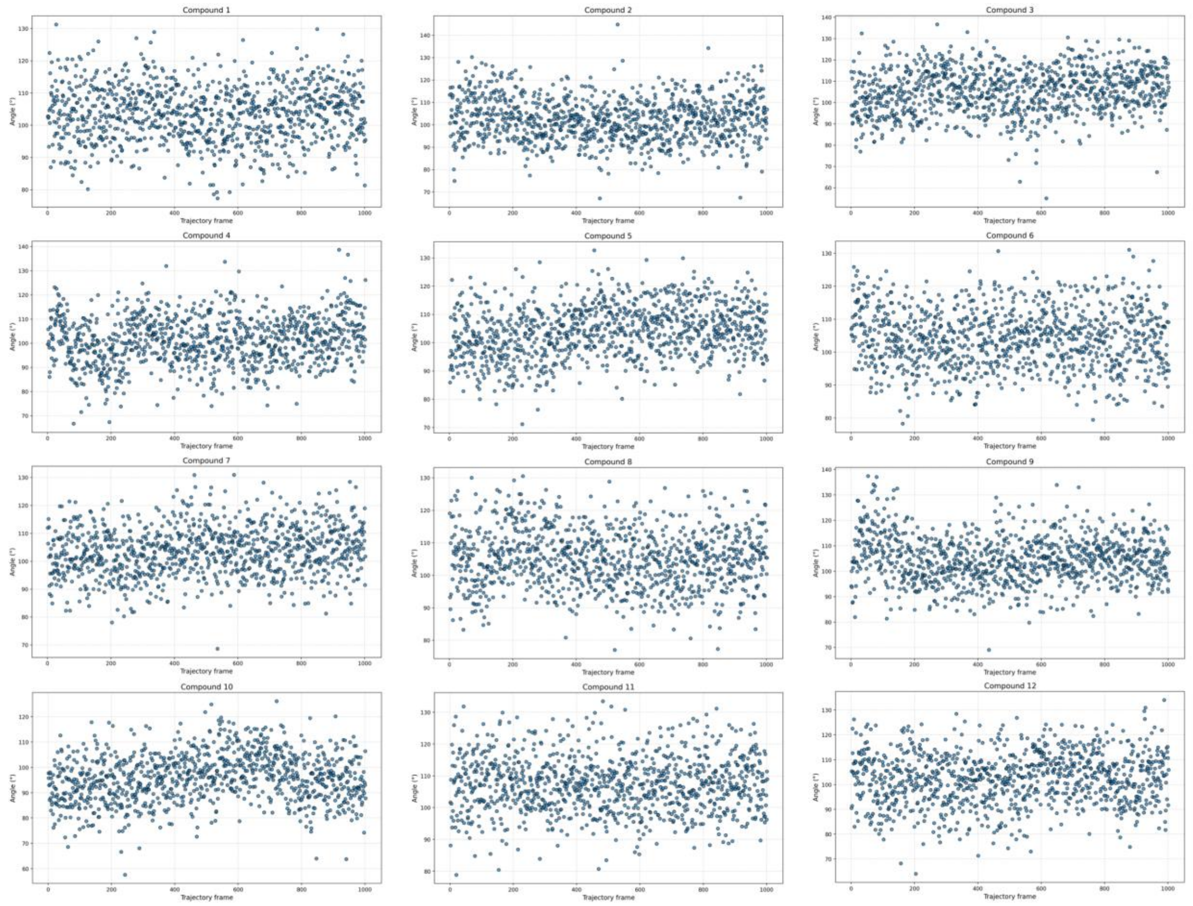

Figure S12. Dihedral angle 3 values – defined as the angle between atoms Cα, Cβ, Cγ, and Cδ2 in Trp336$^{6.48}$ - measured across individual simulation frames.

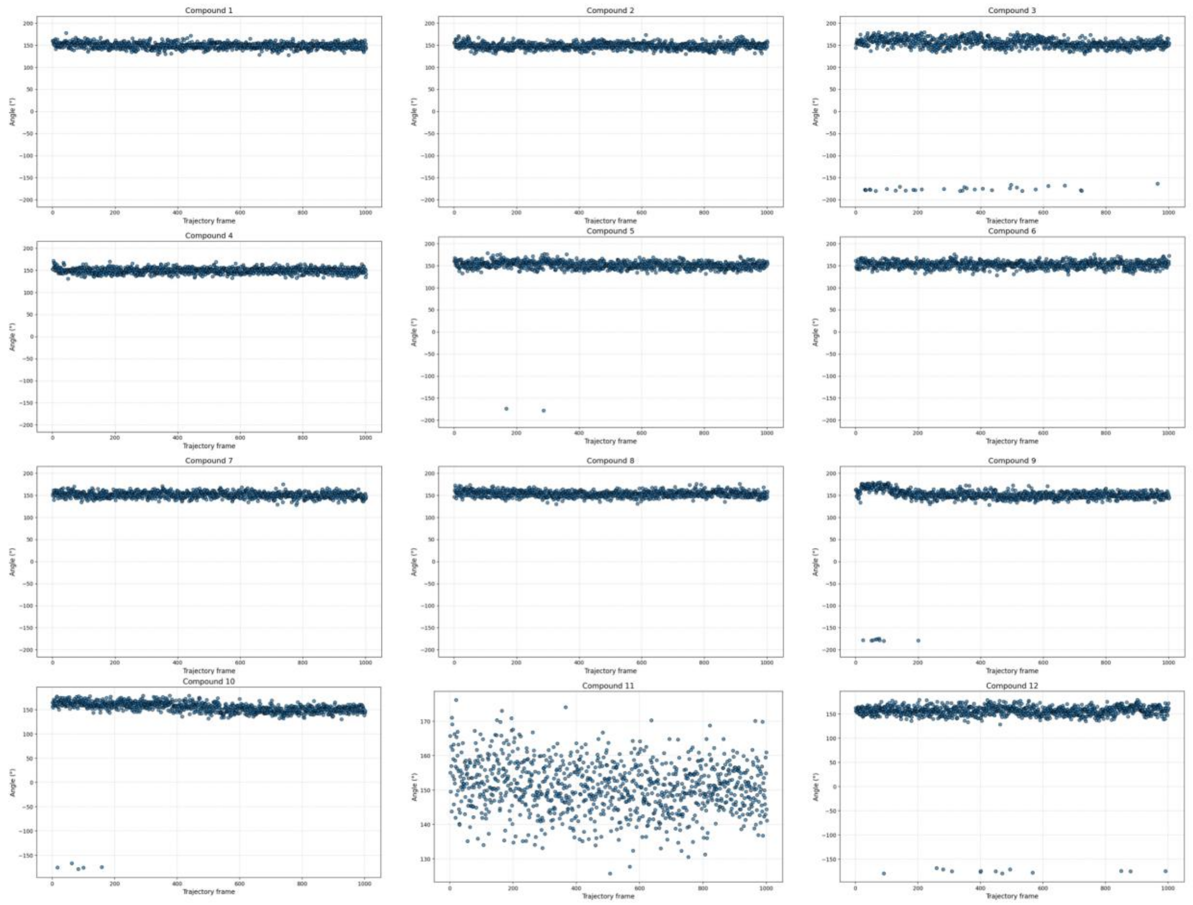

Figure S13. Dihedral angle 4 values – defined as the angle between atoms C, Cα, Cβ and Cγ in Trp336$^{6.48}$ - measured across individual simulation frames.

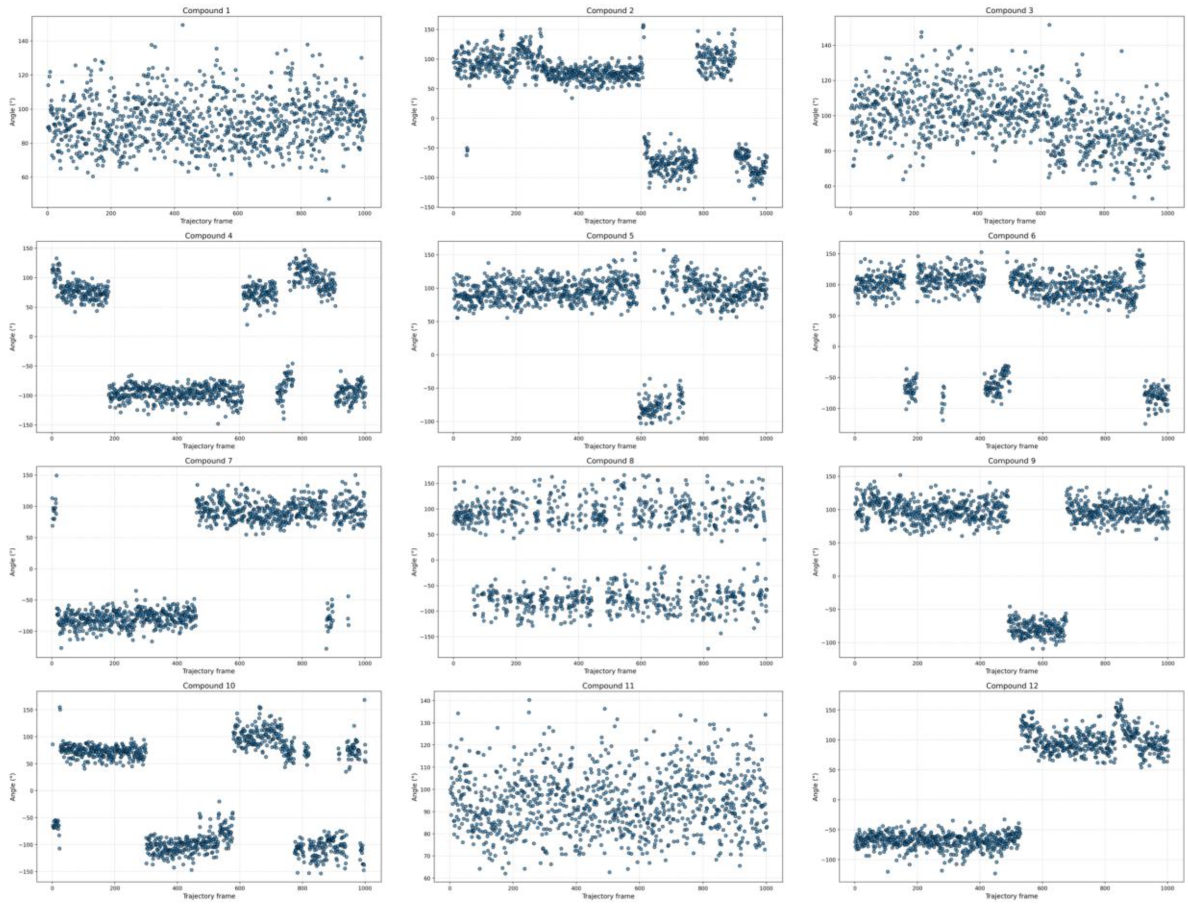

Figure S14. Dihedral angle 5 values – defined as the angle between atoms Cα, Cβ, Cγ, and Cδ2 in Phe340$^{6.52}$ - measured across individual simulation frames.

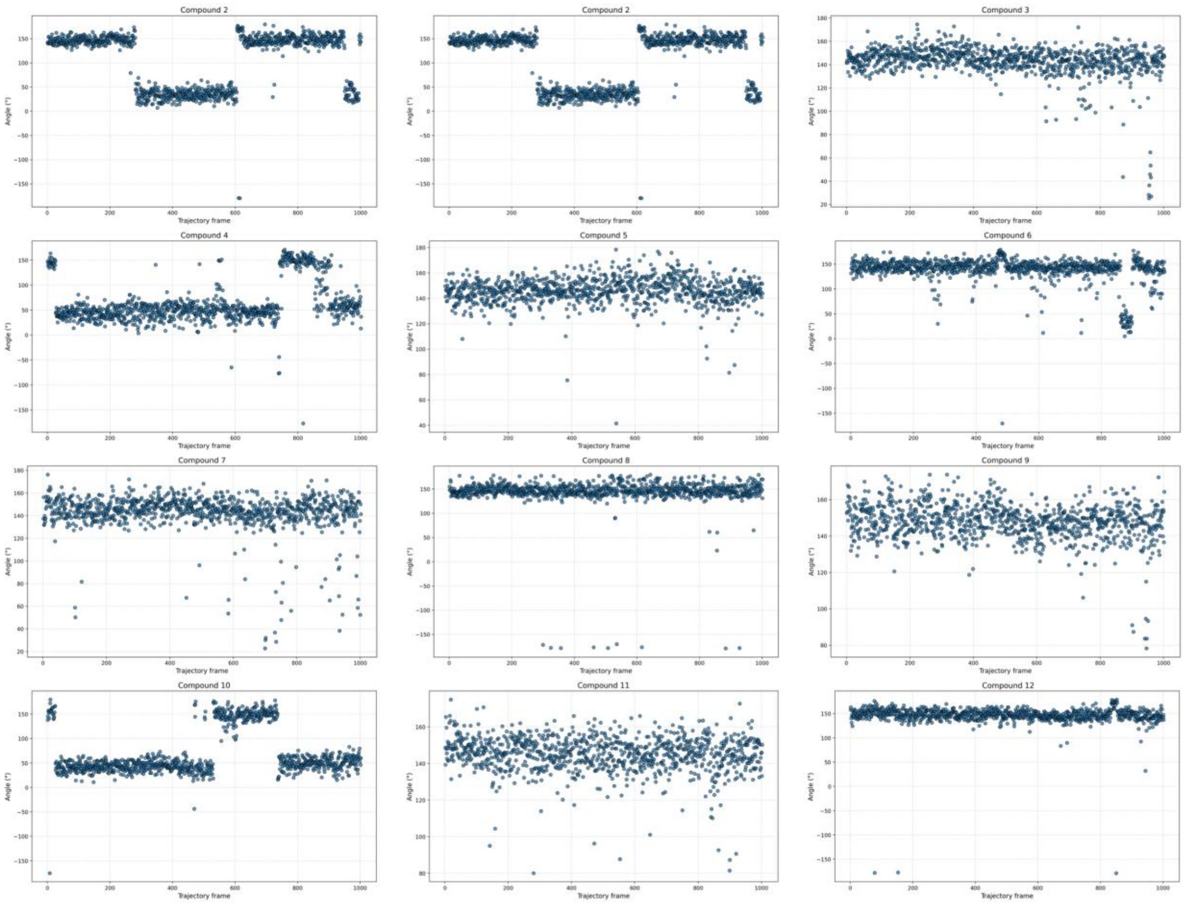

Figure S15. Dihedral angle 6 values – defined as the angle between atoms C, Cα, Cβ and Cγ in Phe340$^{6.52}$ - measured across individual simulation frames.

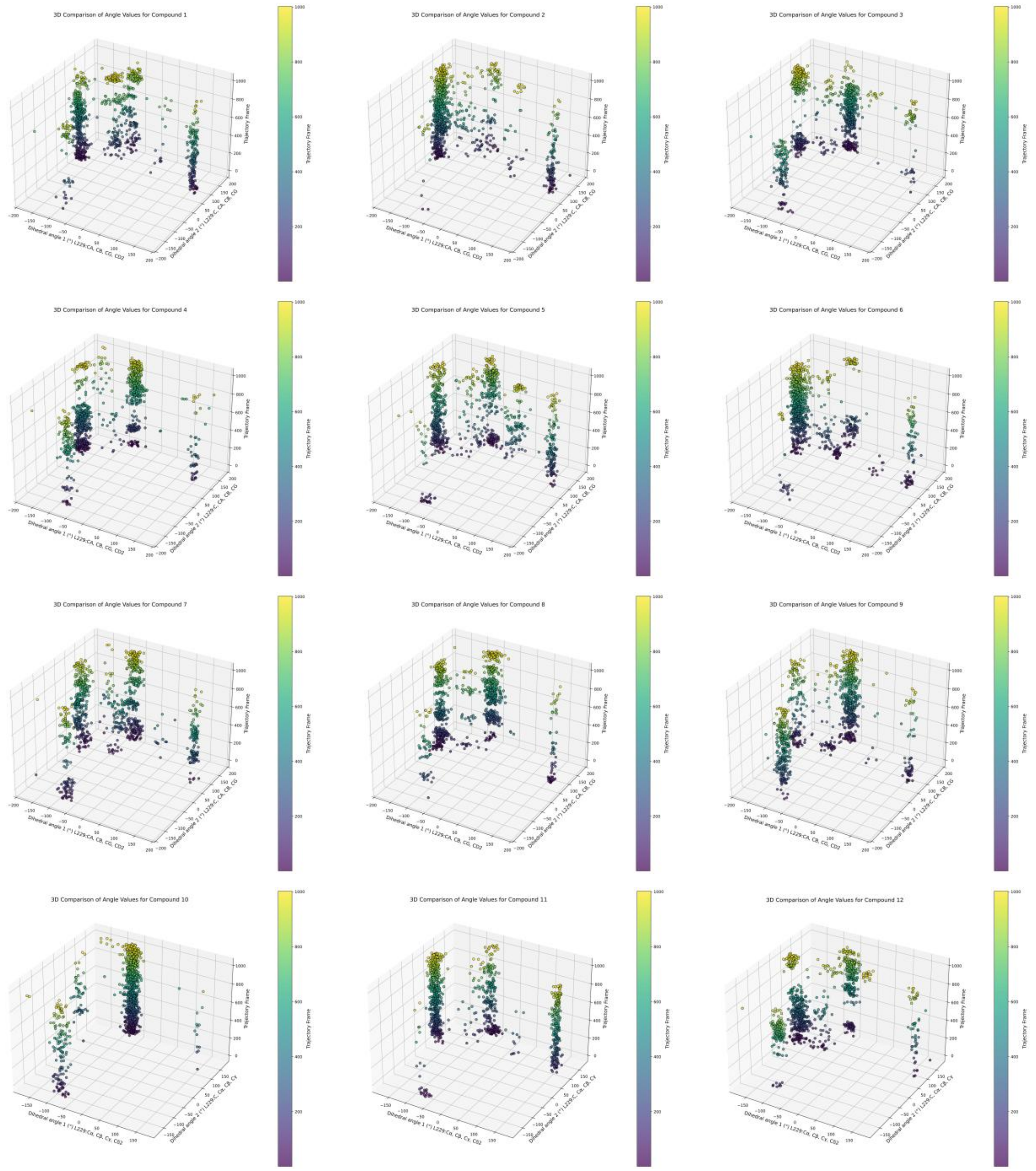

Figure S16. Plot showing the relationship between dihedral angle 1 (defined as the angle between atoms Cα–Cβ–Cγ–Cδ2 in Leu229) and dihedral angle 2 (defined as the angle between atoms C–Cα–Cβ–Cγ in Leu299) across individual frames of the molecular dynamics simulation.

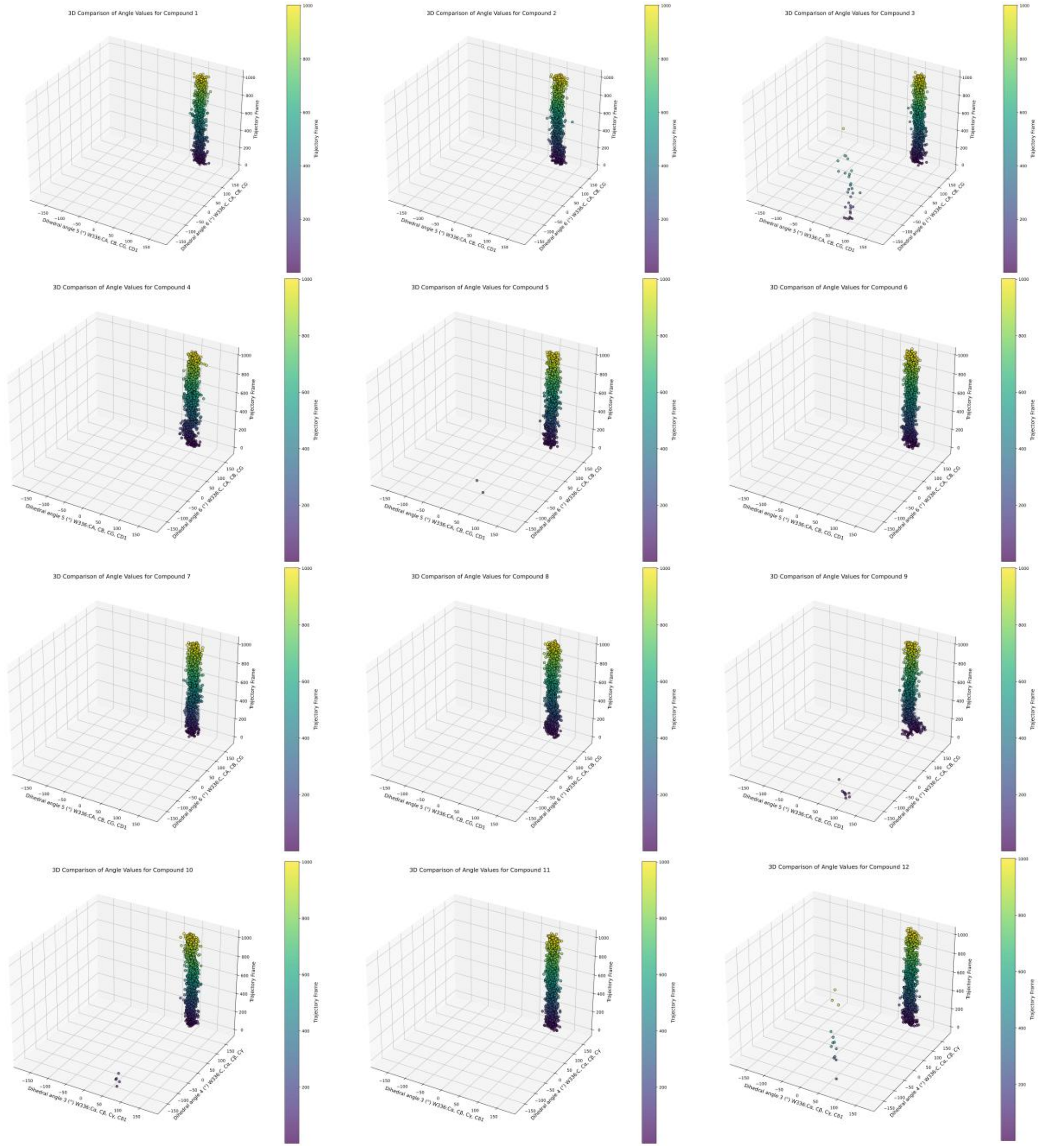

Figure S17. Plot showing the relationship between dihedral angle 3 (defined as the angle between atoms Cα–Cβ–Cγ–Cδ2 in Trp336$^{6.48}$) and dihedral angle 4 (defined as the angle between atoms C–Cα–Cβ–Cγ in Trp336$^{6.48}$) across individual frames of the molecular dynamics simulation.

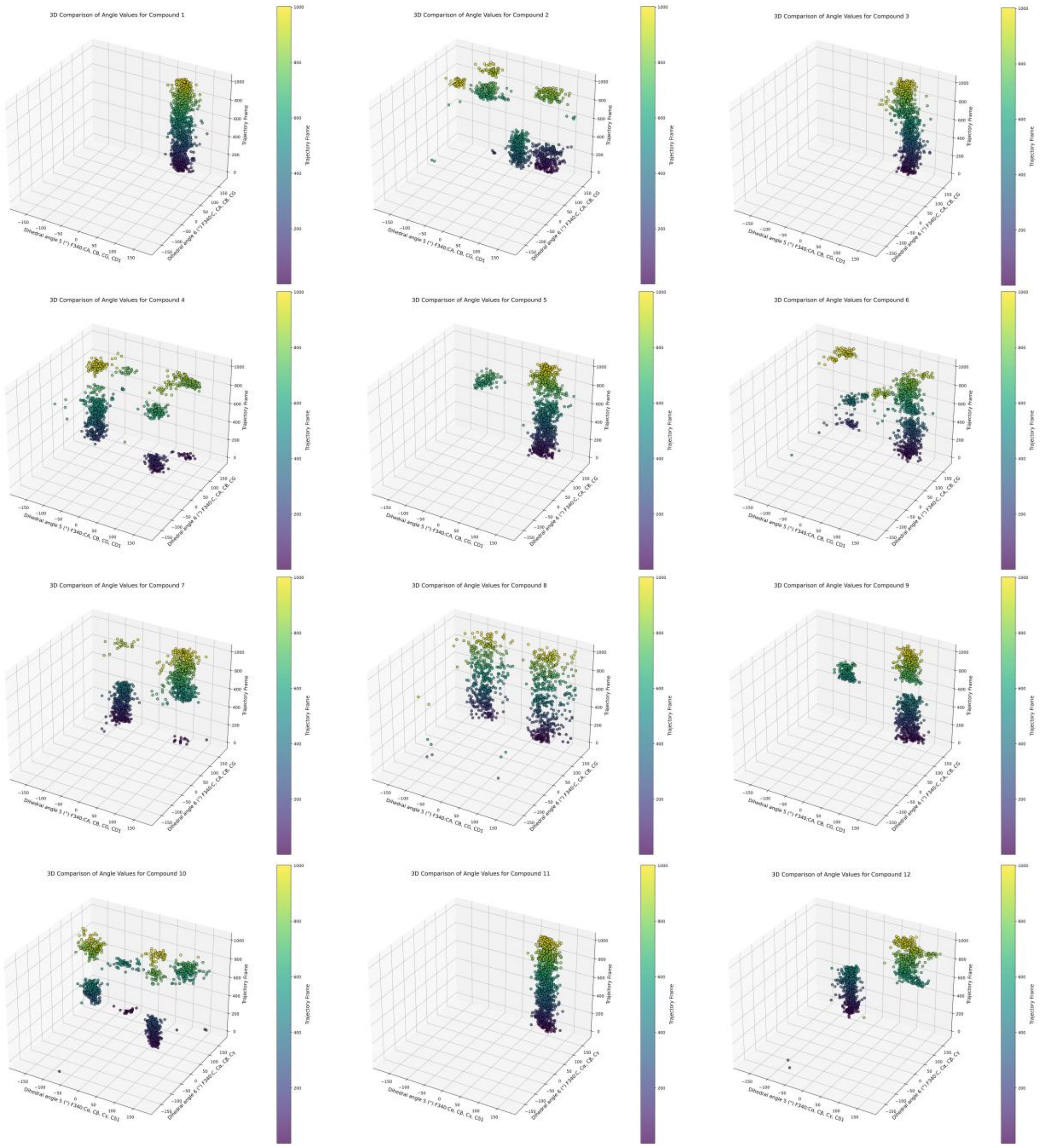

Figure S18. Plot showing the relationship between dihedral angle 5 (defined as the angle between atoms Cα–Cβ–Cγ–Cδ2 in Phe340$^{6.52}$) and dihedral angle 6 (defined as the angle between atoms C–Cα–Cβ–Cγ in Phe340$^{6.52}$) across individual frames of the molecular dynamics simulation.

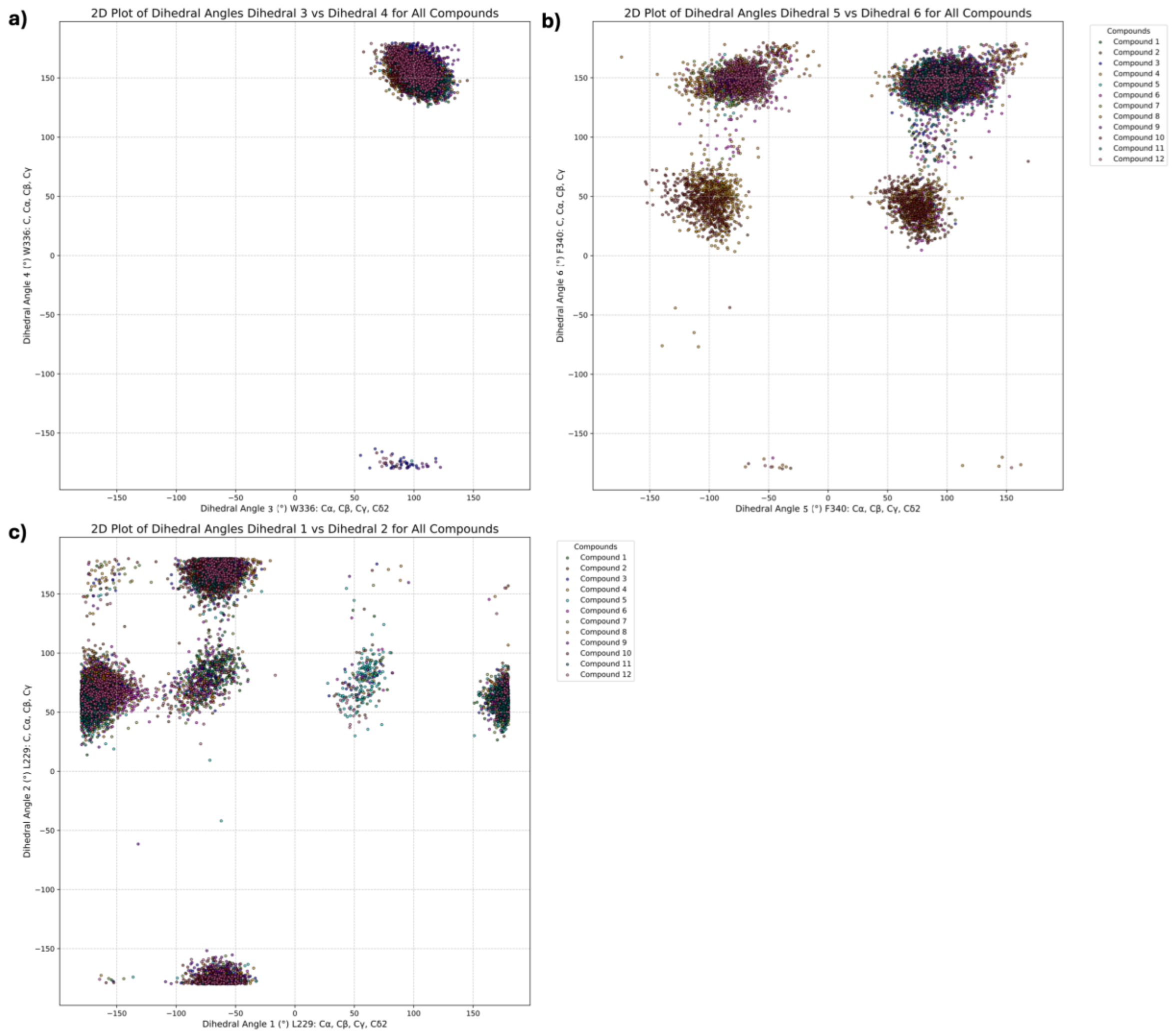

Figure S19. (a) Correlation plots of dihedral angles in Trp336[6.48]. Angle 3 is defined by the C–C_{α}–C_{β}–C_{γ} atoms, and Angle 4 by the C_{α}–C_{β}–C_{γ}–$C_{\delta 2}$ atoms. (b) Correlation plots of the dihedral angles in Phe340[6.52]. Angle 5 is defined by the C–C_{α}–C_{β}–C_{γ} atoms, and Angle 6 by the C_{α}–C_{β}–C_{γ}–$C_{\delta 2}$ atoms. (c) Correlation plots of the dihedral angles in Leu229[ECL2]. Angle 1 is defined by the C–C_{α}–C_{β}–C_{γ} atoms, and Angle 2 by the C_{α}–C_{β}–C_{γ}–$C_{\delta 2}$ atoms.

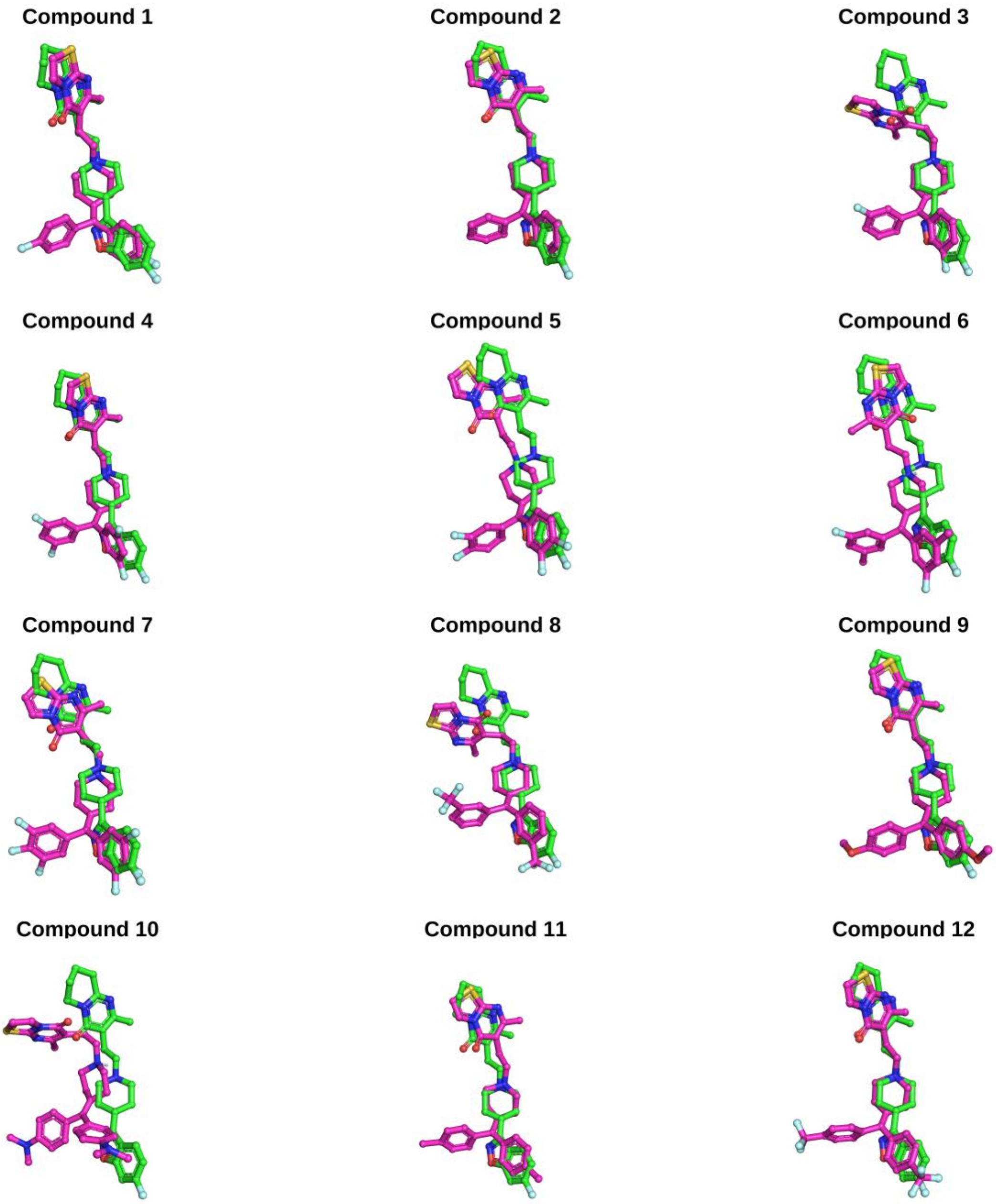

Figure S20. Structural validation against the risperidone-bound 5-HT$_{2A}$ receptor. Superposition of the final MD simulation frame (green sticks) with the experimental crystal structure of the 5-HT$_{2A}$ receptor co-crystallized with risperidone (magenta sticks, PDB ID: 6A93).

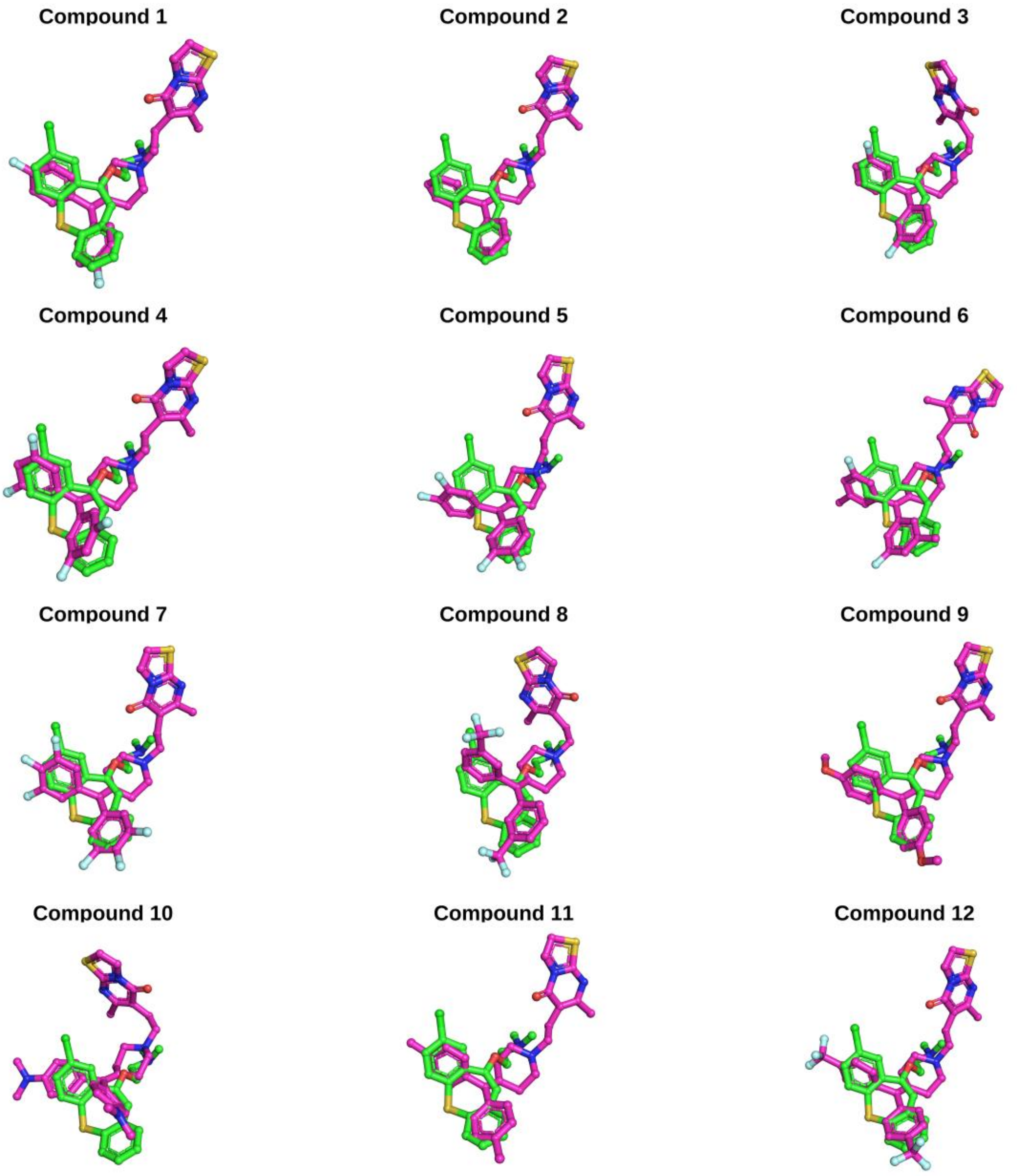

Figure S21. Structural validation against the zotepine-bound 5-HT$_{2A}$ receptor. Superposition of the final MD simulation frame (green sticks) with the experimental crystal structure of the 5-HT$_{2A}$ receptor co-crystallized with zotepine (magenta sticks, PDB ID: 6A94).

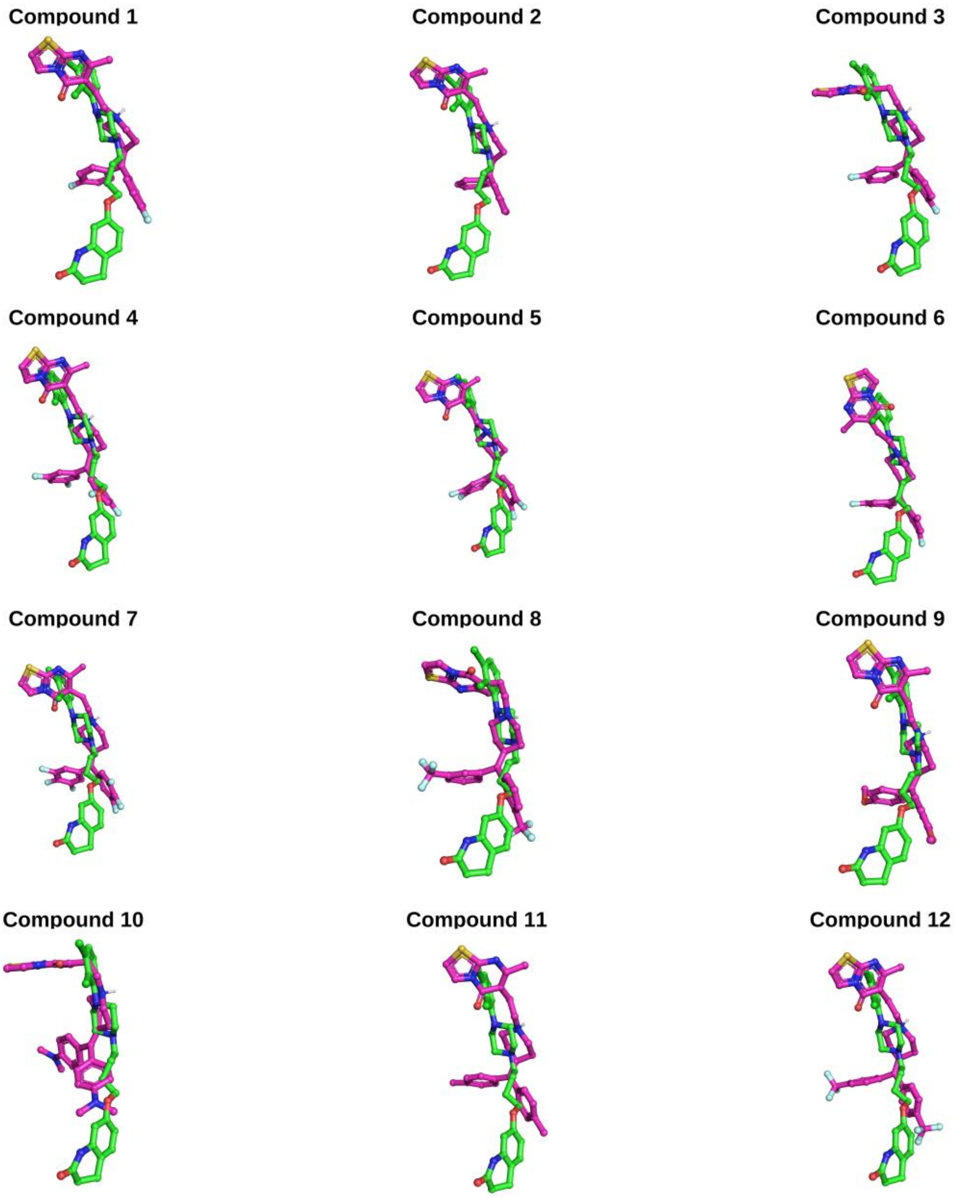

Figure S22. Structural validation against the aripiprazole-bound 5-HT_{2A} receptor. Superposition of the final MD simulation frame (green sticks) with the experimental crystal structure of the 5-HT_{2A} receptor co-crystallized with aripiprazole (magenta sticks, PDB ID: 7VOE).

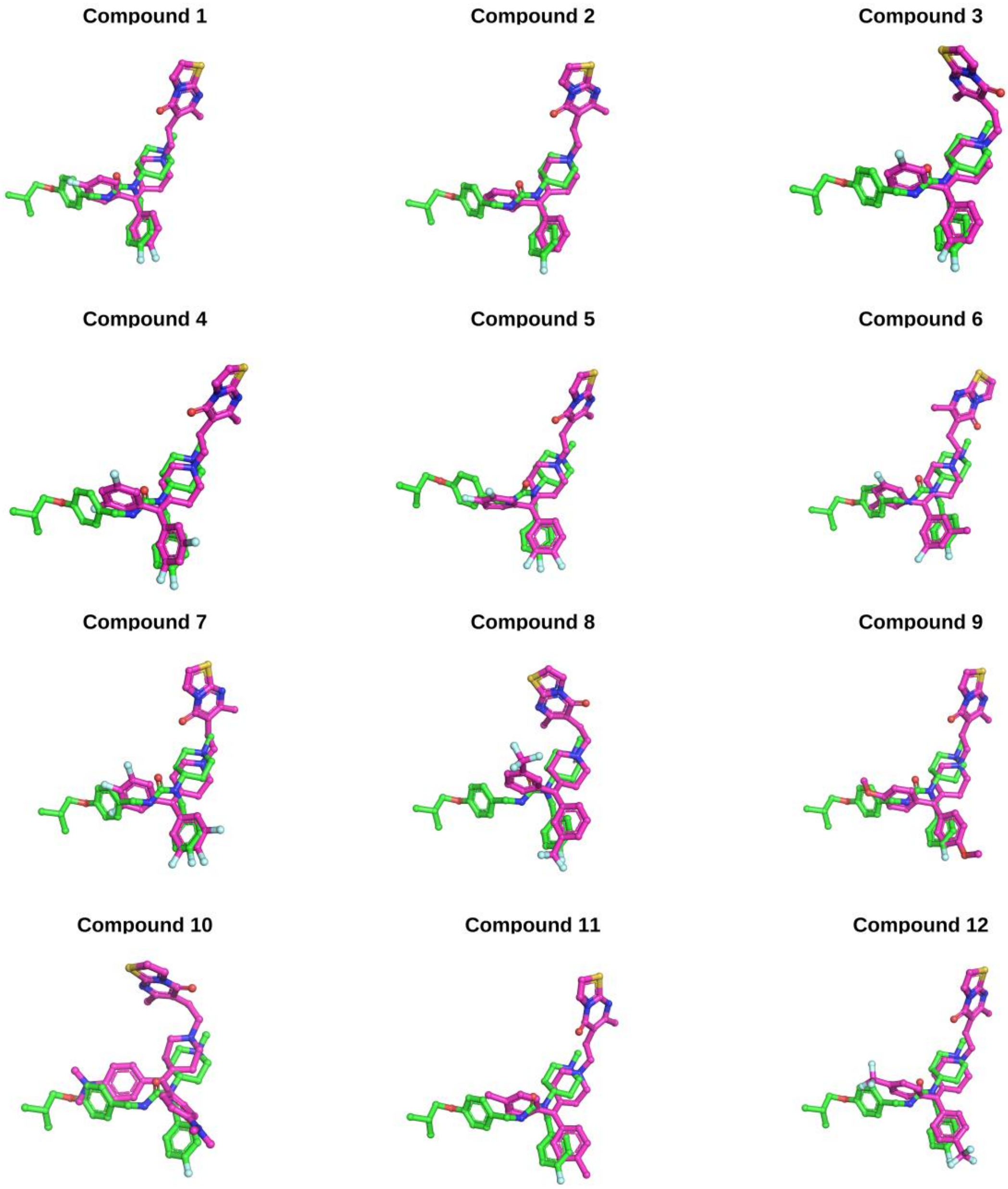

Figure S23. Structural validation against the pimavanserin-bound 5-HT$_{2A}$ receptor. Superposition of the final MD simulation frame (green sticks) with the experimental crystal structure of the 5-HT$_{2A}$ receptor co-crystallized with pimavanserin (magenta sticks, PDB ID: 8ZMG).

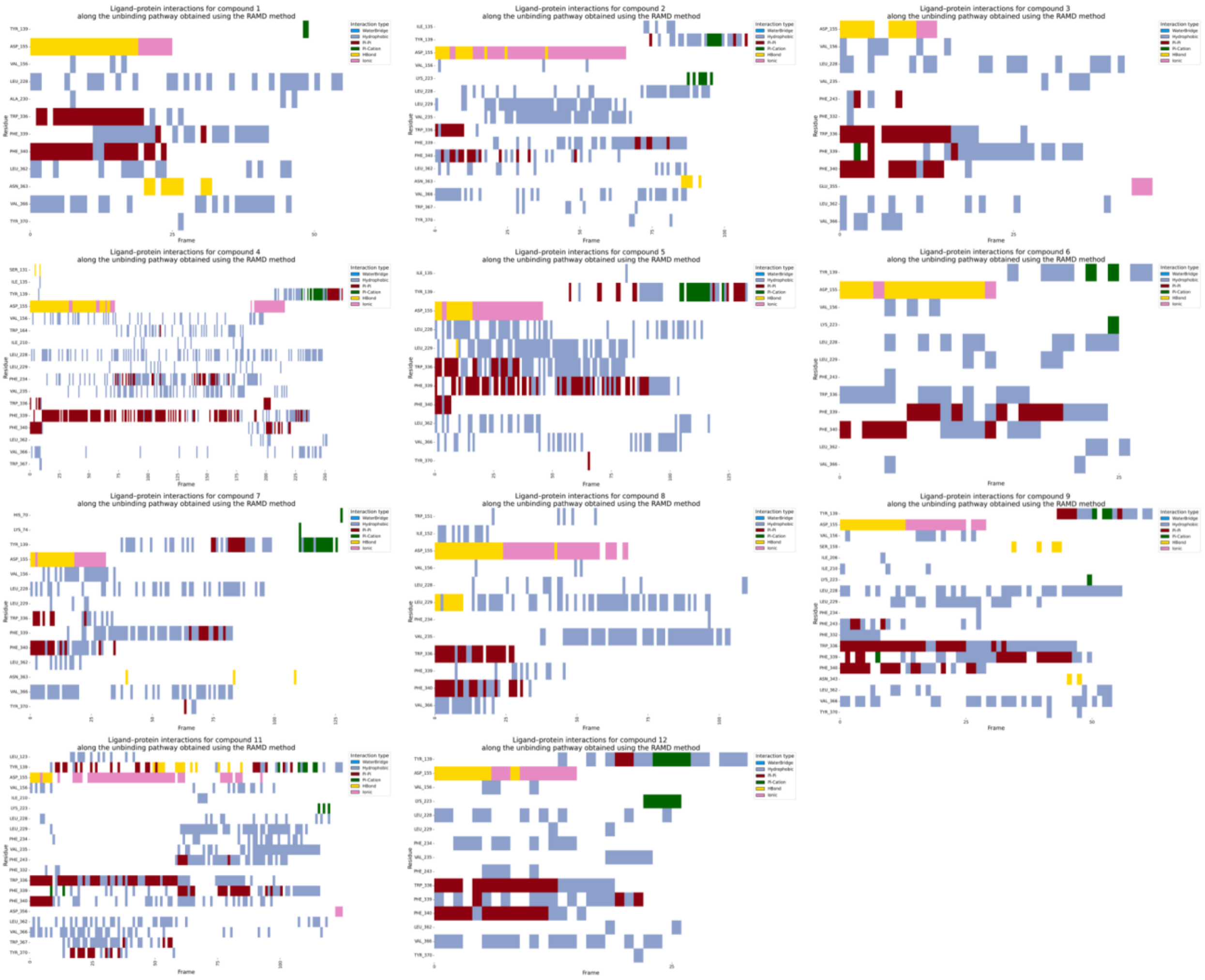

Figure S24. Interactions between individual amino acid residues and the ligand as identified through RAMD unbinding simulations. Different colors denote distinct types of interactions: water bridge – blue; hydrophobic – blue-gray; π–π – ruby; π–cation – green; hydrogen bond – yellow; ionic – magenta.

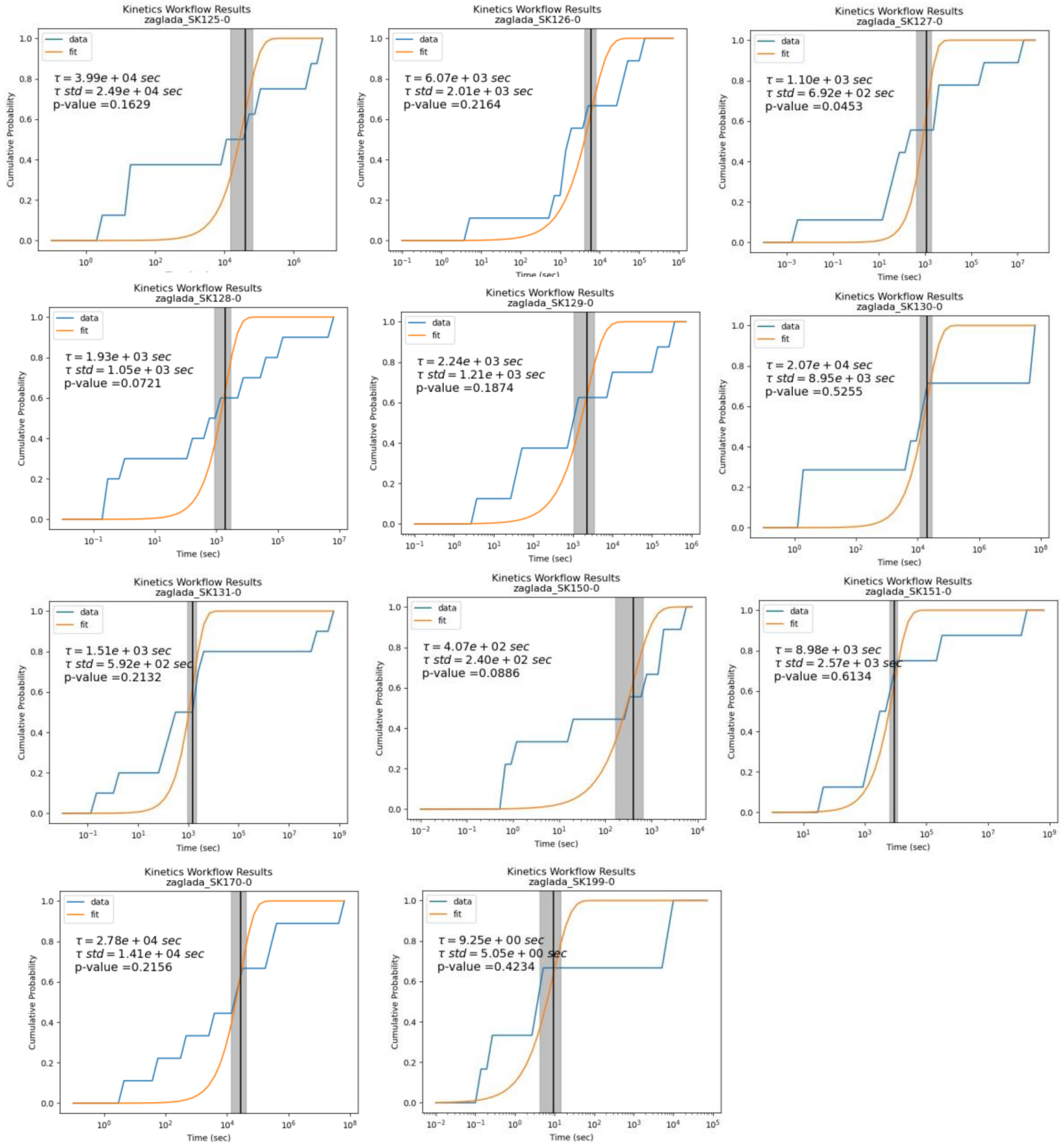

Figure S25. Computational estimation of ligand residence times. Quantitative analysis of dissociation kinetics for the investigated compounds. The data illustrates the cumulative distribution of simulated unbinding events used to extrapolate absolute residence time (RT) values, as generated by the automated analysis protocol within the Schrödinger suite.

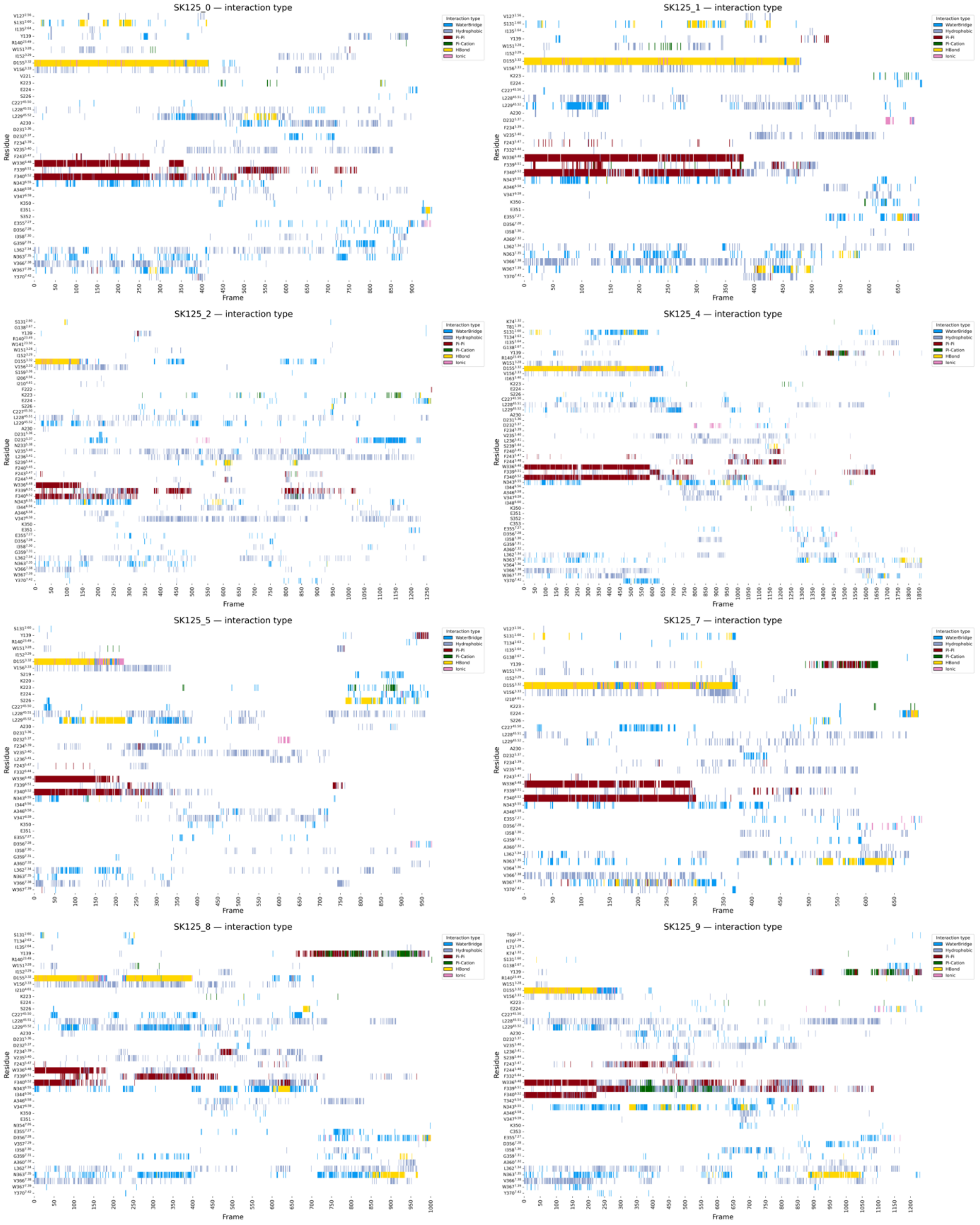

Figure S26. Interactions between individual amino acid residues and the ligand as identified through iMetaD simulation (**compound 1**). Different colors denote distinct types of interactions: water bridge – blue; hydrophobic – blue-gray; π–π – ruby; π–cation – green; hydrogen bond – yellow; ionic – magenta.

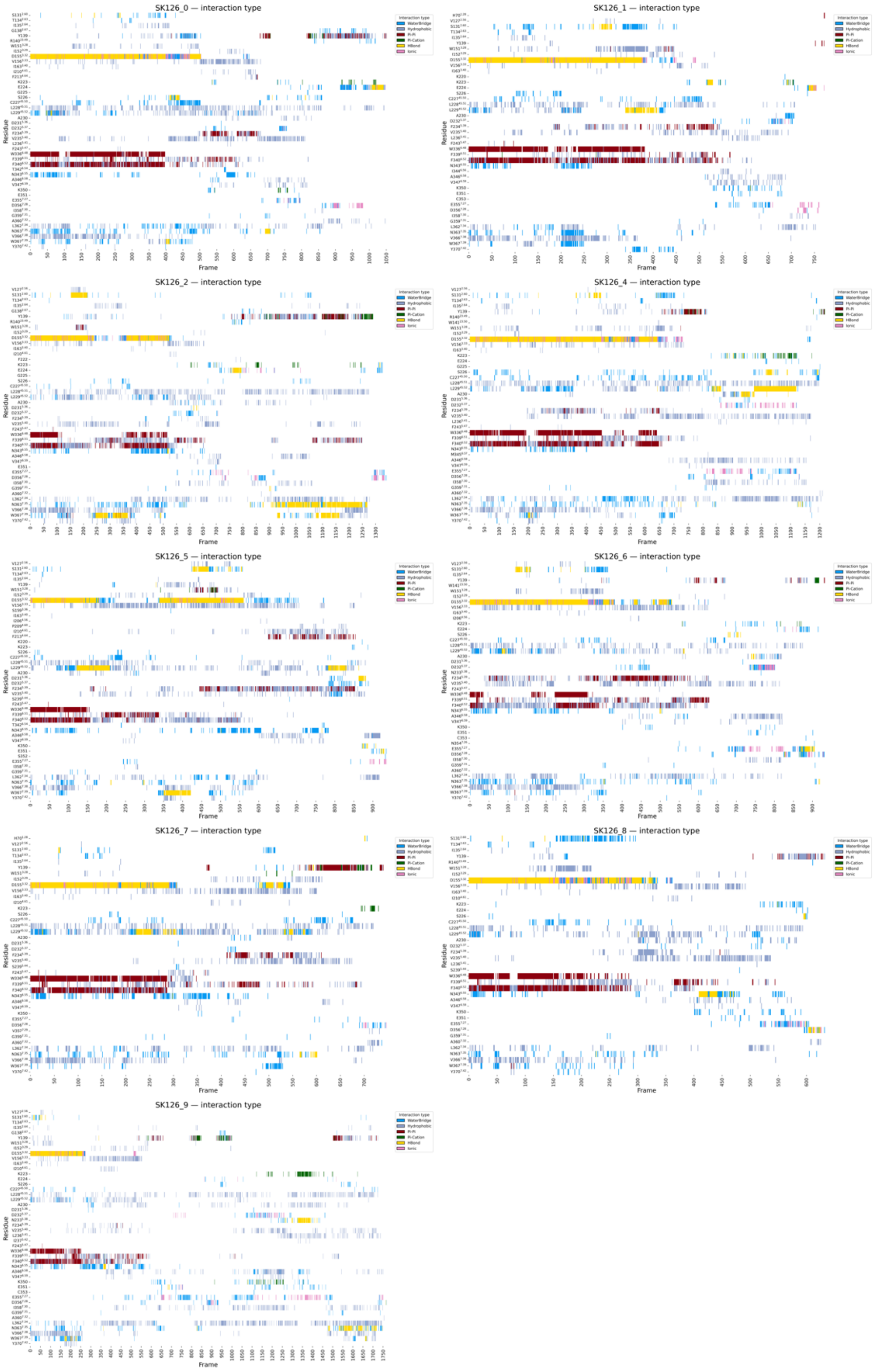

Figure S27. Interactions between individual amino acid residues and the ligand as identified through iMetaD simulation (**compound 2**). Different colors denote distinct types of interactions: water bridge – blue; hydrophobic – blue-gray; π–π – ruby; π–cation – green; hydrogen bond – yellow; ionic – magenta.

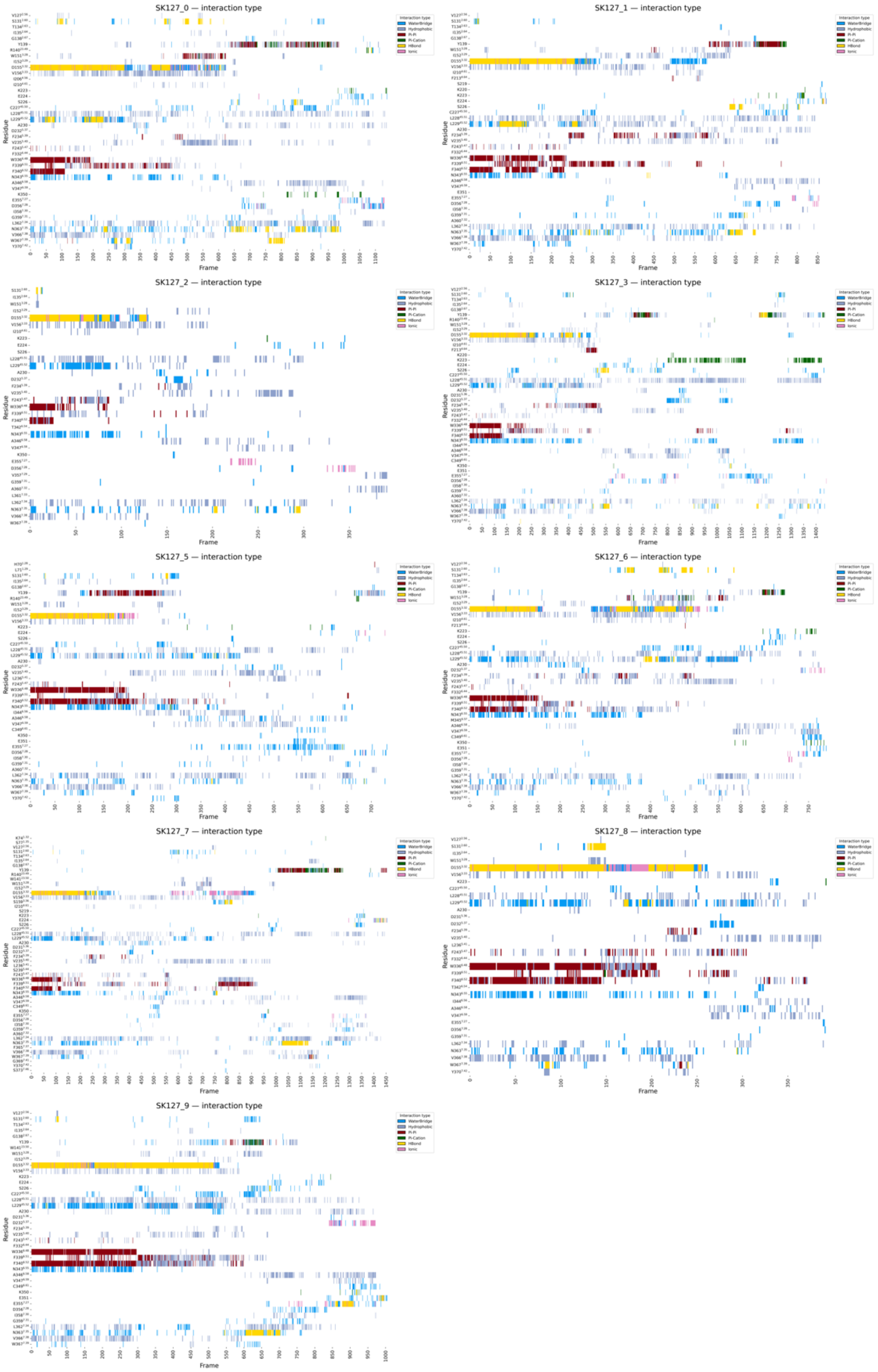

Figure S28. Interactions between individual amino acid residues and the ligand as identified through iMetaD simulation (**compound 3**). Different colors denote distinct types of interactions: water bridge – blue; hydrophobic – blue-gray; π–π – ruby; π–cation – green; hydrogen bond – yellow; ionic – magenta.

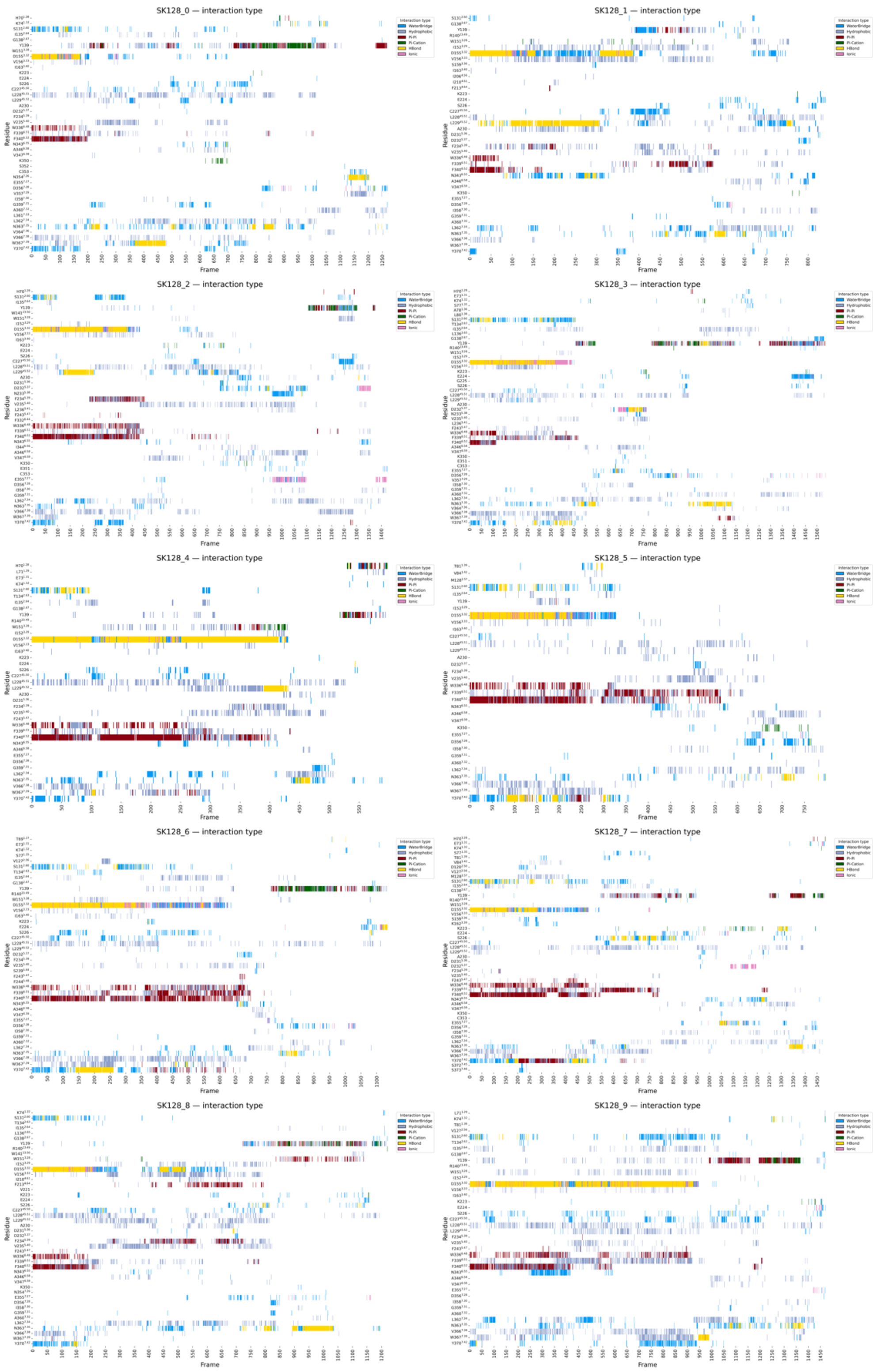

Figure S29. Interactions between individual amino acid residues and the ligand as identified through iMetaD simulation (**compound 4**). Different colors denote distinct types of interactions: water bridge – blue; hydrophobic – blue-gray; π–π – ruby; π–cation – green; hydrogen bond – yellow; ionic – magenta.

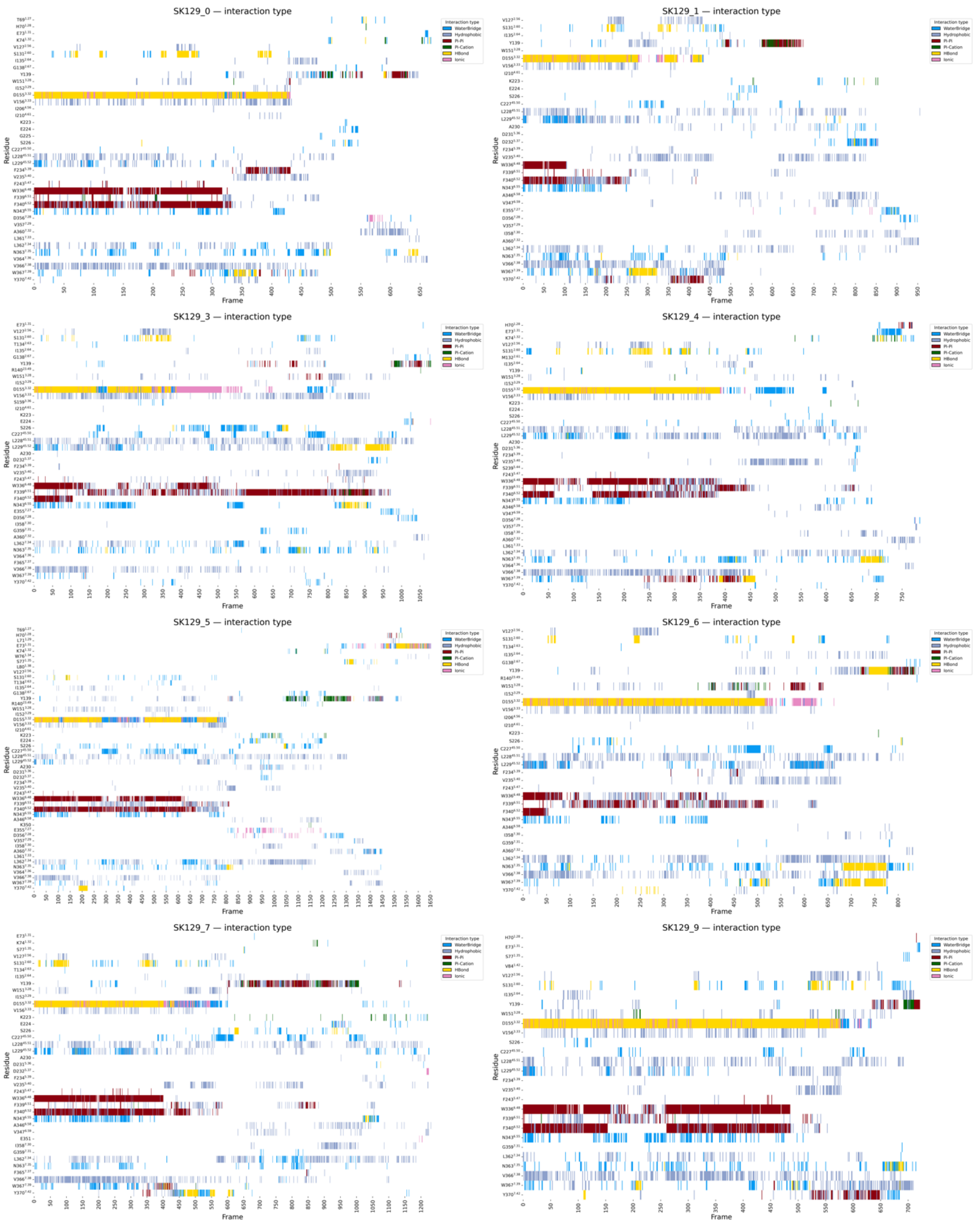

Figure S30. Interactions between individual amino acid residues and the ligand as identified through iMetaD simulation (**compound 5**). Different colors denote distinct types of interactions: water bridge – blue; hydrophobic – blue-gray; π–π – ruby; π–cation – green; hydrogen bond – yellow; ionic – magenta.

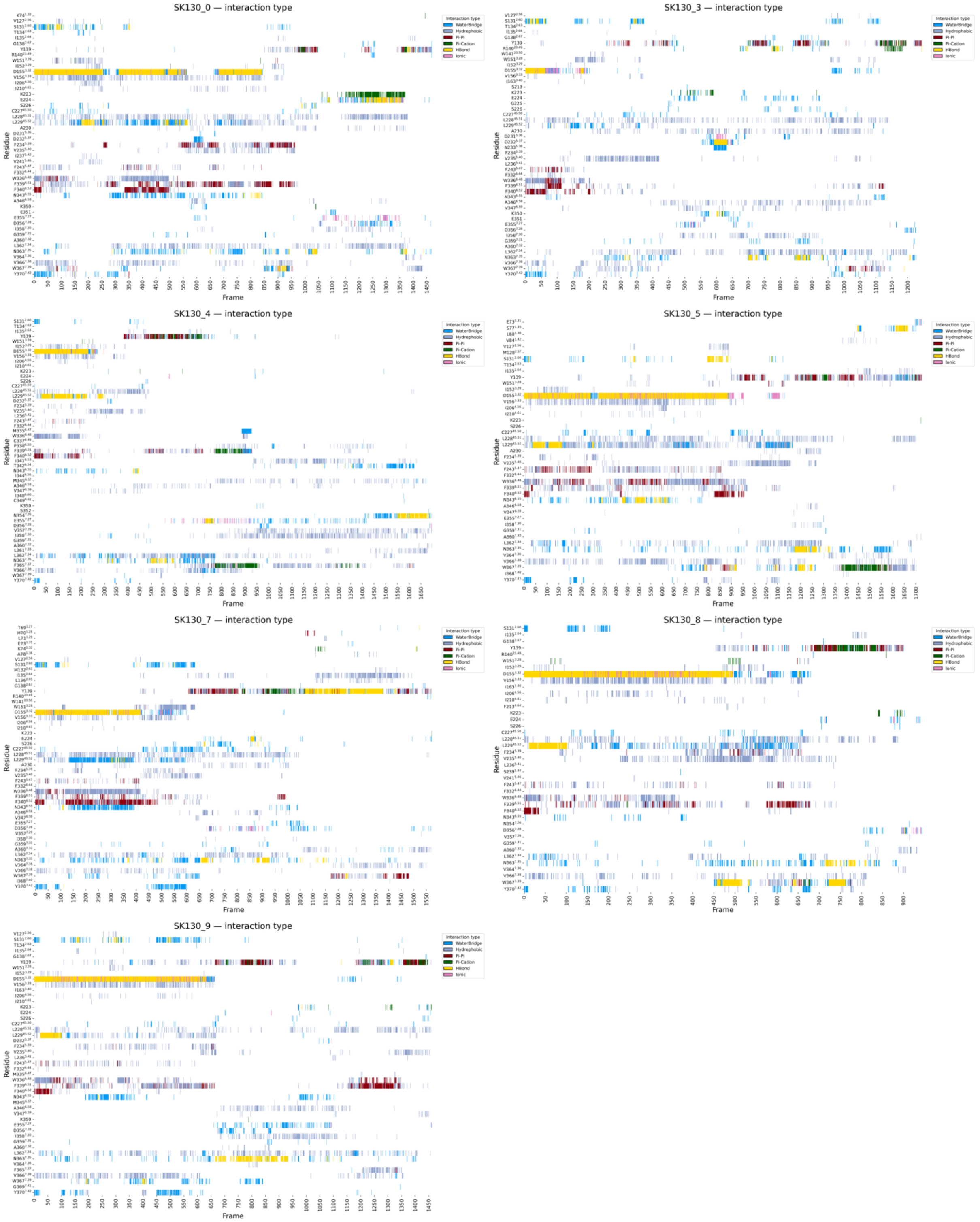

Figure S31. Interactions between individual amino acid residues and the ligand as identified through iMetaD simulation (**compound 6**). Different colors denote distinct types of interactions: water bridge – blue; hydrophobic – blue-gray; π–π – ruby; π–cation – green; hydrogen bond – yellow; ionic – magenta.

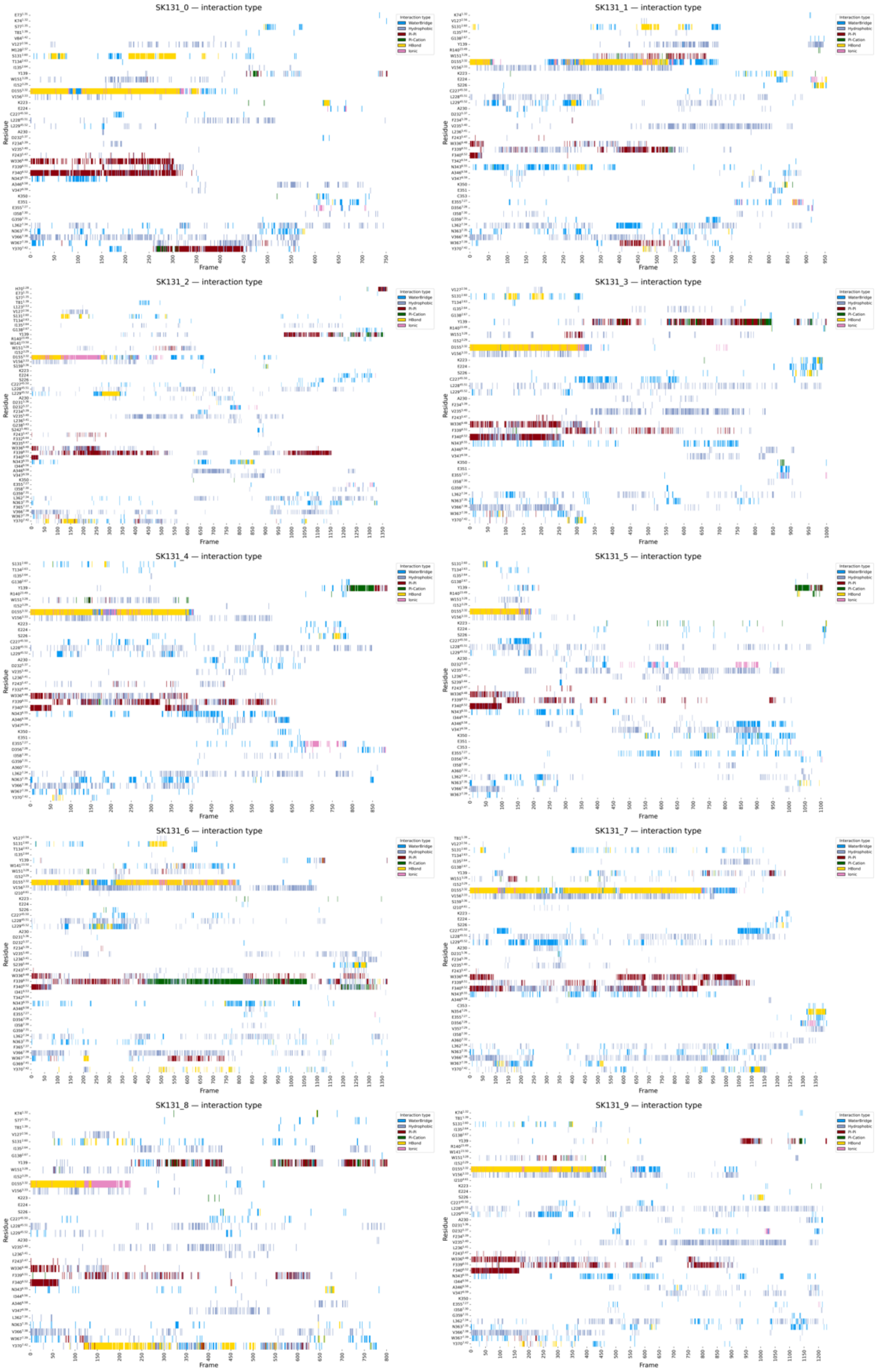

Figure S32. Interactions between individual amino acid residues and the ligand as identified through iMetaD simulation (**compound 7**). Different colors denote distinct types of interactions: water bridge – blue; hydrophobic – blue-gray; π–π – ruby; π–cation – green; hydrogen bond – yellow; ionic – magenta.

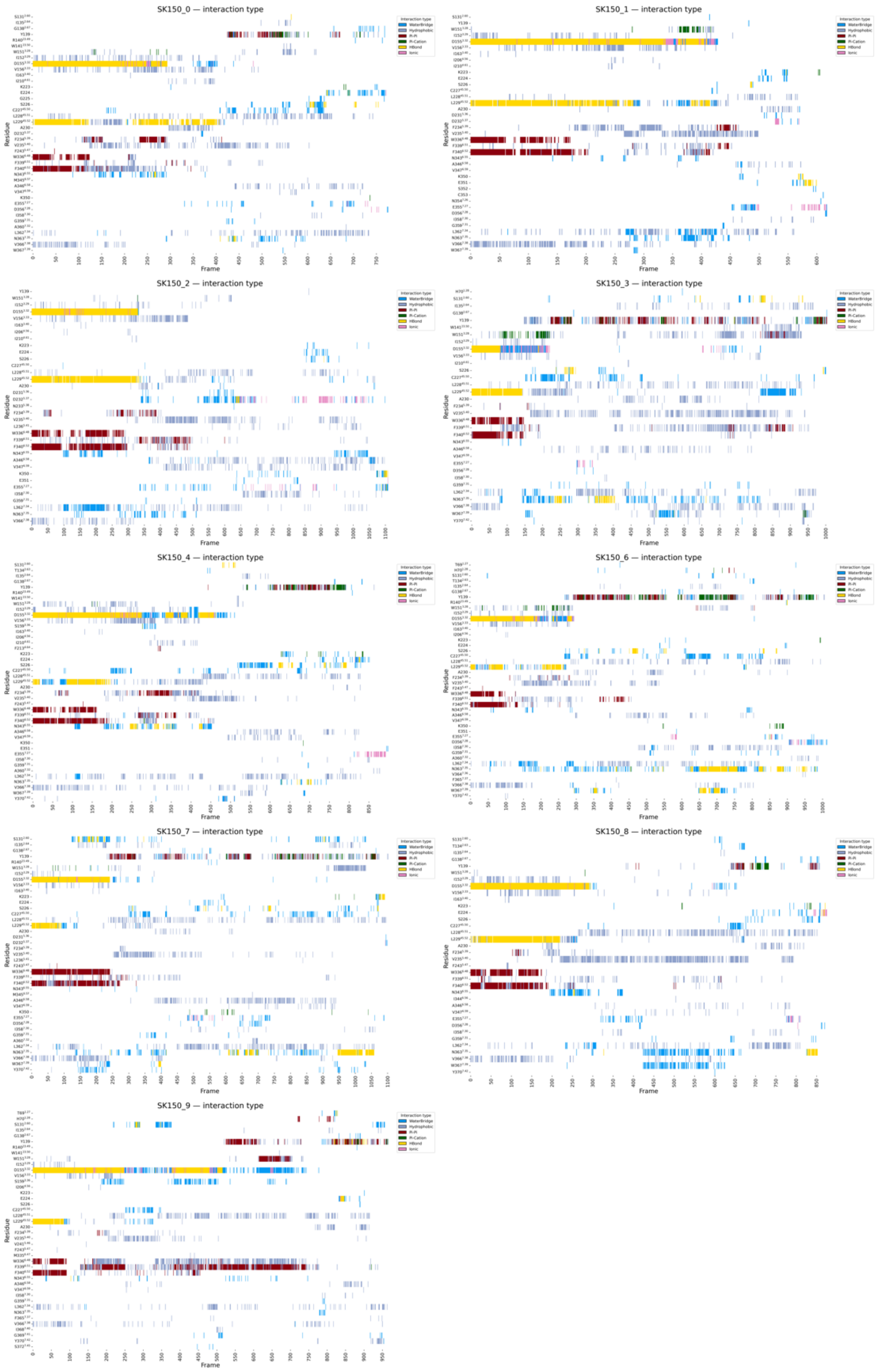

Figure S33. Interactions between individual amino acid residues and the ligand as identified through iMetaD simulation (**compound 8**). Different colors denote distinct types of interactions: water bridge – blue; hydrophobic – blue-gray; π–π – ruby; π–cation – green; hydrogen bond – yellow; ionic – magenta.

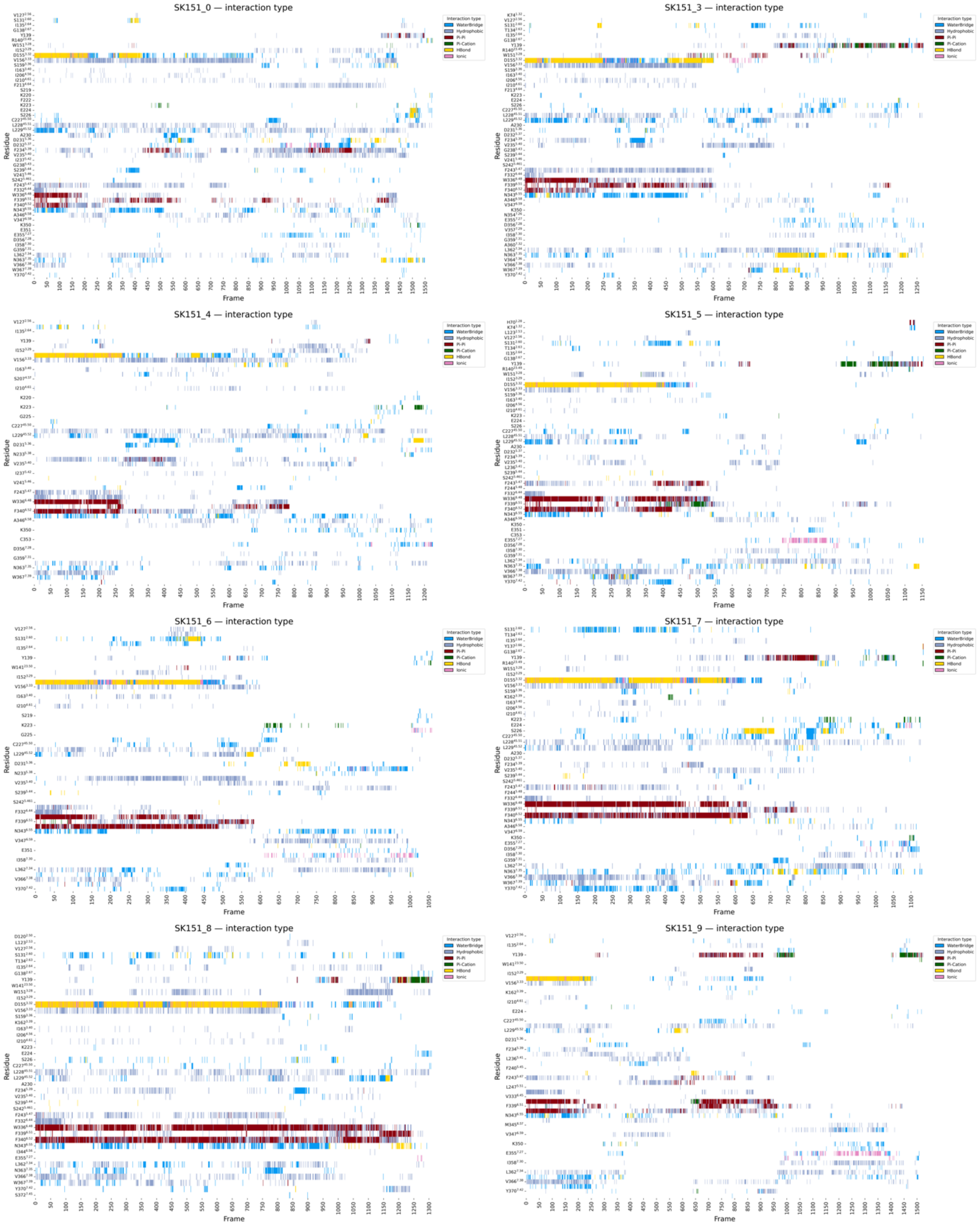

Figure S34. Interactions between individual amino acid residues and the ligand as identified through iMetaD simulation (**compound 9**). Different colors denote distinct types of interactions: water bridge – blue; hydrophobic – blue-gray; π–π – ruby; π–cation – green; hydrogen bond – yellow; ionic – magenta.

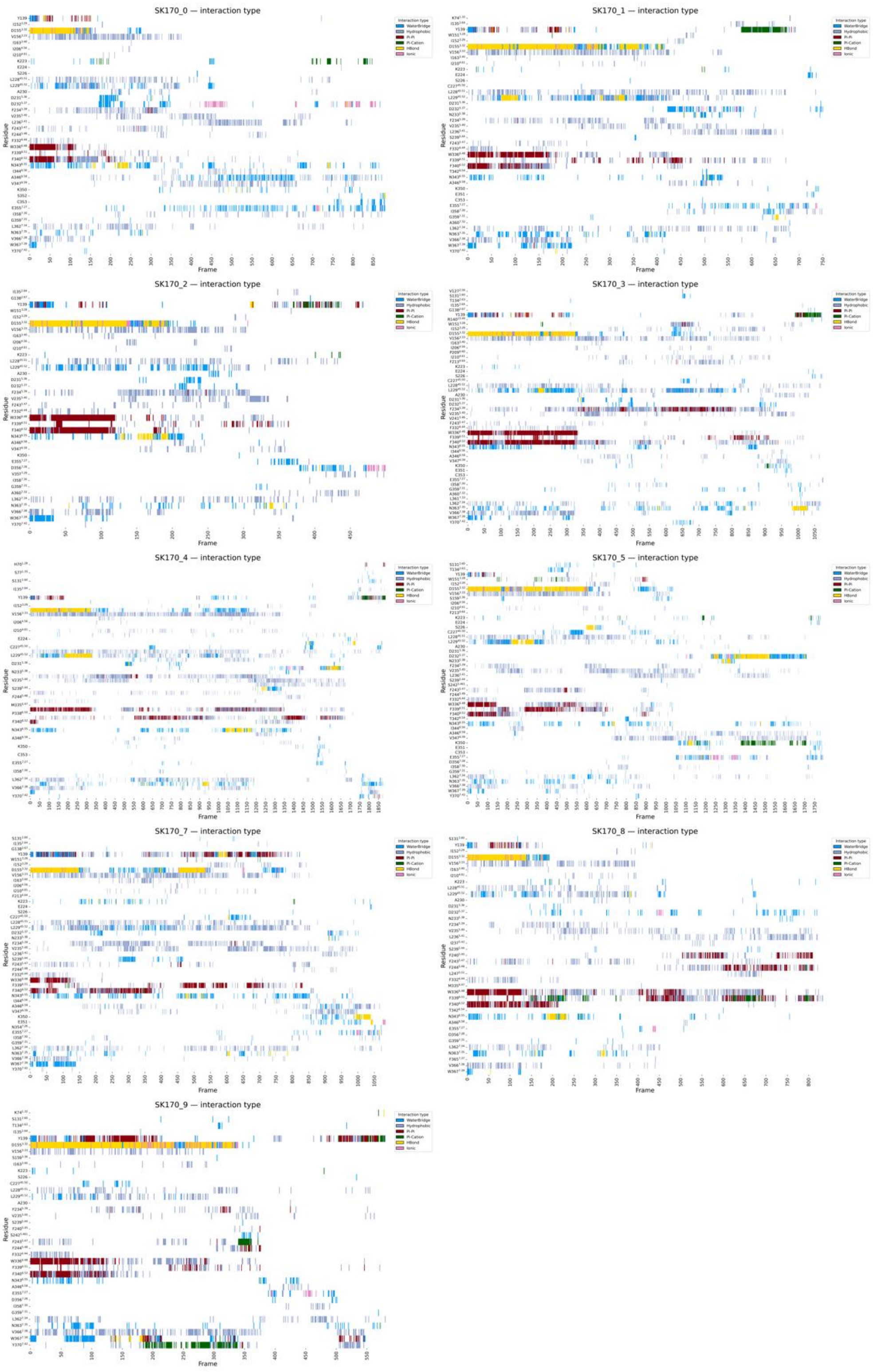

Figure S35. Interactions between individual amino acid residues and the ligand as identified through iMetaD simulation (**compound 11**). Different colors denote distinct types of interactions: water bridge – blue; hydrophobic – blue-gray; π–π – ruby; π–cation – green; hydrogen bond – yellow; ionic – magenta.

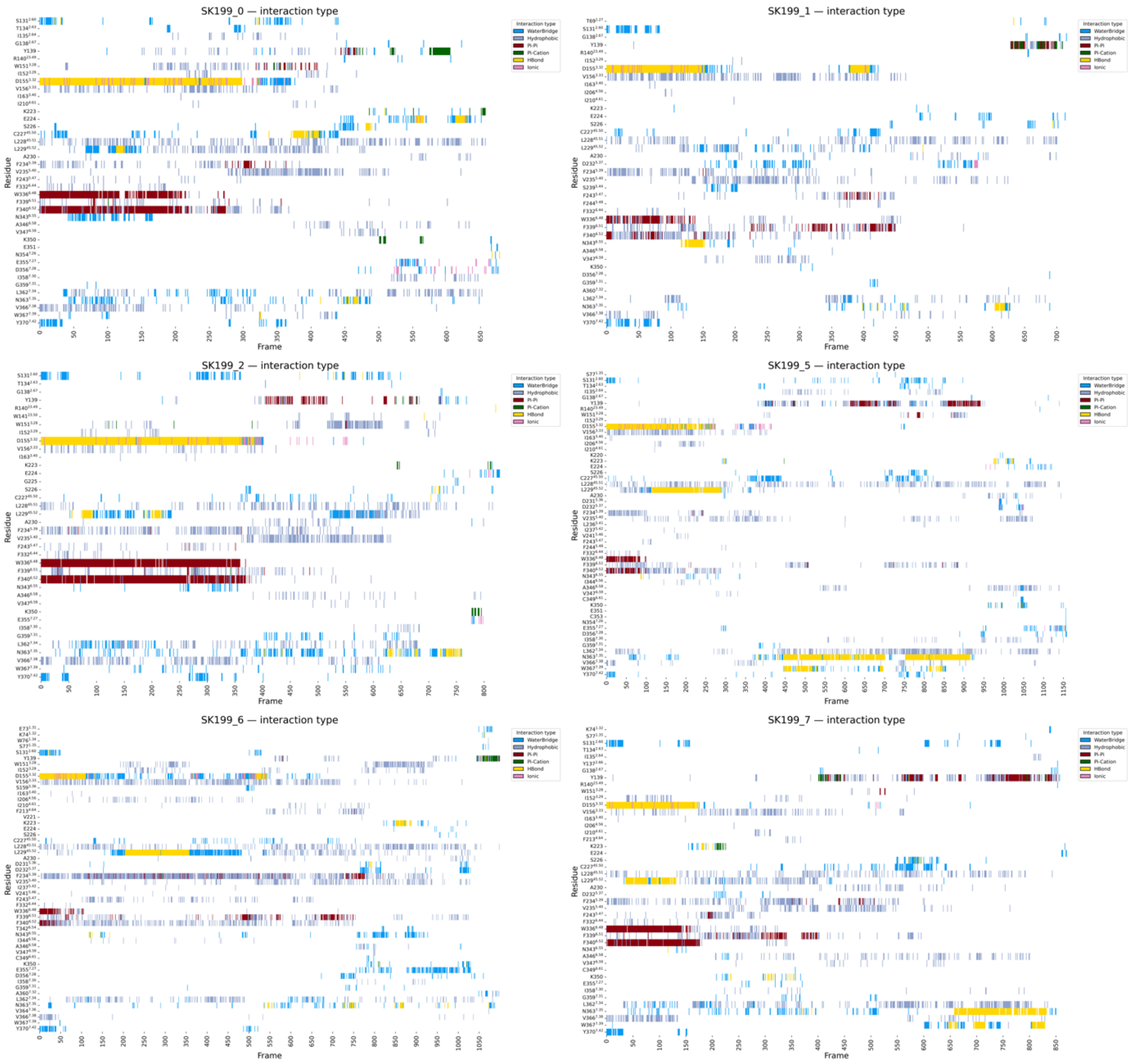

Figure S36. Interactions between individual amino acid residues and the ligand as identified through iMetaD simulation (**compound 12**). Different colors denote distinct types of interactions: water bridge – blue; hydrophobic – blue-gray; π–π – ruby; π–cation – green; hydrogen bond – yellow; ionic – magenta.

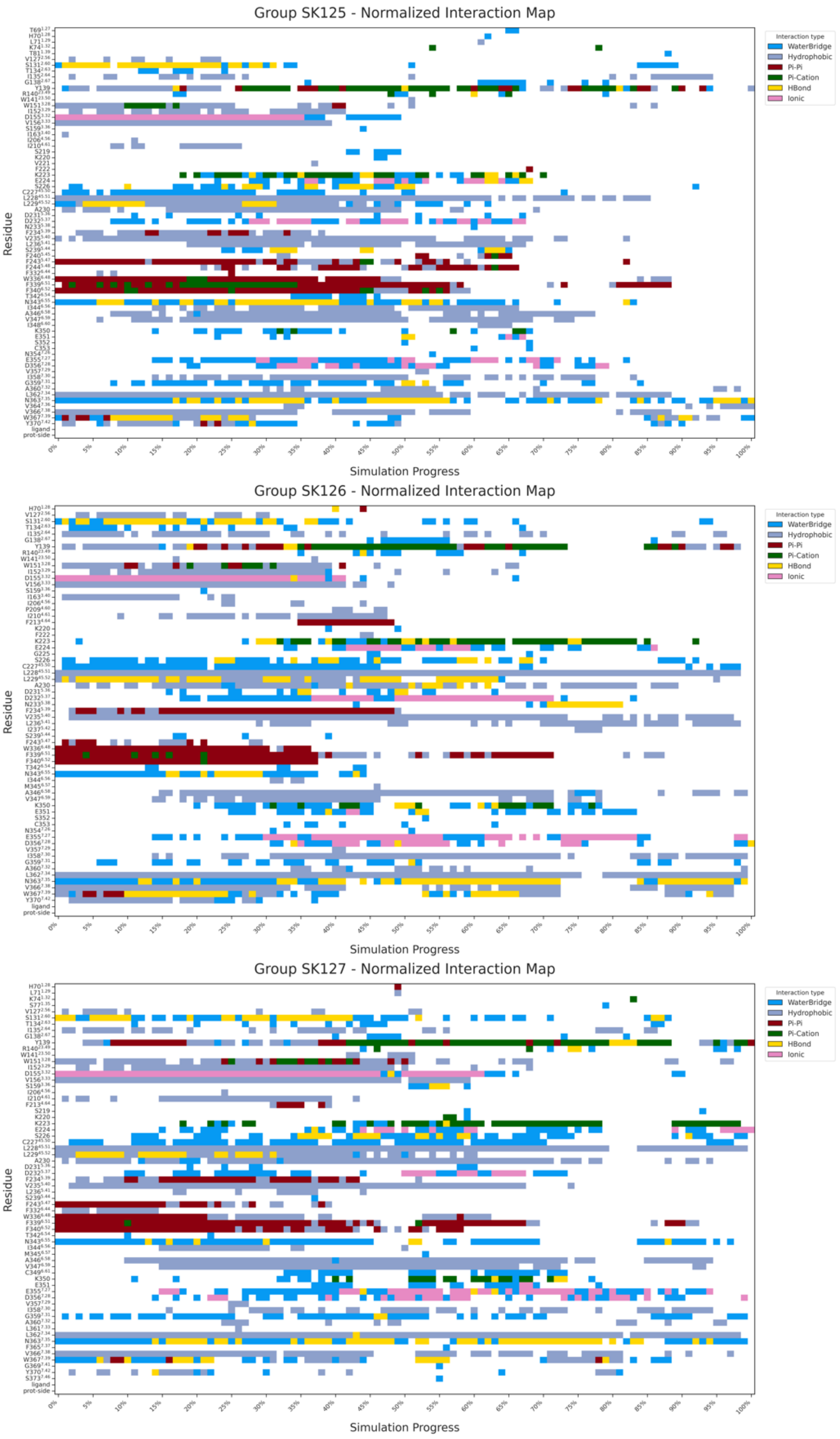

Figure S37. Normalized interaction map between individual amino acid residues and the ligand as identified through iMetaD simulation (**compounds 1-3**). Different colors denote distinct types of interactions: water bridge – blue; hydrophobic – blue-gray; π–π – ruby; π–cation – green; hydrogen bond – yellow; ionic – magenta.

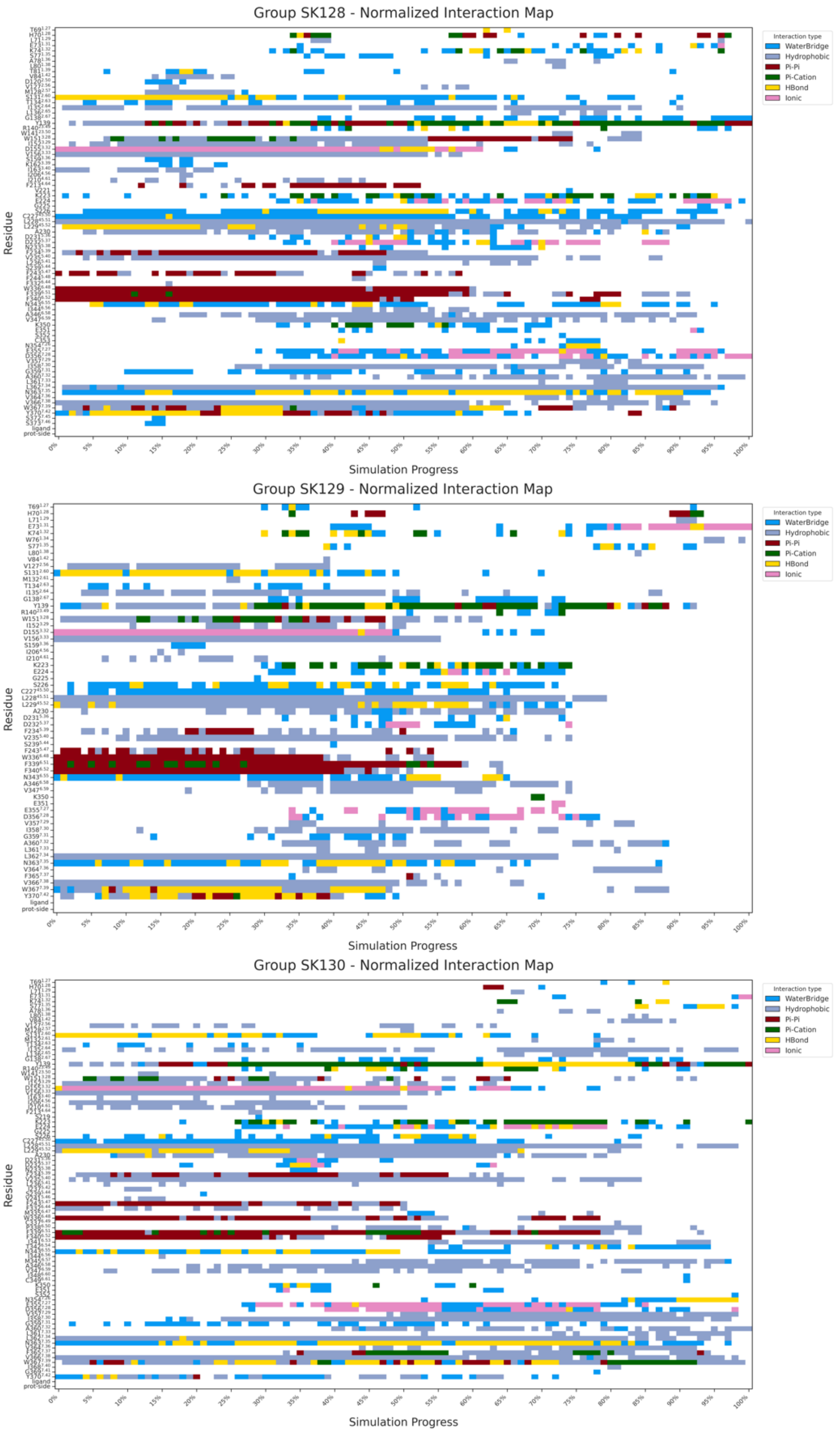

Figure S38. Normalized interaction map between individual amino acid residues and the ligand as identified through iMetaD simulation (**compounds 4-6**). Different colors denote distinct types of interactions: water bridge – blue; hydrophobic – blue-gray; π–π – ruby; π–cation – green; hydrogen bond – yellow; ionic – magenta.

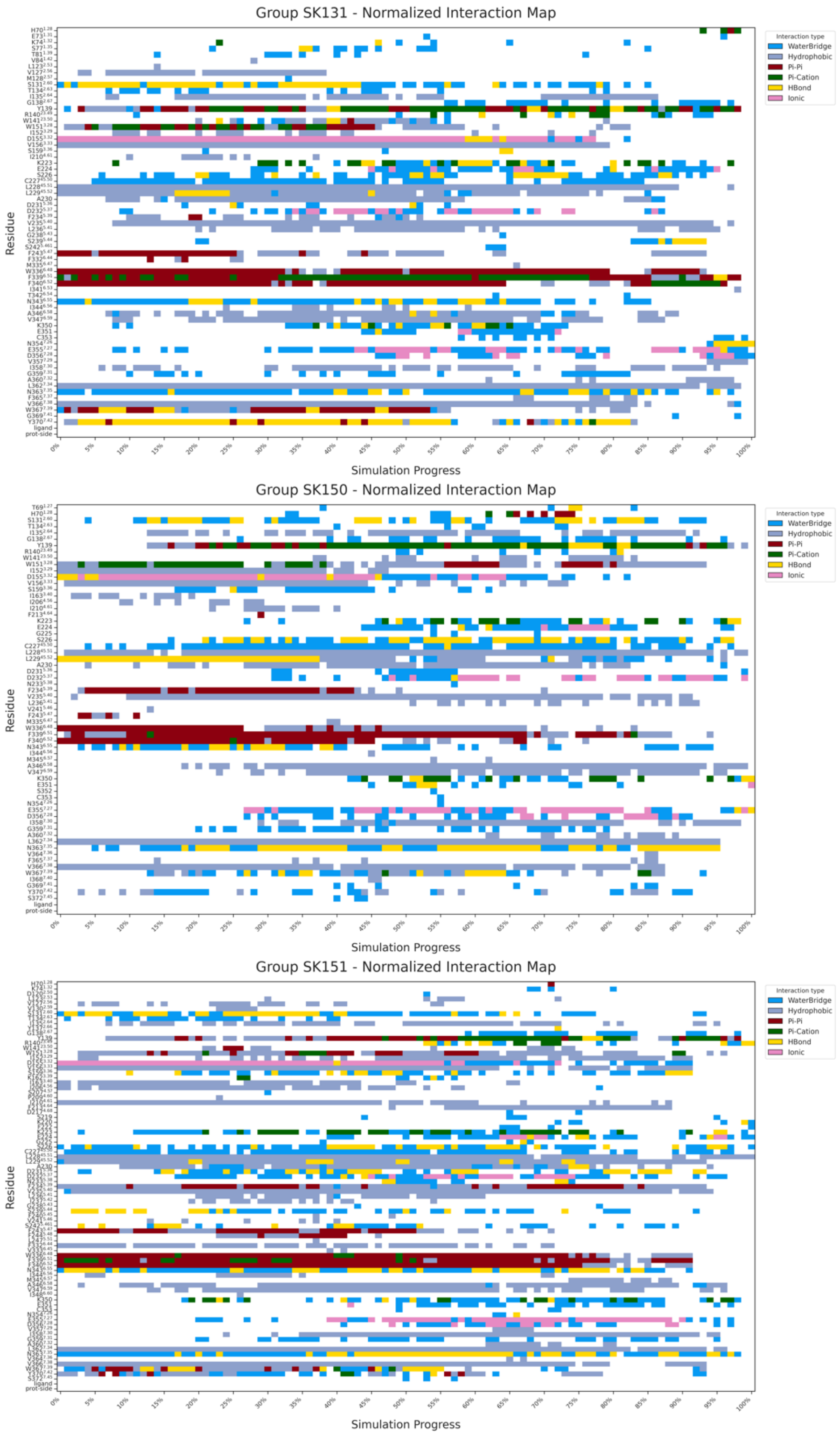

Figure S39. Normalized interaction map between individual amino acid residues and the ligand as identified through iMetaD simulation (**compounds 7-9**). Different colors denote distinct types of interactions: water bridge – blue; hydrophobic – blue-gray; π–π – ruby; π–cation – green; hydrogen bond – yellow; ionic – magenta.

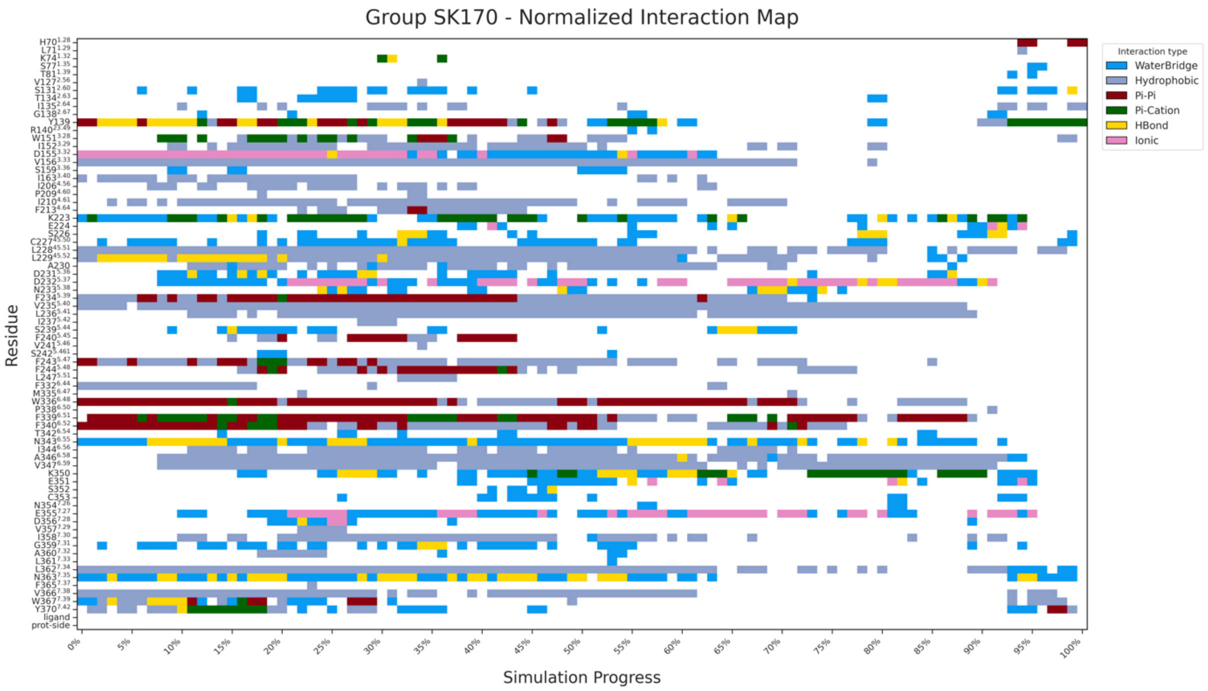

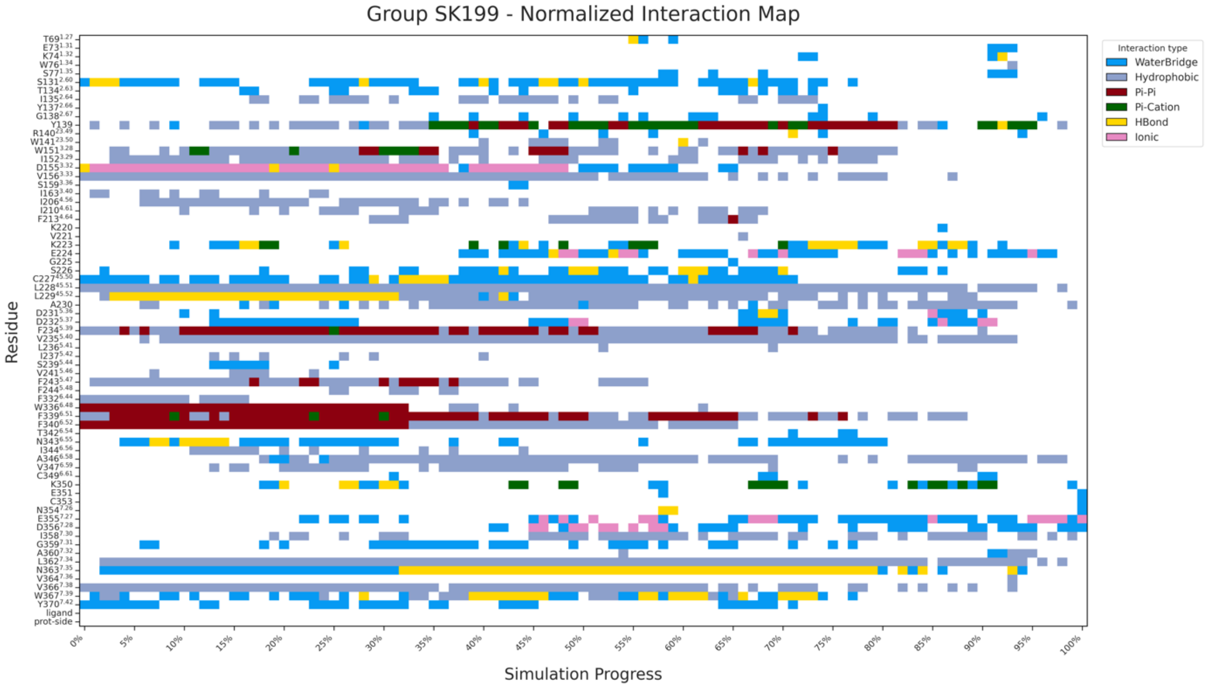

Figure S40. Normalized interaction map between individual amino acid residues and the ligand as identified through iMetaD simulation (**compounds 11-12**). Different colors denote distinct types of interactions: water bridge – blue; hydrophobic – blue-gray; π–π – ruby; π–cation – green; hydrogen bond – yellow; ionic – magenta.

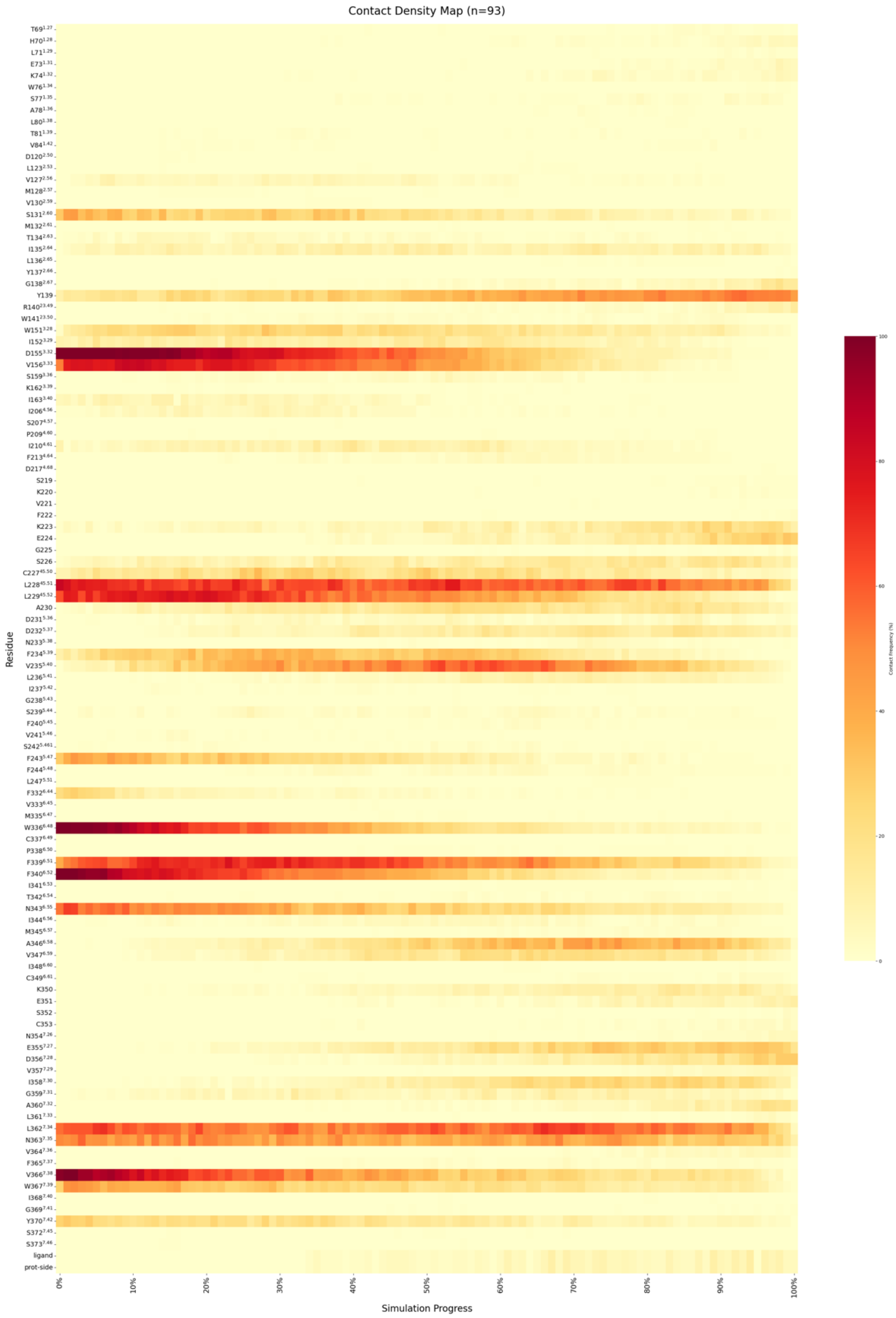

Figure S41. Global interaction density map. Spatiotemporal mapping of ligand–residue contacts derived from iMetaD simulations. The heatmap illustrates the frequency and persistence of individual non-covalent interactions throughout the normalized simulation time (0–100%).

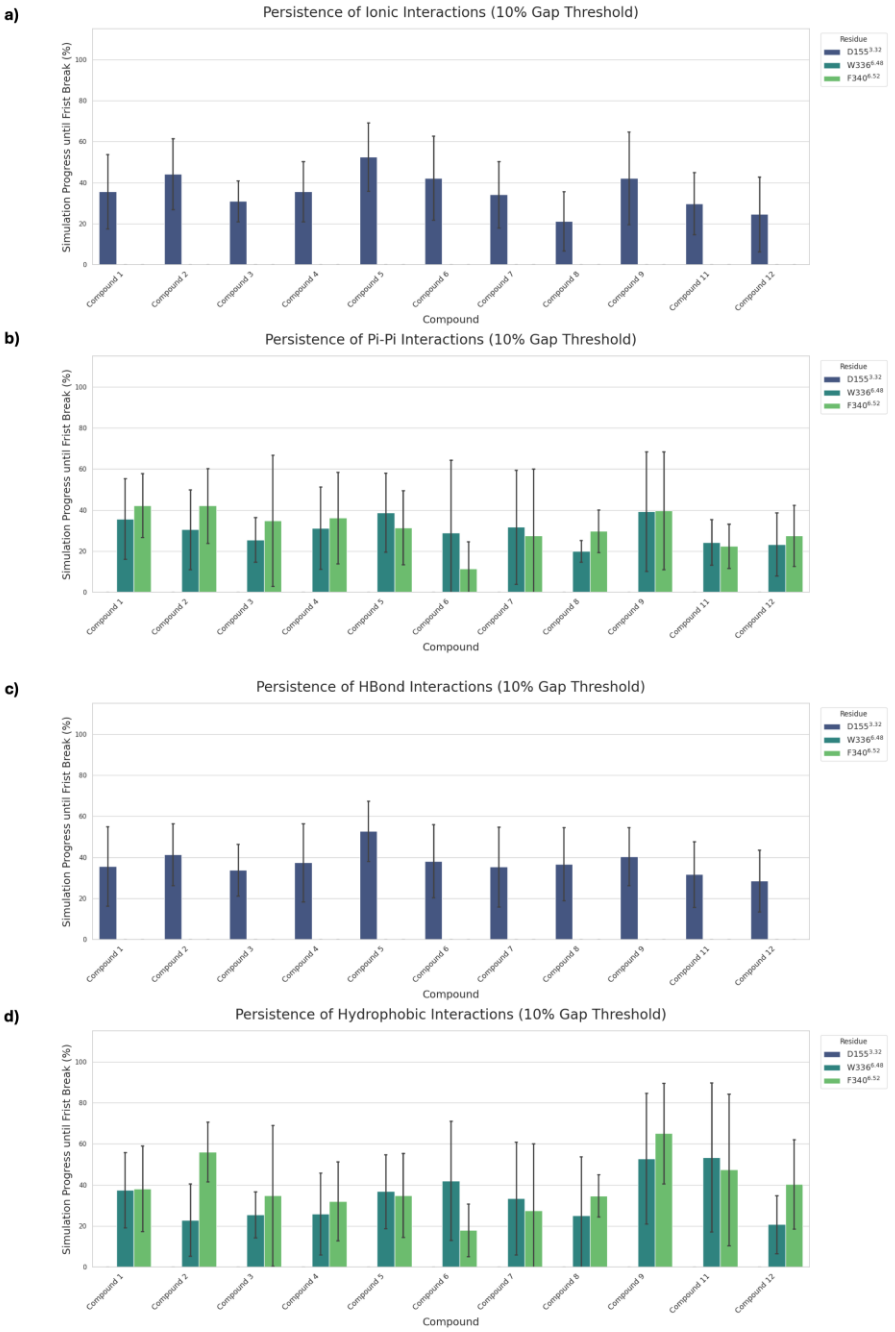

Figure S42. Kinetic profiling of specific interaction ruptures during ligand egress. The charts quantify the mean temporal point of dissociation for individual ligand-receptor contacts, expressed as a percentage of the normalized simulation time. The rupture point is strictly defined as the threshold beyond which a given interaction remains undetected for a contiguous 10% of the trajectory. Panels denote the dissociation profiles for distinct non-covalent modalities: (a) ionic interactions, (b) π–π stacking, (c) hydrogen bonds, and (d) hydrophobic contacts

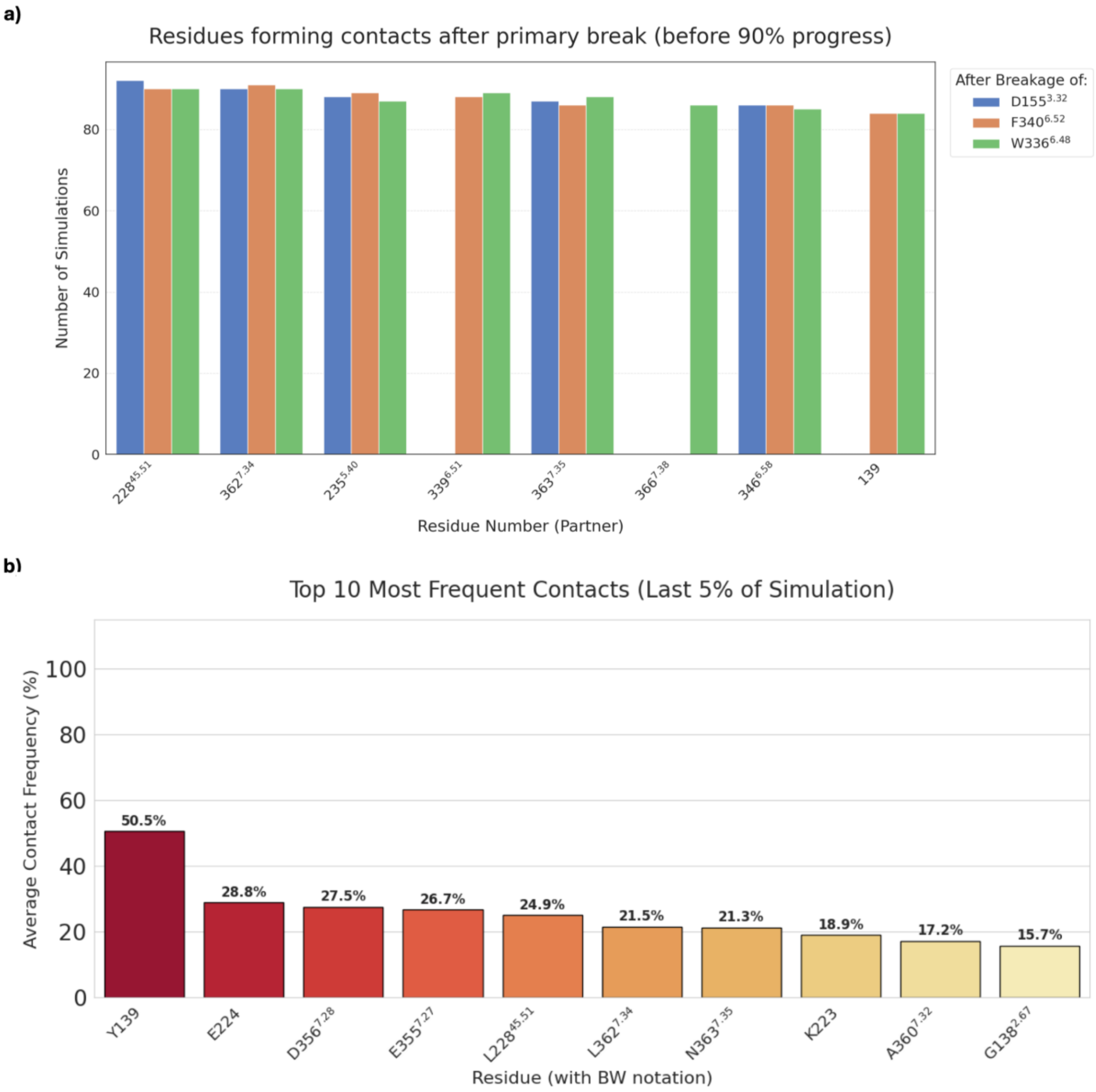

Figure S43. Characterization of the intermediate and terminal phases of ligand egress. (a) Frequencies of transient interactions formed with secondary receptor residues following the targeted rupture of primary orthosteric anchors. (b) Dominant terminal contacts observed during the final 5% of the unbinding trajectories, immediately preceding complete ligand dissociation.

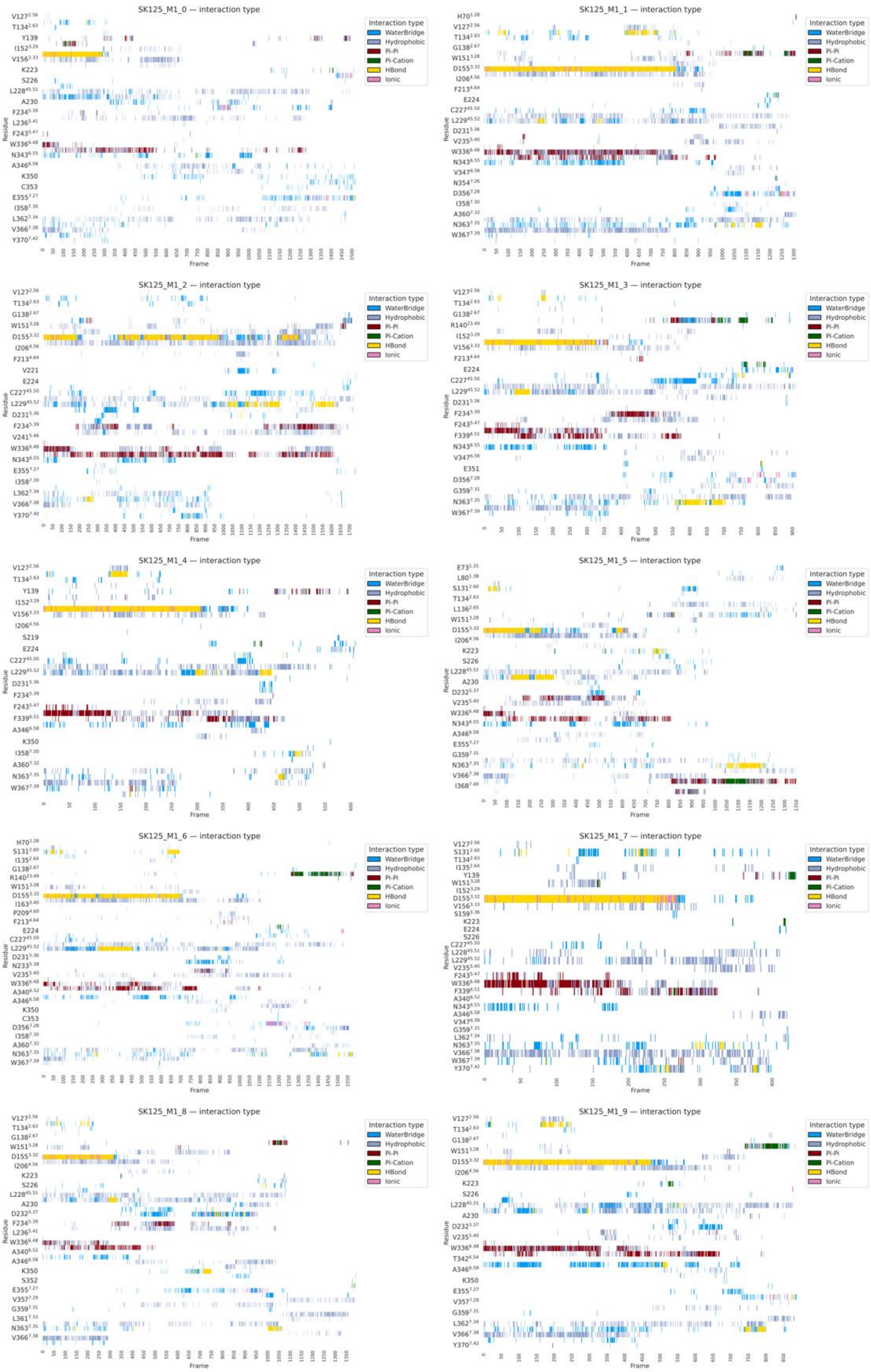

Figure S44. Interactions between individual amino acid residues and the ligand as identified through iMetaD simulation (**compound 1 mutation F340$^{6.52}$A**). Different colors denote distinct types of interactions: water bridge – blue; hydrophobic – blue-gray; π–π – ruby; π–cation – green; hydrogen bond – yellow; ionic – magenta.

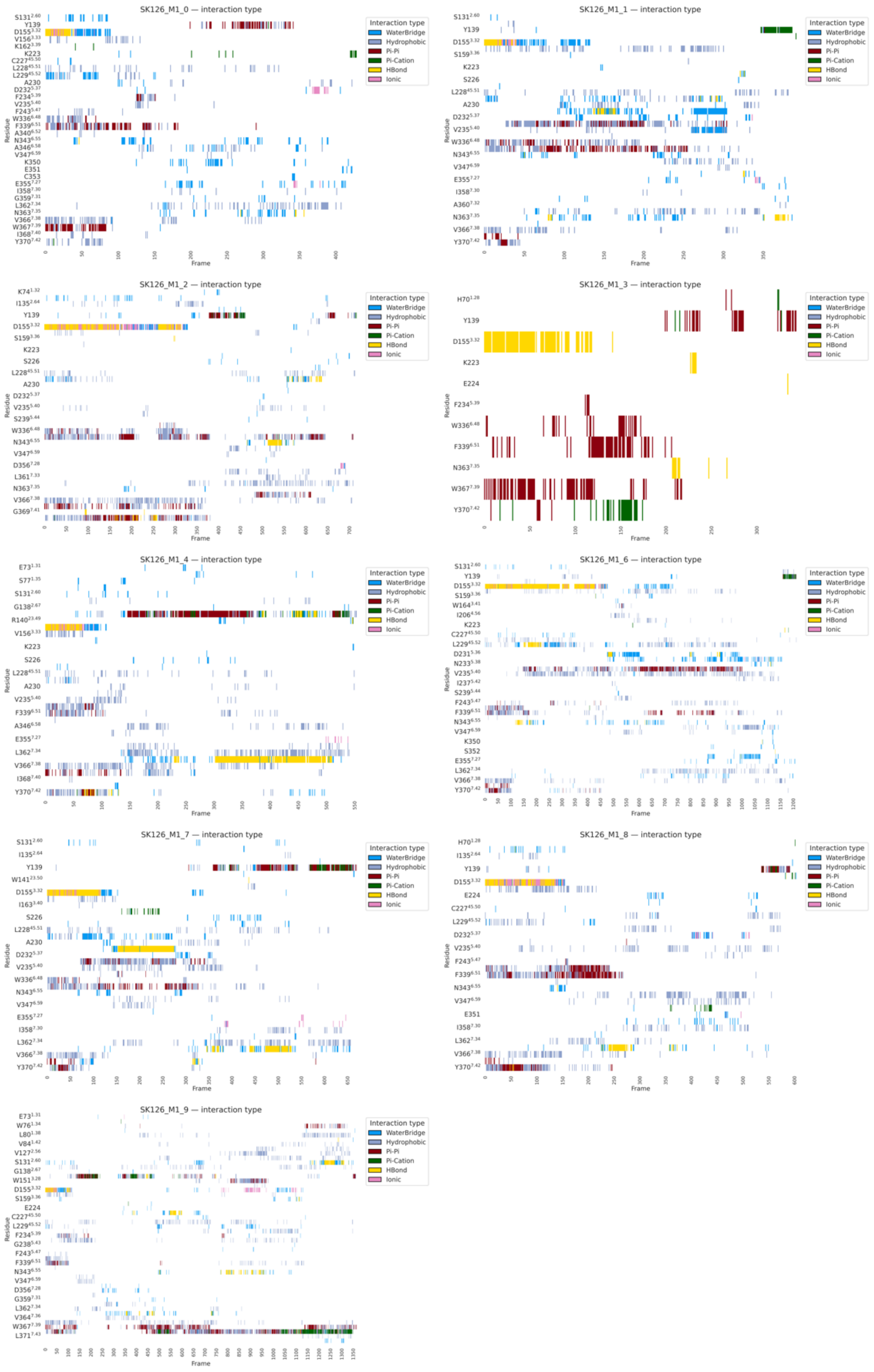

Figure S45. Interactions between individual amino acid residues and the ligand as identified through iMetaD simulation (**compound 2 mutation F340$^{6.52}$A**). Different colors denote distinct types of interactions: water bridge – blue; hydrophobic – blue-gray; π–π – ruby; π–cation – green; hydrogen bond – yellow; ionic – magenta.

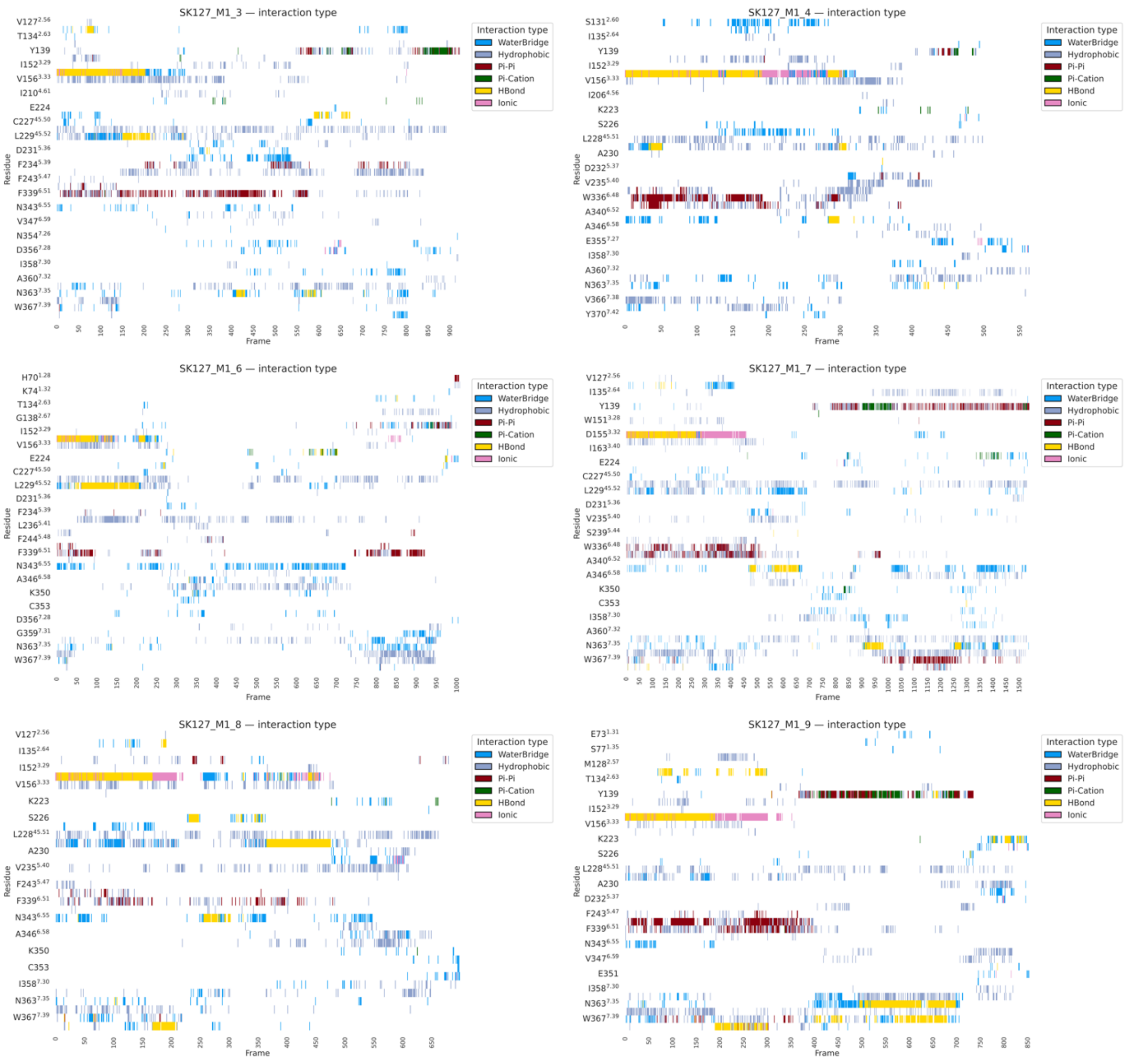

Figure S46. Interactions between individual amino acid residues and the ligand as identified through iMetaD simulation (**compound 3 mutation F340$^{6.52}$A**). Different colors denote distinct types of interactions: water bridge – blue; hydrophobic – blue-gray; π–π – ruby; π–cation – green; hydrogen bond – yellow; ionic – magenta.

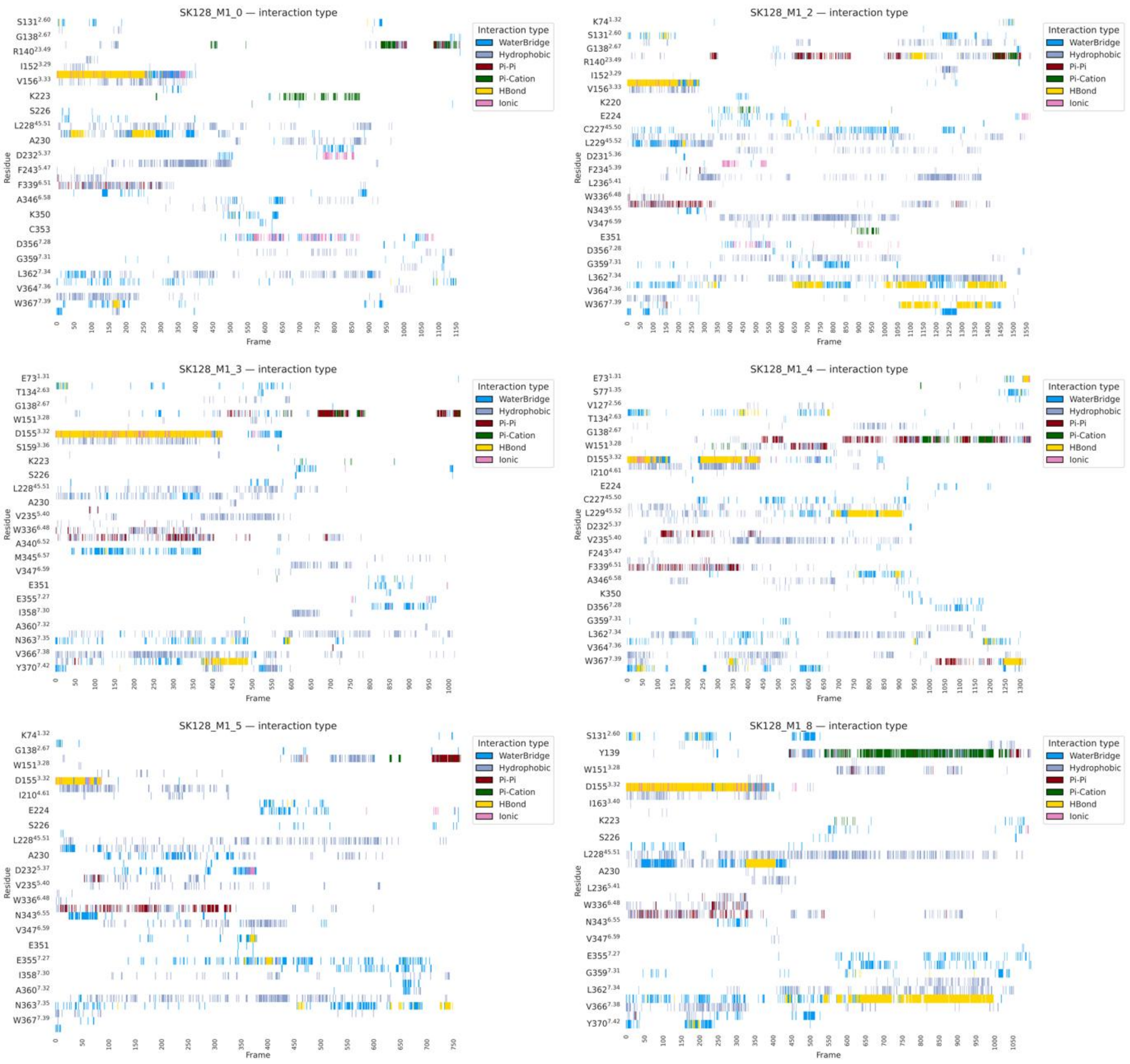

Figure S47. Interactions between individual amino acid residues and the ligand as identified through iMetaD simulation (**compound 4 mutation F340$^{6.52}$A**). Different colors denote distinct types of interactions: water bridge – blue; hydrophobic – blue-gray; π–π – ruby; π–cation – green; hydrogen bond – yellow; ionic – magenta.

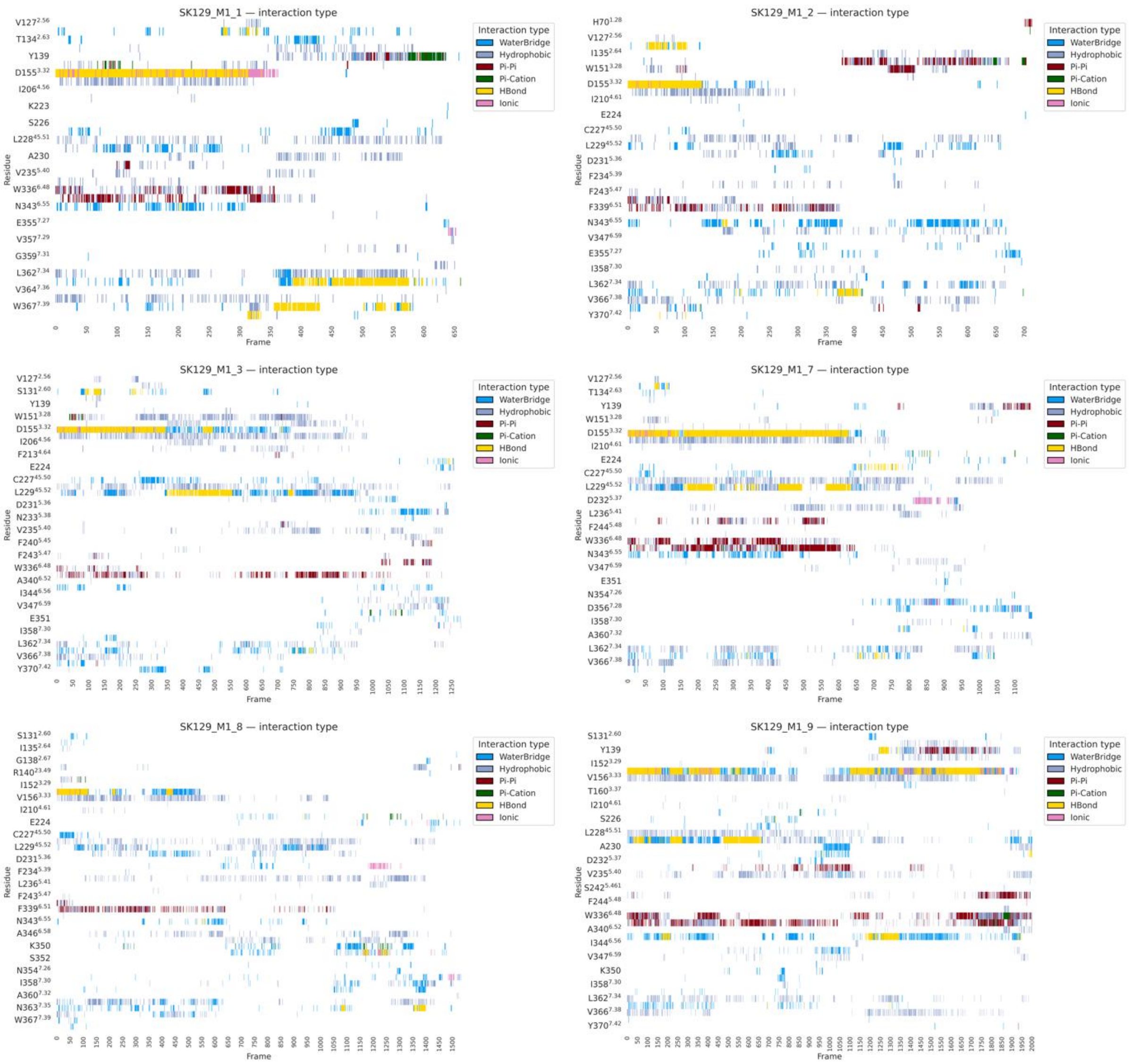

Figure S48. Interactions between individual amino acid residues and the ligand as identified through iMetaD simulation (**compound 5 mutation F340$^{6.52}$A**). Different colors denote distinct types of interactions: water bridge – blue; hydrophobic – blue-gray; π–π – ruby; π–cation – green; hydrogen bond – yellow; ionic – magenta.

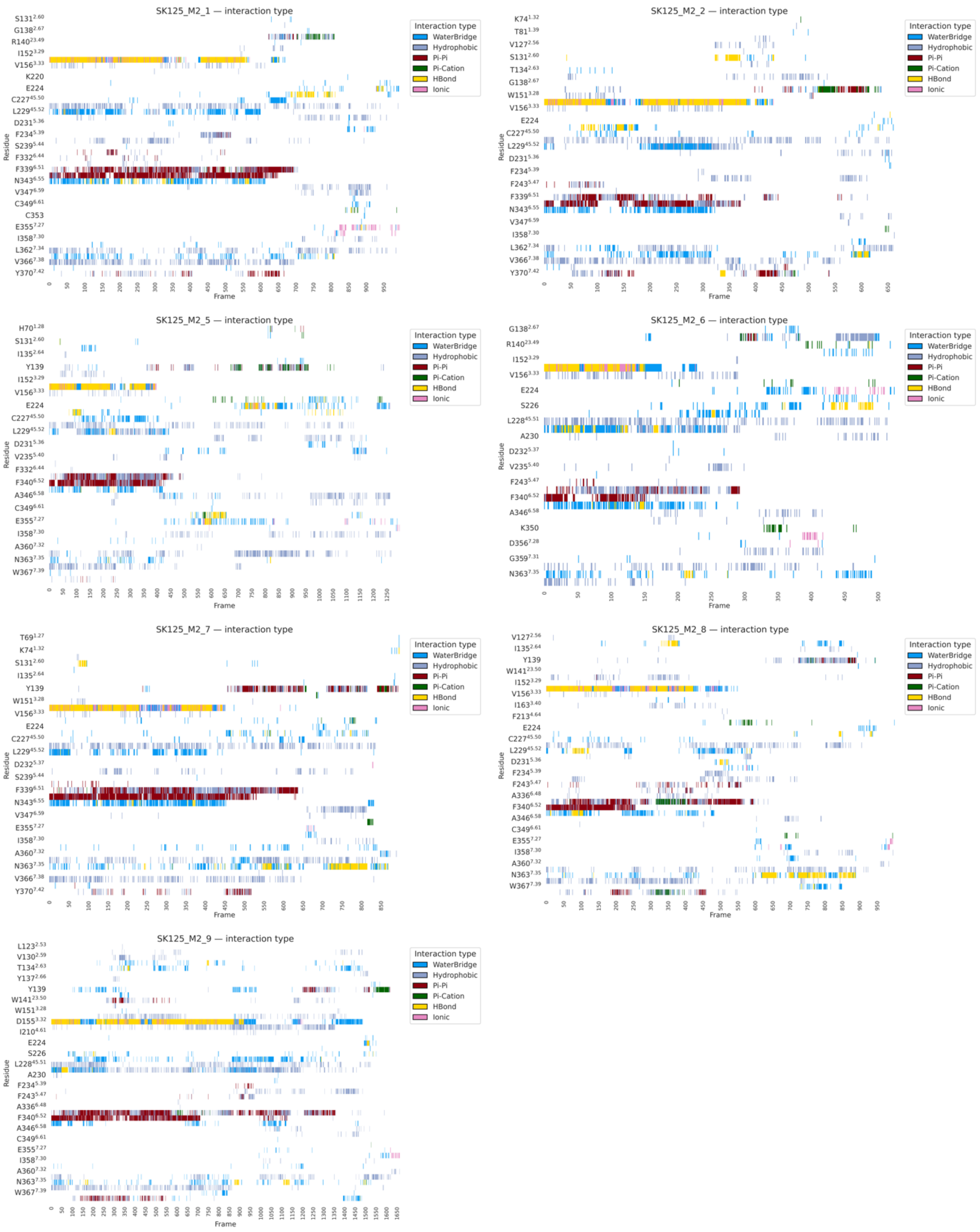

Figure S49. Interactions between individual amino acid residues and the ligand as identified through iMetaD simulation (**compound 1 mutation W336$^{6.48}$A**). Different colors denote distinct types of interactions: water bridge – blue; hydrophobic – blue-gray; π–π – ruby; π–cation – green; hydrogen bond – yellow; ionic – magenta.

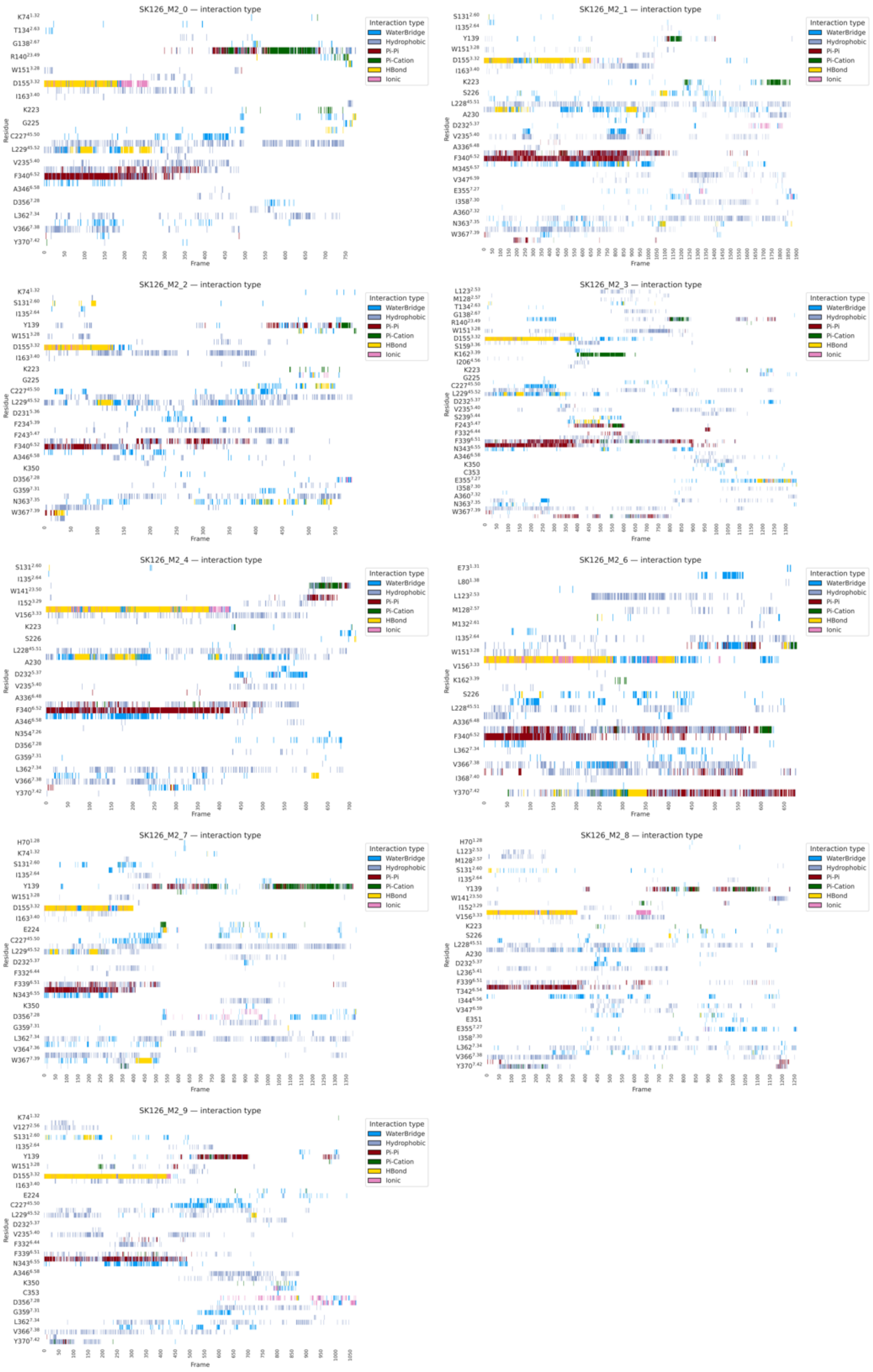

Figure S50. Interactions between individual amino acid residues and the ligand as identified through iMetaD simulation (**compound 2 mutation W336$^{6.48}$A**). Different colors denote distinct types of interactions: water bridge – blue; hydrophobic – blue-gray; π–π – ruby; π–cation – green; hydrogen bond – yellow; ionic – magenta.

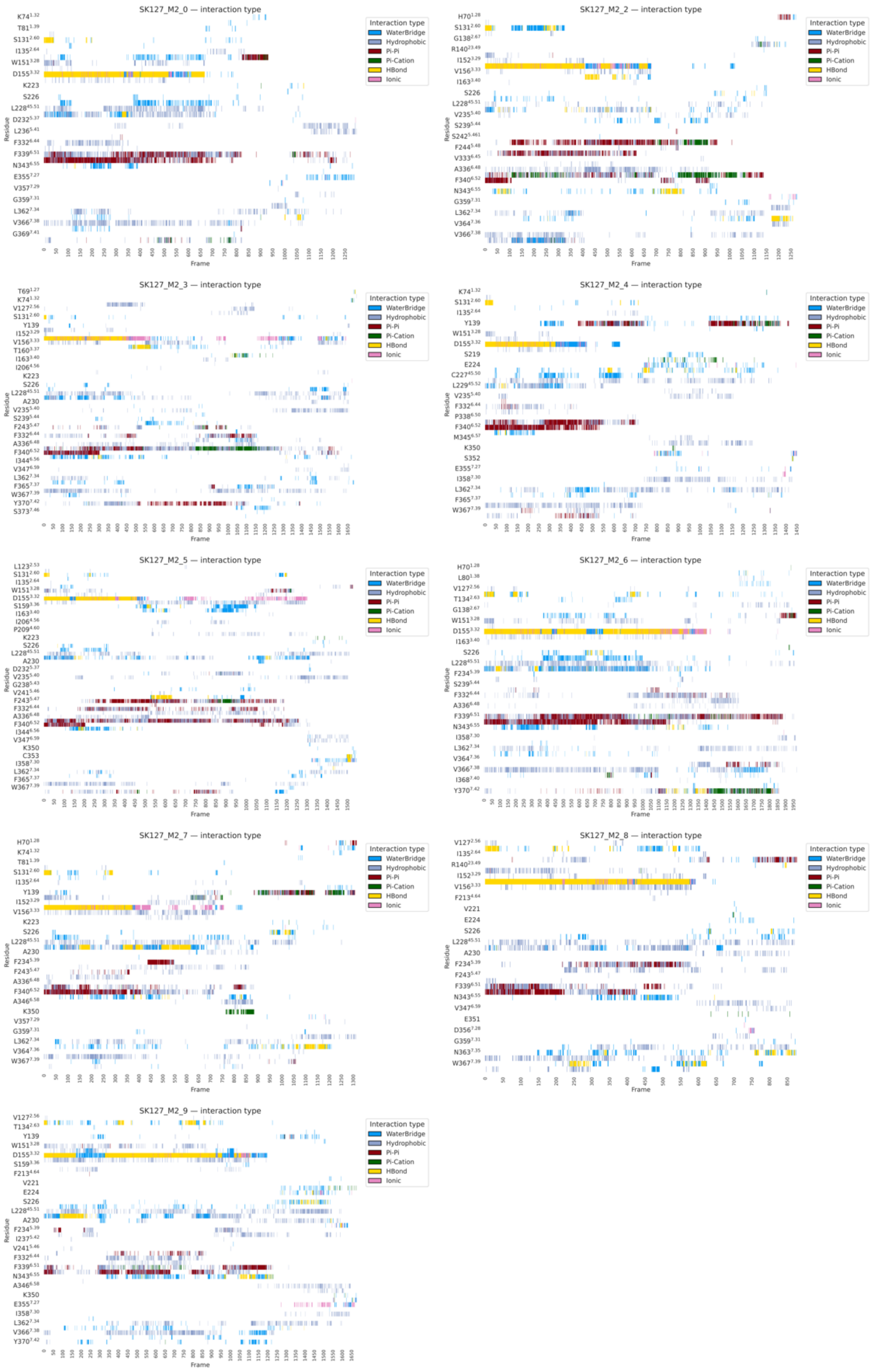

Figure S51. Interactions between individual amino acid residues and the ligand as identified through iMetaD simulation (**compound 3 mutation W336$^{6.48}$A**). Different colors denote distinct types of interactions: water bridge – blue; hydrophobic – blue-gray; π–π – ruby; π–cation – green; hydrogen bond – yellow; ionic – magenta.

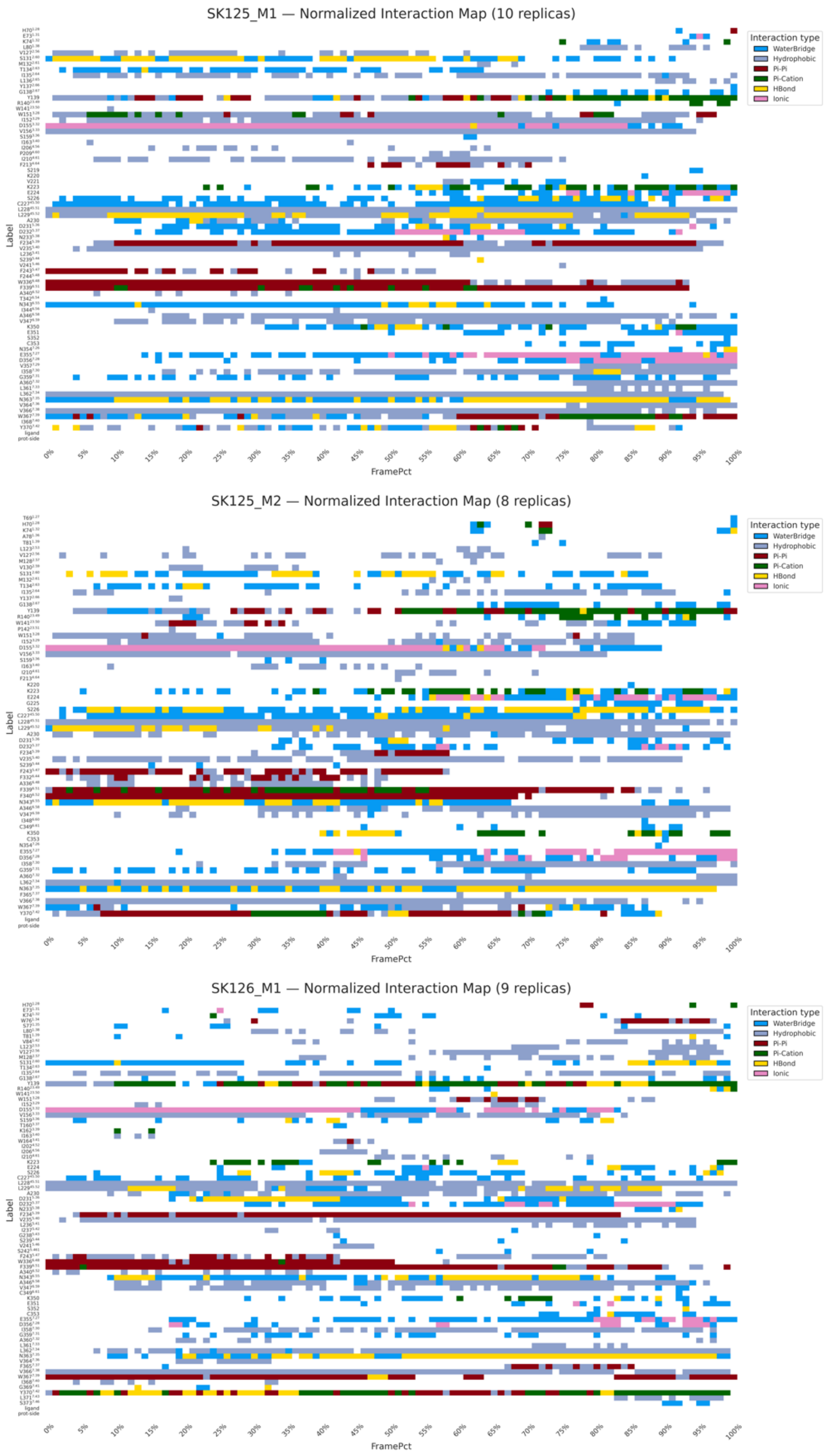

Figure S52. Normalized interaction map between individual amino acid residues and the ligand as identified through iMetaD simulation (**compounds 1_M1-2_M1**). Different colors denote distinct types of interactions: water bridge – blue; hydrophobic – blue-gray; π–π – ruby; π–cation – green; hydrogen bond – yellow; ionic – magenta.

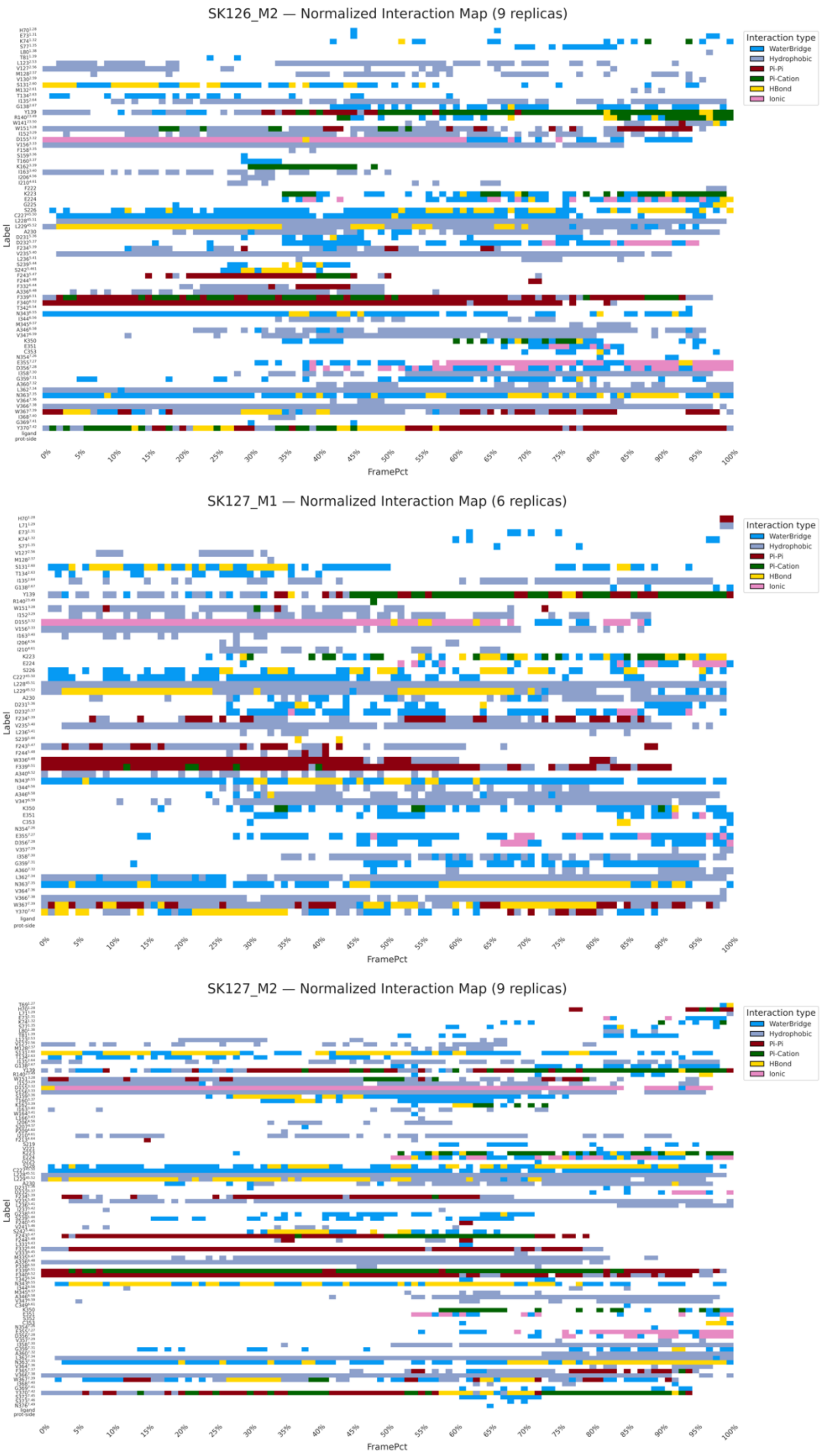

Figure S53. Normalized interaction map between individual amino acid residues and the ligand as identified through iMetaD simulation (**compounds 2_M2-3_M1**). Different colors denote distinct types of interactions: water bridge – blue; hydrophobic – blue-gray; π–π – ruby; π–cation – green; hydrogen bond – yellow; ionic – magenta.

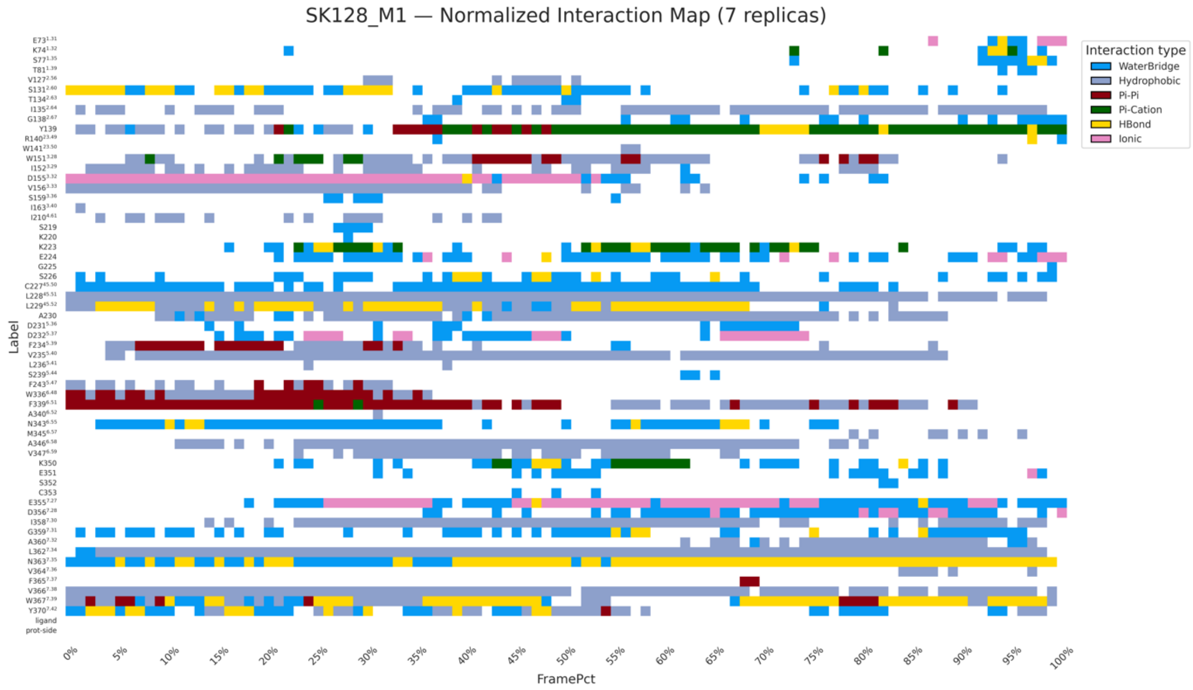

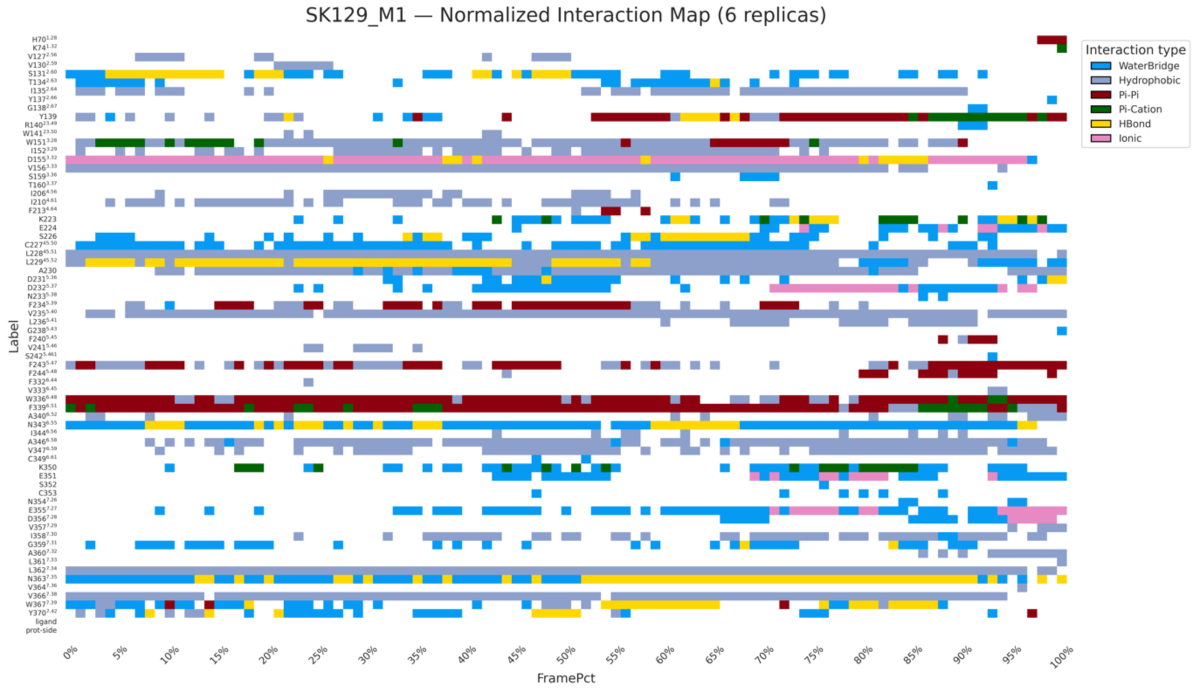

Figure S54. Normalized interaction map between individual amino acid residues and the ligand as identified through iMetaD simulation (**compounds 3_M1-4_M1**). Different colors denote distinct types of interactions: water bridge – blue; hydrophobic – blue-gray; π–π – ruby; π–cation – green; hydrogen bond – yellow; ionic – magenta.

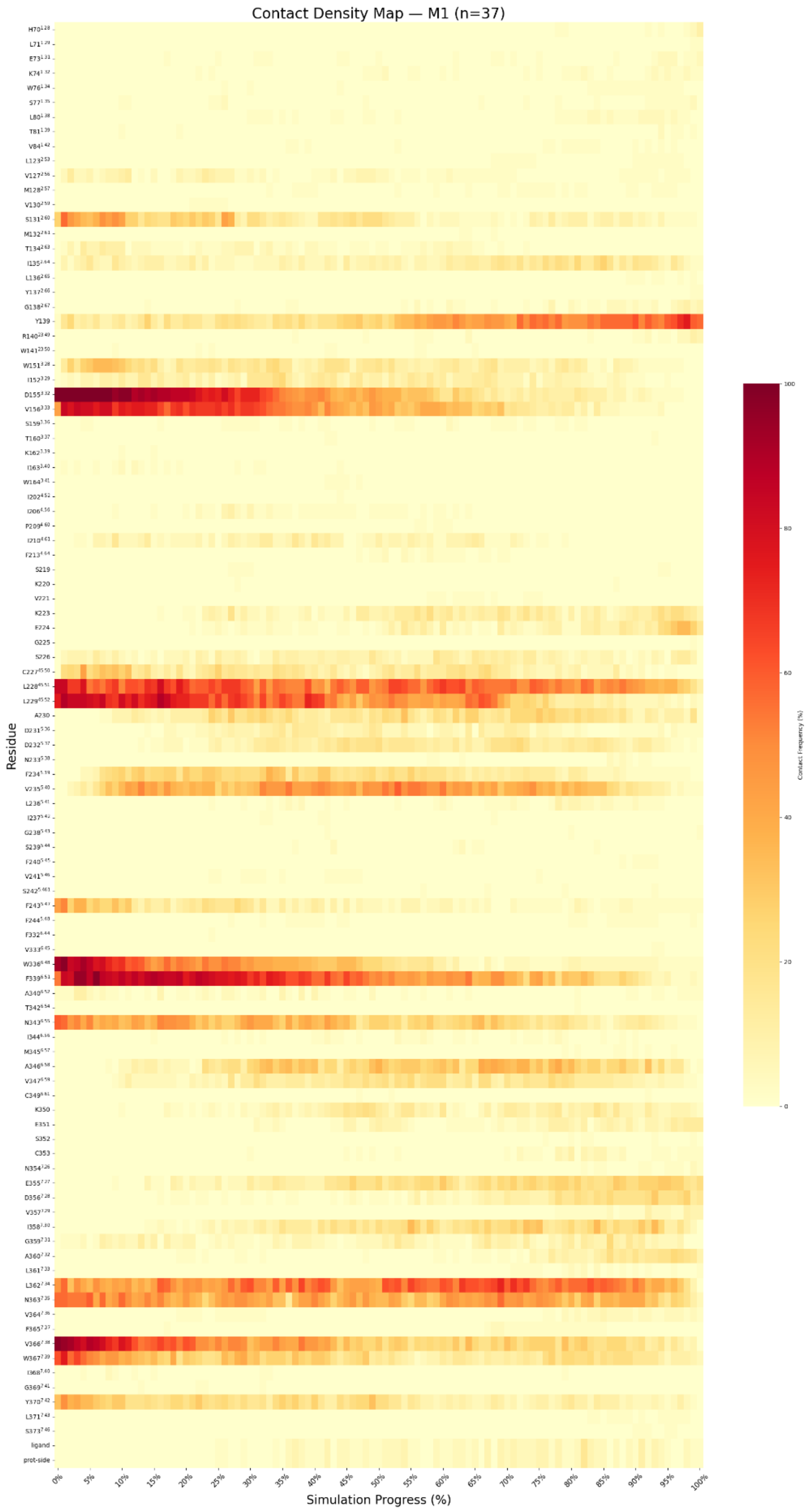

Figure S55. Global interaction density map for the F340$^{6.52}$A mutant. Consensus map of ligand–residue interactions identified across iMetaD trajectories for the 5-HT$_{2A}$ receptor carrying the F340$^{6.52}$A substitution.

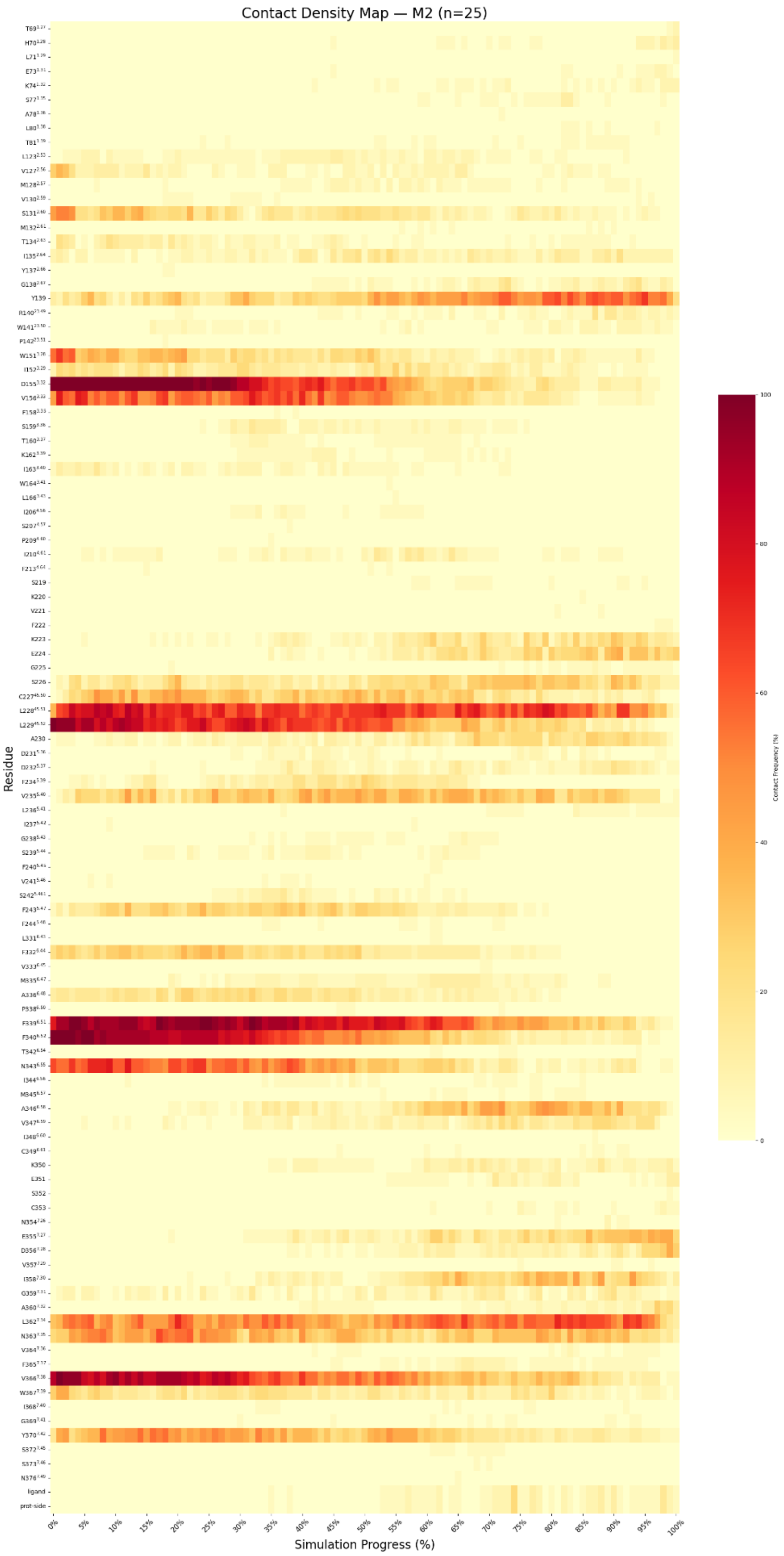

Figure S56. Global interaction density map for the W336$^{6.48}$A mutant. Consensus map of ligand–residue interactions identified across iMetaD trajectories for the 5-HT$_{2A}$ receptor carrying the W336$^{6.48}$A substitution.

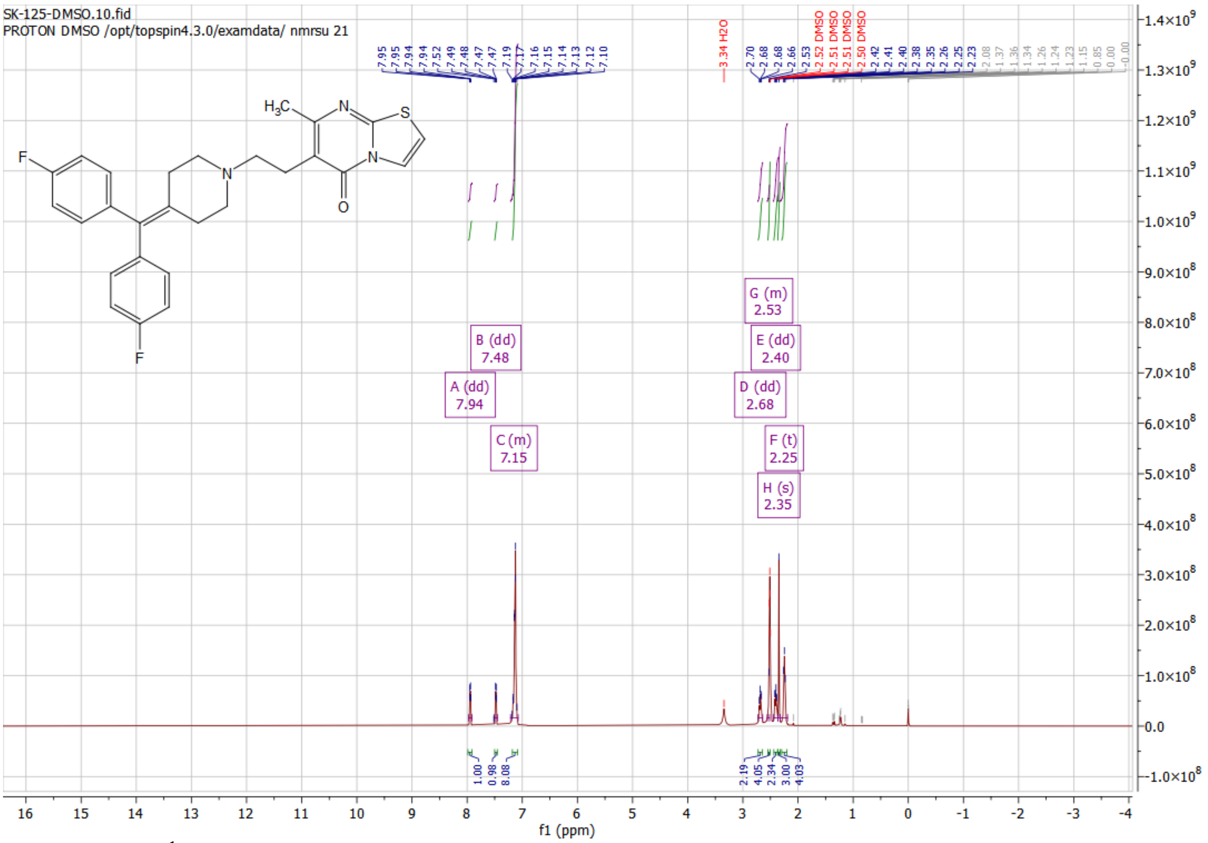

Figure S57. ^{1}H NMR spectrum for Compound 1.

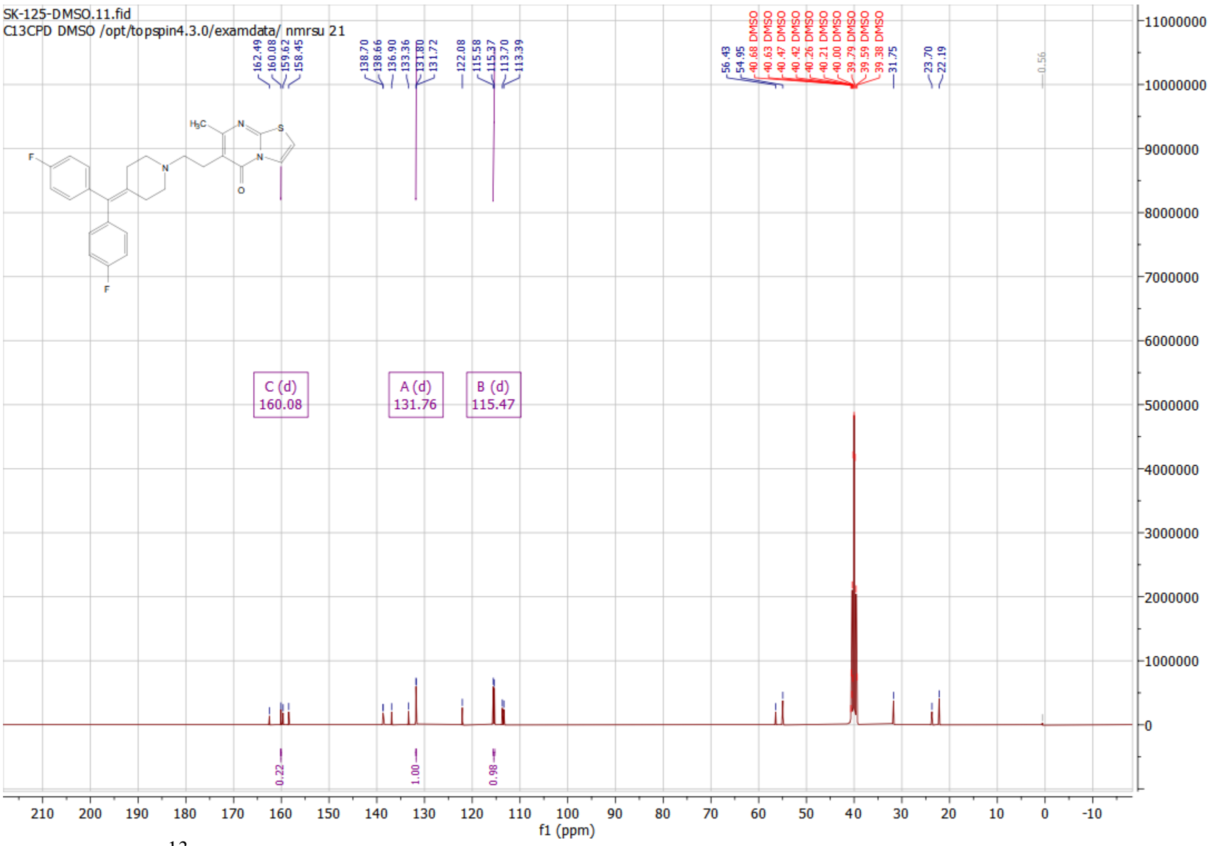

Figure S58. ^{13}C NMR spectrum for Compound 1.

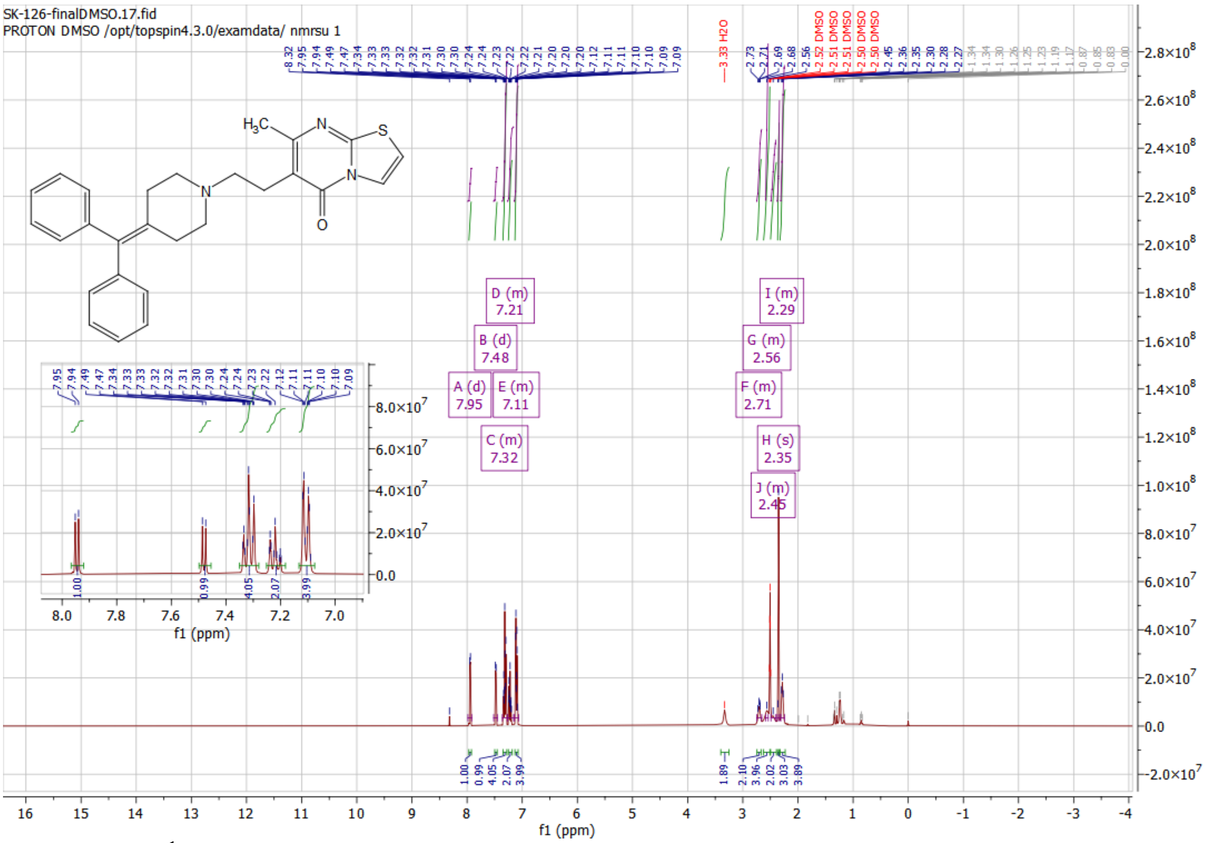

Figure S59. ^{1}H NMR spectrum for Compound 2.

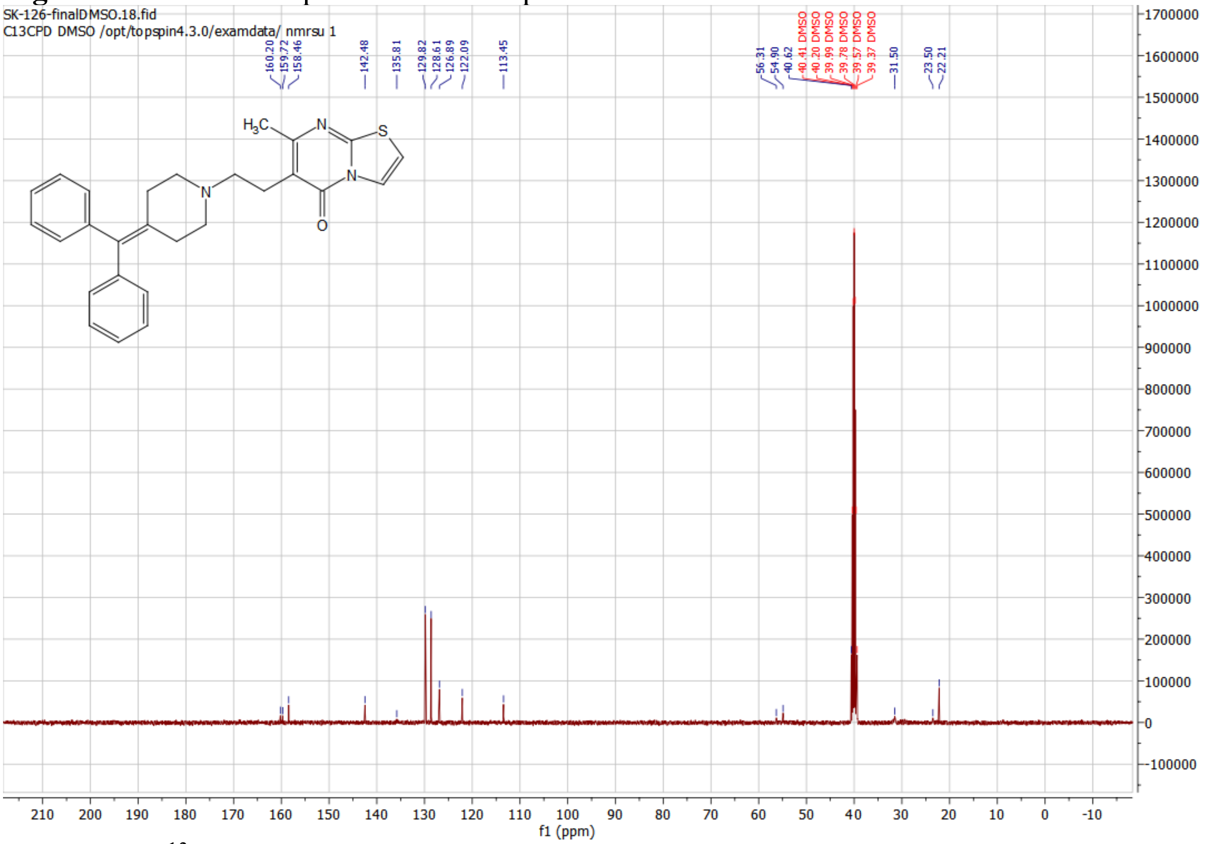

Figure S60. ^{13}C NMR spectrum for Compound 2.

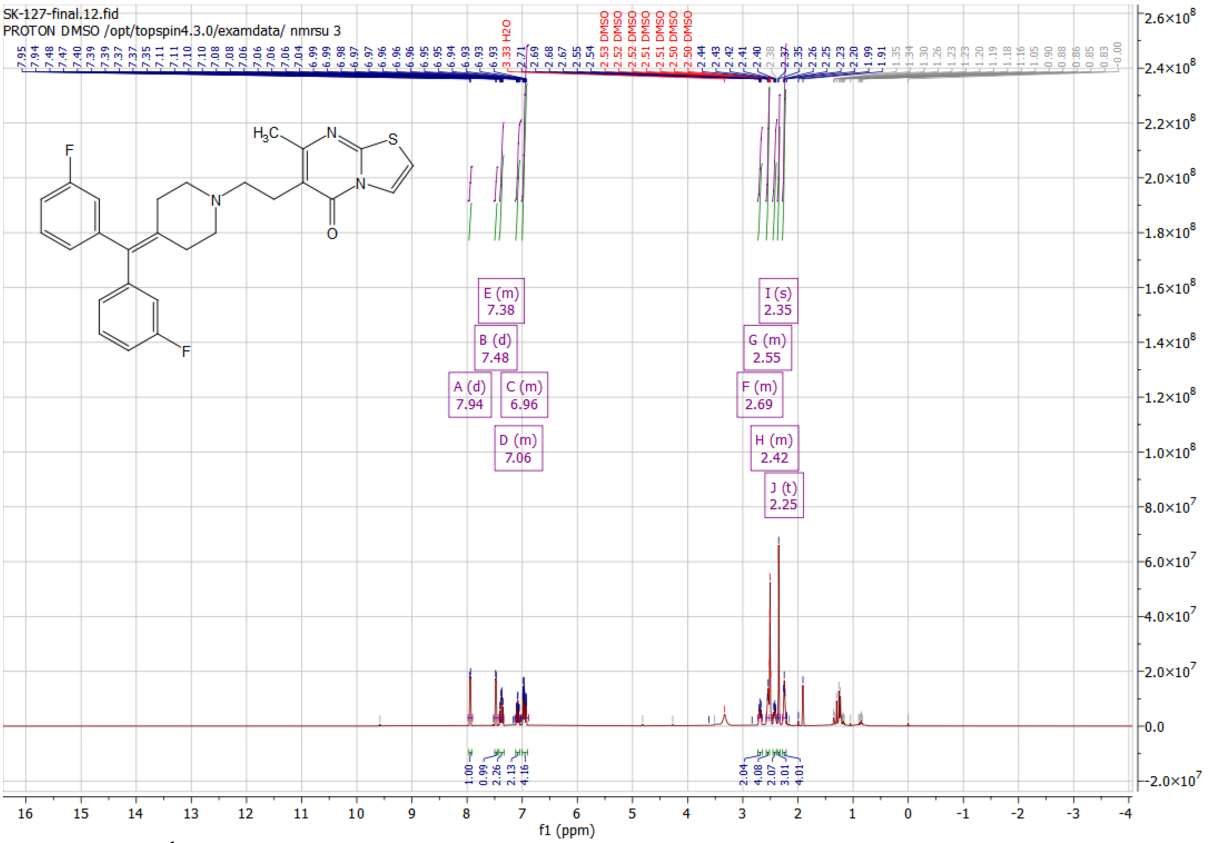

Figure S61. ^{1}H NMR spectrum for Compound 3.

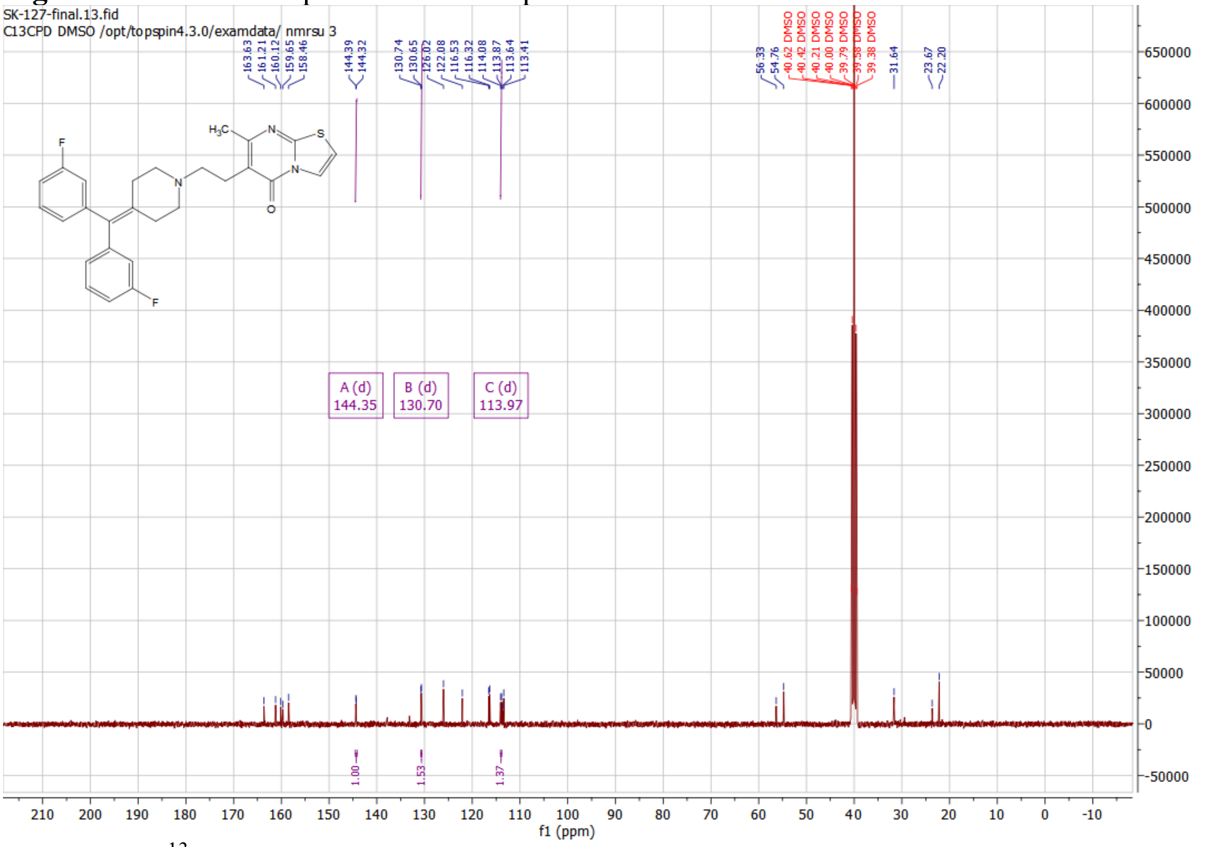

Figure S62. ^{13}C NMR spectrum for Compound 3.

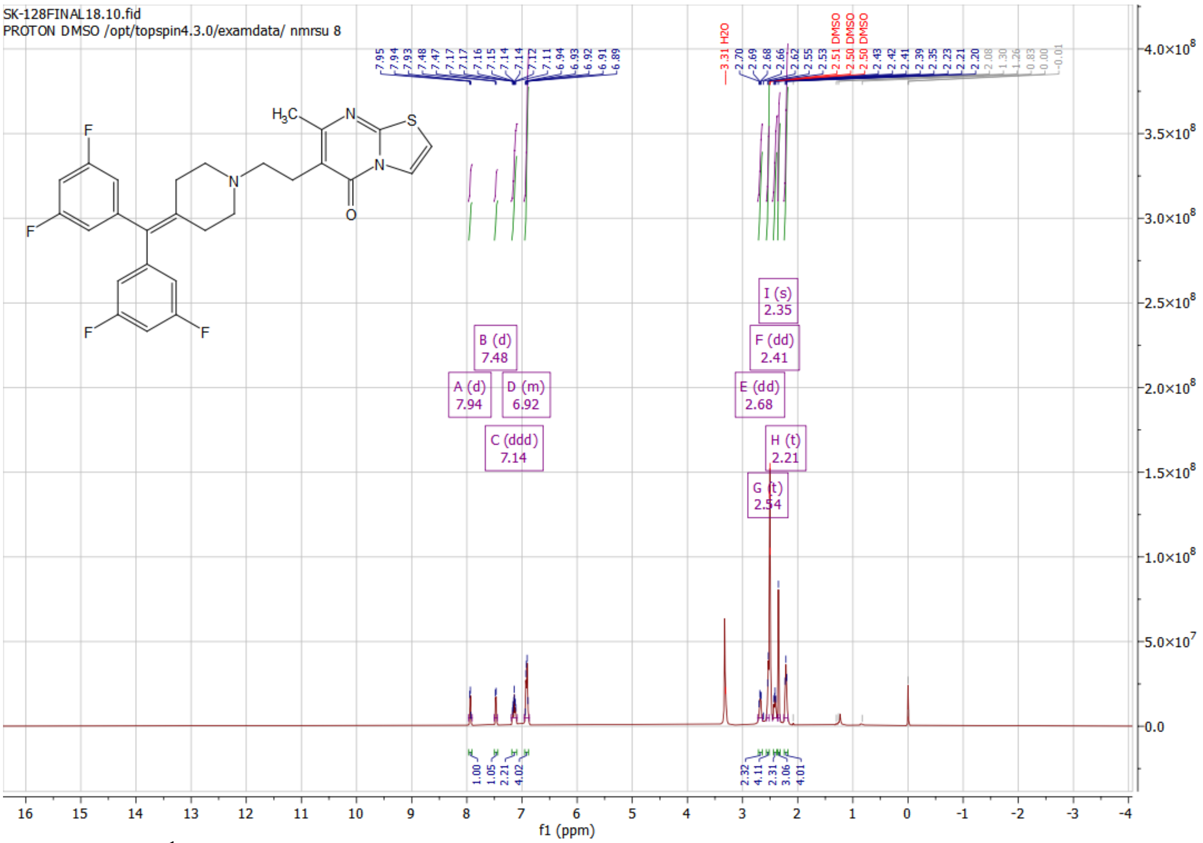

Figure S63. ^{1}H NMR spectrum for Compound 4.

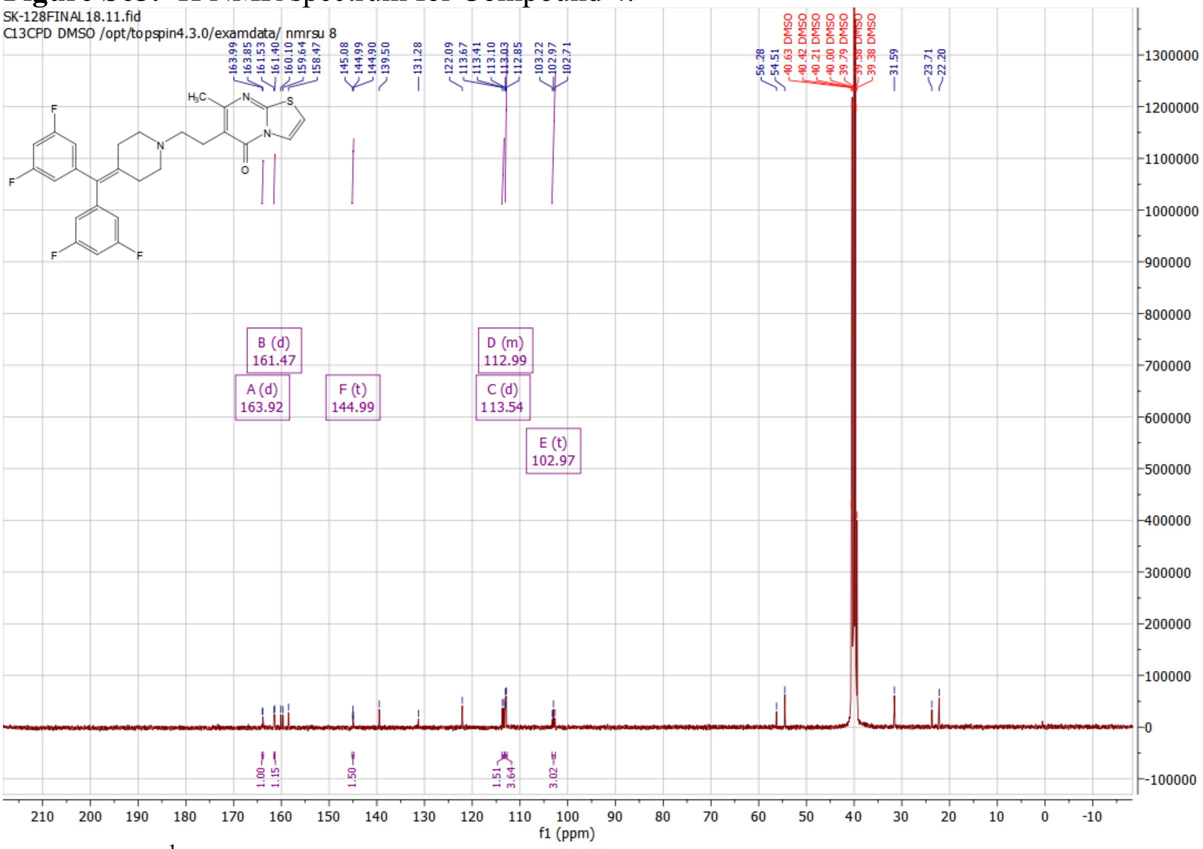

Figure S64. ^{1}H NMR spectrum for Compound 4.

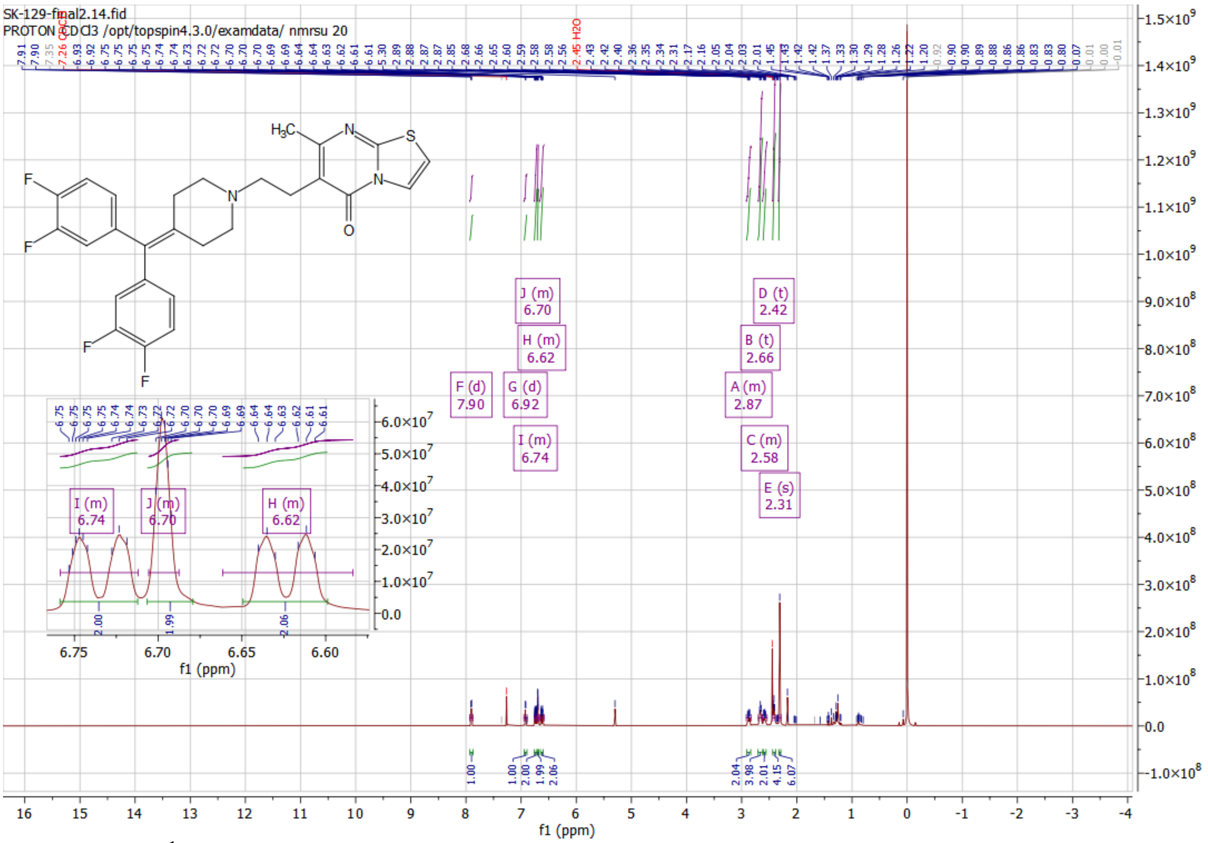

Figure S65. ^{1}H NMR spectrum for Compound 5.

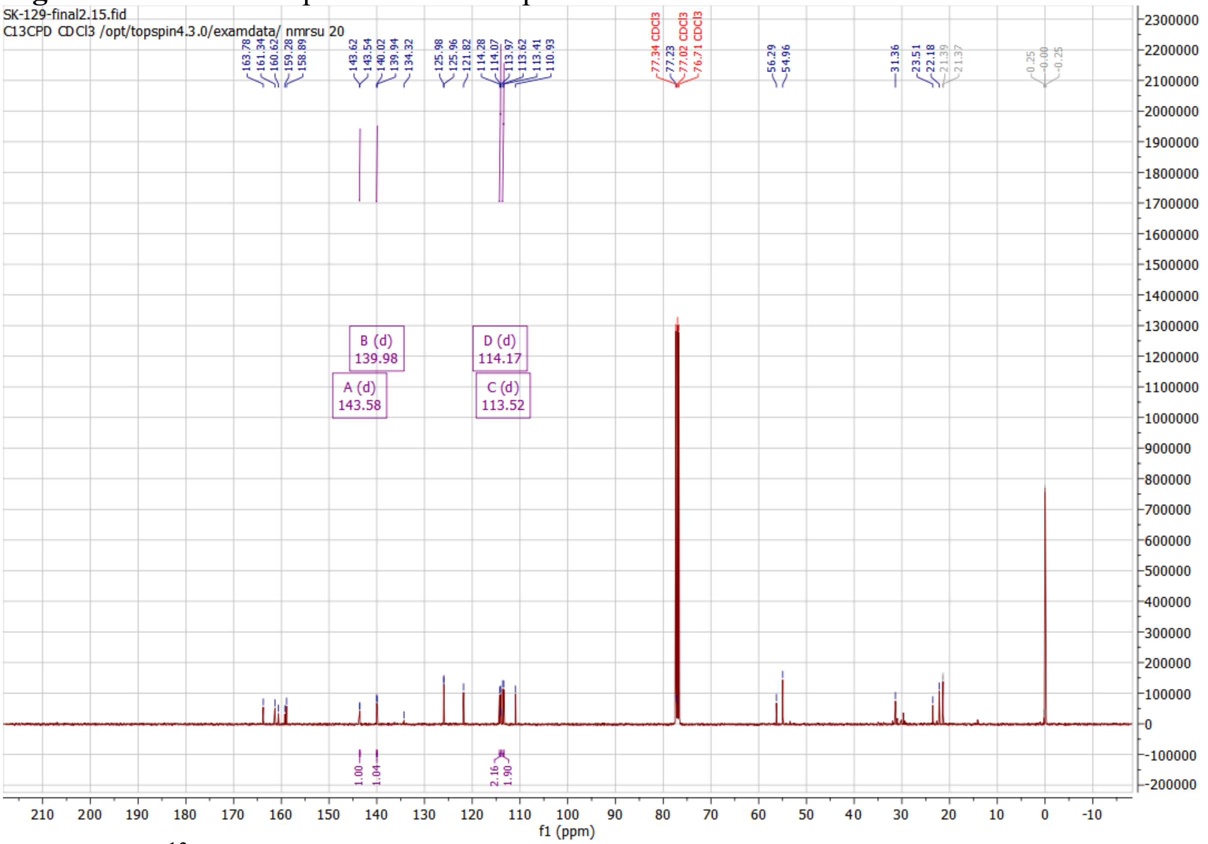

Figure S66. ^{13}C NMR spectrum for Compound 5.

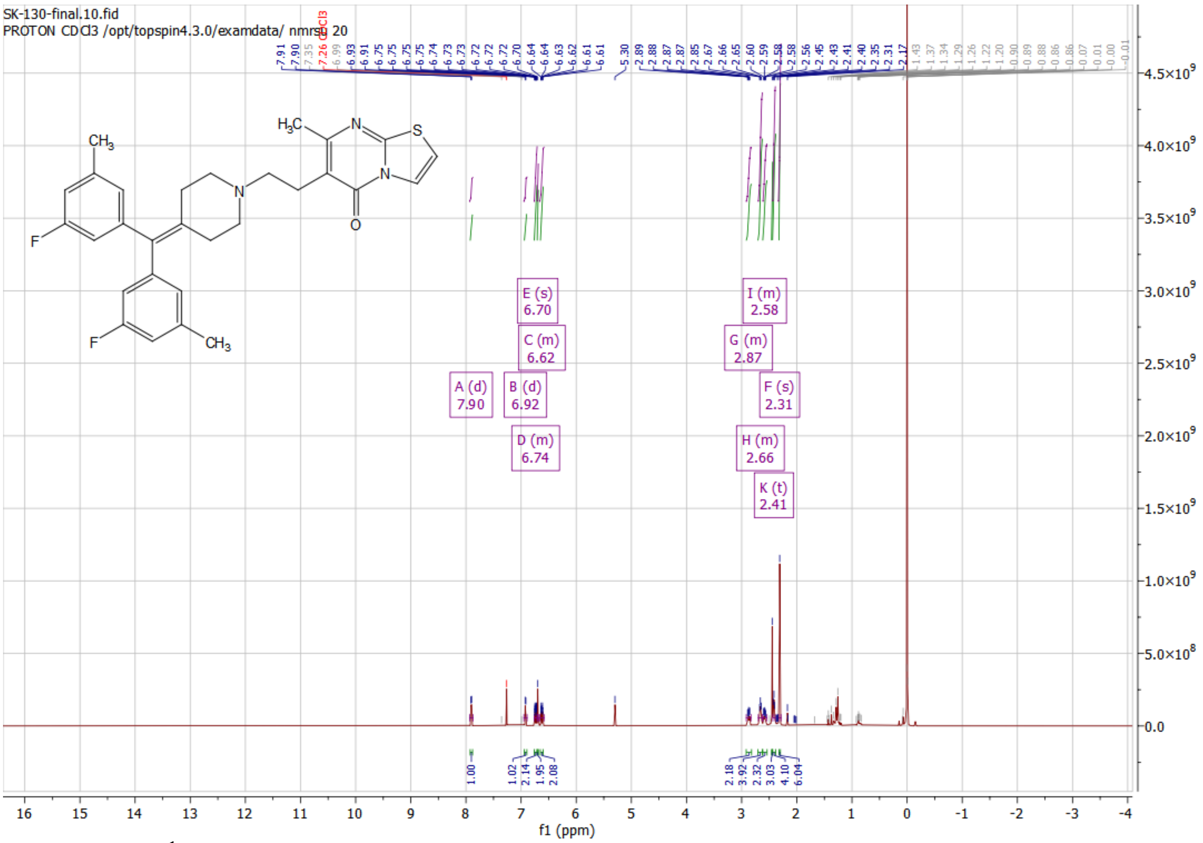

Figure S67. ^{1}H NMR spectrum for Compound 6.

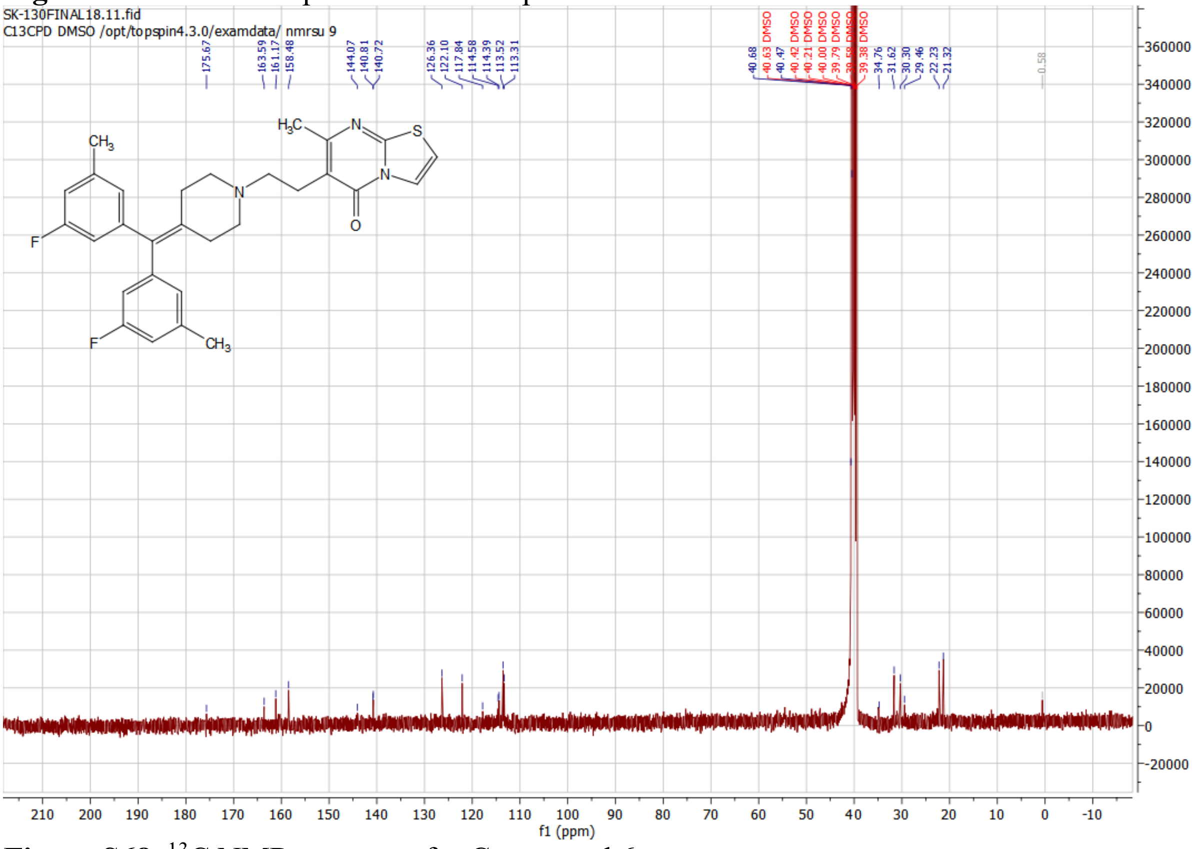

Figure S68. ^{13}C NMR spectrum for Compound 6.

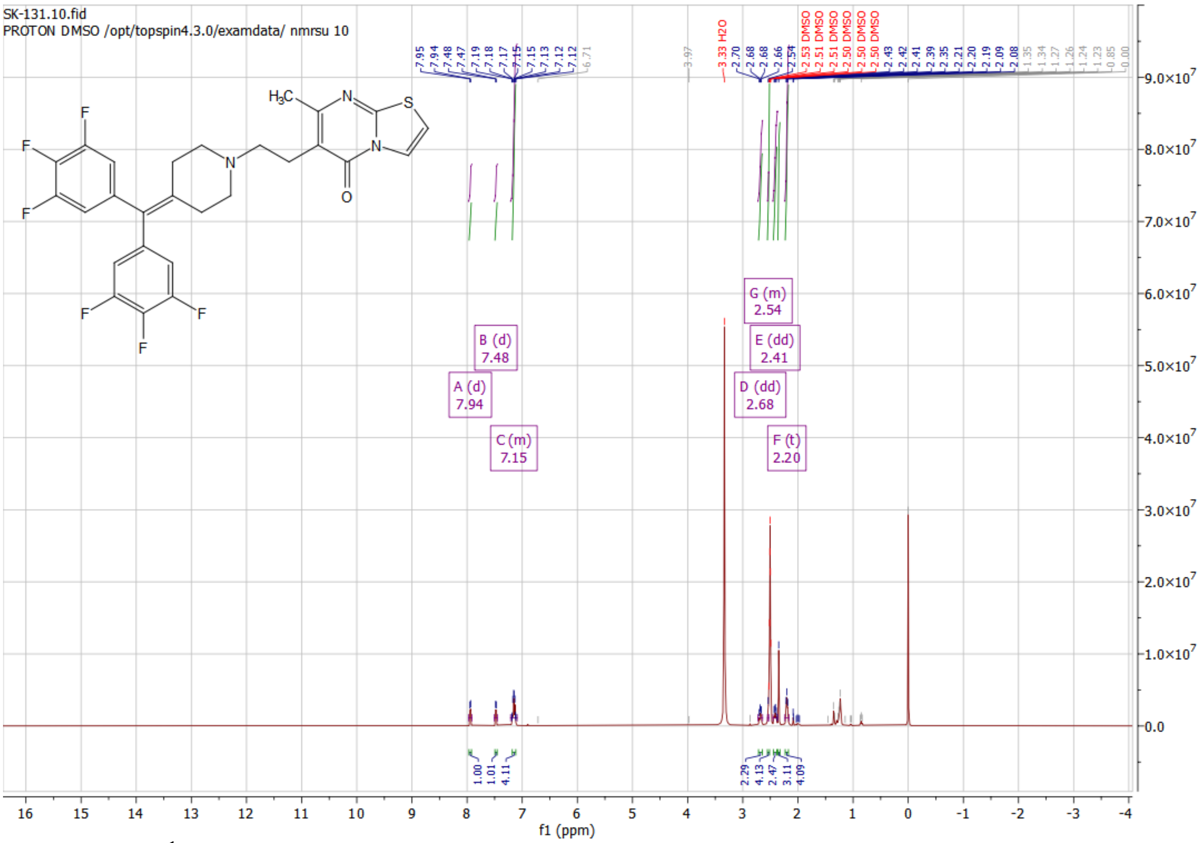

Figure S69. ^{1}H NMR spectrum for Compound 7.

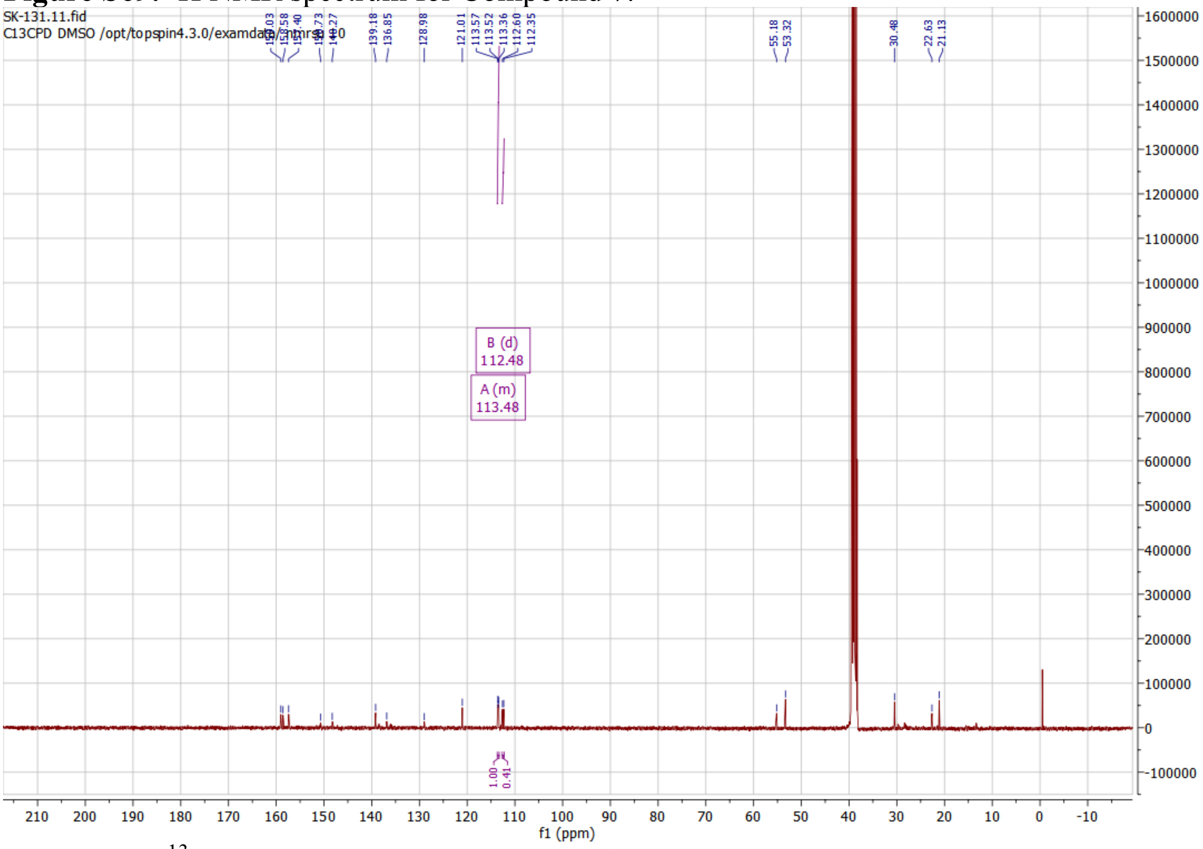

Figure S70. ^{13}C NMR spectrum for Compound 7.

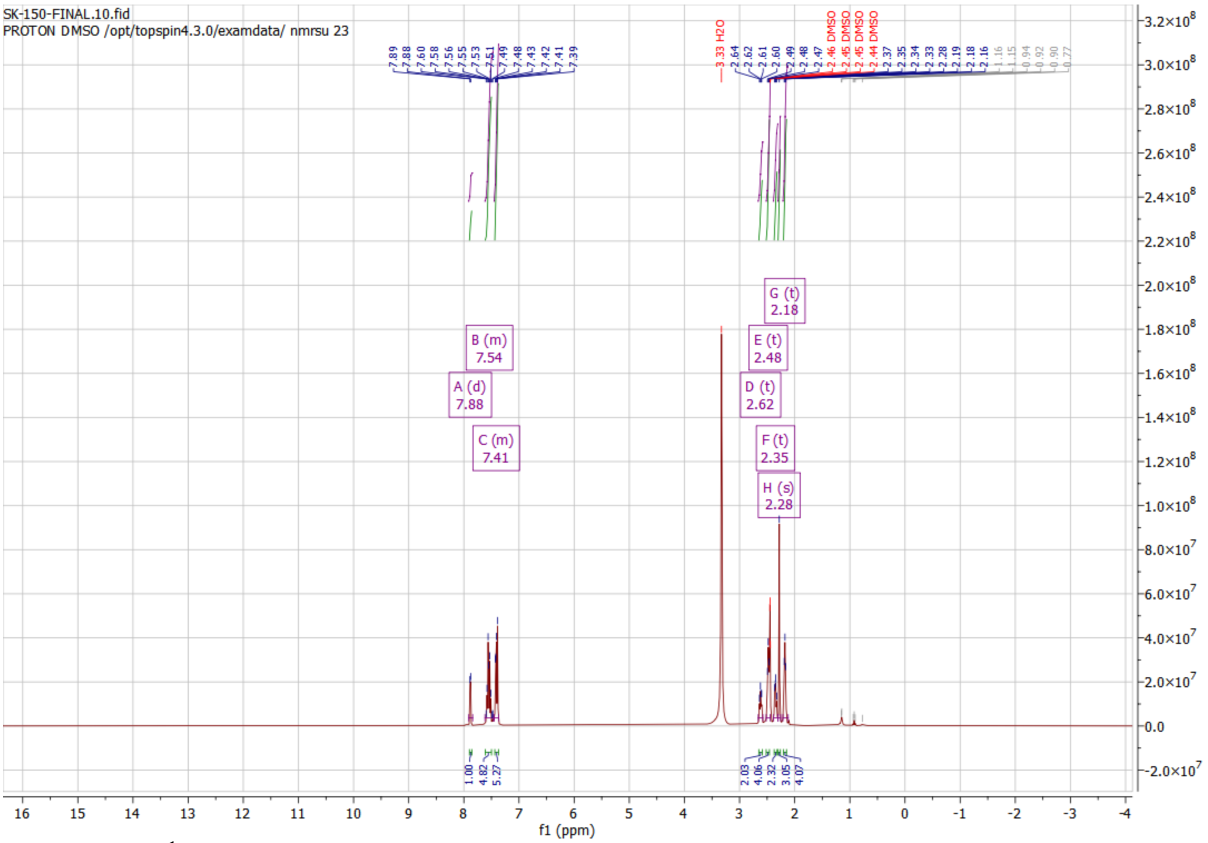

Figure S71. ^{1}H NMR spectrum for Compound 8.

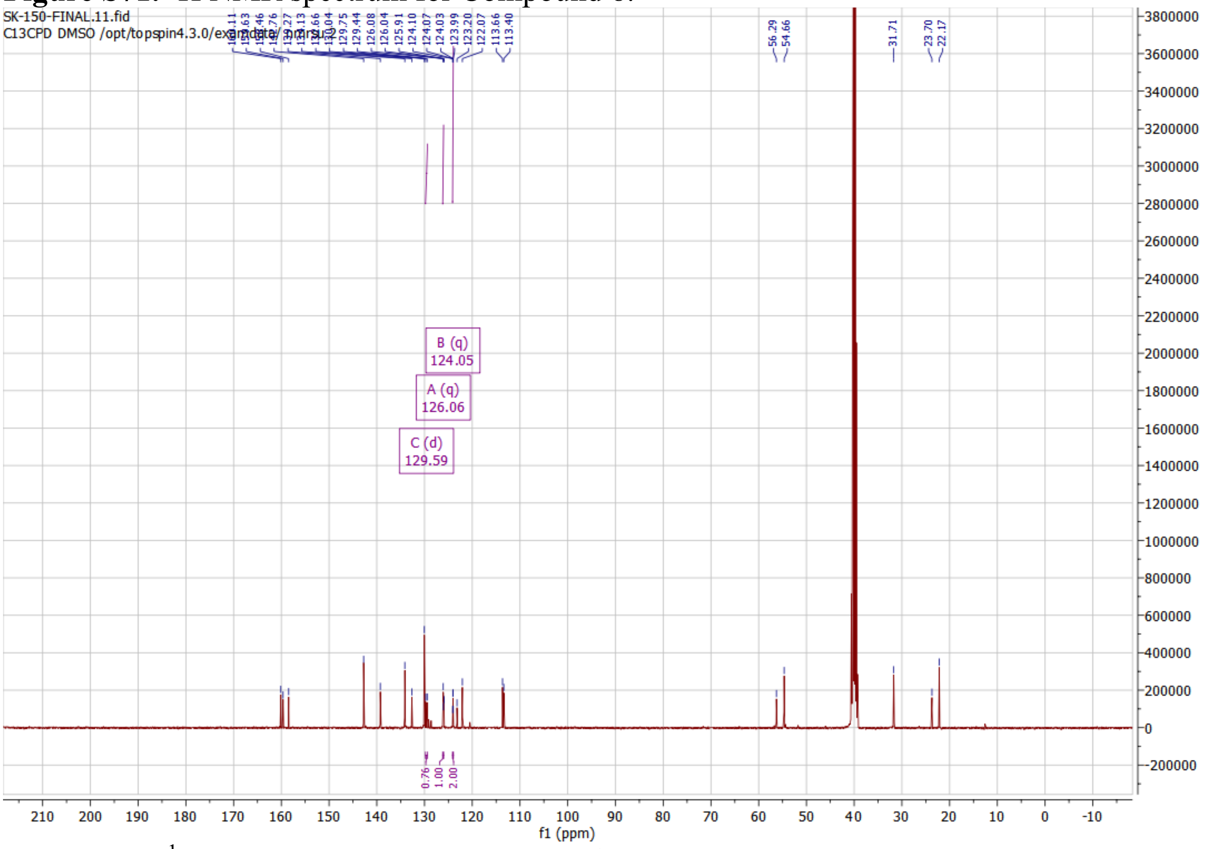

Figure S72. ^{1}H NMR spectrum for Compound 8.

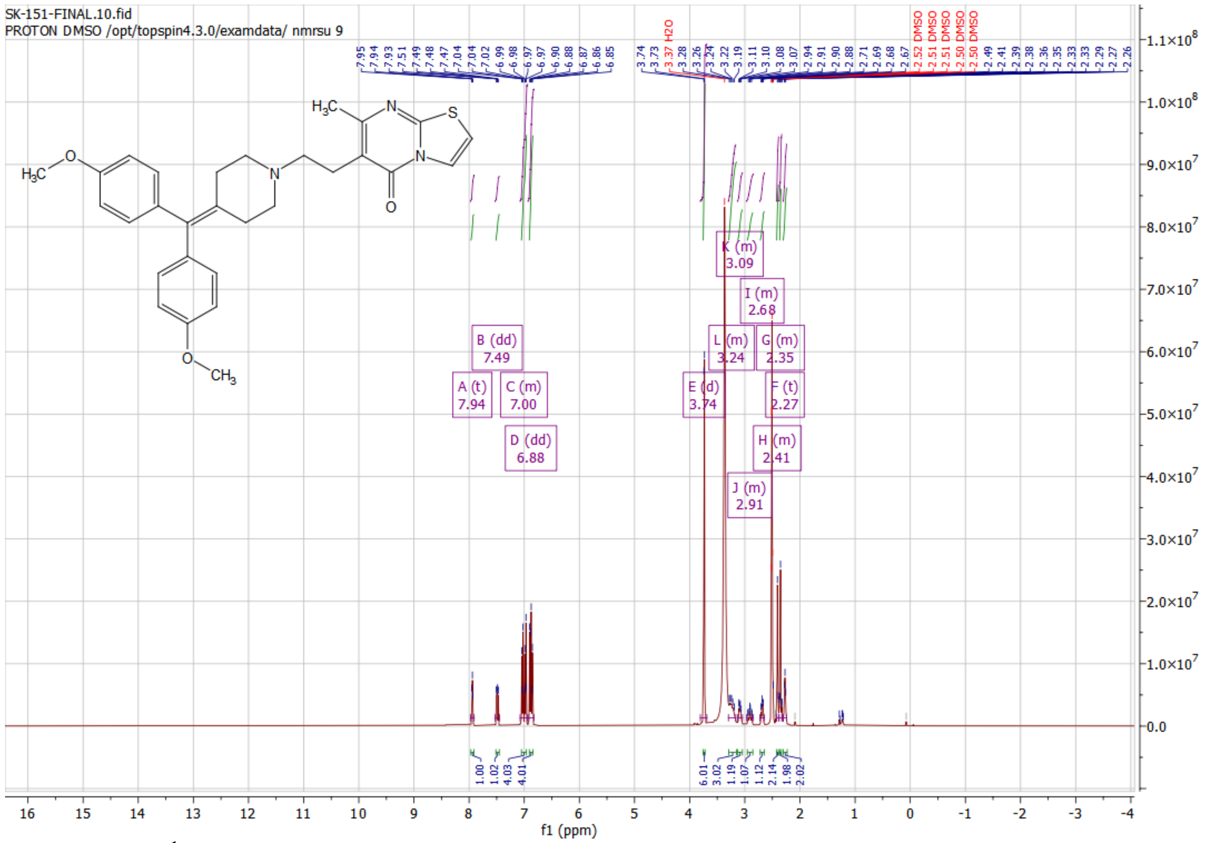

Figure S73. ^{1}H NMR spectrum for Compound 9.

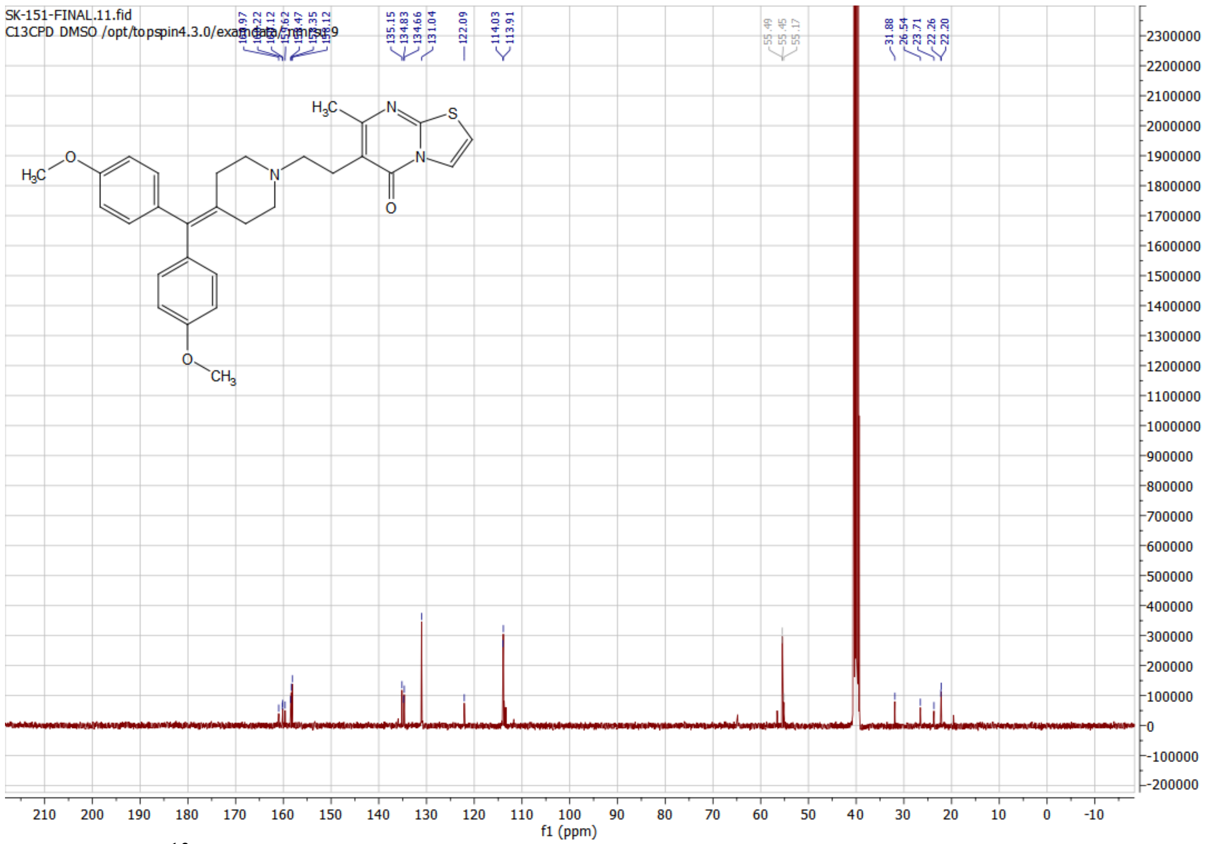

Figure S74. ^{13}C NMR spectrum for Compound 9.

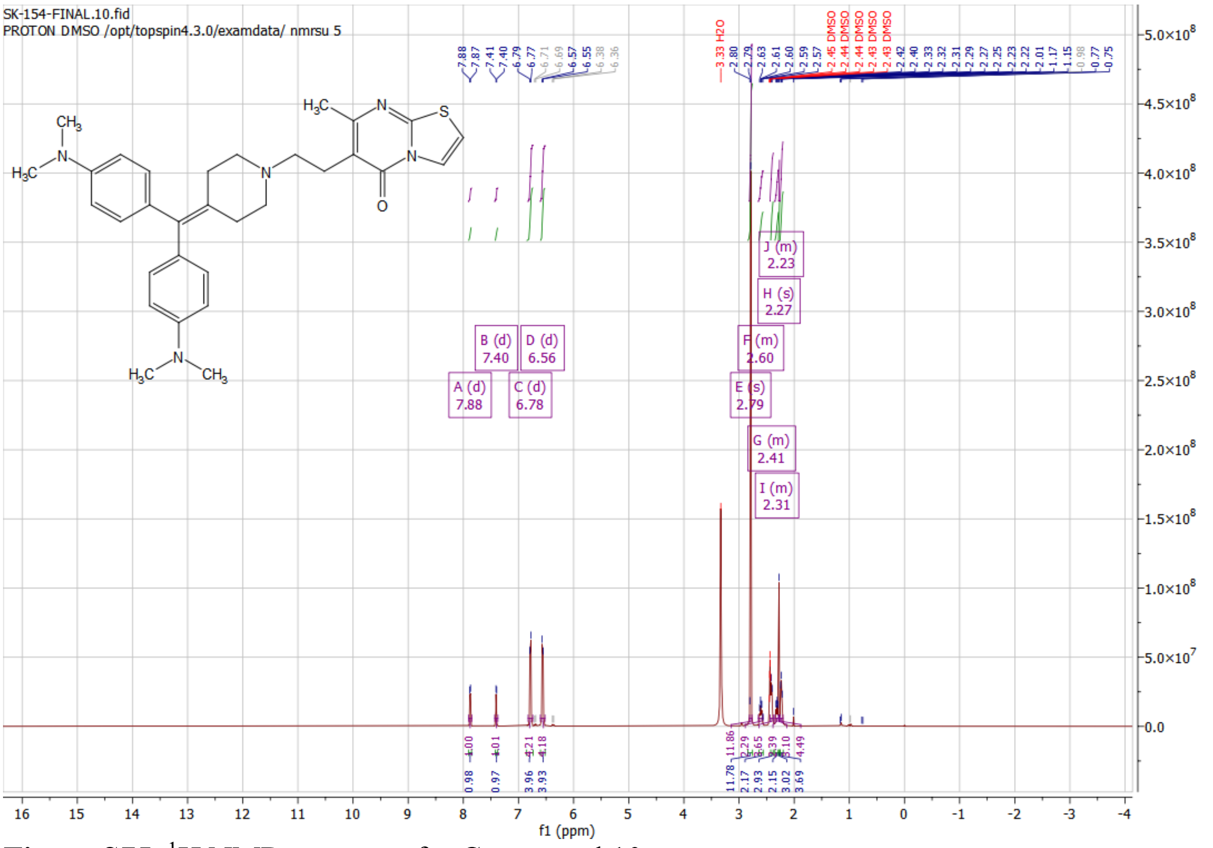

Figure S75. ^{1}H NMR spectrum for Compound 10.

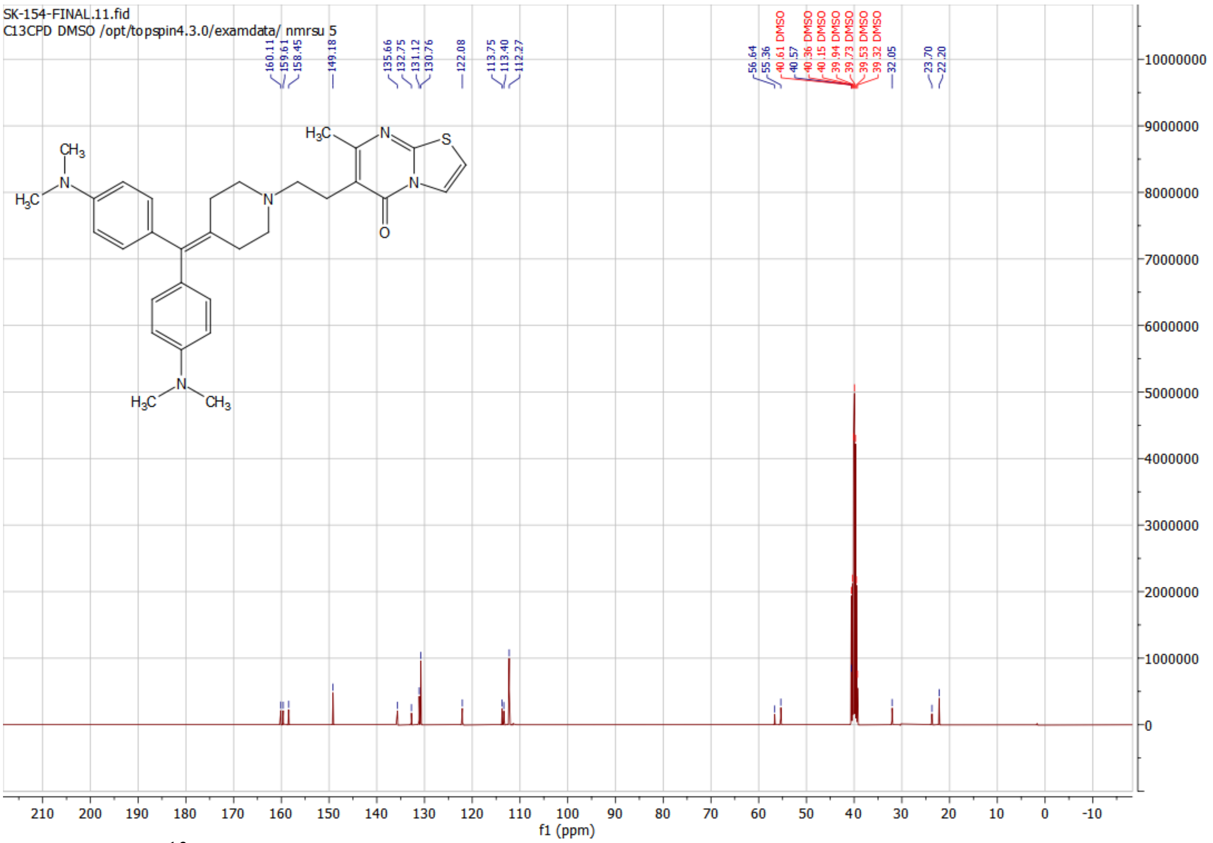

Figure S76. ^{13}C NMR spectrum for Compound 10.

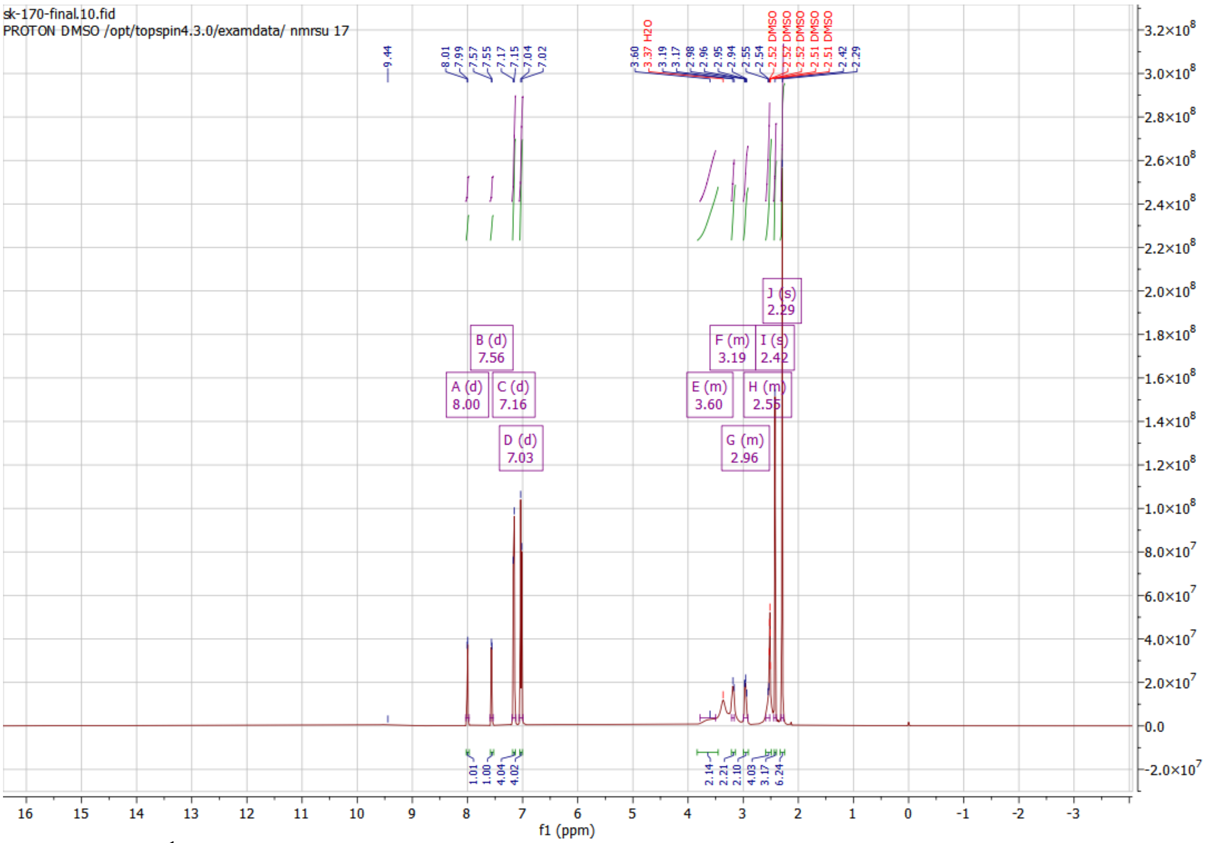

Figure S77. ^{1}H NMR spectrum for Compound 11.

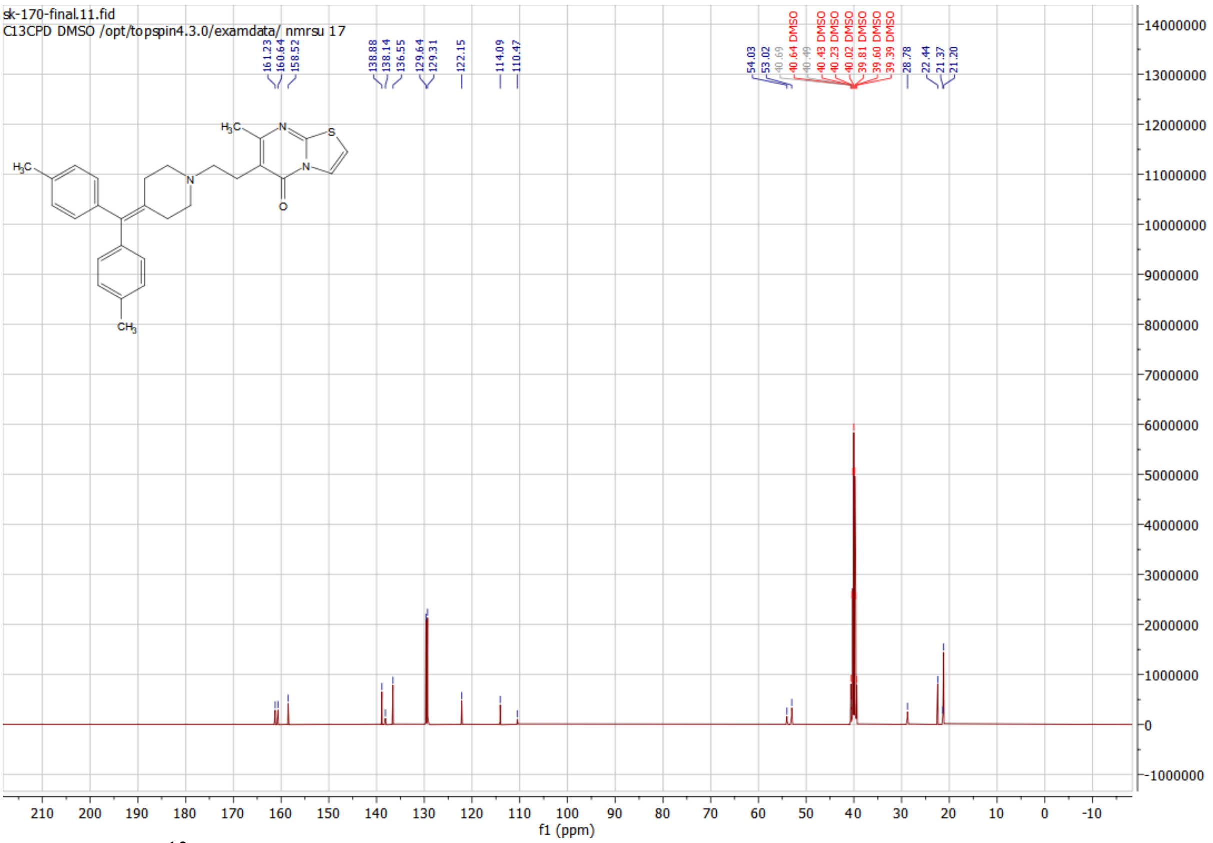

Figure S78. ^{13}C NMR spectrum for Compound 11.

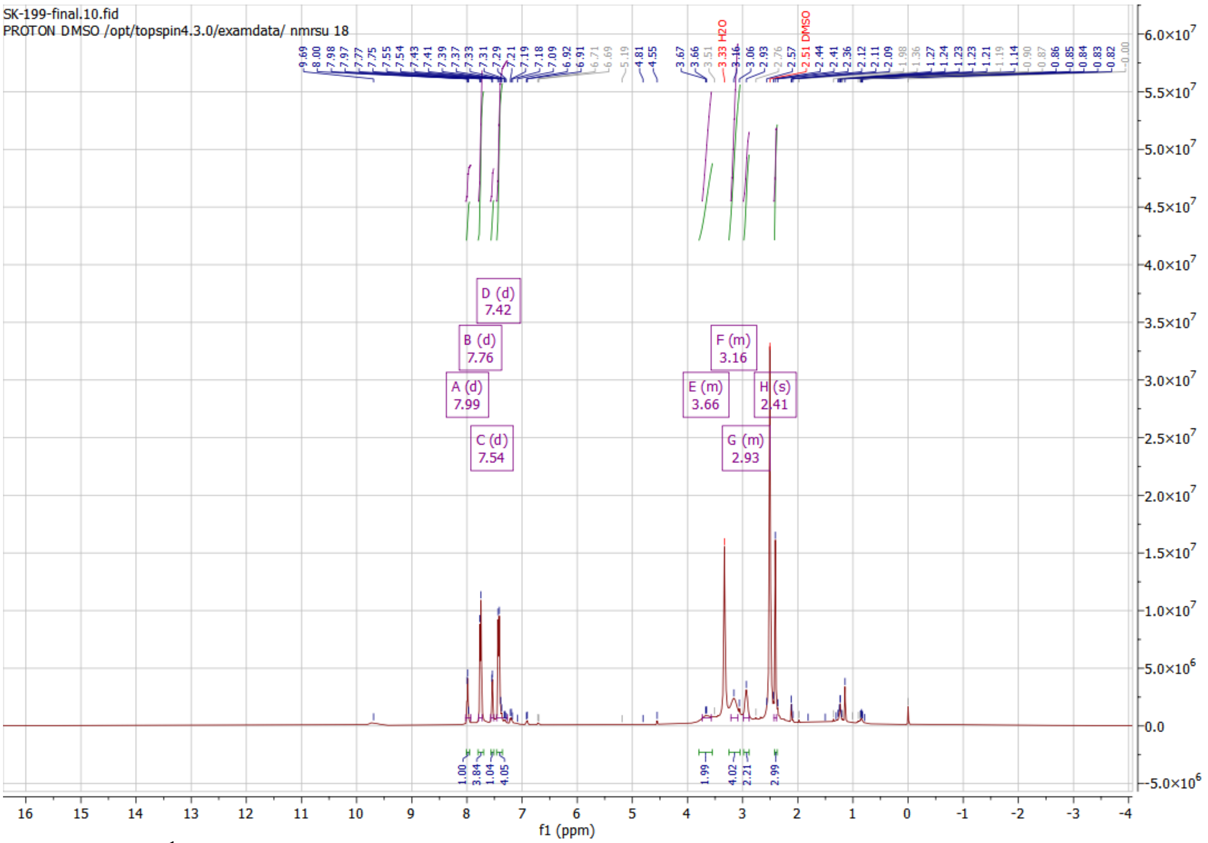

Figure S79. ^{1}H NMR spectrum for Compound 12.

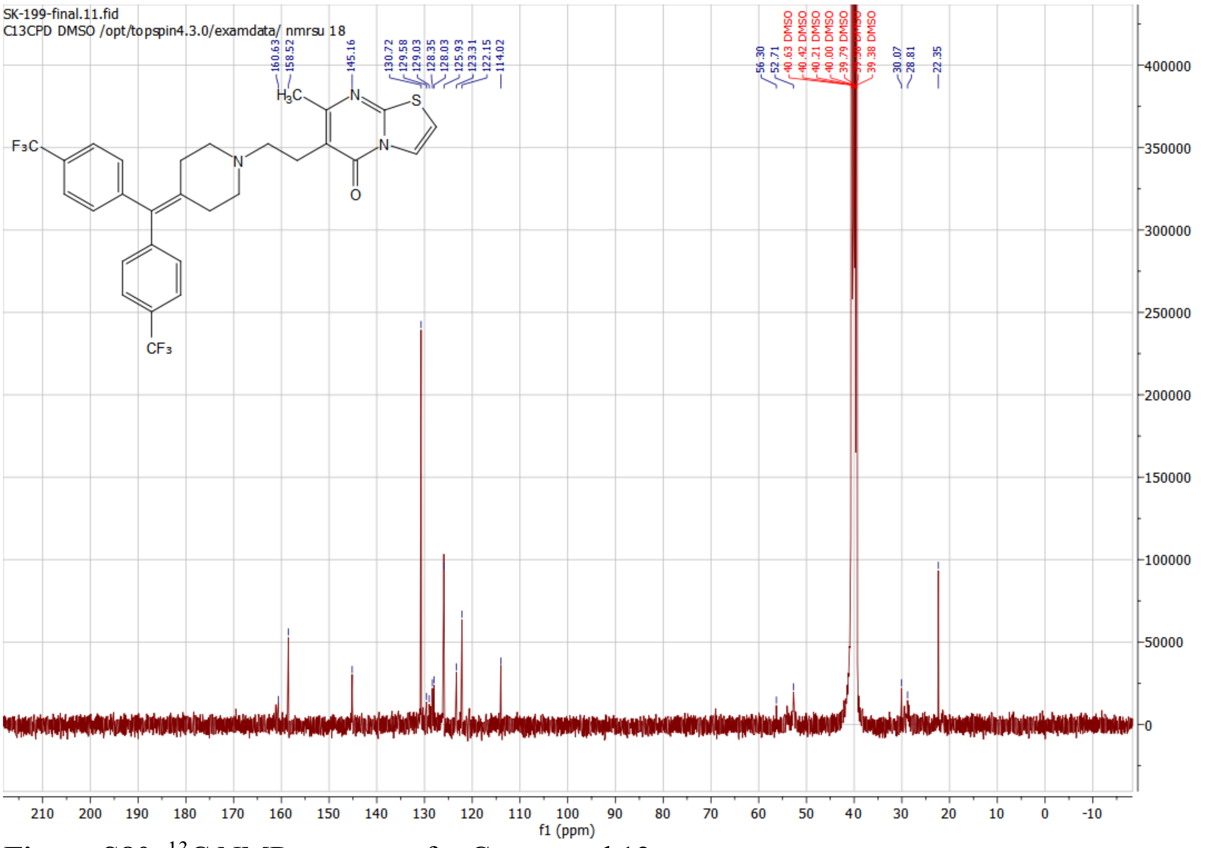

Figure S80. ^{13}C NMR spectrum for Compound 12.

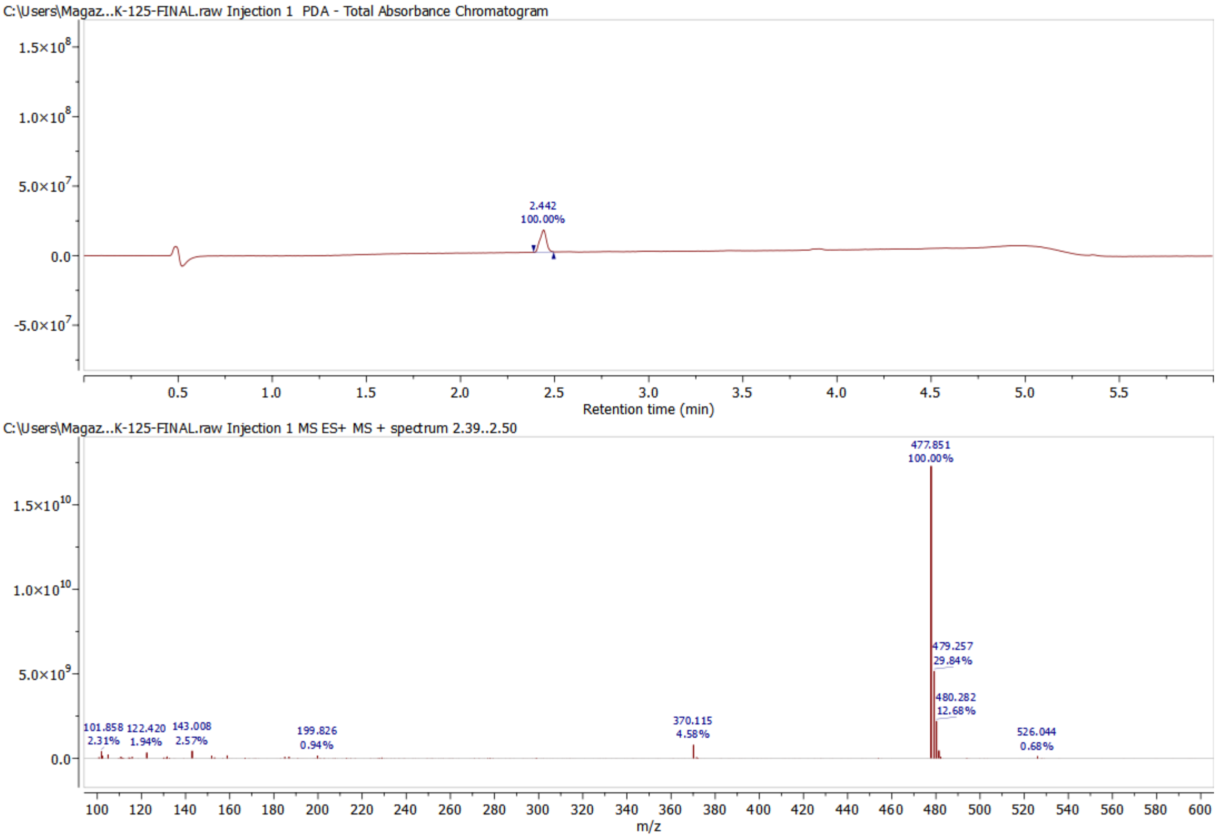

Figure S81. HPLC-MS trace and spectrum for Compound 1.

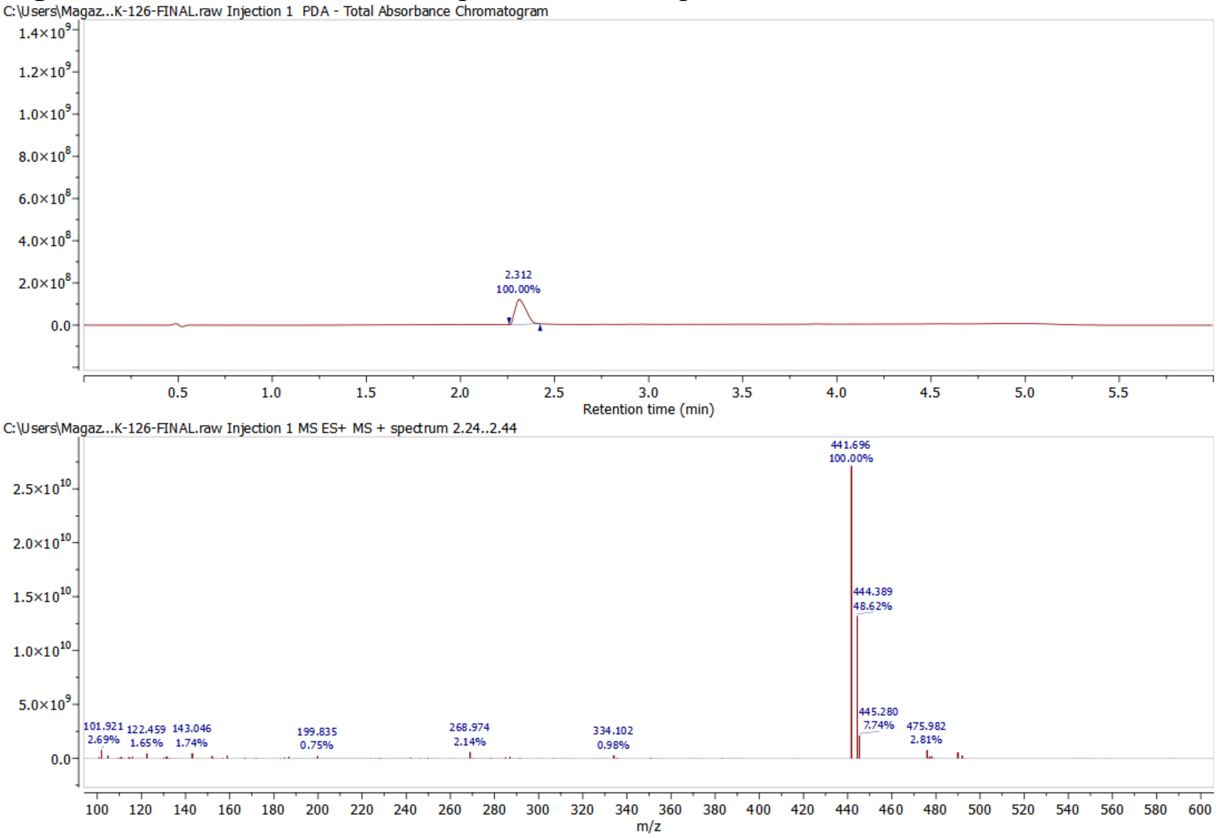

Figure S82. HPLC-MS trace and spectrum for Compound 2.

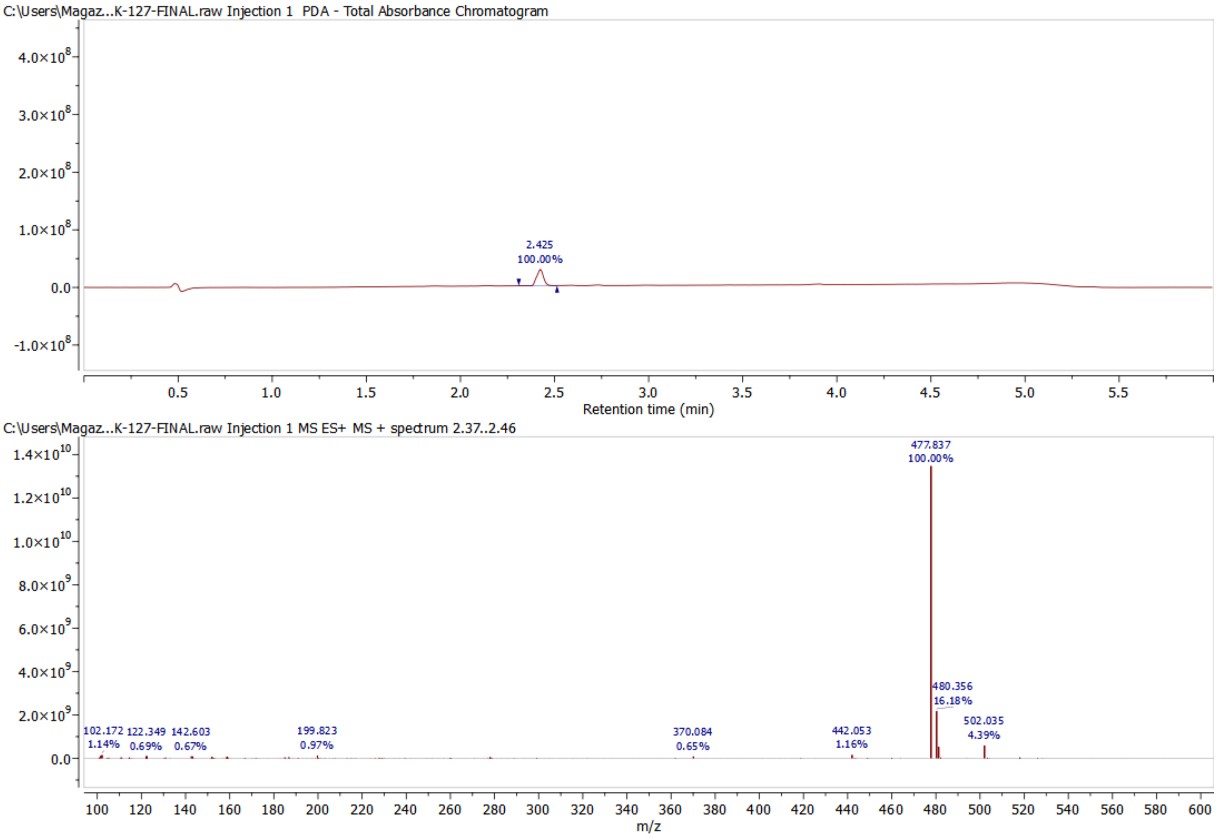

Figure S83. HPLC-MS trace and spectrum for Compound 3.

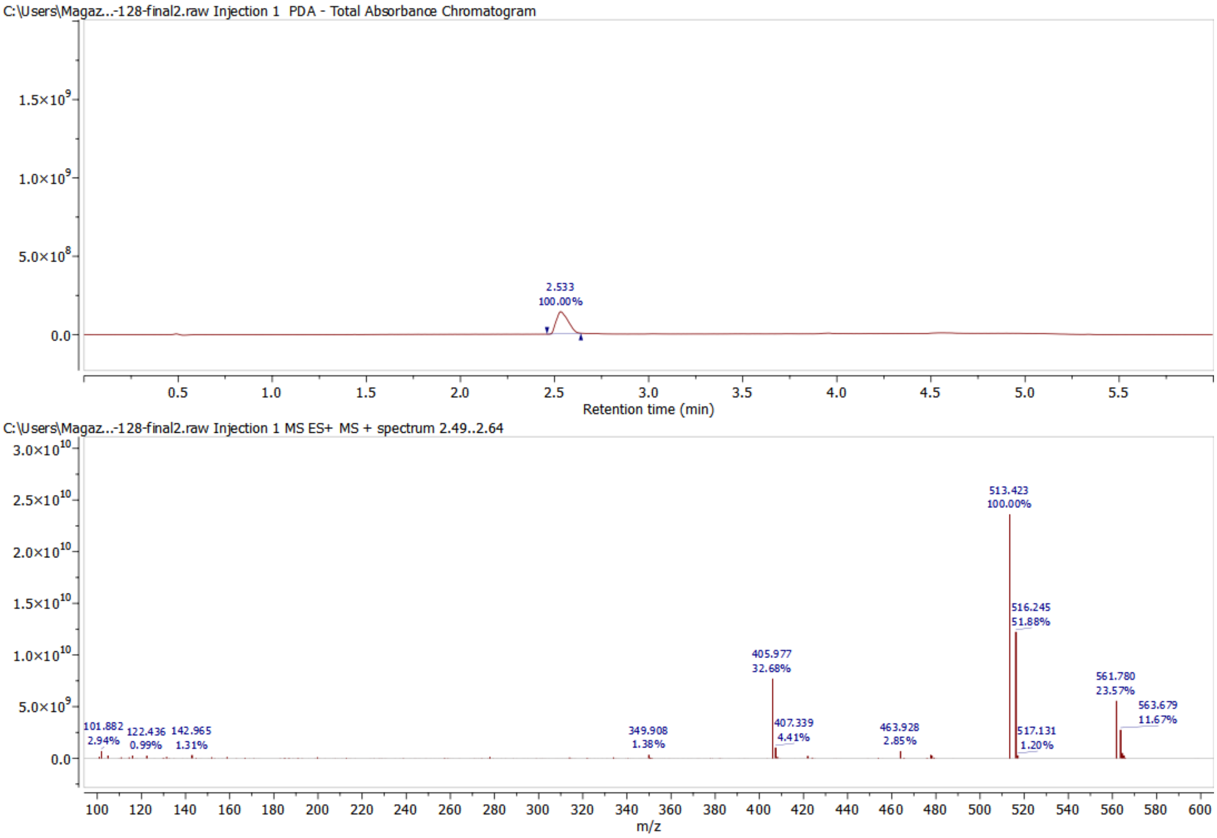

Figure S84. HPLC-MS trace and spectrum for Compound 4.

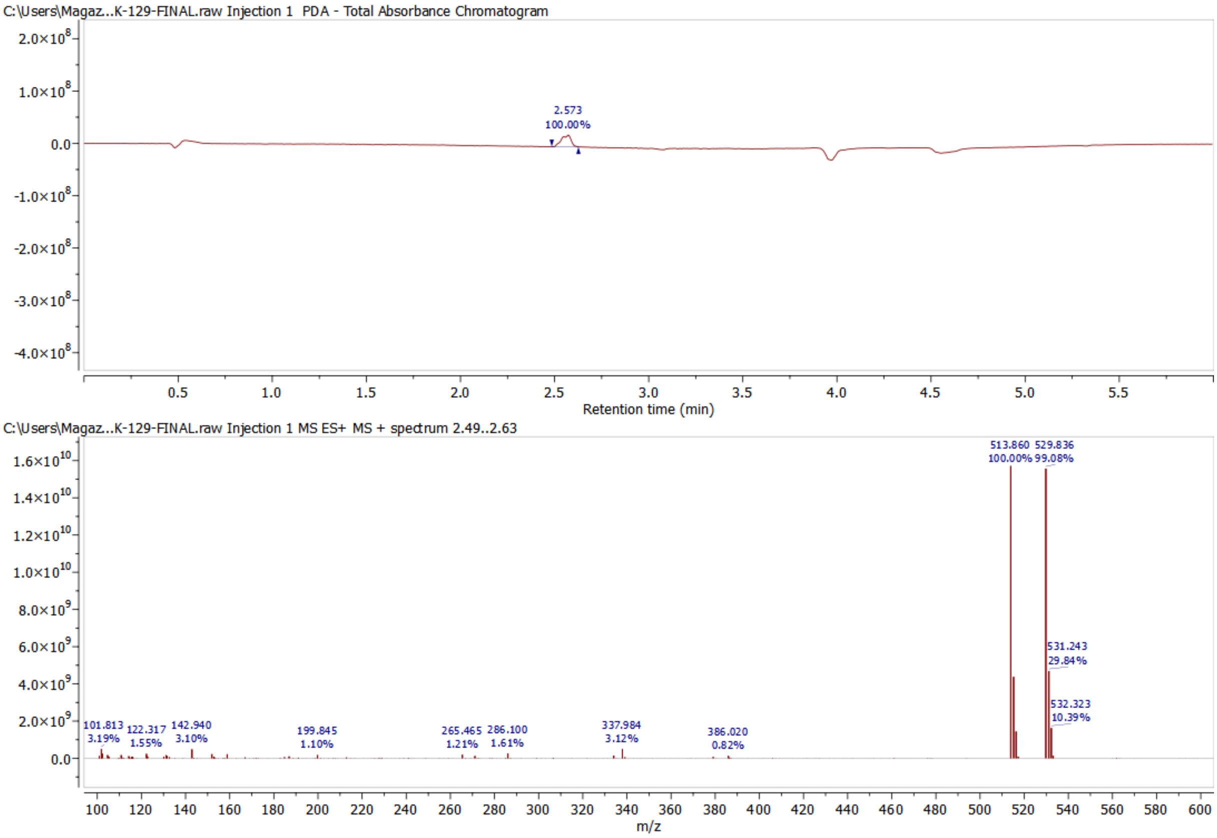

Figure S85. HPLC-MS trace and spectrum for Compound 5.

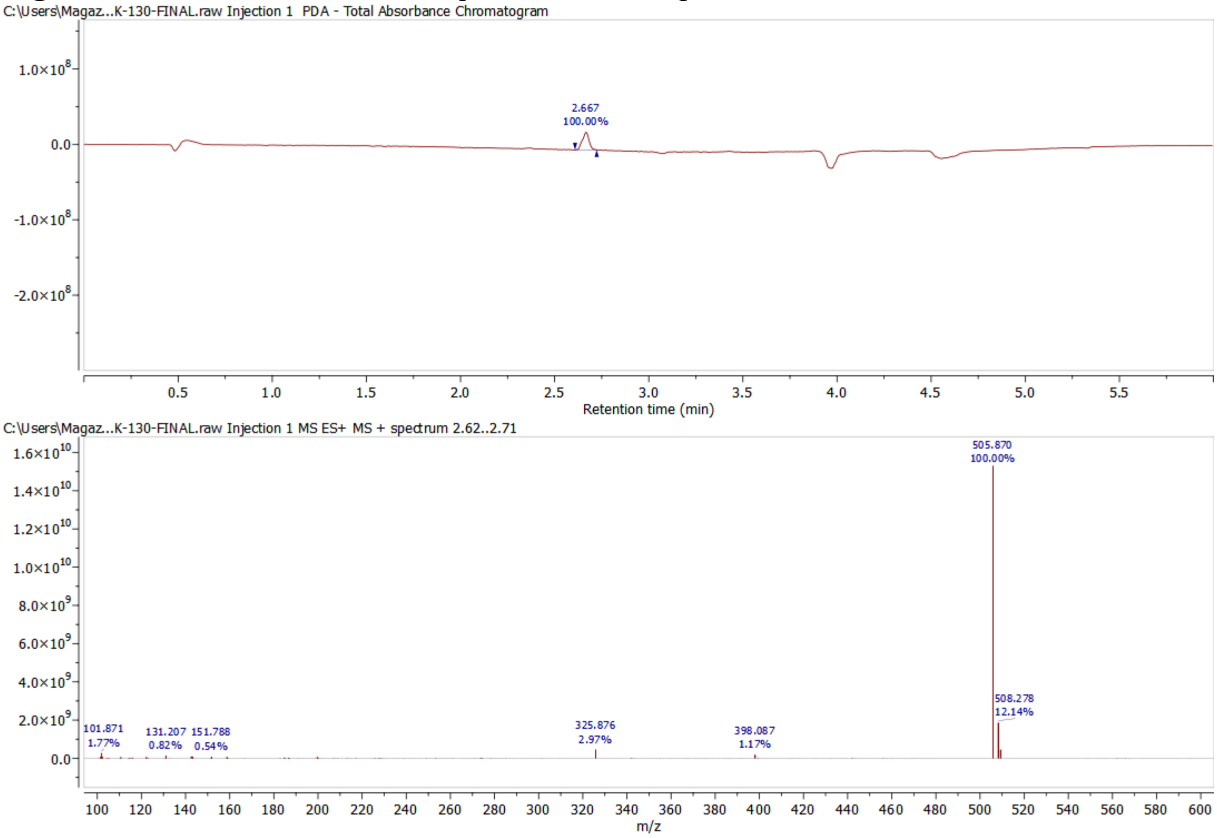

Figure S86. HPLC-MS trace and spectrum for Compound 6.

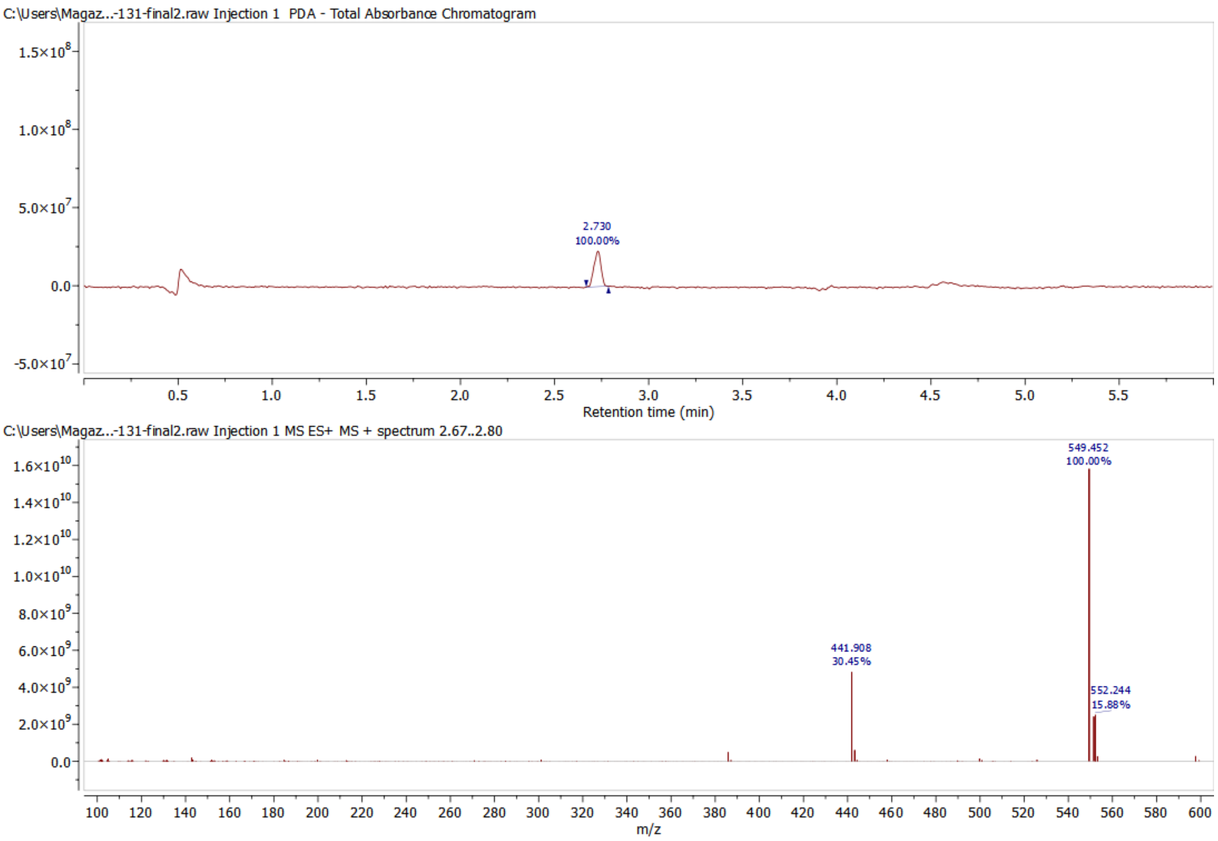

Figure S87. HPLC-MS trace and spectrum for Compound 7.

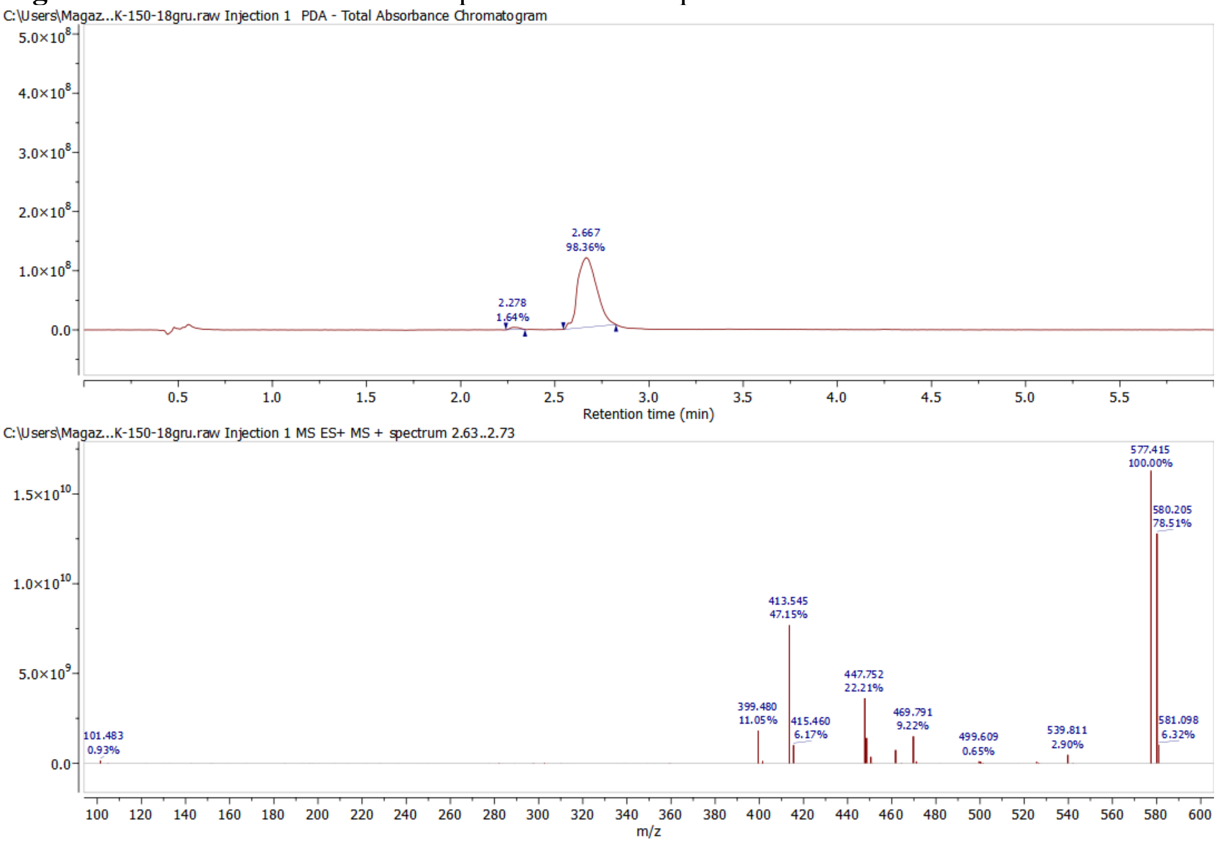

Figure S88. HPLC-MS trace and spectrum for Compound 8.

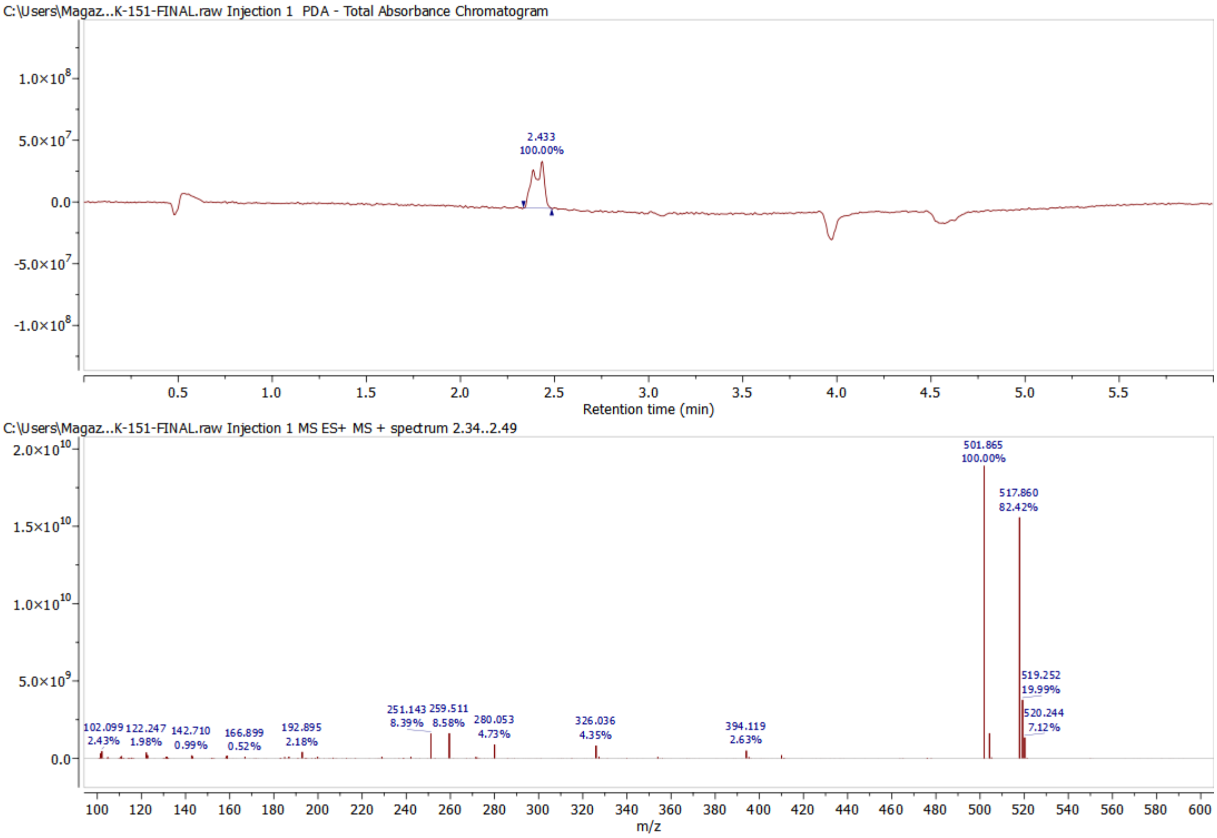

Figure S89. HPLC-MS trace and spectrum for Compound 9.

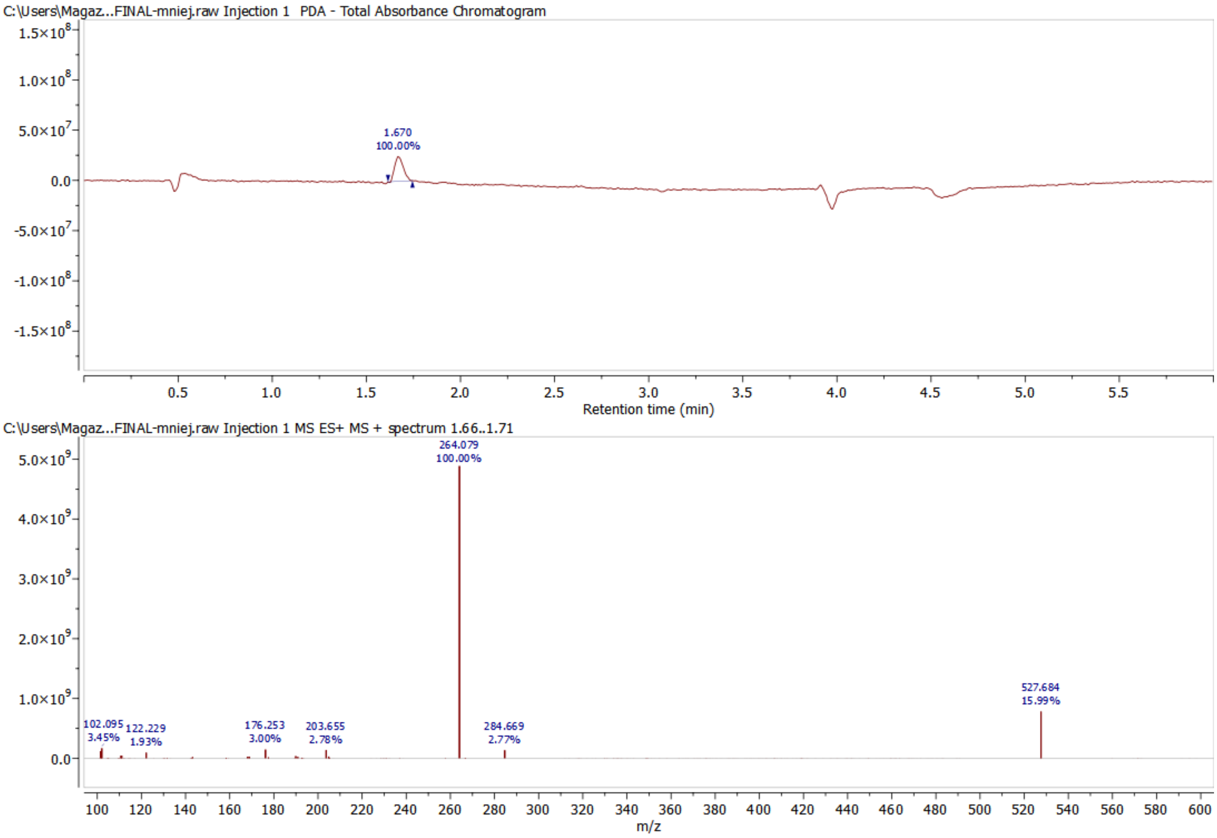

Figure S90. HPLC-MS trace and spectrum for Compound 10.

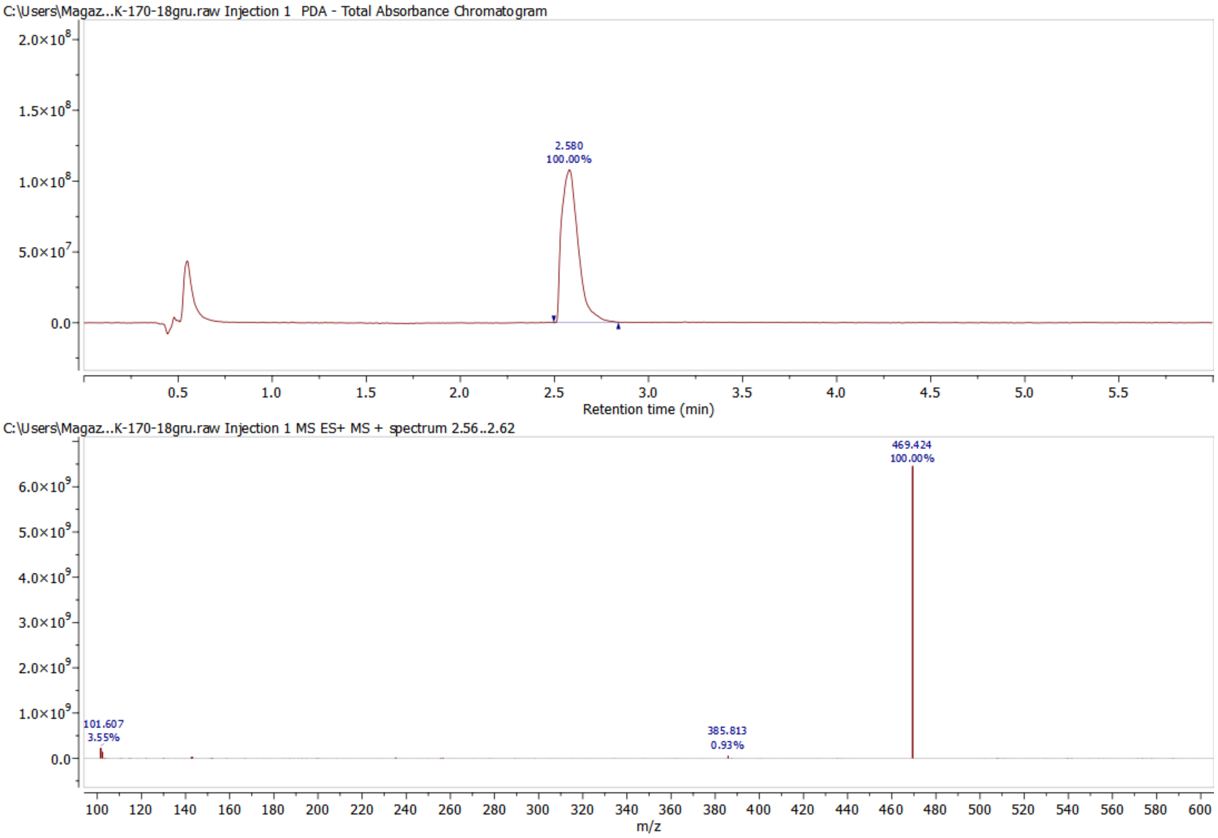

Figure S91. HPLC-MS trace and spectrum for Compound 11.

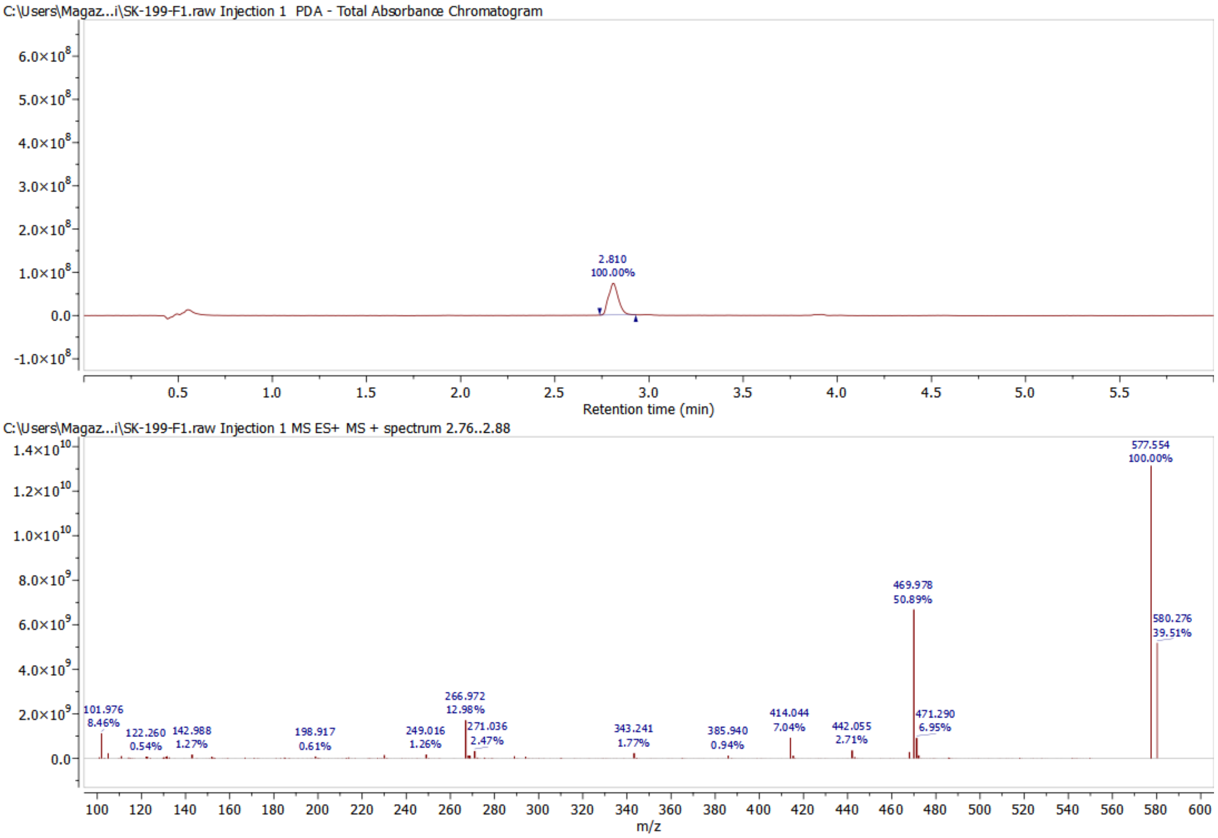

Figure S92. HPLC-MS trace and spectrum for Compound 12.

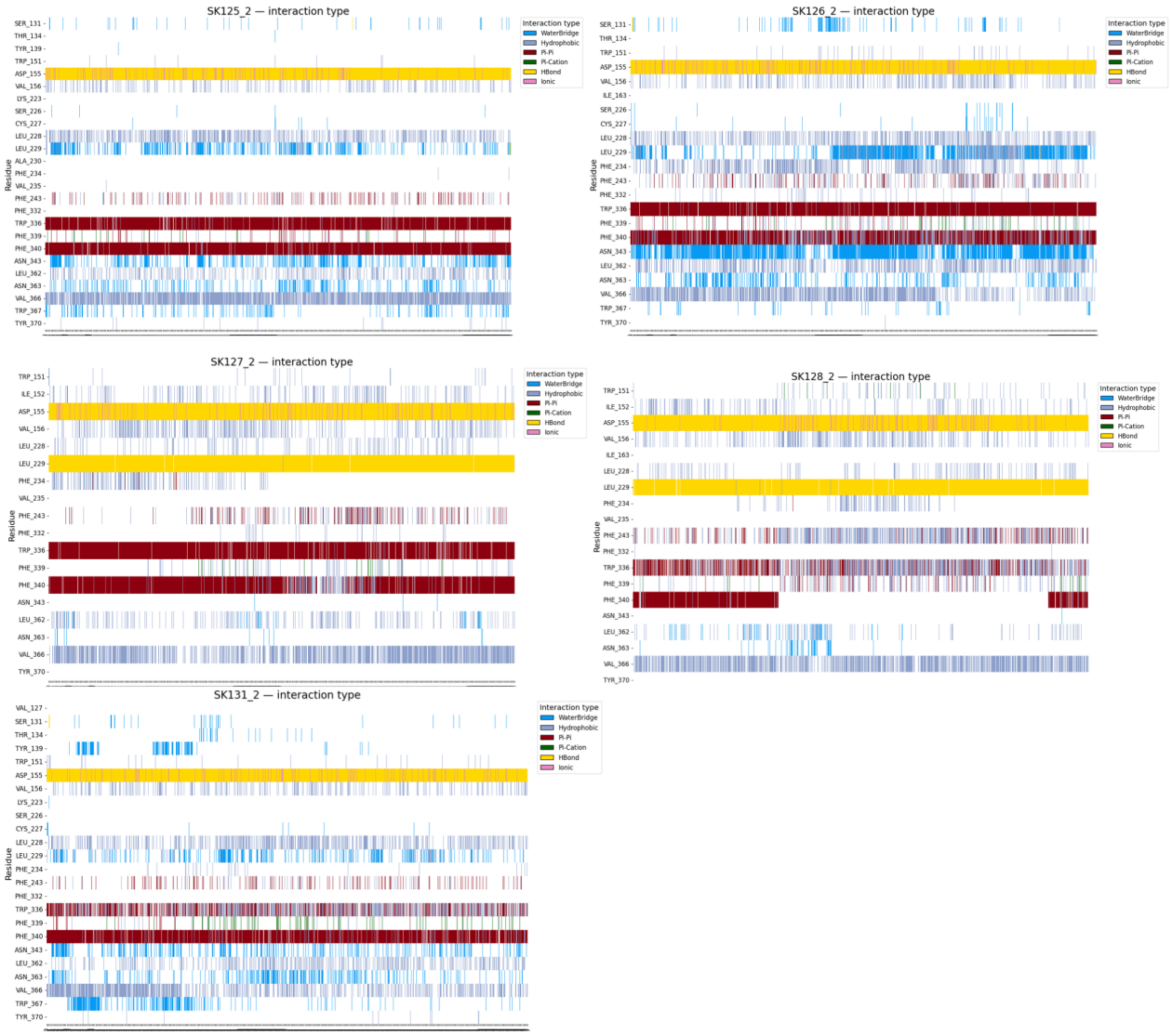

Figure S93. Results of the second MD simulation for the selected compounds; simulation time: 500 ns; 10,000 measurement frames.

Table S1. Representative competition association curves

**1 (SK125) ritanserin**	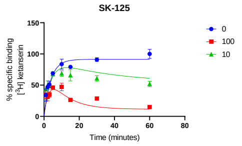
**2 (SK126)**	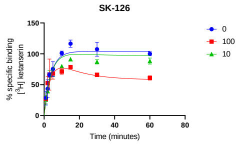
**3 (SK127)**	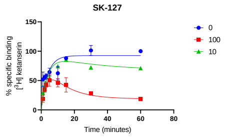
**4 (SK128)**	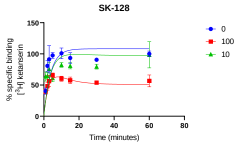
**5 (SK129)**	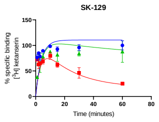
**6 (SK130)**	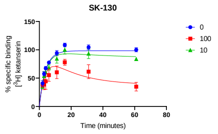
**7 (SK131)**	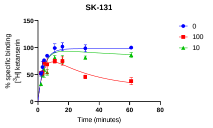
**8 (SK150)**	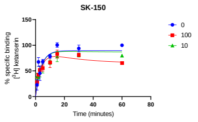
**9 (SK151)**	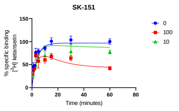
**10 (SK154)**	n/a
**11 (SK170)**	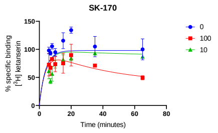
**12 (SK199)**	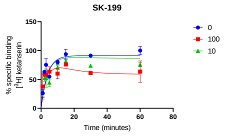
